# *Bumble*‐BEEHAVE: A systems model for exploring multifactorial causes of bumblebee decline at individual, colony, population and community level

**DOI:** 10.1111/1365-2664.13165

**Published:** 2018-05-22

**Authors:** Matthias A. Becher, Grace Twiston‐Davies, Tim D. Penny, Dave Goulson, Ellen L. Rotheray, Juliet L. Osborne

**Affiliations:** ^1^ Environment and Sustainability Institute University of Exeter, Penryn Campus Cornwall UK; ^2^ School of Biological and Chemical Sciences Queen Mary University of London London UK; ^3^ School of Life Sciences University of Sussex Sussex UK

**Keywords:** agent‐based modelling, *Bombus terrestris*, bumblebees, colony decline, cross‐level interactions, foraging, multiple stressors, pollination

## Abstract

1. World‐wide declines in pollinators, including bumblebees, are attributed to a multitude of stressors such as habitat loss, resource availability, emerging viruses and parasites, exposure to pesticides, and climate change, operating at various spatial and temporal scales. Disentangling individual and interacting effects of these stressors, and understanding their impact at the individual, colony and population level are a challenge for systems ecology. Empirical testing of all combinations and contexts is not feasible. A mechanistic multilevel systems model (individual‐colony‐population‐community) is required to explore resilience mechanisms of populations and communities under stress.
2. We present a model which can simulate the growth, behaviour and survival of six UK bumblebee species living in any mapped landscape. *Bumble*‐BEEHAVE simulates, in an agent‐based approach, the colony development of bumblebees in a realistic landscape to study how multiple stressors affect bee numbers and population dynamics. We provide extensive documentation, including sensitivity analysis and validation, based on data from literature. The model is freely available, has flexible settings and includes a user manual to ensure it can be used by researchers, farmers, policy‐makers, NGOs or other interested parties.
3. Model outcomes compare well with empirical data for individual foraging behaviour, colony growth and reproduction, and estimated nest densities.
4. Simulating the impact of reproductive depression caused by pesticide exposure shows that the complex feedback mechanisms captured in this model predict higher colony resilience to stress than suggested by a previous, simpler model.
5. *Synthesis and applications*. The *Bumble*‐BEEHAVE model represents a significant step towards predicting bumblebee population dynamics in a spatially explicit way. It enables researchers to understand the individual and interacting effects of the multiple stressors affecting bumblebee survival and the feedback mechanisms that may buffer a colony against environmental stress, or indeed lead to spiralling colony collapse. The model can be used to aid the design of field experiments, for risk assessments, to inform conservation and farming decisions and for assigning bespoke management recommendations at a landscape scale.

## Abstract 2

The *Bumble*‐BEEHAVE model represents a significant step towards predicting bumblebee population dynamics in a spatially explicit way. It enables researchers to understand the individual and interacting effects of the multiple stressors affecting bumblebee survival and the feedback mechanisms that may buffer a colony against environmental stress, or indeed lead to spiralling colony collapse. The model can be used to aid the design of field experiments, for risk assessments, to inform conservation and farming decisions and for assigning bespoke management recommendations at a landscape scale.

## 1 INTRODUCTION

World‐wide declines in pollinators, including bumblebees, are attributed to the chronic exposure of populations to a multitude of stressors such as habitat loss and resource availability, emerging viruses and parasites, exposure to pesticides, and climate change operating at various spatial and temporal scales (Baude et al., 2016; Goulson, 2015; IPBES, 2016; Kerr et al., 2015; Williams & Osborne, 2009). Disentangling the individual and interacting effects of these stressors and understanding their effects at the individual, colony and population level are a considerable challenge for systems ecology. Yet it is essential to inform policy and management recommendations to support pollinators and the pollination service they provide to crops and wild flowers (Vanbergen et al., 2013). Crone and Williams (2016) pointed out that in the case of bumblebees, this challenge is amplified by our lack of knowledge of their population dynamics. Despite being a well‐studied taxon, we have few estimates of colony densities in the landscape (Goulson et al., 2010; Osborne, Martin, Shortall, et al., 2008), and we do not have means of predicting future patterns of population change. This is primarily because of their annual and social life history and the difficulty of locating colonies and measuring reproductive success in the field. Added to this, the systematic empirical testing of the combined and synergistic effects of stressors on bumblebee colonies is largely infeasible (Becher, Osborne, Thorbek, Kennedy, & Grimm, 2013; Goulson, Nicholls, Botias, & Rotheray, 2015; Henry et al., 2017). We propose that a mechanistic multilevel systems model (individual‐colony‐population‐community) is required to explore the resilience mechanisms of bumblebee populations and communities under stress, and inform management decisions. We present such a model, *Bumble*‐BEEHAVE, and explain how it is radically different to other published bumblebee models.

Six contrasting bumblebee models have recently been published (Banks et al., 2017; Bryden, Gill, Mitton, Raine, & Jansen, 2013; Cresswell, 2017; Crone & Williams, 2016; Häussler, Sahlin, Baey, Smith, & Clough, 2017; Olsson, Bolin, Smith, & Lonsdorf, 2015). However, while useful in exploring the impact of individual stressors, such as food availability (Crone & Williams, 2016) or pesticides (Bryden et al., 2013; Cresswell, 2017), none as yet have the structural realism to incorporate multiple stressors or competition, operating at different organisational levels (individual or colony or population). They have limited flexibility to incorporate feedback mechanisms that may buffer the colony against environmental stress, or indeed lead to spiralling collapse. This mechanistic richness is essential for deep and broad understanding of risk (EFSA, 2015)—indeed Crone and Williams (2016) and Banks et al. (2017) noted that further processes and stage structure need incorporation. Most existing studies do not model multiple colonies (Bryden et al., 2013; Cresswell, 2017; Häussler et al., 2017; although see Banks et al., 2017) or capture the spatio‐temporal dynamics of resource availability (although see Häussler et al., 2017; Olsson et al., 2015; Polce et al., 2013) which are essential to make accurate predictions in real landscapes. Table 1 summarises the approach and capability of each model in contrast to the *Bumble*‐BEEHAVE model presented here.

**Table 1:** Comparison of aims, processes and output captured by different bumblebee models. + = explicitly included in the model, (+) = only implicitly included or authors state that this could be simulated, directly or indirectly, or, under Verification, that emergent patterns match empirical. Key to abbreviations: Differential Equations (Diff'ntial Eqns), Difference Equations (Diff Eqns), Agent‐Based Models (ABM), individual (Ind), colony (Col), population (Pop), colony founding queens (Qu), new offspring queens (q), males (m), workers (w), eggs (e), larvae (l), pupae (p), day/s (d), production (prod)

Comparator	Bryden et al. (2013)	Olsson et al. (2015)	Crone and Williams (2016)	Cresswell (2017)	Banks et al. (2017)	Häussler et al. (2017)	*Bumble*‐BEEHAVE
Type of model	Diff'ntial Eqns	Distance decay	Statistical, Diff Eqn	Matrix	Delay Diff'ntial Eqns	Process‐based	ABM, Monte Carlo method
Model aims to predict:	Impact of sublethal stress	Effect of landscape on flower visit rate and bee fitness	Impact of floral resources on col growth, q prod	Col demography and impact of pesticide and predation	Impact of many stressors on multiple Col growth	Effect of landscape on flower visit rate	Impact of many stressors on Ind, Col, Pop & community—with mapping
Main outputs	Col size, survival	Nest fitness, flower visit rates	Col size & mass, q prod, survival	Col size, reproduction, survival	Col size & composition, q & m prod, stores, survival	Col no., survival, flower visit rates	Behaviour, Col no., size & composition, stores, q & m prod, survival, flower visits
Scale							
Space (grid size/map size)		25 m/3 km				25 m/3 km	25 m/5 km
Time	Continuous		Discrete, 15 weeks	1 day steps, 40 days	Continuous, 120 days	2 flowering periods/year, 35 years	1 day steps, event‐based within day, 10 years
Organisational level:							
Individual level	(+)	+					+
Energy/nectar consumption		+			+		+
Colony level	(w)		Qu, q, m; mass	w, m, q	Qu,(l),w,m,q; nectar, pollen stores	Qu,q,w	Qu,e,l,p,w,m,q; nectar, pollen stores
Multiple Colonies					(+)	+	+
Population level					(+)	+	+
Multiple species							+
Stressors:							
Forage availability		+	+		+	+	+
Nest site availability		+				+	+
Pathogens/Parasites	(+)						(+)
Predation		(+)		+			+
Pesticide exposure	(+)			+	(+)		(+)
Weather/Climate							(+)
Competition emerges							+
Testing							
Sensitivity analyses			+		+		+
Verification	+		+	+	(+)	(+)	+

**Figure 1:**
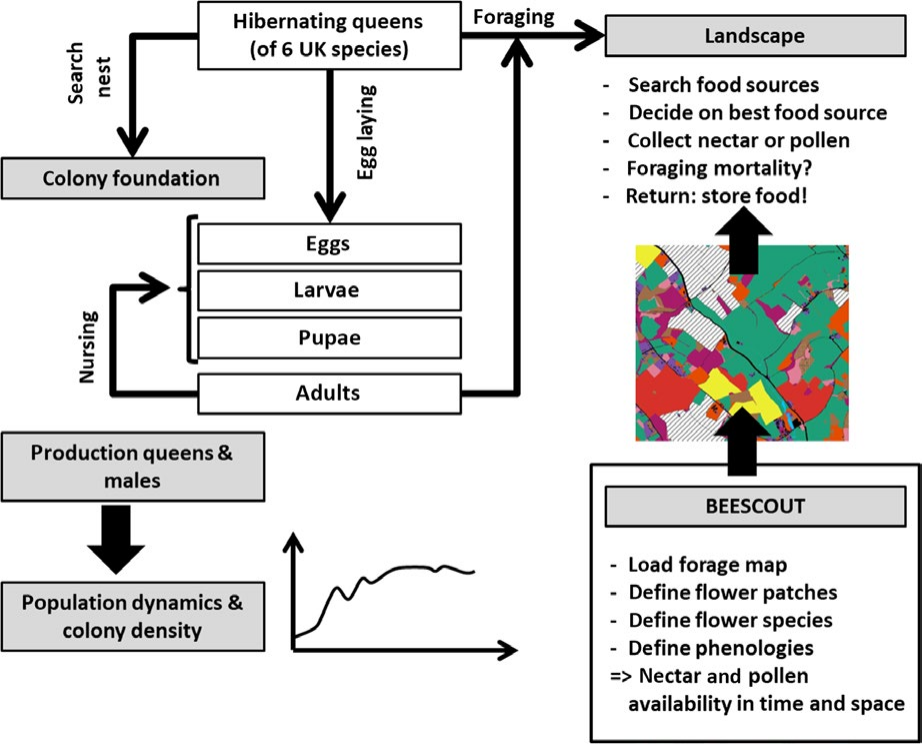
Overview of the *Bumble*‐BEEHAVE model structure. Starting with an initial number of hibernating queens, the colony, population and community dynamics of up to six UK bumblebee species can be simulated. In an agent‐based approach, nest search and colony foundation by the queen are modelled. Brood needs incubation as well as nectar and pollen to develop. Foraging takes place in a realistic landscape of crop or seminatural habitat patches in which a number of flower species provide nectar and pollen. Foraging efficiency of the bees depends on their size, tongue length and flower morphology. Successful colonies produce males and/or queens, allowing the model to run over a number of years [Colour figure can be viewed at wileyonlinelibrary.com]

*Bumble*‐BEEHAVE is an open source model (www.beehave-model.net) based on bumblebee behaviour and life history, designed to simulate colony growth and survival in any landscape where nectar and pollen sources can be approximated from maps with the intention of predicting the effects of multifactorial stressors on bumblebee survival at the individual, colony and population levels (Figure 1). We have taken a broadly similar approach to that used for development of the well‐used BEEHAVE model of honeybee colony dynamics (Becher et al., 2014; EFSA, 2015), incorporating our BEESCOUT model of bees searching for forage in landscapes (Becher et al., 2016) and including substantial Supporting Information. *Bumble*‐BEEHAVE is an agent‐based model (Grimm & Railsback, 2005) where individual behaviour is determined by stimuli and thresholds that scale up to colony‐ and population‐level processes. *Bumble*‐BEEHAVE is built on empirical data describing colony dynamics and foraging in realistic digitised landscapes. It has basic parameterisation for six common UK species, and is structured so that it can be updated as data for further life stage parameters become available. We present sensitivity analyses and compare simulations with empirical data to illustrate the potential of *Bumble*‐BEEHAVE in predicting (a) individual foraging behaviour, (b) colony growth and reproduction and (c) population nest density, in realistic landscape settings.

## 2 MATERIALS AND METHODS

### 2.1 *The Bumble‐*BEEHAVE model

Here we provide a condensed overview of the *Bumble*‐BEEHAVE model. The Supporting Information provides the complete, detailed description of the model, following the Overview, Design concepts, Details (ODD) protocol (Grimm et al., 2006, 2010), the scheduling of the procedures, lists of all variables, full explanation and references used for parameterisation (Appendix S03), and a user manual (Appendix S02). *Bumble‐*BEEHAVE itself is available in Appendix [Link], [Link], [Link] and free to download at www.beehave-model.net. To ensure it is suitable for a wide range of users, it is implemented using the free open source software platform NETLOGO (5.3.1; Wilensky, 1999) and licensed under the GNU General Public Licence (Appendix S09).

### 2.2 Purpose

The purpose of the model is to explore the colony and population dynamics of bumblebees as a result of the spatial and temporal distribution of resources. It also has the potential for use in understanding risks of pathogen prevalence and pesticide exposure. Weather and/or foraging conditions, predation by badgers and social‐parasitism from cuckoo bees are implemented in a relatively simplified way in the current version of the model, but could be developed in later versions.

*Bumble*‐BEEHAVE simulates, in an agent‐based approach, the life cycle of bumblebees, foraging for nectar and pollen from a variety of plant species in a spatially explicit landscape (Figure 1). Starting with an initial number of hibernating queens of up to six European bumblebee species, the foundation of nests in suitable habitat, and raising of brood by the queen, and later by worker bees, is modelled (parameterisation in Appendix S04). The population dynamics then result from the number of reproductives, particularly queens, produced by colonies of the same species. Here we focus simulations on *Bombus terrestris* L. but if several species are included in the simulation then community dynamics also emerge.

### 2.3 Environment

Time in the model proceeds in daily steps, during which bees can perform different tasks of various durations. The modelled landscape comprises a number of food sources, seasonally providing nectar and pollen of varying quality and quantity and can be created using the BEEHAVE landscape module BEESCOUT (Becher et al., 2016). Weather is not explicitly implemented in the model but it is represented by specifying the daily allowance of foraging hours (i.e. the maximal time foragers can spend every day on foraging). Furthermore, climate and weather conditions are implicitly taken into account by the phenology of flower patches and the timing of queen emergence from hibernation. Optionally, predation by badgers can be simulated by distributing badger setts in the landscape and, with a certain probability, destroying colonies within the foraging range of the badgers.

### 2.4 Bees

Each “bumblebee” in the model represents either a single individual or a 1‐day age cohort. Adult queens are always implemented as individuals. Bees differ in their age, caste (worker, queen, male or undefined), their activity and their size (which affects their tongue length and forage loads). Furthermore, bees belong to a defined species and are member of a certain colony (except for hibernating and nest searching queens).

Bumblebee species in the model differ in the number of eggs laid by the queen (batch size), durations and weights of developmental stages, tongue lengths (and hence the floral rewards that are available to them), suitable nesting habitat and period of emerging from hibernation. Parameterisation (and associated references) for the six most common bumblebee species in the UK and for a generic cuckoo bee are provided in Appendix S04.

### 2.5 Model processes

A simulation starts on the first of January with an initial number of hibernating queens for each bumblebee species. After emergence, queens need to find a nest site in a suitable habitat, which can take several days. If they are successful, they collect and store nectar and pollen before laying their first batch of eggs. The brood needs to be incubated and larvae additionally need to be fed. Once the first batch of larvae has developed into pupae, a second batch of eggs can be laid. When the first adult workers emerge, the queen stops foraging and specialises in egg laying. The activities of bees are based on stimuli in the colony and individual thresholds (See Appendix S03 ODD: p. 14—Tasks and activities; p. 76—ActivityProc) for each of the three main tasks:

1. Egg laying: eggs are produced in batches (e.g. *B. terrestris* lays 12) and can be male or female, with female brood either developing into workers or queens.
2. Nursing: reflects the time a bee spends on the brood for incubation and feeding.
3. Foraging (for nectar or pollen): foraging bees leave the colony to collect food.

While naive bees first have to find a food source, with the detection probability depending on the distance to the colony, experienced bees typically know a number of food sources already. Successful foragers remove the collected nectar or pollen from the food source (which is replenished overnight), and return it to the colony's stores. Depending on the duration of the foraging trip and the foraging mortality per second, the survival of the forager is determined at each trip. The foraging choices of the bees are based on efficiency, which decreases during the day as the food source is depleted. Flower handling times depend on the flower specifications and the bee's tongue length (Harder, 1983) and affect the probability that a foraging bee switches to (or searches for) a more profitable food source (Appendix S03, ODD: p. 16—Foraging).

Eggs require a species‐specific minimum age and minimum incubation energy to hatch. As larvae, they need to be fed and will develop into pupae when they reach a certain minimum age, minimum weight and when summed minimum incubation energy has been received. A larva will develop into a worker, unless the colony reaches conditions appropriate for queen production (Appendix S03, ODD: p. 34—Production of males and queens) and the larva has already reached a species‐specific minimal weight. Pupae finally develop into adults, when they reach a minimum age and summed amount of incubation energy received. The weight a bee has gained during larval development determines its size and hence the size of its honey stomach, the size of pollen pellets that can be collected, and the proboscis length, affecting its foraging efficiency (Appendix S03, ODD: p. 49—CropAndPelletSizeREP; p. 106—ProboscisLengthREP). If bees are unable to proceed to the next developmental stage within a certain time frame, they die.

The timing of queen production in the model is derived from data on *B. terrestris* by Duchateau and Velthuis (1988). At the beginning of the colony development, female larvae develop into workers, whereas later, they may develop into queens. The onset of queen production follows, with *c*. 5 days of delay, the queen's switch from laying diploid eggs to haploid, male eggs. However, this requires also a sufficient number of workers relative to larvae (larvae to worker ratio less than 3) in the colony. Diploid larvae of 3 days of larval age can then develop into queens instead of workers.

As soon as young queens have developed into adults, they leave their mother's colony and mate with an adult male. They then go into hibernation and will not be active until they emerge in the following year.

### 2.6 Key output of the model and emerging patterns

Outputs and patterns can emerge at all organisational levels:

1. Individual level: bee activities and foraging decisions (when and where to go in the landscape, which plants they exploit) emerge as a result of the needs of a colony and the resources available in the landscape. Bee life spans emerge from their individual behaviour (mainly time spent foraging) and the colony performance.
2. Colony level: colony dynamics, number and sex ratio of reproductives produced emerge from the activities of colony members and resources available in the landscape.
3. Population level: the number of hibernating queens shaping the population dynamics, genetic diversity, and overall sex ratios emerge from colony performances and individual behaviour of the bees.
4. Landscape level: the number of visits to the various food sources (flower patches and flower species), the locations where colonies produced males and queens, and the colony densities emerge, again based on colony performances and individual bees' behaviour.

### 2.7 Default settings

All simulations were run using the default settings (Appendix S04) unless stated otherwise. Simulations start on 1 January with a user‐defined number of *B. terrestris* colonies and number of days (see Appendix S10 for simulation settings). Simulations were run using the RNetLogo package (Thiele, 2014) in r (version 3.2.3, R Core Team, 2015).

### 2.8 Model inputs

#### 2.8.1 Realistic spatially explicit forage landscapes

Creation of the realistic spatially explicit forage landscapes required the combination of digitised landscape maps, a habitats input file of flower species composition in the different habitats and crop types, calculated flower patch characteristics (size and distance from colony), average patch detection probability (using BEESCOUT) and a flower species input file of resource characteristics (nectar and pollen quantity, quality and availability) (Appendix S03, ODD: p. 27—Input data).

One 25‐km^2^ digitised landscape map of Sussex, UK was created in ArcMap (Version 10.2) consisting of suitable nesting habitat and sources of pollen and nectar. Polygon data from Land Cover Map 2007, Ordnance Survey and Google Maps were used to classify habitats that provided suitable nesting habitat (Appendix S03, ODD: p. 32—Searching nests) and floral resources for bumblebees, and included permanent grassland, seminatural scrub, hedgerows and woodland (gardens were not included in this first stage as pollen and nectar data are not available, but can be incorporated when data allow). Hedgerows were manually digitised using Google Earth. The location of mass flowering crops considered as sources of nectar and/or pollen of oilseed rape, field beans and maize (pollen only) was recorded from field surveys of the landscapes during 2014. Areas categorised as manmade (e.g. buildings and roads), freshwater, cereal crop and bare ground were assumed to be devoid of resources. These maps were converted to Raster 25‐m grid cells and then converted to Ascii text files to be used as map input files for BEESCOUT (version 2.0, Appendix S05).

The habitats input file was created using the flower species abundance per m^2^ of 34 major bumblebee forage plants and the three mass flowering crops in the different flower patch types which were identified and surveyed in the field. Flower patch characteristics were calculated as the area, *X* and *Y* coordinates, and detection probability of each patch, and the flower species input file was created using the quantity of nectar (ml) and pollen (g), quality of nectar sugar (mol/l) and pollen (percentage protein), available during the specified flowering period of the different flower species and their phenology and morphology (Appendix [Link], [Link], [Link], [Link], [Link], [Link], [Link], Fowler, Rotheray, & Goulson, 2016; R. E. Fowler, E. L. Rotheray, & D. Goulson, unpublished data). Then the habitat input file and flower patch characteristics were combined to create the *Bumble*‐BEEHAVE input text file and a new compatible map image file.

Detection probability (Becher et al., 2016; Appendix S03, ODD: p. 88—DetectionProbREP) for each patch was calculated from its distance to the colony using BEESCOUT (version 2.0, Appendix S05) and assuming approximate maximal foraging range of 758 m for *B. terrestris* (Knight et al., 2005).

### 2.9 Model testing

#### 2.9.1 Verification of the code

The model code was checked throughout all stages of model development by both developers (MB, TP). Visual testing was performed using the *Bumble*‐BEEHAVE output plots (graphs showing the emerging results of the model) to verify model behaviour. “Assertions” are included at various locations in the code to halt a simulation run if state variables go beyond a defined range.

#### 2.9.2 Sensitivity analysis

We examined *Bumble*‐BEEHAVE model sensitivity to biologically relevant parameters defined as numeric, noninteger global variables (either on the interface or the code) with a Default value of less or more than zero. For each run, we multiplied a parameter's Default value by either 0.5, 0.75, 1, 1.25, 1.5 or 2 separately and left all other parameters at Default values. Each combination was run for 1 year, 20 different times (aka Random Seeds), and the number of queens and males produced at the end of each run were recorded (full results in Appendix S08).

#### 2.9.3 Empirical testing of the model

We compared graphical outputs of *Bumble*‐BEEHAVE simulations with empirical data at the individual level (Stelzer, Stanewsky, & Chittka, 2010), colony level (Duchateau & Velthuis, 1988; Duchateau, Velthuis, & Boomsma, 2004; Gosterit & Gurel, 2016; Lopez‐Vaamonde et al., 2009) and at the population level (Knight et al., 2005). For clarity, we present the setup of the simulations in the result section. We do not present statistical analyses since the data on the environmental variables underpinning the empirical results, e.g. forage availability in the landscape, are not available so the model cannot be calibrated exactly to those conditions. It is therefore most appropriate to describe data trends and match patterns (Grimm & Railsback, 2005).

#### 2.9.4 Model applications

To illustrate the applications of *Bumble‐*BEEHAVE, we determined the number of colonies supported by habitats with differing forage quantity and quality. These could then be used to estimate *B. terrestris* colony densities in any landscape based on the areas of seminatural habitats of grassland, hedges, scrub or woodland. We also simulated the impact of a reduction in colony foundation on population dynamics as a potential effect of pesticide exposure.

## 3 RESULTS

### 3.1 Sensitivity analysis

We comment on the three most sensitive parameters here: further details are in Table 2 and Appendix S08. Similar to the honeybee model BEEHAVE, *Bumble*‐BEEHAVE is most sensitive to changes in the probability of foraging mortality as it directly affects the work force and food influx of the colony.

**Table 2:** The complete sensitivity analysis can be found in Appendix S08. We present the difference in the number of queens (Δ queens) and males (Δ males) produced, calculated as default value × 2—default value × 0.5, e.g. when *ForagingMortalityFactor* (default 1) is set to 2, 1,239 queens less are produced than when it is set to 0.5. Parameters are sorted by their impact on the number of queens produced (Δ queens). Δ (males/queens) described how the sex ratio is affected, with negative numbers indicating a smaller proportion of males. Under default setting, 590.4 hibernating queens and 757.2 adult males are produced (ratio m:q = 1.3)

Parameter (default value)	Description	Δ queens	Δ males	Δ (males/queens)
ForagingMortalityFactor (1)	Factor to modify the foraging mortality	−1,239	−1,796	−0.17
QueenDestinedEggsBeforeSP_d (5 days)	Max. days before switch point when queen destined eggs may be laid	853	−953	−6.24
NestSearchTime_h (6 hr)	Time a queen spent on searching for a nest site per day	−473	−439	0.22
DailySwitchProbability (0.13)	Daily probability that a queen switches to lay haploid eggs (only if larvae:worker ratio is <3)	−448	579	1.60
Lambda_detectProb (−0.005)	From BEESCOUT: describes how detection probability of a food source increases with distance	247	466	0.19
Weather (8 hr)	Constant, daily foraging allowance	239	458	0.05
AbundanceBoost (1)	Factor to modify the amount of nectar and pollen at each food source	203	310	0.09
LarvaWorkerRatioTH (3)	max. larvae:worker ratio under which switching to lay haploid eggs and queen production is possible	172	−550	−1.05
EnergyRequiredForPollenAssimilation_kJ_per_g (6.2 kJ/g)	Energy required to digest and assimilate proteins from pollen consumed	145	−866	−2.66
ForagingRangeMax_m (758 m)	Maximal foraging distance	−125	−350	−0.26
FoodSourceLimit (25)	Approx. number of trips a food source must be able to supply with nectar or pollen, otherwise it is removed	121	232	0.13
MetabolicRateFlight_W/kg (488.6 W/kg)	Metabolic rate during flight (depends on weight of bee)	−86	−211	−0.16
MaxLifespanMales (30 days)	Maximal lifespan (days) of male bumblebees	47	−5	−0.11
EnergyFactorOnFlower (0.3)	Reduces energy spent on flying while a bee is in a flower patch	39	−48	−0.16

Parameter *QueenDestinedEggsBeforeSP_d* defines when the colony starts to raise queens rather than workers, relative to the switch point (when the queen lays haploid instead of diploid eggs). While an earlier onset of queen production increases the number of queens, it reduces the number of males produced and hence has the biggest impact on the sex ratio of all parameters tested. *NestSearchTime_h* describes the time in hours a queen spends on searching a nest site per day, which is associated with a high mortality.

### 3.2 Empirical testing of the model

#### 3.2.1 Individual‐level comparison

##### Setting

We compared modelled individual forager behaviour to that measured by Stelzer et al. (2010) who recorded foraging trip duration for all foraging flights of one individual. We ran simulations with 7,500 initial *B. terrestris* queens to increase the competition; other settings were kept at default. (*n* = 1 simulation run, 365 time steps; Appendix S10).

##### Output

**Figure 2:**
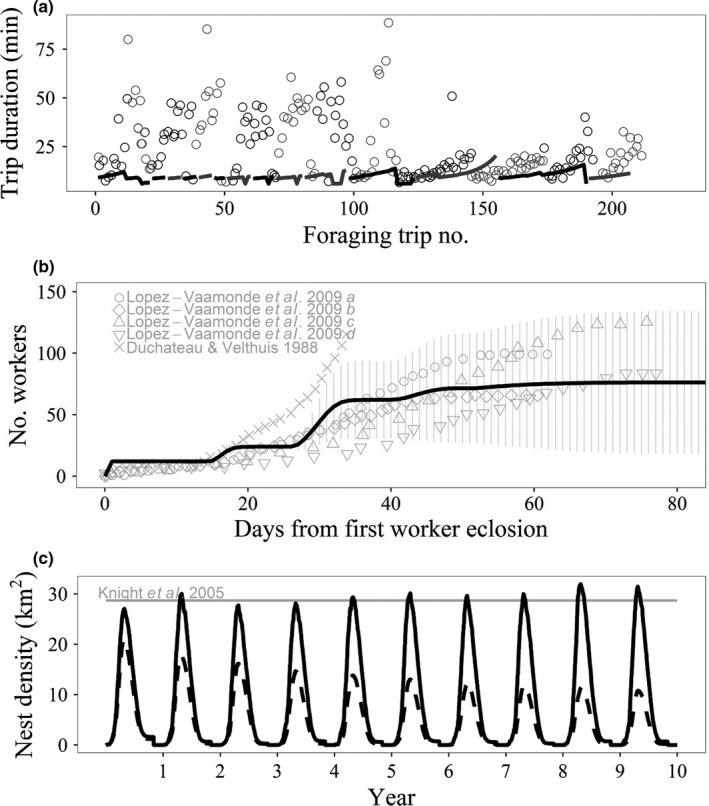
Individual, Colony and Population comparison of *Bumble*‐BEEHAVE model simulations of *Bombus terrestris* to empirical data. (a) Foraging trip duration for all foraging trips made by one individual bee (solid lines) compared to empirical data (open circles) from Stelzer et al. (2010). Trips during the same day are shown in the same colour and colours alternate daily between black and grey. (b) Number of workers (mean ± *SD*) produced since first worker eclosion (solid line) compared to empirical data from Duchateau and Velthuis (1988) and Lopez‐Vaamonde et al. (2009). Lopez‐Vaamonde et al. (2009) provided two datasets (a, b and c, d) and distinguished colonies producing queens (a, c) or not (b, d). (c) Nest densities over 10 years compared to empirical average of 28.7 nests per km^2^ (grey arrowed line) from Knight et al. (2005), for realistic landscape (solid line) and when applying the Baron et al. (2017; dashed line) pesticide exposure effect on reproduction, resulting in 26% of emerged queens being unable to found a colony

We selected the first bee that had foraged (>5 min) for at least 200 trips and plotted the foraging trip duration against the foraging trip number for that individual to compare to Stelzer et al. (2010). Figure 2a shows that the increasing durations of foraging trips throughout each day in the model and empirical data follow a similar pattern, although the absolute trip durations in the empirical data are considerably higher until ca. trip 115, where the experimental bee (as suggested by Stelzer et al., 2010) might have found a rich food source close to the colony. This could either indicate that the modelled landscape is rather beneficial for the bees or that the handling times are somewhat underestimated.

#### 3.2.2 Colony‐level comparison

##### Setting

For colony‐level comparison, we compared modelled colony investment in queen production and the days when colonies produced queens, switched to producing males and when workers started to lay their own eggs to data from Duchateau et al. (2004), Duchateau and Velthuis (1988) and Gosterit and Gurel (2016) (*n* = 7,500 simulation runs, 365 time steps; Appendix S10).

We compared the number of workers produced by a colony with empirical datasets from Duchateau and Velthuis (1988) and Lopez‐Vaamonde et al. (2009). These experimental colonies were located in a (climate) room but had access to either the outside environment or a flight arena and received additional food at least during colony initiation.

We simulated the colony development in the realistic landscape with one initial *B. terrestris* queen, implemented in the fully individual‐based mode (Appendix S03 ODD, p. 66—CreateColoniesProc). Simulations ran for 365 days with 7,500 replicates (Appendix S10). We recorded the days of major colony events and developmental traits of the colony, including the number of males and queens produced to calculate the colony investment in queens (Table 3).

**Table 3:** Results of simulations in a realistic landscape compared to empirical data from literature (Duchateau & Velthuis, 1988; Gosterit & Gurel, 2016). Mean (±*SD*) of each output per colony or replicate is given. *At the colony level: n*_colony_ = number of replicates where workers were produced; colony establishment prob = *n*_colony_/7,500; Colony foundation = day on which colony was founded by the queen; Worker eclosion = the first day on which workers emerge (i.e. eusocial phase); queen production = first day new queens are produced; switch point = first day male eggs are produced; competition date = when workers lay their own eggs. The average values for total colony weight gain (Weight gain) and the average total numbers of brood (Eggs), (Larvae) and (Pupae) produced by the colony were calculated on day 365. The number of reproductives (males and queens) (Duchateau & Velthuis, 1988 E = Early male production; L = late male production); and the sex ratio when using Queen investment conversion of 1.69 (Duchateau et al., 2004). *At population level:* mean (±*SD*) nest density per km^2^ for the realistic landscape is shown. *N*_replicates_ = number of replicates and compared to Knight et al. (2005)

Measure	Simulations mean (±*SD*)	Empirical data (*M* ± *SD*)	
*Colony*		Duchateau and Velthuis (1988)	Gosterit and Gurel (2016)
*n*_colony_	919		25–41
Colony establish prob	0.12		
Colony foundation (day)	95.6 (27.0)		
Worker eclosion (day)	119.3 (27.0)		
After foundation/initiation	23.7	21	33.4 (5.3)
Queen production (day)	125.1 (33.0)		
After eusocial phase	5.8		7.9 (11.4)
After foundation/initiation	29.5		30.4^a^
Switch point (day)	129.0 (31.2)		
After eusocial phase	9.7	E: 9.8 (2.4), L: 23.4 (4.6)	−6.42 (14.9)^b^
After foundation/initiation	33.4		16.1^a^
Competition point (day)	138.3 (32.9)		
After eusocial phase	19.0	E: 29.6 (4.0), L: 32.0 (5.2)	
After foundation/initiation	42.7	52	
Weight gain (g)	111.5 (36.7)		
Workers (no.)	76.2 (57.5)	E: 136.9 (58.8), L: 284.3 (145.0)	86.3 (50.9)
Eggs (no.)	379.3 (124.8)		
Pupae (no.)	118.6 (55.3)		
Larvae (no.)	119.5 (55.0)		
Males (no.)	21.8 (18.7)	E:164.5 (130.4), L: 70.4 (89.7)	30.1 (28.2)
Queens (no.)	19.1 (19.1)	E: 9.5 (19.1), L: 55.8 (72.8)	24.8 (15.8)
Queen investment 1.69	0.46	E: 0.06, L: 0.44	0.45
*Population*			Knight et al. (2005)
*n*_replicates_	3		
Max. nest density (colonies/km^2^)	34.31 (2.4)		28.7 (range 26.6–30.7)

*Note*.

a Calculated from table II in Gosterit and Gurel (2016).

b 6.42 days *before* eusocial phase.

##### Output

For colonies where workers were produced (919), the average simulated timings of colony events, such as the day when a colony produces queens, switches to producing males or when workers start laying their own eggs, are approximately in the range of those reported in the literature (Table 3), although there is strong variation in reported data for experimental colonies which were kept in climate rooms and fed supplementary pollen and sugar water. The number of workers produced in the model matched the data from the literature quite well and the colony growth in model showed a similar pattern to experimental colonies with a roughly sigmoid curve (Figure 2b). We calculated the queen investment ratio, accounting for differences in average biomass between queens and males (Appendix S11). The simulations resulted in an average queen investment ratio of 0.46–0.49 (depending on estimated cost ratio), matching the empirical range of 0.44–0.51 (Duchateau & Velthuis, 1988; Duchateau et al. 2004; Table 3). The simulations also captured the overall bimodal distribution in queen investment per colony that was found by Duchateau et al., 2004 (Appendix S11).

#### 3.2.3 Population‐level comparison

##### Setting

We compared predicted nest densities of simulated colonies of *B. terrestris* to estimated field nest densities (Knight et al., 2005). Simulations (*n* = 3) started with 7,500 queens and ran for 10 years in the realistic landscapes (Appendix S03 ODD, p. 66—CreateColoniesProc, Appendix S10).

We recorded the number of colonies in the landscape per km^2^ and calculated the maximum colony density per year and then averaged this over the last 5 years for each simulation run. We compared modelled mean nest densities to nest densities calculated from genetic data, and based on an approximate foraging range of 758 m for *B. terrestris* (Knight et al., 2005).

##### Output

At the population level, the modelled average peak nest of 34 nests/km^2^ is close to the empirical average of 28.7 nests/km^2^ (range 26.6–30.7) for *B. terrestris* in agricultural landscapes (Knight et al., 2005). Figure 2c shows how this changes over the 10‐year simulation.

#### 3.2.4 Model applications

##### Setting 1: single habitat maps

To determine the number of colonies supported by different habitats, an artificial, single‐patch landscape was simulated, starting with 1,000 *B. terrestris* queens. The patch had a size of 1 km^2^ and represented one habitat: grassland, hedgerows, scrub or woodland. Simulations ran for 10 years (*n* = 20).

*Output 1*. The number of all adult bees produced in the last (10th) year, including hibernating queens, and the peak number of colonies in the last year (of the simulations are shown in Table 4. The peak nest densities (per ha) were 0.4 for grassland, 7.0 for hedgerows, 3.6 for scrub and 0.3 for woodland. These habitat specific population measures can be used to estimate bumblebee population sizes on a larger spatial scale, based on the landscape composition. For example, we could predict from the habitat specific colony densities a peak colony number of 974.0 colonies in the realistic landscape, which, however, is higher than the peak of 857.7 colonies from the actual simulations in this landscape. The reason for this discrepancy seems to be that hedgerows are represented by a large number of very small patches in the model, but inefficiently small food sources are automatically removed when the map is processed (see SI03 ODD: p. 44—CreateLayersProc). So linear features such as hedgerows, and the forage they afford to bees, are potentially underrepresented in this model version.

**Table 4:** The number of hibernating queens (*n*. hibernating queens) and the peak number of colonies (*n*. colonies (peak)) (*M* ± *SD*,* N* = 20) predicted in year 10 of the simulation. We used an artificial, single‐patch landscape with 1 km^2^ of the respective habitat and show the number of foraging trips per million (*n*. million foraging trips) and the percentage of nectar foragers (% nectar). Number of bees (*n*. bees) refers to the total number of adult workers, queens and males produced during the last (10th) year

	No. hibernating queens	No. colonies (peak)	No. million foraging trips (% nectar)	No. bees
Grassland	399 (197.9)	40.6 (18.7)	3.2 (76)	134,707.8 (24,019.1)
Hedgerows	7455.6 (530.3)	704.25 (54.9)	31.1 (72)	1,467,027.6 (68,561.1)
Scrub	3752.4 (431.8)	361.6 (43.2)	17.2 (73)	795,631.2 (40,067.7)
Woodland	281.4 (211.6)	30.6 (23.9)	2.7 (77)	107,839.2 (34,476.1)

##### Setting 2: effects of pesticide exposure

Additionally, we simulated reproduction depression as a result of colony‐level pesticide exposure. Baron, Jansen, Brown, and Raine (2017) reported a 26% reduction in colony foundation after queens have been treated with field‐relevant levels of a neonicotinoid pesticide. We simulated the population dynamics based on 7,500 (or 500) initial *B. terrestris* queens and removed 26% of those queens emerging from hibernation every year (n = 20, time steps 3,650, all other settings default, see Appendix S10).

*Output 2*. The annual removal of 26% of emerging queens (from an initial population of 7500) led to a strong reduction in the number of colonies (Figure 2c). However, unlike Baron et al.'s (2017) prediction, the population does not go extinct. Repeating these simulations with 500 initial queens results in an *increase* in nest densities (results not shown).

## 4 DISCUSSION

We have described a new agent‐based systems model, *Bumble*‐BEEHAVE, rich in structural realism and mechanism that can be used to examine the effects of multiple stressors on bumblebee colonies and populations over multiple years, in realistic landscapes. It includes the option of modelling up to six different bumblebee species. We illustrate that individual, colony and population level processes of bumblebee colonies can be predicted by *Bumble‐*BEEHAVE within the boundaries of independent empirical data. Indeed the values for different life stages and key time points (Table 3) show strong agreement with published data. Despite some values for parameters still being approximate in the literature, the design and structure of *Bumble‐*BEEHAVE means that a user can add or alter those values as they become available to run the model in future.

In addition, *Bumble‐*BEEHAVE is the only model to our knowledge to incorporate energy budgets and depletion of resources in mapped landscapes so that interspecific and intraspecific competition can emerge. Also, because the flower patches are spatially explicit, then the patchy exposure to pesticides in the landscape can be simulated in future, by programming different pesticide applications to different crops, and implementing differential mortality and sublethal effects depending on when and where the bees are foraging—a pesticide module of this type is currently being implemented for BEEHAVE and *Bumble*‐BEEHAVE.

Our simulations demonstrate that colony dynamics and population are driven by the spatio‐temporal availability of resources as expected (Crone & Williams, 2016; Goulson, Hughes, Derwent, & Stout, 2002; Williams, Regetz, & Kremen, 2012).

While the comparative simulations are promising, without duplicating the realistic spatially explicit forage landscape of the independent empirical studies, we were unable to replicate all absolute values and trends in the empirical data. Additionally, when we do have a landscape map that replicates an empirical landscape, this is a simplification of the full range of resources that pollinators utilise in the wild. It is vital for pollinator models to operate in realistic landscapes (EFSA, 2015) at a scale relevant to bumblebee ecology and to policy and land management. *Bumble‐*BEEHAVE input maps represent a 5 km × 5 km landscape covering the likely foraging range of bumblebees of up to 2 km (Osborne, Martin, Carreck, et al., 2008), and so provide a flexible tool for scientists and practitioners to explore the effects of multifactorial stressors and their potential mitigation at relevant scales. The outputs can include documentation of which foragers, and how many, have foraged on different resource patches in the landscapes and the results can be compared to other landscape‐scale pollination service models (Lonsdorf et al., 2009; Olsson et al., 2015; Polce et al., 2013), but the novelty of *Bumble*‐BEEHAVE is that because it explicitly programmes the life cycle of the colony via individual behaviours, it includes resource depletion, and has the potential to include predation, pathogen and pesticide exposure effects. Importantly, we simulated the impact of reproductive depression caused by a pesticide, as measured by Baron et al. (2017). They used a simple model to predict that the impact could be colony extinction. Our simulations gave a more nuanced result, suggesting that the impact will depend on the initial population size: so while colony numbers might reduce, the population is likely to stabilise, though at a lower density than for control.

### 4.1 Applications

*Bumble‐*BEEHAVE is open‐source (Appendix S01 and via www.beehave-model.net), thoroughly documented and has flexible settings, enabling even nonspecialist users to simulate the effects of stressors by adjusting and/or updating parameters as data become available. The predecessor honeybee model, BEEHAVE (Becher et al., 2014) is being used by regulators, industry and land managers for risk assessment and decision support relating to honeybees. Thus, we foresee the integration of *Bumble‐*BEEHAVE beyond academia to industry, conservation and policy.

*Bumble‐*BEEHAVE can be used to:

1. Explore how stressors combine, resulting in emergent properties of colony and population success in realistic landscapes.
2. Identify tipping points as a result of multiple stressors that lead to colony failures as well as the feedback mechanisms that can buffer the effects of stressors.
3. Predict pollination services for current and/or future cropping patterns in realistic landscape settings.
4. Test the relative effects of specific policy recommendations for pollinators in agricultural landscapes, such as planting pollen and nectar strips (Dicks et al., 2015).
5. Explore multiple landscapes comprising various habitat types with unique forage species composition in combination with the UK nectar database (Baude et al., 2016).

## 5 CONCLUSIONS

*Bumble*‐BEEHAVE represents a significant step towards predicting individual to population level effects of multiple stressors operating at multiple scales in a spatially explicit way and is designed to leave scope for future model comparison and development. With sensitivity analysis and verification, we have demonstrated that *Bumble*‐BEEHAVE makes realistic predictions, and thus has the potential to be a powerful decision support tool to be used by scientists and stakeholders to explore a range of questions in bumblebee ecology and conservation—used to aid the design of field experiments, for risk assessments and for assigning bespoke management recommendations at a landscape scale.

## ACKNOWLEDGEMENTS

This work was undertaken with BBSRC funding BB/J014753/1 and BB/J014915/1. Data collection and fieldwork assistance for the creation of the input maps and the habitats and flower species input files was from Jennifer Swain and Trish Wells at Rothamsted Research Centre and Beth Nicholls, Rob Fowler, Cristina Botias Talamantes and Elinor Jax from the University of Sussex. Advice from Alison Haughton from Rothamsted Research Centre.

## AUTHORS' CONTRIBUTIONS

M.A.B. was the main developer of the model, wrote the model documentation and co‐wrote the manuscript. G.T.D. created spatially explicit forage landscape maps, contributed to model development and data collation, ran model comparison and application simulations, wrote R scripts and wrote and edited the manuscript. M.A.B. and G.T.D. contributed equally to this work. T.D.P. contributed to data collation, sub‐models, sensitivity analyses, ODD and R scripts. D.G. led the BBSRC application that funded the work, was involved in discussions over model development and parameterisation and commented on the manuscript. E.L.R. collected empirical data on flower density, nectar and pollen quality and quantity, and land‐use. J.L.O. co‐designed the project, contributed to model development and design of simulations, and co‐wrote the manuscript. All authors gave final approval for publication.

## DATA ACCESSIBILITY

Data available via the Dryad Digital Repository https://doi.org/10.5061/dryad.ft3tq32 (Becher et al., 2018).

## References

[jpe13165-bib-0001] Banks, H. T. , Banks, J. E. , Bommarco, R. , Laubmeier, A. N. , Myers, N. J. , Rundlof, M. , … Tillman, K. (2017). Modeling bumble bee population dynamics with delay differential equations. Ecological Modelling, 351, 14–23. 10.1016/j.ecolmodel.2017.02.011

[jpe13165-bib-0002] Baron, G. L. , Jansen, V. A. A. , Brown, M. J. F. , & Raine, N. E. (2017). Pesticide reduces bumblebee colony initiation and increases probability of population extinction. Nature Ecology & Evolution, 1, 1308–1316. 10.1038/s41559-017-0260-1 29046553 PMC6485633

[jpe13165-bib-0003] Baude, M. , Kunin, W. E. , Boatman, N. D. , Conyers, S. , Davies, N. , Gillespie, M. A. K. , … Memmott, J. (2016). Historical nectar assessment reveals the fall and rise of floral resources in Britain. Nature, 530, 85–88. 10.1038/nature16532 26842058 PMC4756436

[jpe13165-bib-0004] Becher, M. A. , Grimm, V. , Knapp, J. , Horn, J. , Twiston‐Davies, G. , & Osborne, J. L. (2016). BEESCOUT: A model of bee scouting behaviour and a software tool for characterizing nectar/pollen landscapes for BEEHAVE. Ecological Modelling, 340, 126–133. 10.1016/j.ecolmodel.2016.09.013 27890965 PMC5070411

[jpe13165-bib-0005] Becher, M. A. , Grimm, V. , Thorbek, P. , Horn, J. , Kennedy, P. J. , & Osborne, J. L. (2014). BEEHAVE: A systems model of honeybee colony dynamics and foraging to explore multifactorial causes of colony failure. Journal of Applied Ecology, 51, 470–482. 10.1111/1365-2664.12222 25598549 PMC4283046

[jpe13165-bib-0006] Becher, M. A. , Osborne, J. L. , Thorbek, P. , Kennedy, P. J. , & Grimm, V. (2013). REVIEW: Towards a systems approach for understanding honeybee decline: A stocktaking and synthesis of existing models. Journal of Applied Ecology, 50, 868–880. 10.1111/1365-2664.12112 24223431 PMC3810709

[jpe13165-bib-0007] Becher, M. A. , Twiston‐Davies, G. , Penny, D. T. , Goulson, D. , Rotheray, E. L. , & Osborne, J. L. (2018). Data from: *Bumble*‐BEEHAVE: A systems model for exploring multifactorial causes of bumblebee decline at individual, colony, population and community level. Dryad Digital Repository, 10.5061/dryad.ft3tq32 PMC6221040 30449898

[jpe13165-bib-0008] Bryden, J. , Gill, R. J. , Mitton, R. A. A. , Raine, N. E. , & Jansen, V. A. A. (2013). Chronic sublethal stress causes bee colony failure. Ecology Letters, 16, 1463–1469. 10.1111/ele.12188 24112478 PMC4299506

[jpe13165-bib-0009] Cresswell, J. E. (2017). A demographic approach to evaluating the impact of stressors on bumble bee colonies. Ecological Entomology, 42, 221–229. 10.1111/een.12376

[jpe13165-bib-0010] Crone, E. E. , & Williams, N. M. (2016). Bumble bee colony dynamics: Quantifying the importance of land use and floral resources for colony growth and queen production. Ecology Letters, 19, 460–468. 10.1111/ele.12581 26913696

[jpe13165-bib-0011] Dicks, L. V. , Baude, M. , Roberts, S. P. M. , Phillips, J. , Green, M. , & Carvell, C. (2015). How much flower‐rich habitat is enough for wild pollinators? Answering a key policy question with incomplete knowledge. Ecological Entomology, 40, 22–35. 10.1111/een.12226 26877581 PMC4737402

[jpe13165-bib-0012] Duchateau, M. J. , & Velthuis, H. H. W. (1988). Development and reproductive strategies in Bombus colonies. Behaviour, 107, 186–207. 10.1163/156853988X00340

[jpe13165-bib-0013] Duchateau, M. J. , Velthuis, H. H. W. , & Boomsma, J. J. (2004). Sex ratio variation in the bumblebee *Bombus terrestris* . Behavioral Ecology, 15, 71–82. 10.1093/beheco/arg087

[jpe13165-bib-0014] EFSA . (2015). Statement on the suitability of the BEEHAVE model for its potential use in a regulatory context and for the risk assessment of multiple stressors in honeybees at the landscape level. PPR Panel (EFSA Panel on Plant Protection Products and their Residues), EFSA. EFSA Journal, 13(6):4125

[jpe13165-bib-0015] Fowler, R. E. , Rotheray, E. L. , & Goulson, D. (2016). Floral abundance and resource quality influence pollinator choice. Insect Conservation and Diversity, 9, 481–494. 10.1111/icad.12197

[jpe13165-bib-0016] Gosterit, A. , & Gurel, F. (2016). Male remating and its influences on queen colony foundation success in the bumblebee, *Bombus terrestris* . Apidologie, 47, 828–834. 10.1007/s13592-016-0438-6

[jpe13165-bib-0017] Goulson, D. (2015). Neonicotinoids impact bumblebee colony fitness in the field: A reanalysis of the UK's Food & Environment Research Agency 2012 experiment. Peerj, 3, e854 10.7717/peerj.854 25825679 PMC4375969

[jpe13165-bib-0100] Goulson, D. , Hughes, W. , Derwent, L. , & Stout, J. (2002). Colony growth of the bumblebee, Bombus terrestris, in improved and conventional agricultural and suburban habitats. Oecologia, 130, 267–273. 28547150 10.1007/s004420100803

[jpe13165-bib-0018] Goulson, D. , Lepais, O. , O'Connor, S. , Osborne, J. L. , Sanderson, R. A. , Cussans, J. , … Darvill, B. (2010). Effects of land use at a landscape scale on bumblebee nest density and survival. Journal of Applied Ecology, 47, 1207–1215. 10.1111/j.1365-2664.2010.01872.x

[jpe13165-bib-0019] Goulson, D. , Nicholls, E. , Botias, C. , & Rotheray, E. L. (2015). Bee declines driven by combined stress from parasites, pesticides, and lack of flowers. Science, 347, 1435–1444. 10.1126/science.1255957 25721506

[jpe13165-bib-0020] Grimm, V. , Berger, U. , Bastiansen, F. , Eliassen, S. , Ginot, V. , Giske, J. , … DeAngelis, D. L. (2006). A standard protocol for describing individual‐based and agent‐based models. Ecological Modelling, 198, 115–126. 10.1016/j.ecolmodel.2006.04.023

[jpe13165-bib-0021] Grimm, V. , Berger, U. , DeAngelis, D. L. , Polhill, J. G. , Giske, J. , & Railsback, S. F. (2010). The ODD protocol: A review and first update. Ecological Modelling, 221, 2760–2768. 10.1016/j.ecolmodel.2010.08.019

[jpe13165-bib-0022] Grimm, V. , & Railsback, S. F. (2005). Individual‐based modeling and ecology. Princeton and Oxford: Princeton University Press, 428 pp. 10.1515/9781400850624

[jpe13165-bib-0023] Harder, L. D. (1983). Flower handling efficiency of bumblebees – Morphological aspects of probing time. Oecologia, 57, 274–280. 10.1007/BF00379591 28310186

[jpe13165-bib-0024] Häussler, J. , Sahlin, U. , Baey, C. , Smith, H. G. , & Clough, Y. (2017). Pollinator population size and pollination ecosystem service responses to enhancing floral and nesting resources. Ecology and Evolution, 7, 1898–1908. 10.1002/ece3.2765 28331597 PMC5355185

[jpe13165-bib-0025] Henry, M. , Becher, M. A. , Osborne, J. L. , Kennedy, P. J. , Aupinel, P. , Bretagnolle, V. , … Requier, F. (2017). Predictive systems models can help elucidate bee declines driven by multiple combined stressors. Apidologie, 48, 328–339. 10.1007/s13592-016-0476-0

[jpe13165-bib-0026] IPBES (2016). In Potts S. G., Imperatriz‐Fonseca V. L., Ngo H. T., Biesmeijer J. C., Breeze T. D., Dicks L. V., Garibaldi L. A., Hill R., Settele J., Vanbergen A. J., Aizen M. A., Cunningham S. A., Eardley C., Freitas B. M., Gallai N., Kevan P. G., Kovacs‐Hostyanszki A., Kwapong P. K., Li J., Li X., Martins D. J., Nates‐Parra G., Pettis J. S., Rader R., & Viana B. F. (Eds.), Summary for policymakers of the assessment report of the Intergovernmental Science‐Policy Platform on Biodiversity and Ecosystem Services on pollinators, pollination and food production. Bonn, Germany: Secretariat of the Intergovernmental Science‐Policy Platform on Biodiversity and Ecosystem Services.

[jpe13165-bib-0027] Kerr, J. T. , Pindar, A. , Galpern, P. , Packer, L. , Potts, S. G. , Roberts, S. M. , … Pantoja, A. (2015). Climate change impacts on bumblebees converge across continents. Science, 349, 177–180. 10.1126/science.aaa7031 26160945

[jpe13165-bib-0028] Knight, M. E. , Martin, A. P. , Bishop, S. , Osborne, J. L. , Hale, R. J. , Sanderson, A. , & Goulson, D. (2005). An interspecific comparison of foraging range and nest density of four bumblebee (*Bombus*) species. Molecular Ecology, 14, 1811–1820. 10.1111/j.1365-294X.2005.02540.x 15836652

[jpe13165-bib-0029] Lonsdorf, E. , Kremen, C. , Ricketts, T. , Winfree, R. , Williams, N. , & Greenleaf, S. (2009). Modelling pollination services across agricultural landscapes. Annals of Botany – London, 103, 1589–1600. 10.1093/aob/mcp069 PMC2701767 19324897

[jpe13165-bib-0030] Lopez‐Vaamonde, C. , Raine, N. E. , Koning, J. W. , Brown, R. M. , Pereboom, J. M. M. , Ings, T.C. , … Bourke, A. F. G. (2009). Lifetime reproductive success and longevity of queens in an annual social insect. Journal of Evolutionary Biology, 22, 983–996 10.1111/j.1420-9101.2009.01706.x 19298495

[jpe13165-bib-0031] Olsson, O. , Bolin, A. , Smith, H. G. , & Lonsdorf, E. V. (2015). Modeling pollinating bee visitation rates in heterogeneous landscapes from foraging theory. Ecological Modelling, 316, 133–143. 10.1016/j.ecolmodel.2015.08.009

[jpe13165-bib-0032] Osborne, J. L. , Martin, A. P. , Carreck, N. L. , Swain, J. L. , Knight, M. E. , Goulson, D. , … Sanderson, R. A. (2008). Bumblebee flight distances in relation to the forage landscape. Journal of Animal Ecology, 77(2), 406–415. 10.1111/j.1365-2656.2007.01333.x 17986207

[jpe13165-bib-0033] Osborne, J. L. , Martin, A. P. , Shortall, C. R. , Todd, A. D. , Goulson, D. , Knight, M. E. , … Sanderson, R. A. (2008). Quantifying and comparing bumblebee nest densities in gardens and countryside habitats. Journal of Applied Ecology, 45, 784–792.

[jpe13165-bib-0034] Polce, C. , Termansen, M. , Aguirre‐Gutierrez, J. , Boatman, N. D. , Budge, G. E. , Crowe, A. , … Biesmeijer, C. (2013). Species distribution models for crop pollination: A modelling framework applied to Great Britain. PLoS ONE, 8(10), e76308 10.1371/journal.pone.0076308 24155899 PMC3796555

[jpe13165-bib-0035] R Core Team . (2015). R: A language and environment for statistical computing. Vienna, Austria: R Foundation for Statistical Computing https://www.R-project.org/

[jpe13165-bib-0036] Stelzer, R. J. , Stanewsky, R. , & Chittka, L. (2010). Circadian foraging rhythms of bumblebees monitored by radio‐frequency identification. Journal of Biological Rhythms, 25, 257–267. 10.1177/0748730410371750 20679495

[jpe13165-bib-0037] Thiele, J. C. (2014). R Marries NetLogo: Introduction to the RNetLogo package. Journal of Statistical Software, 58(2), 1–41.

[jpe13165-bib-0038] Vanbergen, A. J. , Baude, M. , Biesmeijer, J. C. , Britton, N. F. , Brown, M. J. F. , Brown, M. , … Wright, G. A. (2013). Threats to an ecosystem service: Pressures on pollinators. Frontiers in Ecology and the Environment, 11, 251–259. 10.1890/120126

[jpe13165-bib-0039] Wilensky, U. (1999). Netlogo. Evanston, IL: Center for Connected Learning and Computer‐based Modeling Northwestern University.

[jpe13165-bib-0040] Williams, P. H. , & Osborne, J. L. (2009). Bumblebee vulnerability and conservation world‐wide. Apidologie, 40, 367–387. 10.1051/apido/2009025

[jpe13165-bib-0041] Williams, N. M. , Regetz, J. , & Kremen, C. (2012). Landscape‐scale resources promote colony growth but not reproductive performance of bumble bees. Ecology, 93, 1049–1058. 10.1890/11-1006.1 22764491

