# Supplementary material for: Bumble‐BEEHAVE: A systems model for exploring multifactorial causes of bumblebee decline at individual, colony, population and community level
Source: J Appl Ecol. 2018 May 22;55(6):2790–801. doi: 10.1111/1365-2664.13165 (PMC6221040; doi:10.1111/1365-2664.13165)
Supplement: Supplementary file 2 [file JPE-55-2790-s002.pdf]

SI_01_*Bumble*-BEEHAVE model screenshot

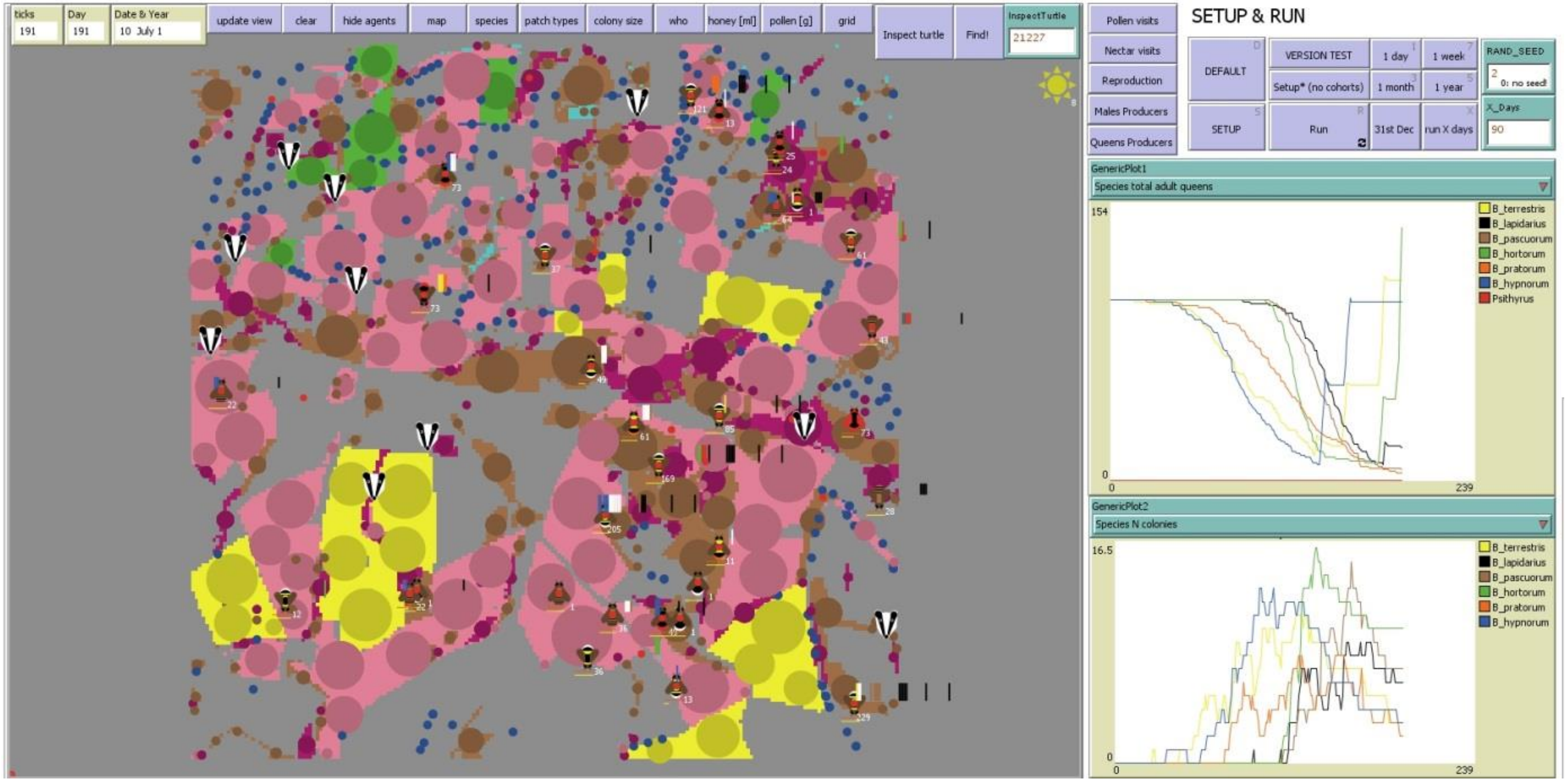

Figure SI 01. Screenshot of *Bumble*-BEEHAVE Netlogo interface with buttons (lilac) and variable inputs (green). Landscape depicted on left with coloured patches denoting different forage types, bee symbols denote location of nests, badger faces denote badger sett location. A variety of outputs can be plotted over time (on the right): in this case the plots show the number of queens, and the number of colonies for each of 6 species (shown in different colours).
