# Supplementary material for: Bumble‐BEEHAVE: A systems model for exploring multifactorial causes of bumblebee decline at individual, colony, population and community level
Source: J Appl Ecol. 2018 May 22;55(6):2790–801. doi: 10.1111/1365-2664.13165 (PMC6221040; doi:10.1111/1365-2664.13165)
Supplement: Supplementary file 4 [file JPE-55-2790-s004.pdf]

BUMBLE-BEEHAVE: Manual

To run the Bumble-BEEHAVE model you need to install the free, open source software NetLogo version 5.3.1: https://ccl.northwestern.edu/netlogo/5.3.1/

Bumble-BEEHAVE can be downloaded here: http://beehave-model.net/

Make sure the Bumble-BEEHAVE folder is unzipped and all input files are in the same folder as the program. We recommend to also carefully read the ODD protocol for the model.

Overview Interface

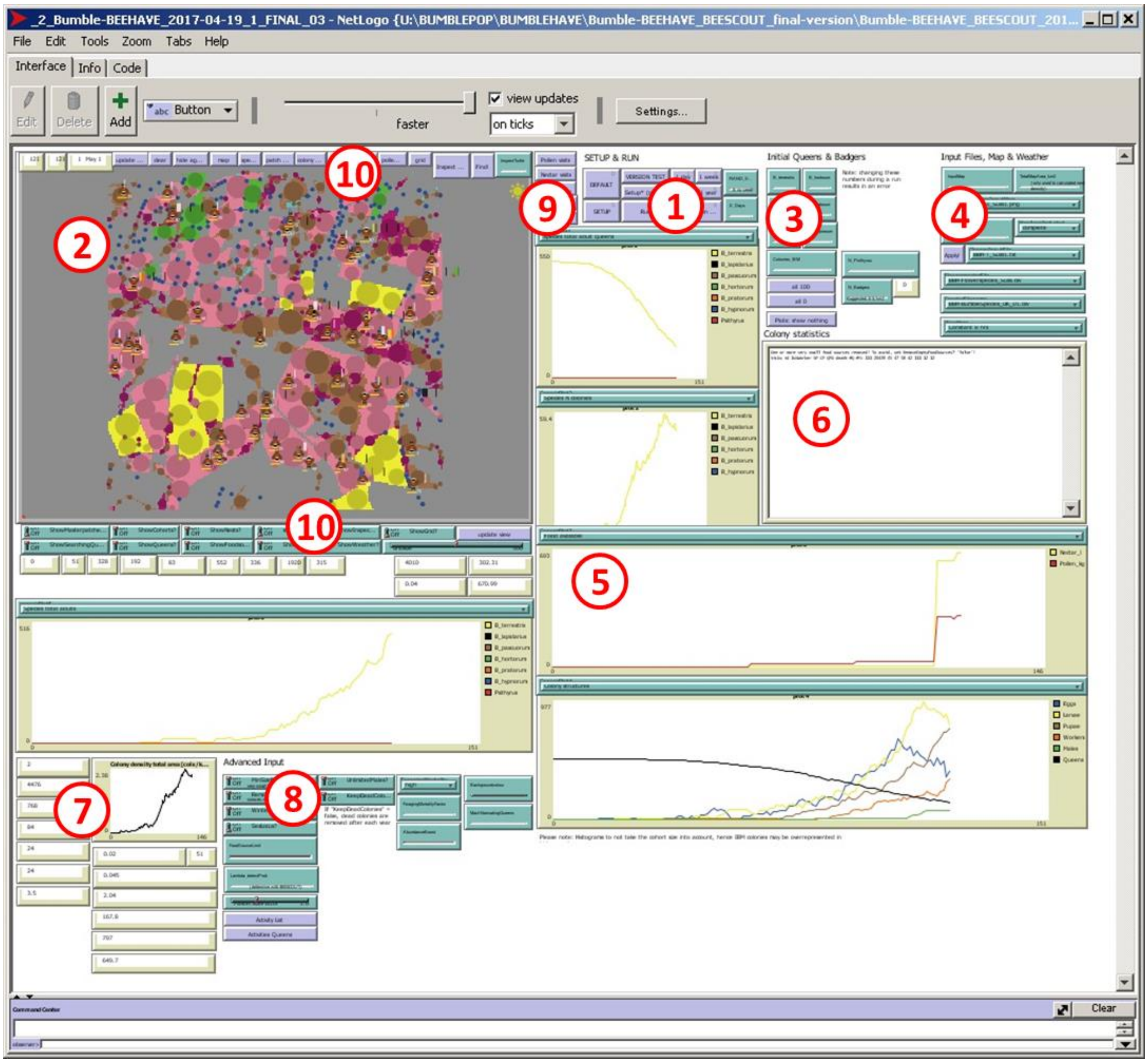

1

1.) To run the model, press "SETUP" and "Run" (Or "1 day", "1 week" etc.). "31stDec" runs the model until the end of the year. "run X Days" runs it for "X_Days" (Note: green boxes are input fields). "DEFAULT" sets all input options on the interface (except of "RAND_SEED") to their default value. "VERSION TEST" runs the model under specified conditions to determine whether or not the code was changed (However, some changes might remain undetected). "RAND_SEED" defines the initial value for the pseudo-random number generator and hence the sequence of random numbers created during a model run, i.e. without other changes, the same random-seed creates exactly the same results. If "RAND_SEED" is 0, the seed is automatically set, based on the current date and time. In this case, the results of a run are not replicable and every run (slightly) differs from previous runs.

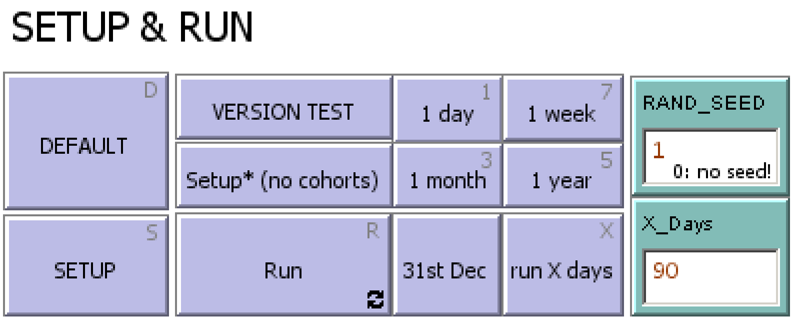

Use the NetLogo speed slider to increase the running speed of the model:

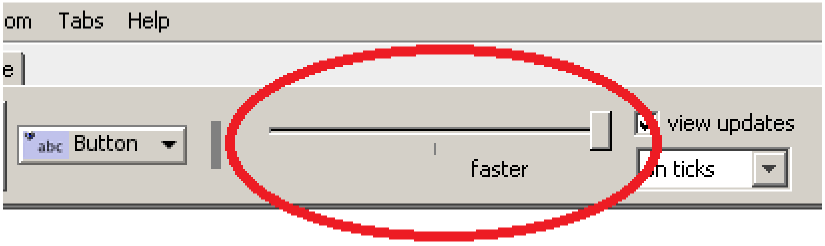

2

2) The simulated "world" shows locations of food sources and bumblebee colonies as well as colony structures and stores.

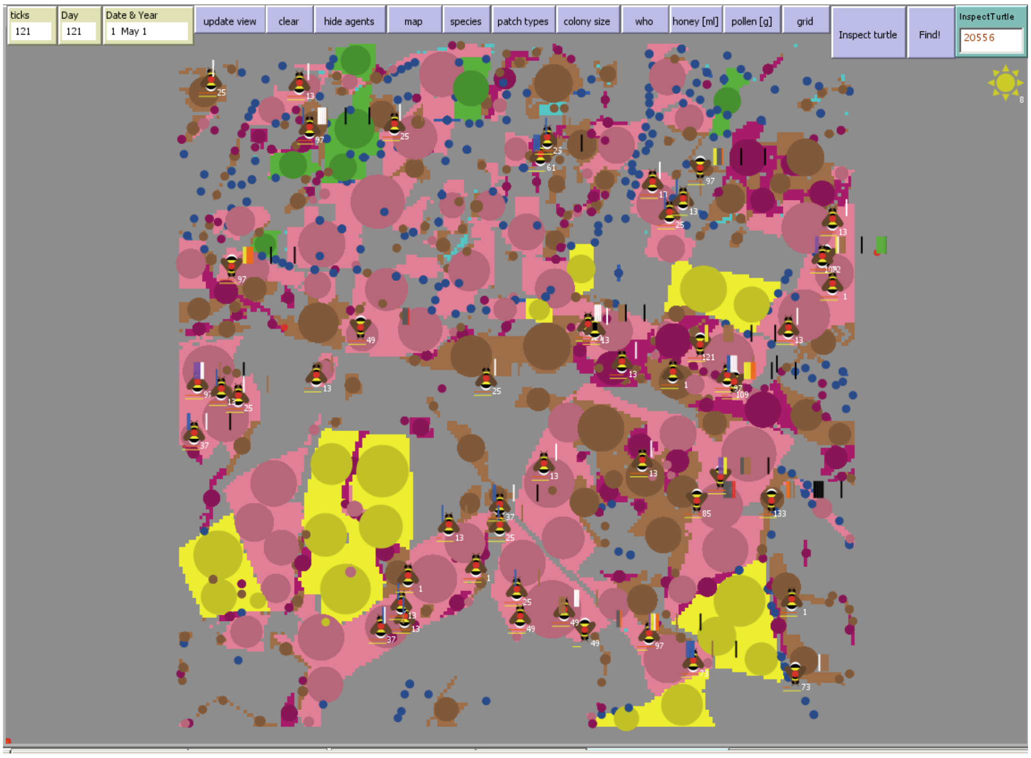

Food sources are shown as circles, colonies as bumble bees:

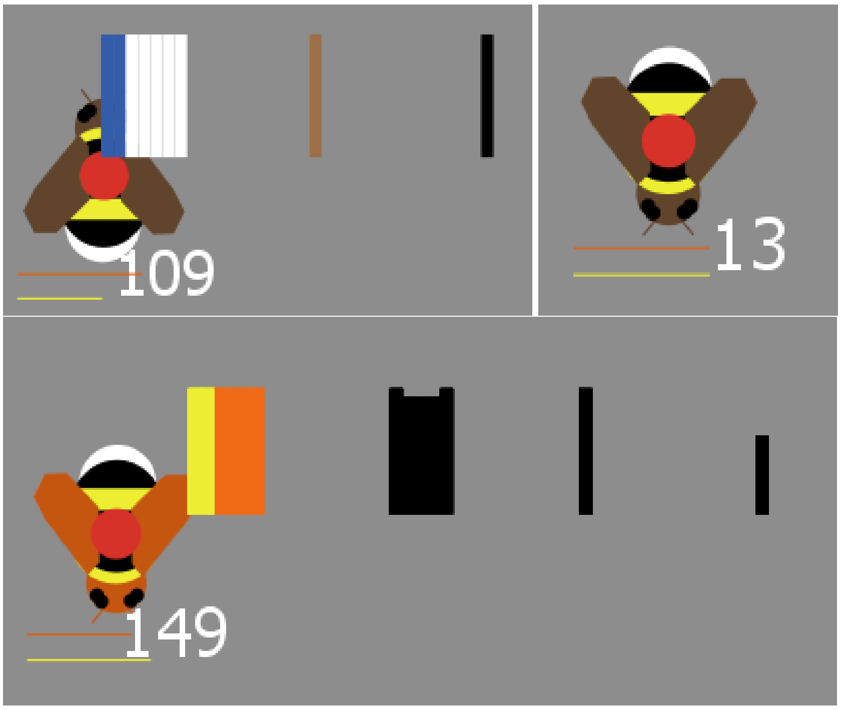

Adult queens are shown as red circles (mother queens on the "thorax" of their colony). Each (vertical) bar represents a cohort of bees, with the size of the bar reflecting the number of bees (in "cohort-based colonies" (see 3) this number is constant (= 12 bees)). Colonies are shown upside down once they reached their competition point and no more eggs can be produced. "Individual-based colonies" are shown with orange wings and head.

The horizontal bars below the colony show the pollen (orange) and nectar (yellow) stores of the colony, relative to the colony's need for nectar and pollen. The number in the bottom right corner (the colony's label) shows the total colony size (including brood) (to show other colony statistics, see 10).

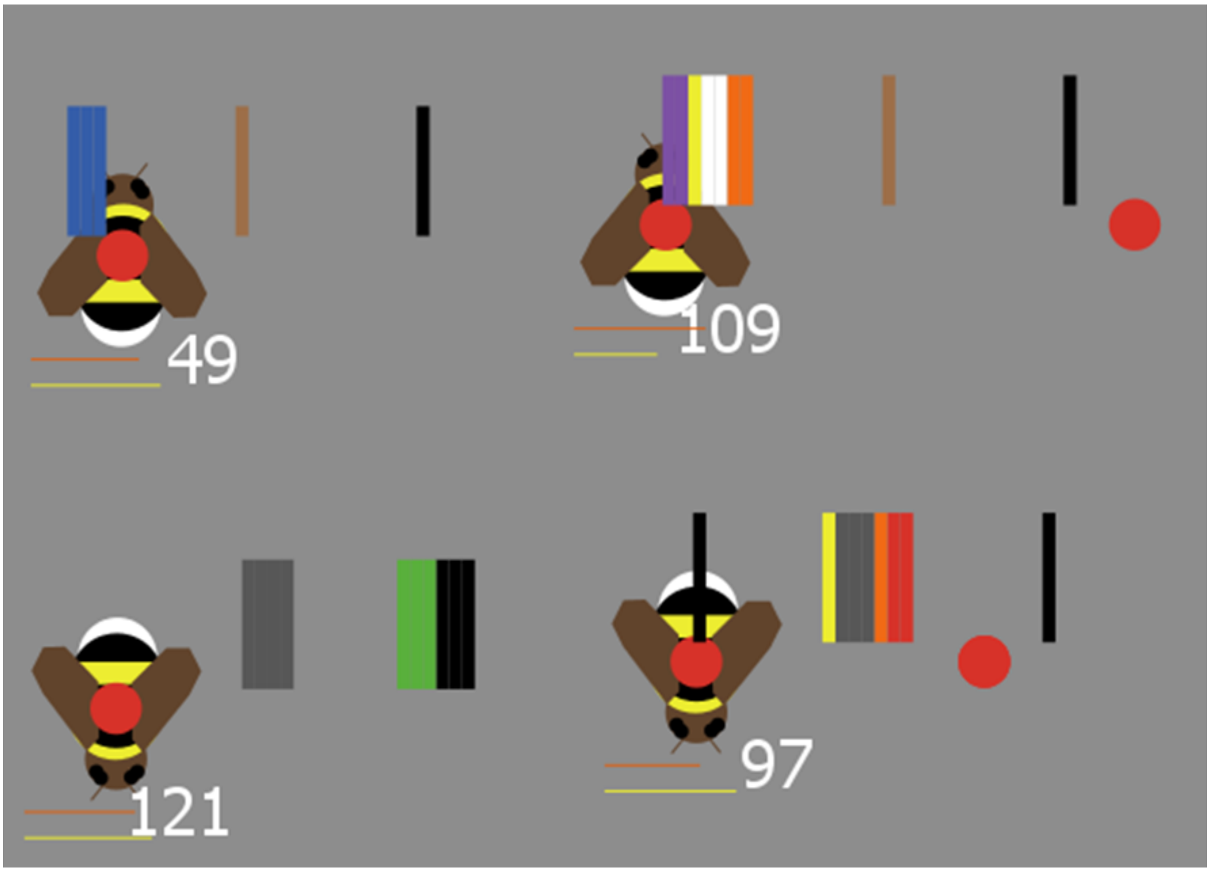

Blue bars are diploid (female) eggs, white bars (female) larvae, brown bars are worker pupae, black bars are adult workers. Purple bars are haploid (male) eggs, yellow bars male larvae, grey bars male pupae and green bars male adults. Orange bars are queen destined larvae, red bars queen pupae (red circles away from a colony are hibernating queens). Diploid male brood follows the same colour schemes as diploid worker brood but diploid adult males are shown in green (if "SexLocus?" is true).

3

3.) Modify the initial number of queens (you have to press "Setup" afterwards to run the model). "N_Psithyrus" refers to (generic) cuckoo bees which can infest the colonies of any bumblebee species. "N_Badgers" defines the number of badger setts. Colonies in the vicinity of a badger sett might be destroyed. If not enough habitat for badgers are available, the actual number of setts created (shown on the yellow monitor "N badgers") might be lower than the input value of "N_Badgers". "Colonies_IBM" defines the number of colonies created as fully individual based (as

opposed to "cohort-based colonies" where each cohort of bees is represented by only one bee agent in the model).

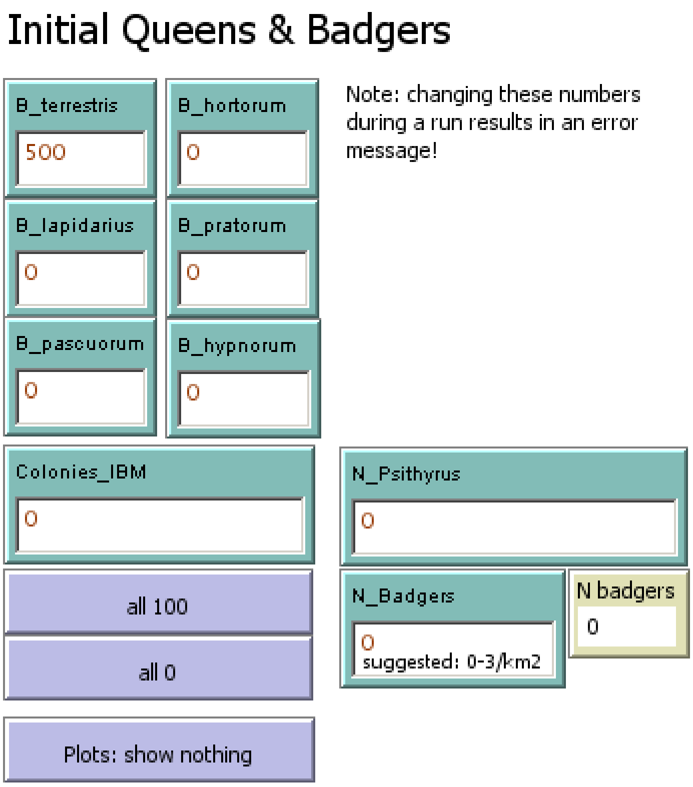

Representations of colonies of the different bumblebee species (top left to bottom right: B. hypnorum, B. pratorum, B. terrestris, B. hortorum, B. pascuorum and B. lapidarius):

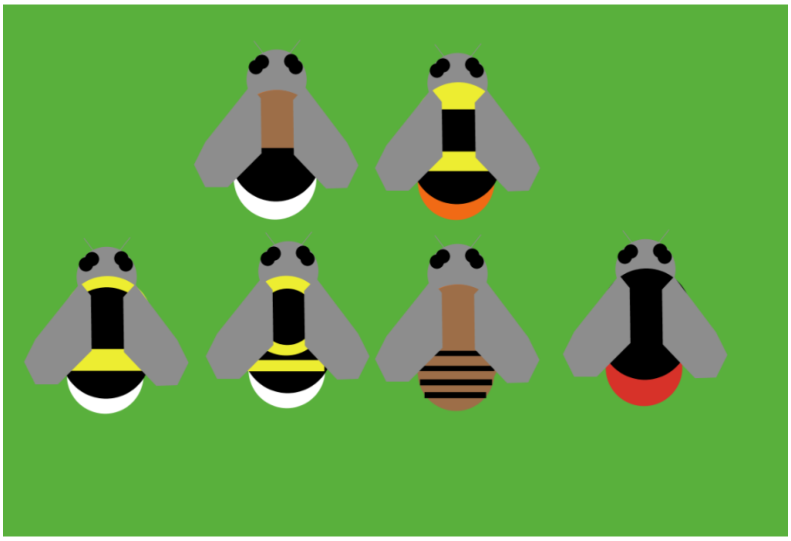

Colonies infested by a Psithyrus queen are surrounded by a circle:

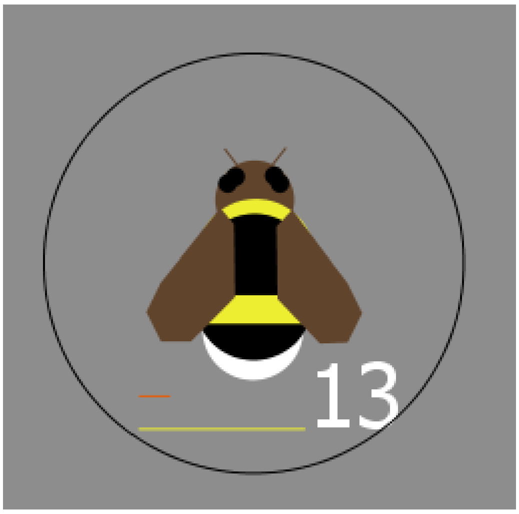

Representation of a badger sett:

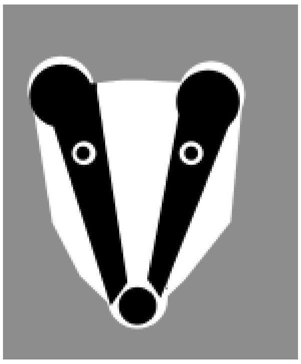

4

4.) "InputMap" is an image file showing the map of the model area. It serves only to inform the user but is not used by the model itself. "ChooseInputMap" and pressing "Apply" is a quick way to set "InputMap", given that the file name is already defined (right mouse click on "ChooseInputMap" and "Edit" allows the user to add/delete options). "TotalMapArea_km2" is the area [km^2] of the loaded map and is used to calculate the nest density per km^2 ("ColonyDensity_km2").
"Input_File" specifies the the text file that defines the food sources in the landscape (their location, the amount of nectar and pollen they provide etc.). "MapAreaIncluded" is a quick way to restrict the simulation to only a half or a quarter of the total area, which speeds up model runs."FlowerspeciesFile" defines the characterstics of forage plant species, "SpeciesFilename" defines the input files that contains parameterisation for the bumblebee species. "Weather" defines the maximal time the bees are allowed to forage on each day.

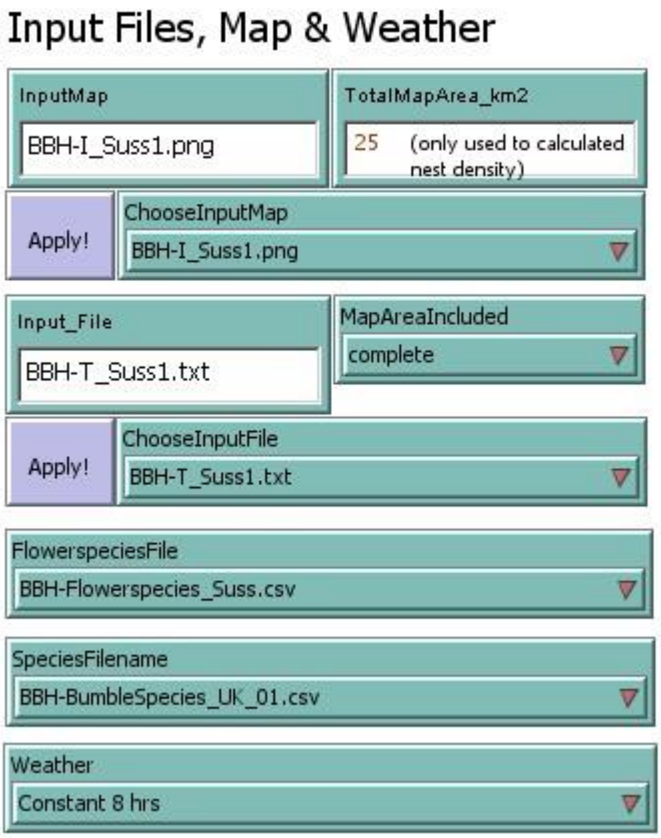

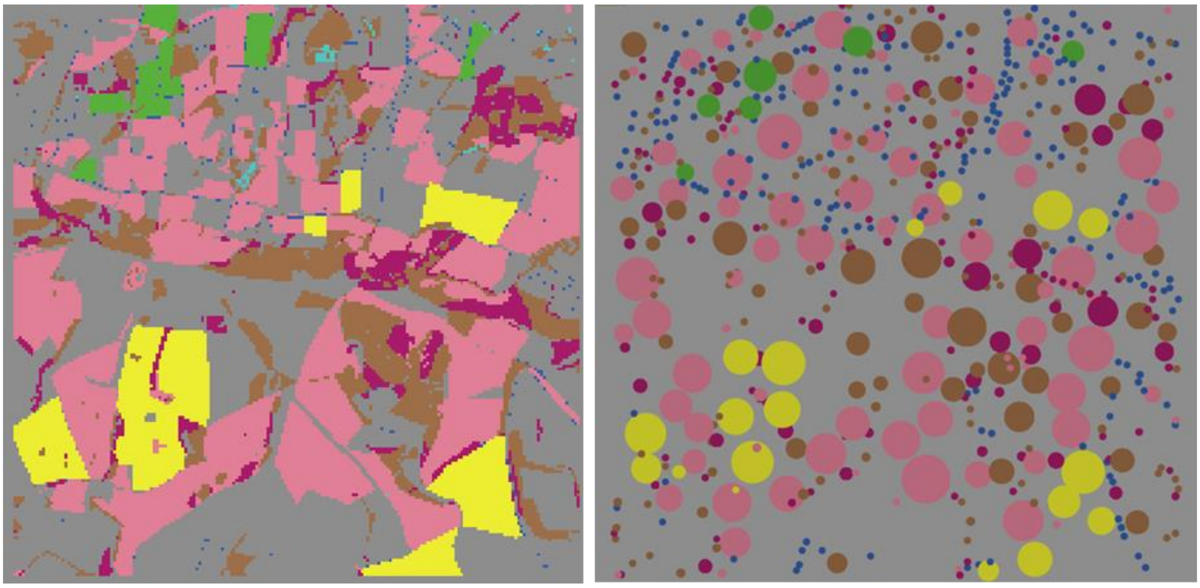

Left: Landscape as defined by "InputMap" only. Right: Same landscape represented by food sources, defined in "Input_File"). The map on the left only serves to inform the user, the model itself is solely based the location and size of defined food sources (right map).

The weather symbols illustrate todays foraging conditions, with the maximal hours of foraging shown at the bottom right of the symbol:

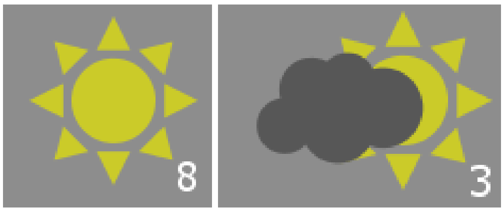

⑤

5.) A number of plots visualise the results of the simulation run. Click on the red triangle to change the output shown on each of the "generic" plots.

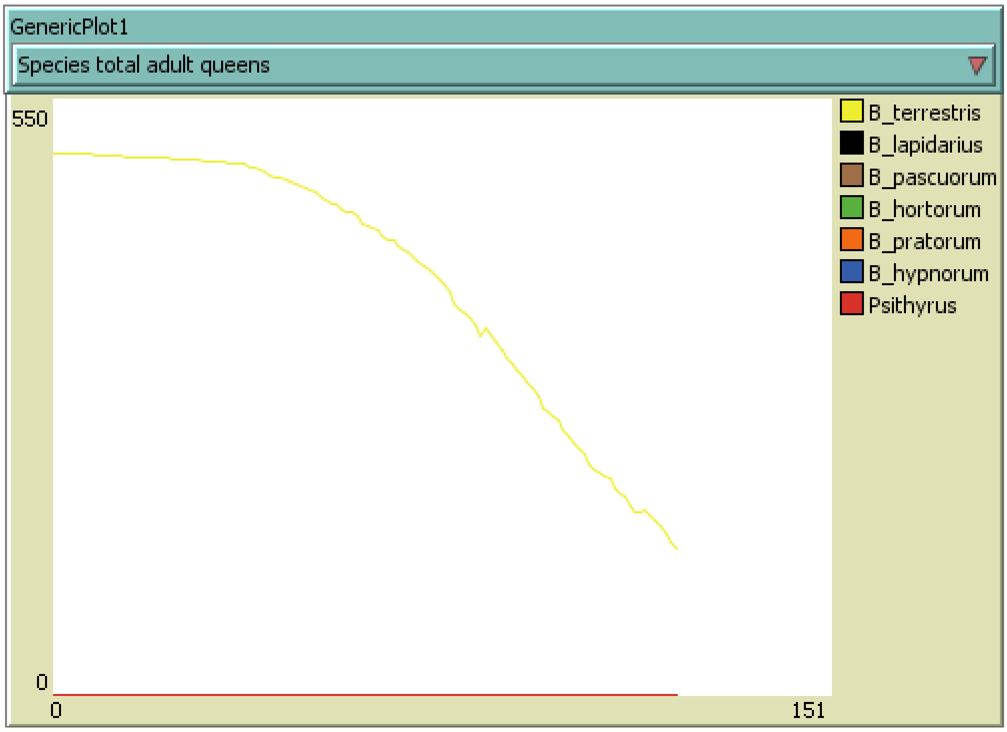

⑥

6.) Some results are also shown on the "Output" window, e.g. characteristic dates of colony development (time step of emergence of first worker, switch point (SP), competition point (CP), queen production, death of colony)and the number of queens and males produced whenever a colony dies.

Colony statistics

```
One or more very small food sources removed! To avoid, set RemoveEmptyFoodSources? 'false'!
ticks id 1stWorker SP CP QPD death #Q #M: 111 20439 45 47 58 42 111 12 12
ticks id 1stWorker SP CP QPD death #Q #M: 134 20514 89 89 101 84 134 12 0
ticks id 1stWorker SP CP QPD death #Q #M: 138 20577 98 103 118 104 138 0 0
ticks id 1stWorker SP CP QPD death #Q #M: 138 20810 123 124 136 119 138 0 0
ticks id 1stWorker SP CP QPD death #Q #M: 141 20914 129 129 141 124 141 0 0
ticks id 1stWorker SP CP QPD death #Q #M: 142 20875 127 129 140 124 142 0 0
ticks id 1stWorker SP CP QPD death #Q #M: 142 20507 87 111 116 106 142 24 0
ticks id 1stWorker SP CP QPD death #Q #M: 144 20558 95 96 108 91 144 24 0
ticks id 1stWorker SP CP QPD death #Q #M: 145 20491 81 81 93 76 145 12 72
ticks id 1stWorker SP CP QPD death #Q #M: 145 20938 131 131 143 126 145 0 0
ticks id 1stWorker SP CP QPD death #Q #M: 148 20556 95 115 122 112 148 12 0
ticks id 1stWorker SP CP QPD death #Q #M: 155 20730 114 115 127 110 155 24 0
ticks id 1stWorker SP CP QPD death #Q #M: 157 20644 105 108 119 103 157 0 0
```

(7)

7.) More information is provided by monitors and (non generic) plots.

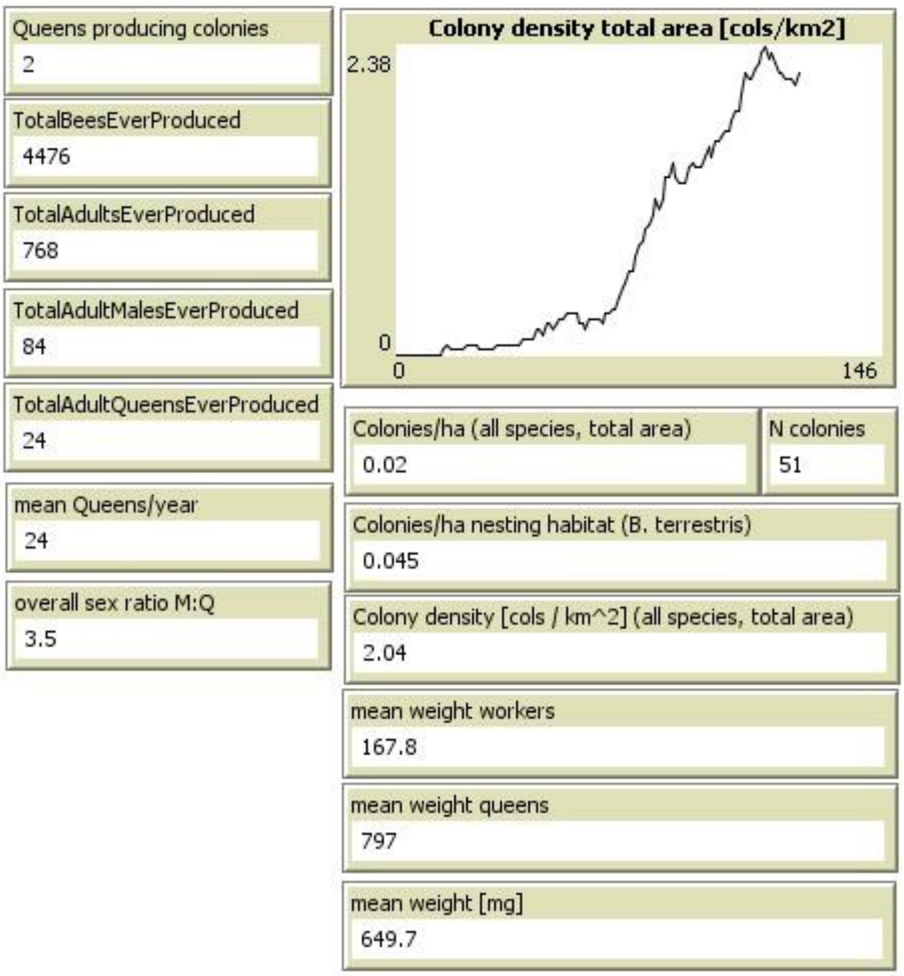

Right mouse click & "Edit" to show under "Reporter" how the value is calculated. For the calculation, of a global variable, go to the code and search for "set VARIABLENAME"

For example: calculation of "Colony density [cols / km^2] (all species, total area)":

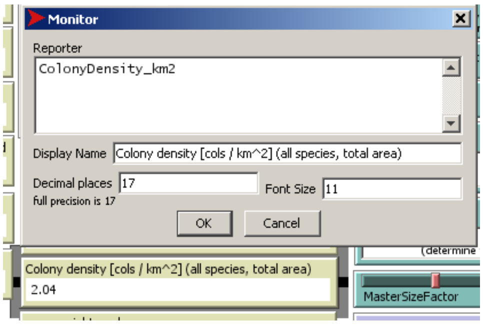

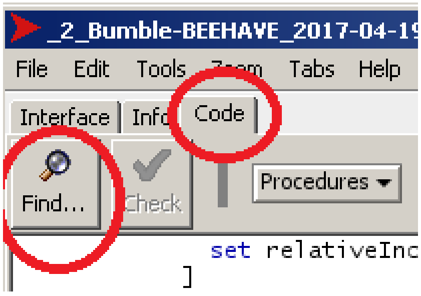

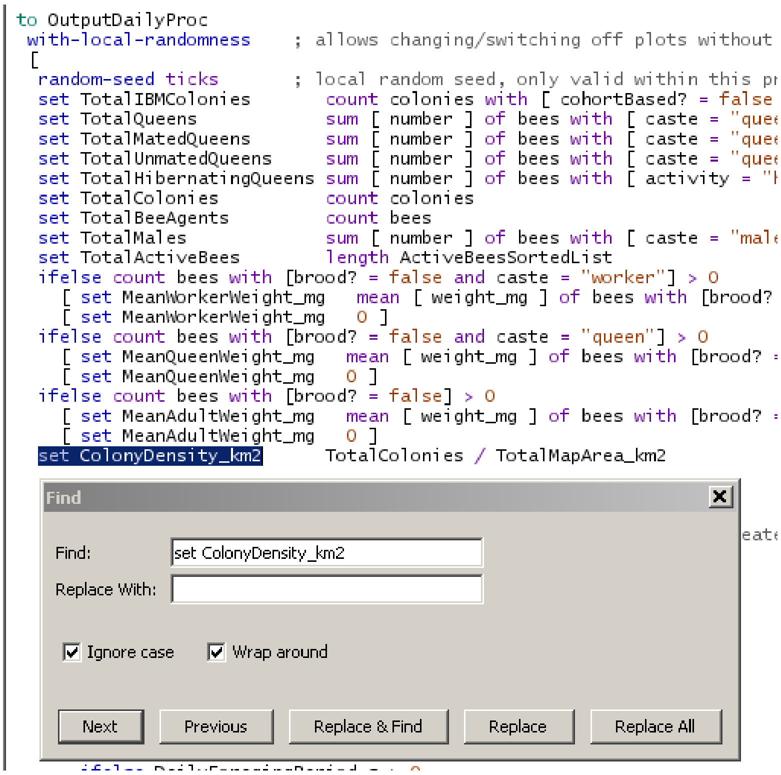

8

8.) More input options are given under "Advanced input" (see section "Bumble-BEEHAVE input options (interface)" for a description).

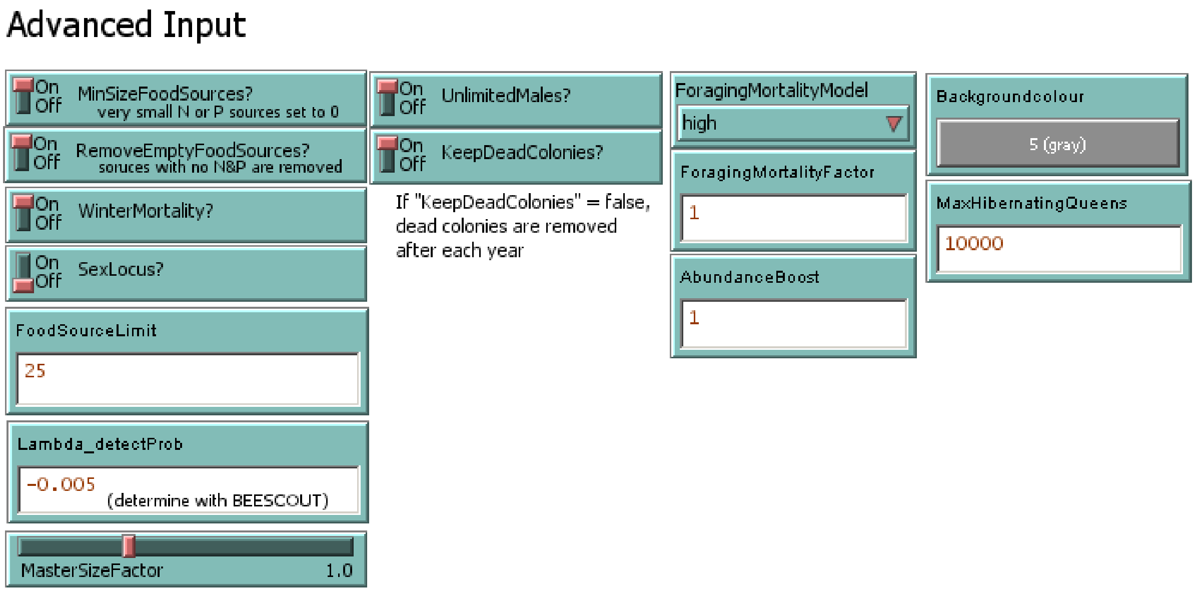

9

9. Display options on "world"

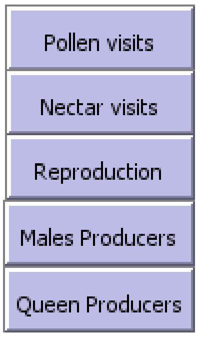

"Pollen visits", "Nectar visits": displays the number of pollen/nectar visits at each food source (white refers to a high number, black to a low number of visits)

"Reproduction": Highlights grid cells where at least one colony had produced reproductives in the past. Yellow grid cells: males had been produced, red: queens had been produced, green: males and queens had been produced

"Males producers", "Queens Producers": displays food source where either males (green) or queens (red) had been produced in the past.

10

10.)
Choose which agents are shown on the "world" by clicking the switches or pressing the buttons on bottom or top of the world:

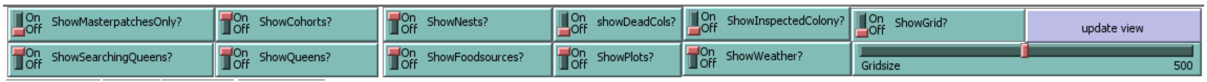

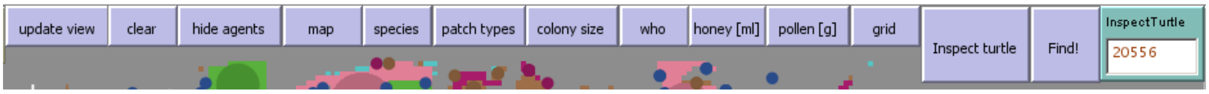

"update view": refreshes the view
"clear": removes labels, grid and the image map
"hide agents": hides all bees, food sources etc.
"map": shows or hides the image map
"species" shows the species names of colonies
"patch types": shows the habitat types of food sources
"colony size": shows the colony sizes
"who" shows the ID of colonies
"honey [ml]": shows the nectar store [ml] of the colonies
"pollen [g]": shows the pollen sore [g] of the colonies
"grid" shows the grid (the grid size defined by "Gridsize" [m])

"Inspect turtle" opens a NetLogo agent monitor for the agent defined in "InspectTurtle"
"Find!" shows the location of the agent, defined in "InspectTurtle"

BEESCOUT 2.0

Creating Bumble-BEEHAVE input files with BEESCOUT

To create input files for Bumble-BEEHAVE, defining the available food sources, we updated theBEEHAVE landscape module BEESCOUT. The following instructions refer to the updated version BEESCOUT 2.0

BEESCOU 2.0 can distinguish more habitat colours (nine instead of four) and it allows to have multiple flower species in each habitat type. The flower species present in a habitat type and their density is defined in the input file, specified by "HabitatsInput" (BEESCOUT 2.0; user interface: section "Definition food patches" (bottom right))

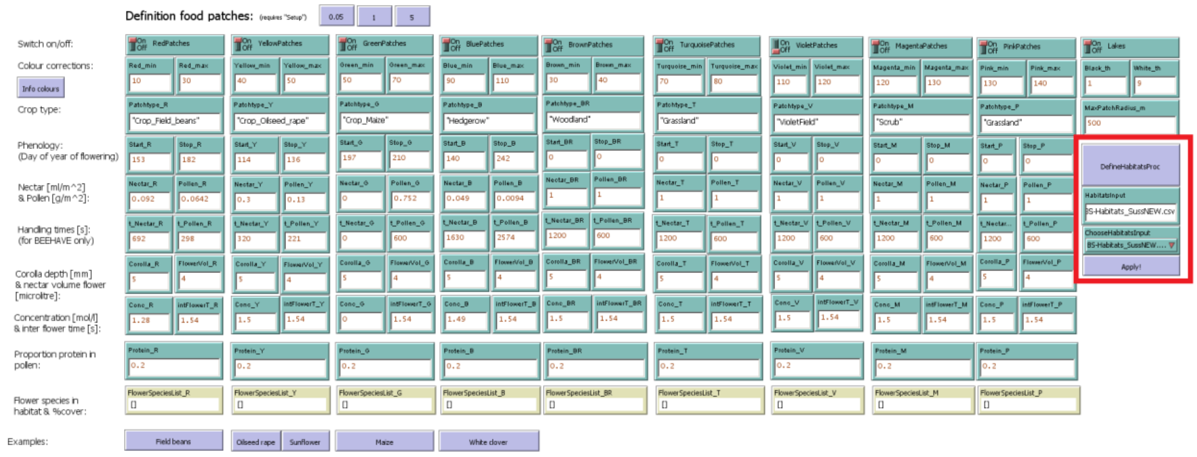

Format of input file "Habitatinput":

Colour(format:FlowerSpeciesList_+abbr.colour)	HabitatType	Bugle	Burdock	Spear_thistle	etc.
FlowerSpeciesList_R	Crop_Field_beans	0	0	0	...
FlowerSpeciesList_Y	Crop_Oilseed_rape	0	0	0	...
FlowerSpeciesList_G	Crop_Maize	0	0	0	...
FlowerSpeciesList_B	Hedgerow	0.119048	0.005	0.0075	...
FlowerSpeciesList_BR	Woodland	0.298333	0.104167	0.007083333	...
FlowerSpeciesList_T	undefined	0	0	0	...
FlowerSpeciesList_V	undefined	0	0	0	...
FlowerSpeciesList_M	Scrub	0.508646	0.05	0.006428571	...
FlowerSpeciesList_P	Grassland	0.028125	0.016667	0.005	...

The first column contains the names of the flower species lists, defined in this line, the second column the habitat type. All other columns list the relevant flower species and their abundances (for details see Supporting Information "SI_07_Methodology for calculating BEESCOUT 2.0 Habitats input file and Bumble-BEEHAVE"). Make sure that the flower species correspond with the flower species listed in the Bumble-BEEHAVE input file "FlowerspeciesFile"

Format if the input file "TextMap"

The "TextMap" file has to be a text (.txt) file with the following format:

201
201
25
[Pink Grey Brown Magenta Green Black Yellow Red Blue]
2 2 2 2 2 2 2 2 2 2 2 2 2 2 2 2 2 2 2 2 2 2 2 2 2 2 2 2 2 2 2 2 2 2 2 2 2...
2 2 2 2 2 2 2 2 2 2 2 2 2 2 2 2 2 2 2 2 2 2 2 2 2 2 2 2 2 2 2 2 2 2 2 2 2...
2 2 2 2 2 2 2 2 2 2 2 2 2 2 2 2 2 2 2 2 2 2 2 2 2 2 2 2 2 2 2 2 2 2 2 2 2...
...

Line 1: a single number defines the number of data columns in the file.
Line 2: a single number defines the number of data rows in the file.
Line 3: a single number defines the "Scaling" i.e. the dimension [m] of each grid cell.
Line 4: in brackets: a list of defined BEESCOUT colours. The position of each colours specifies the number, by which this colour is represented in the following map.
Lines 5+: here, the actual map is defined: each entry represents the colour of one grid cell, the number of entries in each line is given in the first line of the file. Each number refers to one of the colours from line 4, i.e. in the given example: 1 = Pink, 2 = Grey, 3 = Brown etc.

1.) provide an input file of the map (as image or text file). Add the file name as option to the chooser "InputFile" or "TextMap" (with right mouse click on the chooser, "Edit") and select it.

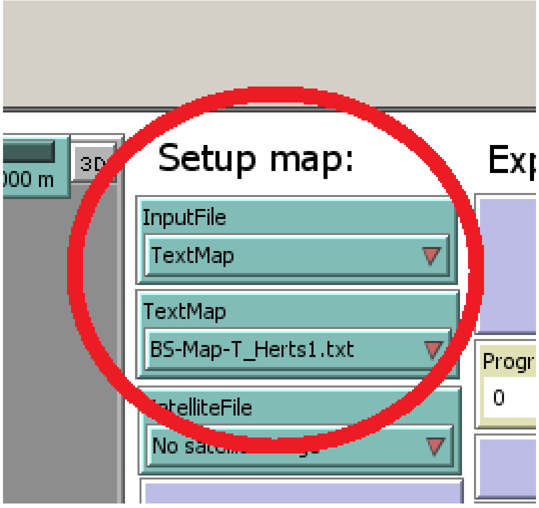

2.) provide a "HabitatsInput" csv-file and insert the file name to the input field

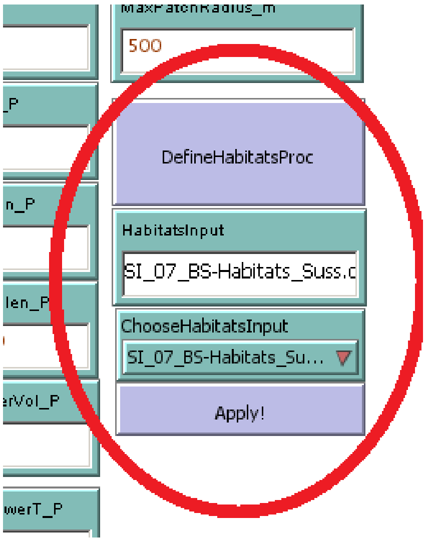

3.) press "Setup" button

4.) provide the names of the files, created as input for Bumble-BEEHAVE (or press the "Create file names" button for a suggestion). Then press "create Bumble-BEEHAVE files" button

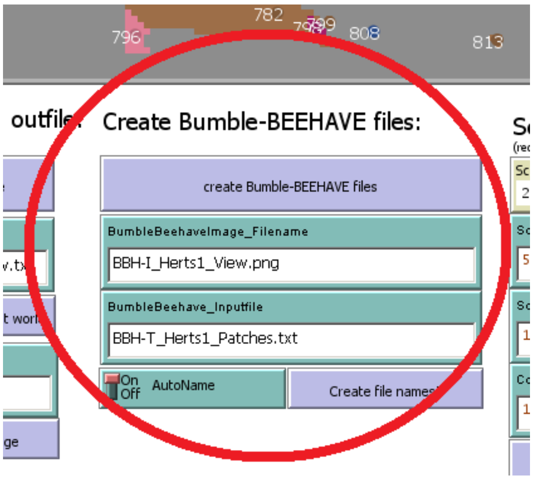

Two files (an image (.pgn) and a text (.txt) file) are created and can be used as input for Bumble-BEEHAVE.

Bumble-BEEHAVE input options (interface)

List of all input options on the interface

VARIABLENAME	Input option	DESCRIPTION
**AbundanceBoost**	input	factor to increase (or decrease) the amount of nectar and pollen at each food source
**B_hortorum**	input	number of initial Bombus hortorum queens
**B_hypnorum**	input	number of initial Bombus hypnorum queens
**B_lapidarius**	input	number of initial Bombus lapidarius queens
**B_pascuorum**	input	number of initial Bombus pascuorum queens
**B_pratorum**	input	number of initial Bombus pratorum queens
**B_terrestris**	input	number of initial Bombus terrestris queens
**Backgroundcolour**	input	colour of the 'matrix' (non-patch area) on the map
**ChooseInputFile**	chooser	a quick way to select a predefined filename as input for "INPUT_FILE" (when pressing "Apply!" button)
**ChooseInputMap**	chooser	a quick way to select a predefined filename as input for "InputMap" (when pressing "Apply!" button)
**Colonies_IBM**	input	(maximal) number of colonies implemented as individual-based
**FlowerspeciesFile**	chooser	input file with the specifications of flower species
**FoodSourceLimit**	input	approx. number of trips a food source must be able to supply with nectar or pollen, otherwise, foodsource might be removed
**ForagingMortalityFactor**	input	is multiplied by MORTALITY_FORAGER_PER_SEC to modify the foraging mortality

**ForagingMortalityModel**	chooser	to set MortalityForager_per_s; 3 options: (1.) "high" (1.0E-05): Visscher&Dukas 1997 (Mortality: 0.036 per hour foraging, honeybees) (2.) "intermediate" (2.14E-06): Schmid-Hempel & Heeb 1991: mortality 30-40% per week (=>35%), survival rate per week: 0.65, assuming 8hrs foraging per day: 7 * 8 * 3600 = 201600 seconds, survival rate/s = 0.65^(1/201600) => mortality rate/s 2.14E-06 (3.) "low" (2.75E-07): Stelzer et al. 2010 (doi:10.1111/j.1469-7998.2010.00709.x), Tab. 1 (from mean of loss rate %/h)
**GenericPlot1**	chooser	defines the graphs shown on associated plot
**GenericPlot2**	chooser	defines the graphs shown on associated plot
**GenericPlot3**	chooser	defines the graphs shown on associated plot
**GenericPlot4**	chooser	defines the graphs shown on associated plot
**GenericPlot5**	chooser	defines the graphs shown on associated plot
**Gridsize**	slider	distance [m] of gridlines, which can be shown on the map
**Input_File**	input	name of the text file read in to define number and specifications of foodsources
**InputMap**	input	name of the image file read in to define the map, (supported formats: BMP, JPG, GIF, and PNG)
**InspectTurtle**	input	ID (who) of a turtle that can be addressed by the buttons "Inspect turtle" or Find!"
**KeepDeadColonies?**	switch	If false, dead colonies are removed after each year
**Lambda_detectProb**	input	to calculate the the probability that a worker of a certain colony finds a certain foodsource, based on the distance [m] between foodsource and colony (detection probability is then e ^ (Lambda_detectProb * relevantDistance_m)). Use the BEESCOUT model to simulate detection probabilities and derive lambda.
**MapAreaIncluded**	chooser	either the "complete" map area is included in the simulation or only one quarter, defined by the user
**MasterSizeFactor**	input	affects the size of elements (turtles) displayed on the map
**MaxHibernatingQueens**	input	maximal number of hibernating queens in the simulation. If exceeded, queens (irrespective of species) are randomly picked and removed
**MinSizeFoodSources?**	switch	If true foodsources offering less nectar or pollen as it is required for ca. than ca. "FoodSourceLimit" foraging trips have their nectar or pollen set to 0.

**N_Badgers**	input	number of badgers (badger's setts) in the simulation; suggestions for initial number of badgers: zero, intermediate (>0–3 setts km2) and high (>3 setts km 2); Reilly & Courtenay 2007 (Preventive Veterinary Medicine 80: 129–142)
**N_Psithyrus**	input	initial number of cuckoo bees
**RAND_SEED**	input	if <> 0: initial seed for the Netlogo pseudo-random number generator; if set to 0, random-seed is not set
**RemoveEmptyFoodSources?**	switch	if true, foodsources that don't provide neither nectar nor pollen (e.g. because MinSizeFoodSources? is true) are removed during Setup
**SexLocus?**	switch	if true, homozygous diploid eggs will develop into males instead of workers or queens (diploid males can survive into adulthood and even mate but cannot reproduce)
**ShowCohorts?**	switch	if true, bee cohorts are shown on the map
**ShowDeadCols?**	switch	if true, dead colonies are shown on the map
**ShowFoodsources?**	switch	if true, foodsources are shown on the map
**ShowGrid?**	switch	if true, a grid is shown on the map
**ShowInspectedColony?**	switch	if true, certain "plotChoices" (e.g. "Colony structures") show the output of a single colony, defines by "inspectTurtle". If false, an average value of all colonies is calculated and shown in these plots
**ShowMasterpatchesOnly?**	switch	if true, non-masterpatches are hidden from the map
**ShowNests?**	switch	if true, colonies are shown on the map in the shape of a bumblebee of the respective species
**ShowPlots?**	switch	if true, "GenericPlots" are updated each time step
**ShowQueens?**	switch	if true, mated queens are shown on the map in the shape of a red circle
**ShowSearchingQueens?**	switch	if true, not-hibernating queens without a colony are shown on the map (in the very bottem left corner)
**ShowWeather?**	switch	if true, the weather symbols (sun/cloud) and the hours of foraging for the current day are shown
**SpeciesFilename**	chooser	name of the input file that provides parameter values of the bumblebee species
**UnlimitedMales?**	switch	if true, queens are allowed to mate, even if no males are currently present in the simulation

**Weather**	chooser	defines the weather conditions as hours of foraging allowed on each day of the simulation
**WinterMortality?**	switch	if true, hibernating queens can die due to winter mortality in the procedure "EmergenceNewQueensProc"
**X_Days**	input	defines how many time steps the model proceeds, when the button "run X days" is pressed
