# Supplementary material for: Bumble‐BEEHAVE: A systems model for exploring multifactorial causes of bumblebee decline at individual, colony, population and community level
Source: J Appl Ecol. 2018 May 22;55(6):2790–801. doi: 10.1111/1365-2664.13165 (PMC6221040; doi:10.1111/1365-2664.13165)
Supplement: Supplementary file 5 [file JPE-55-2790-s005.pdf]

Bumble-BEEHAVE – Model Description

The model description follows the ODD (Overview, Design concepts, Details) protocol, a standard format for describing individual-based models (Grimm et al. 2006, 2010).
Bumble-BEEHAVE was implemented in NetLogo (Wilensky, 1999), version 5.3.1. The program and a user manual are available at http://beehave-model.net/.

1. PURPOSE

The purpose of the model is to explore the colony and population dynamics of bumblebees as a result of the spatial and temporal distribution of forage resources and nesting habitat. Additional factors like weather/foraging conditions, predation by badgers and social-parasitism from cuckoo bees can be added but are implemented in the current version of the model in a relatively simplified way.

Bumble-BEEHAVE simulates in an agent-based approach the life cycle of bumblebees, foraging for nectar and pollen from a variety of forage plant species in a spatially explicit landscape. Starting with an initial number of hibernating queens of up to six European bumblebee species, the foundation of nests in suitable habitat and raising of brood by the queen and later by worker bees is modelled. The population dynamics then results from the number of reproductives, particularly queens, produced by colonies of the same species (Fig. 1).

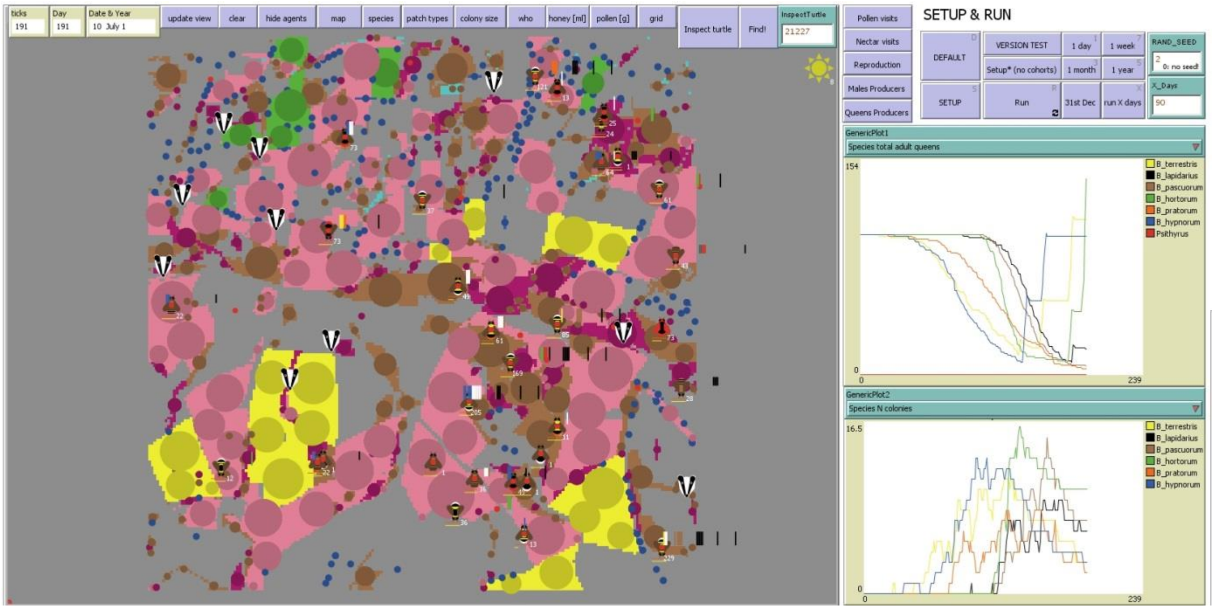

Fig. 1: Screenshot of the Bumble-BEEHAVE interface during a simulation run with badgers being present.

2. ENTITIES, STATE VARIABLES, AND SCALES

I. ENVIRONMENT

Grid cells

The NetLogo *world* (i.e. the main object on the interface, representing the model landscape) is made of a grid of 300 x 210 cells (termed *patches* in NetLogo). The real dimensions of a grid cell varies with the landscape simulated (in the examples provided with the model it is 25 x 25 m). Bumble-BEEHAVE uses these NetLogo *patches* to visualise the map, if an image file, specified by *InputMap* is provided. NetLogo *patches* are also used to show where on the map colonies produced males or queens (Tab. 1).

Table 1: Variables of grid cells (NetLogo *patches*)

VARIABLENAME	DESCRIPTION
nColonies	keeps track of the total number of colonies ever produced on this grid cell (Netlogo patch)
nMalesProduced	keeps track of the total number of males ever produced on this grid cell (Netlogo patch)
nQueensProduced	keeps track of the total number of queens ever produced on this grid cell (Netlogo patch)
pcolorSave	remembers the current colour of a grid cell (Netlogo patch)

Food patches

The simulated, 2-dimensional landscape comprises of a number of flower patches providing nectar, pollen and/or nesting opportunities. Flower patches are implemented as one or more *foodsources* (defined as a NetLogo *breed*). Each *foodsource* represents a single forage plant species, being in flower during a certain time of the year, with a location, area and specifications of flower shape and food production (Tab. 2). To simulate semi-natural habitat, two or more *foodsources* can be "layered", i.e. placed at the same location and linked with each other. One of these layers at each flower patch is called "masterpatch" and addressed for processes concerning the food patch as a whole.

The data is loaded from two input files, defined by *Input_File* and *FlowerspeciesFile*. A third, optional input file, *InputMap*, is an image file, showing a map of the model landscape.

Terminology: We refer to a group of *foodsources* (or "layers") with the same location and the same size, as a "flower patch" (or sometimes "layergroup"). Some statistics concerning the flower patch as a whole (i.e. all *foodsources* forming this flower patch) are saved in a single *foodsource*, the "masterpatch" of this flower patch.

Table 2: Variables of *foodsources*

VARIABLE NAME	DESCRIPTION
area_sqm	size of the *foodsource* (m^2), identical for all 'layers' within a flower patch
colorMemo	saves original colour in which the *foodsoure* is shown, identical for all 'layers' within a flower patch
corollaDepth_mm	average length of the corolla tubes of the flower species represented by this particular *foodsource* ('layer')
cumulNectarVisits	number of all visits of nectar foragers over the whole simulation run at this particular *foodsource* ('layer')
cumulPollenVisits	number of all visits of pollen foragers over the whole simulation run at this particular *foodsource* ('layer')
flowerSpecies_relativeAbundanceList	lists the single flower species represented by this particular *foodsource* ('layer') and its abundance, relative to a reference habitat type (format e.g. ["Common_knapweed" 2.54], indicating that the density of common knapweed here is 2.54 times higher than in the reference habitat)
flowerSpeciesList	lists all flower species and their relative abundances at the current flower patch, identical for all 'layers' within a flower patch (format e.g. [["Bugle" 4.273] ["Burdock" 10][..]..[..]])
id_Beescout	if the input file ("Input_File") was created with BEESCOUT, then id_Beescout refers to the "who" of this food patch as it was identified in the BEESCOUT model; this variable is defined but not used in Bumble-BEEHAVE
interFlowerTime_s	average time a foraging bee spends between two flowers
layersInPatchList	lists "who" of all *foodsources* ('layers') of the flower patch, this particular foodsource is part of
masterpatch?	true for only one *foodsource* ('layer') of each flower patch
masterpatchID	ID ("who") of the (single) 'masterpatch' *foodsource* of each flower patch, hence identical for all 'layers' within a flower patch
nectar_myl	the actual amount of nectar [µl] currently available at a particular *foodsource* (updated after each bee visit)
nectarConcentration_mol/l	the (constant) sugar concentration of the nectar of this particular *foodsource*
nectarFlowerVolume_myl	amount of nectar [µl] available in a single flower (or floret) of the flower species at this particular *foodsource*; a flower can either be full or empty and the proportion of emptied flowers might increase during a day
nectarMax_myl	the maximal amount of nectar [µl] that can be available at this particular *foodsource*
patchInfo	may provide additional information for the user (no other use in the model)
patchType	crop or habitat type of the flower patch (identical for all 'layers' within a flower patch)
pollen_g	the actual amount of pollen [g] currently available at a particular *foodsource* (updated after each bee visit)
pollenMax_g	the maximal amount of pollen [g] that can be available at this particular *foodsource*
proteinPollenProp	the protein content of the pollen at this particular *foodsource*. In the current version of the model, this has NO EFFECT on the bees (e.g. when feeding larvae)

radius_m	the (hypothetical) radius of a *foodsource*, calculated from its area (i.e. assuming a circular shaped flower patch), identical for all 'layers' within a flower patch
startDay	the first day of the year when the *foodsource* ('layer') is in flower and provides resources<br>(it is assumed that startDate < stopDate!)
stopDay	the first day of the year after the flowering period of a *foodsource* ('layer') when no more resources are provided<br>(it is assumed that stopDate > startDate!)

Weather

Weather is not explicitly implemented in the model (e.g. via directly using meteorological data) and hence there is no effect of temperature or humidity on the bee's energy consumption or the survival probability of queens during hibernation. However, it is represented (in the variable "Weather") by the daily allowance of foraging hours (i.e. the maximal time foragers can spend every day on foraging). Furthermore, climate and weather conditions are implicitly taken into account by the phenology of flower patches and by the timing when queens emerge from hibernation, which both have to be provided by the user.

Badgers

To simulate predation by badgers, the NetLogo *breed badgers* is defined and used to distribute a number of badger setts in suitable habitat over the landscape. Badgers in the current version of the model are implemented in a very simplistic way, they do not reproduce or die, and show no other activity than destroying nests they find within their foraging range. *Badgers* are only defined by the variables built-in to all NetLogo *turtles*.

II. BEES

Bumblebees

Bumblebees are implemented as the Netlogo *breed bees*. Each *bee* does either represent a single individual or a 1-day age cohort (with the cohort size specified by *number*). Adult queens are always implemented as individuals. *Bees* differ, among others, in their age (distinguishing between *broodAge* and *adultAge*), *caste* (worker, queen, male or undefined), their *activity* and their size (which affects their tongue length and forage loads) (Tab. 3). Furthermore, *bees* belong to a defined *species* and are usually member of a certain *colony* (except of hibernating and nest searching queens).

Table 3: Variables of *bees*

VARIABLE NAME	DESCRIPTION
activity	current *activity* of a bee, (possible activities: "hibernate", "nestConstruction", "resting", "searching", "returningEmpty", "returningUnhappyN", "returningUnhappyP", "nectarForaging", "collectNectar", "bringingNectar", "expForagingN", "pollenForaging", "collectPollen", "bringingPollen", "expForagingP", "egglaying", "nursing")
activityList	logs the *bee's* activities on current day
adultAge	age [d] of adult bee, being 0 at day of emergence and increasing by 1 in "DevelopmentProc" for bees with stage = "adult"
allelesList	a list with 1 (haploid males) or 2 (females, diploid males) alleles from the bees nuclear DNA, inherited from its mother (and father if diploid). If "InbreedingEffects?" is true, then they refer to the sex alleles with diploid males being sterile; otherwise, no effect on simulation run
brood?	true if the *bee's* "stage" is "egg", "larva" or "pupa", "false" if it is "adult"
broodAge	age [d] of sub-adult *bees*, being 0 when egg is laid and increases by 1 in "DevelopmentProc" for *bees* with stage != "adult"
caste	the *bee's caste*: "undefined", "queen", "worker", "male" (with "undefined" being a diploid egg or larva that can develop into either a worker or a queen)
colonyID	the ID ("who") of the *bee's colony* (-1 for queens without colonies) (colonies are implemented as Netlogo "breed")
cropvolume_myl	amount of nectar [µl] that can be carried by a *bee* (determined in CropAndPelletSizeREP when a new adult *bee* is created/emerges from pupation depending on the *bee's* weight). The value refers to a single bee, hence is multiplied by the cohort size (*number*) when the bee agent collects nectar
cumulIncubationReceived_kJ	cumulative amount of energy [kJ] received (until now) by individual *bee* (in any brood stage) due to incubation
cumulTimeEgg_d	the number of days a *bee* has been an egg (i.e. for eggs equal to broodAge)
cumulTimeLarva_d	the number of days a *bee* has been a larva (i.e. for larvae equal to broodAge - cumulTimeEgg_d)
cumulTimePupa_d	the number of days a *bee* has been a pupa (i.e. for pupae equal to broodAge - cumulTimeEgg_d - cumulTimeLarva_d)
currentFoodsource	ID ("who") of the *foodsource* a foraging bee is currently exploiting
emergingDate	date (time step) when a queen emerges from hibernation
expectation_NectarTrip_s	expected duration [s] of a nectar trip, based on previous experience
expectation_PollenTrip_s	expected duration [s] of a pollen trip, based on previous experience
glossaLength_mm	length [mm] of a *bee's* glossa (proboscis)
knownMasterpatchesNectarList	lists the masterpatchID's of all nectar providing patches ('layergroups') sorted by the distance to the *bee's colony*
knownMasterpatchesPollenList	lists the masterpatchID's of all pollen providing patches ('layergroups') sorted by the distance to the *bee's colony*
mated?	set true when young queens leave their mother's nest (QueensLeavingNestProc)
mtDNA	an allele from the *bee's* mitochondrial DNA, inherited from its mother or - for initial queens - set to a random float number between 0 and 139.9 (i.e. within the range of Netlogo colours) (no effect on simulation run)
nectarLoadSquadron_kJ	amount of energy [kJ] in nectar load carried by a cohort of foraging *bees*
nectarsourceToGoTo	ID ("who")of the *foodsource* the bee will be visiting on its next nectar foraging trip
number	number of *bees* in a cohort: initial value either "batchsize" (*Species*-own variable) if *colony* is "cohortBased?" or 1 if *colony* is individual-based.

personalTime_s	current time of day [s] of a *bee*, updated after each *activity*; set to GET_UP_TIME_s (+ randomTimeToGetUp_s) in UpdateMorning_Proc
ploidy	ploidy of a bee: 1: haploid male, 2: diploid female (or diploid male)
pollenForager?	if true, *bee* is foraging for pollen, otherwise for nectar
pollenLoadSquadron_g	amount of pollen [g] carried by a cohort of foraging *bees*
pollenPellets_g	amount of pollen [g] that can be carried by a *bee* (determined in CropAndPelletSizeREP when a new adult bee is created/emerges from pupation depending on the bee's weight). The value refers to a single bee, hence is multiplied by "number" when the bee agent collects pollen
pollensourceToGoTo	ID/"who" of the *foodsource* the *bee* will be visiting on its next pollen foraging trip
speciesID	ID ("who") of the *bee's species* (*species* are implemented as NetLogo "breed")
speciesName	the *bee's species* name written-out
spermathecaList	a list that stores the allele received from a male by mating. Empty for males and unmated females.
stage	developmental stage of a *bee* ("egg", "larva", "pupa" or "adult")
thEgglaying	threshold that needs to be exceeded by a stimulus to potentially trigger egg laying in a *bee*
thForagingNectar	threshold that needs to be exceeded by a stimulus to trigger nectar foraging in a *bee*
thForagingPollen	threshold that needs to be exceeded by a stimulus to potentially trigger pollen foraging in a *bee*
thNursing	threshold that needs to be exceeded by a stimulus to potentially trigger nursing in a *bee*
weight_mg	the weight [mg] of an individual *bee*

Bumblebee species

Parameter values for bumblebee *species* are stored in the NetLogo *breed species*. They describe i.a. batch size, durations and weights of developmental stages, tongue lengths, suitable nesting habitat, periods of emerging from hibernation, etc. (Tab. 4). The data is loaded from an input file, defined by *SpeciesFilename*. The provided input file "BBH-BumbleSpecies_UK_01.csv" contains data of six common bumblebee *species* in the UK and of a generic cuckoo bee.

Table 4: Variables of bumblebee *species*

VARIABLE NAME	DESCRIPTION
batchsize	number of eggs laid in one batch and hence defining the cohort size
chanceFindNest	daily probability to find a suitable nest site for a searching queen
dev_larvalAge_QueenDetermination_d	the day of larval development (i.e. "cumulTimeLarva_d" and not "broodAge") when it is decided whether a female larva develops into a worker or a queen
dev_Q_DeterminationWeight_mg	minimal weight [mg] a female larva needs to have to possibly develop into a queen (a necessary but not sufficient condition)
devAge_Q_EmergingMax_d	the maximal age ("broodAge") of a queen pupa to emerge
devAge_Q_EmergingMin_d	the minimal age ("broodAge") of a queen pupa to emerge
devAge_Q_PupationMax_d	the maximal age ("broodAge") of a queen larva to pupate
devAge_Q_PupationMin_d	the minimal age ("broodAge") of a queen larva to pupate
devAgeEmergingMax_d	the maximal age ("broodAge") of a worker or male pupa to emerge

devAgeEmergingMin_d	the minimal age ("broodAge") of a worker or male pupa to emerge
devAgeHatchingMax_d	the maximal age ("broodAge") of an egg (male or female) to hatch
devAgeHatchingMin_d	the minimal age ("broodAge") of an egg (male or female) to hatch
devAgePupationMax_d	the maximal age ("broodAge") of a worker or male larva to pupate
devAgePupationMin_d	the minimal age ("broodAge") of a worker or male larva to pupate
devIncubation_Q_EmergingTH_kJ	the minimal, cumulative amount of energy [kJ] from incubation a queen pupa needs to have received during its development to emerge
devIncubation_Q_PupationTH_kJ	the minimal, cumulative amount of energy [kJ] from incubation a queen larva needs to have received during its development to pupate
devIncubationEmergingTH_kJ	the minimal, cumulative amount of energy [kJ] from incubation a worker or male pupa needs to have received during its development to emerge
devIncubationHatchingTH_kJ	the minimal, cumulative amount of energy [kJ] from incubation any egg needs to have received to hatch
devIncubationPupationTH_kJ	the minimal, cumulative amount of energy [kJ] from incubation a worker or male larva needs to have received during its development to pupate
devQuotaIncubationToday_kJ	average amount of energy [kJ] a bee needs to receive daily as incubation during its development
devWeight_Q_PupationMax_mg	the maximal weight [mg] a queen larva can have
devWeight_Q_PupationMin_mg	the minimal weight [mg] a queen larva needs to have to pupate
devWeightEgg_mg	the weight [mg] of an egg
devWeightPupationMax_mg	the maximal weight [mg] a worker or male larva can have
devWeightPupationMin_mg	the minimal weight [mg] a worker or male larva needs to have to pupate
emergingDay_mean	the average day of year when queens of this *species* emerge from hibernation
emergingDay_sd	the standard deviation around "emergingDay_mean" for determining a queen's emerging date from hibernation
flightCosts_kJ/m/mg	energetic flight costs [kJ] of a bee of this *species*, depending on the bee's weight [mg] and and the distance [m] travelled
flightVelocity_m/s	speed [m/s] of a foraging bee when travelling to or back from a *foodsource*
growthFactor	defines the maximal weight gain of a larva during one day: maxWeightGain_mg = (weight_mg * myGrowthFactor) - weight_mg
maxLifespanWorkers	maximal lifespan [d] worker, referring to "adultAge" i.e. excluding brood developement phase
minPollenStore_g	minimal amount of pollen bees are trying to store, independent of expected consumption rates. If the actual pollen store is lower, no eggs are laid.
minToMaxFactor	to calculate the maximal age for a a developmental stage based on the given minimal age, e.g. devAgeHatchingMax_d = devAgeHatchingMin_d * minToMaxFactor
name	*species* name as a string
nestHabitatsList	lists the habitats suitable for nesting for this *species*
nestSiteArea	total area of habitat suitable for nesting
nestsiteFoodsourceList	a set of foodsources which are masterpatches and belong to a habitat suitable for nesting
pollenToBodymassFactor	factor to translate the amount of pollen consumed [mg] into a larva's weight gain [mg]
proboscis_max_mm	maximal possible proboscis length [mm] for a *bee* of this *species*
proboscis_min_mm	minimal possible proboscis length [mm] for a *bee* of this *species*
searchLength_m	length [m] of a scouting trip
seasonStop	day of year when the season for this *species* ends and all *bees* except of hibernating queens die
specMax_cropVolume_myl	maximal possible crop volume [µl] for a bee of this *species*
specMax_pollenPellets_g	maximal possible size of pollen pellets [g] for a *bee* of this *species*
timeUnloading	time [s] to unload a nectar or pollen load to the *colony's* stores

Bumblebee colonies

Bumblebee colonies are implemented as the NetLogo *breed colonies*. *Colonies* are created after a queen has found a suitable nest site. They are defined, amongst others, by a location, nectar and pollen stores, the *bees* belonging to the *colony*, the developmental phase of the *colony* and task stimuli, affecting the decision making of the *bees* (Tab. 5). To not lose data when *colonies* vanish, they are transferred into the *breed deadCols*, instead of just being removed. *DeadCols* do not affect the simulation run and only serve as data storage.

Table 5: Variables of bumblebee *colonies*

VARIABLE NAME	DESCRIPTION
allAdultActiveQueens	number of (not hibernating) adult queens in the *colony*
allAdultMales	number of adult males in the *colony*
allAdultQueens	number of adult queens in the *colony*
allAdults	number of all adult bees in the *colony*
allAdultWorkers	number of all adult workers in the *colony*
allEggs	number of all eggs in the *colony*
allLarvae	number of all larvae in the *colony*
allPatchesInRangeList	lists "who" of all *foodsources* within the foraging distance (i.e. independent of "masterpatch?")
allPupae	number of all pupae in the *colony*
allSourcesInFlowerAndRangeList	lists all *foodsources* (i.e. irrespective of "masterpatch?") within the foraging range that provide nectar and/or pollen
broodDeathBadger	number of eggs, larvae and pupae killed by badger
broodDeathEndSeason	number of eggs, larvae and pupae dying due to end of season
broodDeathsCP	number of eggs, larvae and pupae dying due to competition point
broodDeathsEnergyStores	number of eggs, larvae and pupae dying as nectar stores are depleted
broodDeathsNoAdults	number of eggs, larvae and pupae dying as no adults are left in the *colony*
cohortBased?	if true, *bees* are implemented as cohorts (with number equals batch size), otherwise as individuals
colonyAge	number of days passed since foundation of *colony* (until death of *colony*)
colonyFoundationDay	time step (ticks) when *colony* was founded
colonysize	total number of *bees* (brood, workers, queens, males) in the *colony* (i.e. with "colonyID" = who of the *colony*)
colonyWeight_mg	total weight [mg] of *bees* (brood, workers, queens, males) in the *colony* (but without stores or wax etc.)
competitionPointDate	time step (ticks) of the *colony's* competition point (i.e. when workers start to lay and destroy eggs)
eggDeathsIncubation	number of eggs dying due to lack of incubation
energyNeedToday_kJ	approximate amount of energy [kJ] required today to feed the *colony's* larvae
energyStore_kJ	amount of energy [kJ] stored as nectar in the *colony*
eusocialPhaseDate	time step (ticks) when the *colony* enters the eusocial phase (i.e. when the first workers emerge)
idealEnergyStore_kJ	amount of energy [kJ] the *colony* tries to store as nectar
idealPollenStore_g	amount of pollen [g] the *colony* tries to store

larvaDeathsIncubation	number of larvae dying due to lack of incubation
larvaDeathsWeight	number of larvae dying as they haven't reached the minimum weight for pupation
larvaWorkerRatio	"allLarvae" divided by "allAdultWorkers"
masterpatchesInRangeList	all masterpatches within foraging range, determined only once, when *colony* is created
masterpatchesWithNectarlayers InFlowerAndRangeList	all masterpatches within the foraging range where at least one 'layer' provides nectar today (before foraging starts) (i.e. nectarInFlowerAndRangeList without the non-masterpatches)
masterpatchesWithPollenlayers InFlowerAndRangeList	all masterpatches within the foraging range where at least one 'layer' provides pollen today (before foraging starts) (i.e. pollenInFlowerAndRangeList without the non-masterpatches)
nectarInFlowerAndRangeList	lists "who" of all *foodsources* within foraging range that provide nectar today (before foraging starts)
pollenInFlowerAndRangeList	lists "who" of all *foodsources* within foraging range that provide pollen today (before foraging starts)
pollenNeedLarvaeToday_g	approximate amount of pollen [g] required today to feed the *colony's* larvae
pollenStore_g	amount of pollen [g] stored in the *colony*
pupaDeathsIncubation	number of pupae dying due to lack of incubation
queenProduction?	is set true once the *colony's* criteria for queen production are fulfilled
queenProductionDate	(theoretical) date (time step) when the first queen-destined eggs were laid, back calculated on the day when "queenProduction?" is set true (in QueenProductionDateProc). Does not require that any eggs were actually laid on that day.
queenright?	set false after the death of the mother queen
speciesIDcolony	the *species* of the founding queen in numerical format (i.e. "who" of the corresponding *species*-turtle)
stimEgglaying	stimulus to lay eggs
stimNectarForaging	stimulus to forage nectar
stimNursing	stimulus to nurse brood
stimPollenForaging	stimulus to forage pollen
summedIncubationToday_kJ	total amount of energy [kJ] spent on incubating the brood today
switchPointDate	time step (ticks) of the colony's switch point (i.e. when queens starts to lay haploid eggs)
totalAdultsProduced	total number of *bees* (workers, queens, males) that reach adulthood ever produced by this *colony*
totalEggsProduced	total number of eggs (haploid or diploid) ever produced by this *colony*
totalLarvaeProduced	total number of larvae ever produced by this *colony*
totalMalesProduced	total number of males reaching adulthood ever produced by this *colony*
totalPupaeProduced	total number of pupae ever produced by this *colony*
totalQueensProduced	total number of queens reaching adulthood ever produced by this *colony*
totalWorkersProduced	total number of workers reaching adulthood ever produced by this *colony*
**for deadCols only:**	
colonyDeathDay	time step (ticks) when the *colony* died (only defined for "deadCols")

III. INTERFACE

Signs and storebars

The NetLogo *breeds signs* and *storebars* do not represent real entities but provide information for the user on the interface. *Signs* only use NetLogo built-in variables. They differ in their *shape* ("sun", "cloud" or "circletarget") and show today's foraging conditions or help to locate a certain "*turtle*" (an agent in NetLogo, e.g. a *bee*) on the map.

Storebars represent for each bumblebee *colony* shown on the interface the amount of nectar (energy) and pollen stored, relative to an "ideal" amount of nectar and pollen (based on colony size). Their (additional) variables are *maxSize* (the maximal length of the storebar when displayed on the interface), store (defines whether the storebar represents "Nectar" or "Pollen") and *storeColonyID* (ID/"who" of the colony the storebar is associated with).

State variables & global variables

Low level state variables are those variables which describe the current state of the modelled system, and cannot be derived from other variables. Most of the "bees-own" variables, (such as *adultAge*, *activity* etc.), the majority of "foodsources-own" variables (*like area_sqm*, or *pollen_g*) and a few of the "colonies-own" variables (like *energyStore_kJ* or *pollenStore_g*) are considered to be low level state variables. Not considered as low level state variables are auxiliary variables that e.g. keep track of the number of certain individuals (like *TotalColonies*) or do not describe a real entity (like *AssertionViolated*) (Grimm et al. 2006, 2010), hence none of the "species-own", "patches-own", "storebars-own" variables nor any global or local variables are state variables. For a detailed list of all variables including information whether or not they are low level state variables see Supporting Information SI_04. Variables

3. PROCESS OVERVIEW AND SCHEDULING

SETUP

Bumble-BEEHAVE is initialised by running the procedure *Setup*. This clears all agents, sets back time steps to 0, initialises the random-number generator, and calls a number of sub-procedures to upload data from input files and create initial agents (Tab. 6a)**.**

In *ParametersProc*, parameter values values are set.

Then a map of the landscape can be imported (specified by *InputMap*, file formats: bmp, jpg, gif or png) and *foodsources* are created in *CreateFoodsourcesProc*, based on an input files defined by *Input_File* (file format: txt). *CreateLayersProc* then checks if *foodsources* are composed of more than one flower species and - if this is a case - copies of this *foodsource* ("layers") are created (one for each flower species present) and the *foodsource* specific variables are then changed according to the flower data (e.g. start and stop date of the flowering period, amount of nectar and pollen provided, corolla depth etc.) of the flower species they represent (from *FlowerspeciesFile*, file format: csv). For the first copy created,

masterpatch? is set "true" and *masterpatchID* is set to the ID (who) of this new *foodsource*, for all other copies of this particular flower patch, *masterpatch?* is set "false" and *masterpatchID* is set to the ID (who) of the first copy (i.e. the "masterpatch" of this flower patch). This creates a number of *foodsources* at the same location, which are linked with each other to represent a semi-natural habitat (the original *foodsource* is then no longer needed and removed).

Next, bumblebee *species* are created in the procedure *CreateSpeciesProc*. Specifications of *species* are provided in *SpeciesFilename* (file format: csv) and given for six common UK bumblebee *species* (*Bombus terrestris, lapidarius, pascuorum, hortorum, pratorum and hypnorum*) as well as a (general) cuckoo bee (*Psithyrus*). All of these *species* can be present in a simulation run at the same time.

Badgers are then created in *CreateBadgersProc* in a habitat suitable for badger setts. Badgers don't move, die or reproduce but can destroy bumblebee colonies located within the *badger's* foraging range (see procedure *BadgersOnTheProwlProc*). The initial number of badgers is set by the user on the interface (*N_Badgers*).

Then initial bumblebee queens (including cuckoo bees) are created in *CreateInitialQueensProc*, according to the numbers set by the user (interface input: *B_terrestris*, *B_lapidarius* etc.). The date to emerge from hibernation and the queen's weight are randomly determined, based on a normal distribution with *species*-specific mean and standard deviation.

UpdateMorning_Proc is first called in *Setup* and then again at the beginning of each time step. It will be described under the section "GO".

Finally, *signs* to show the current foraging conditions and the nectar and pollen supply of colonies are created (see *CreateSignsProc*) and *OutputDailyProc* determines some output statistics and updates the plots shown on the interface.

Table 6a: Scheduling of "Setup" procedures (initiated by the Setup-button) (without reporter-procedures). In total, 69 procedures are defined of which 19 are reporter-procedures)

SETUP				
	ParametersProc			
	CreateFoodsourcesProc			
		CreateLayersProc		
	CreateSpeciesProc			
	CreateBadgersProc			
		DieProc		
	CreateInitialQueensProc			
	UpdateMorning_Proc			
		UpdateFoodsourcesProc		
		UpdateSeasonalEventsProc		
		EmergenceNewQueensProc		
			DieProc	
			NestSitesSearchingProc	

				DieProc
			PsithyrusNestSearchProc	
				DieProc
			DieProc	
			CreateColoniesProc	
				PatchesInRangeProc
				FoodsourcesInFlowerAndRangeProc
		UpdateColoniesProc		
			DieProc	
		UpdateColonyStoreBarsProc		
		CheckNumbersProc		
	CreateSignsProc			
	OutputDailyProc			
		PlottingProc		

Table 6b: Scheduling of "Go" procedures (called by the various run buttons), addressing all processes of a time step (day) (reporter-procedures are not shown).

GO				
	UpdateMorning_Proc			
		UpdateFoodsourcesProc		
		UpdateSeasonalEventsProc		
			DieProc	
		EmergenceNewQueensProc		
			DieProc	
			NestSitesSearchingProc	
				DieProc
			PsithyrusNestSearchProc	
				DieProc
			DieProc	
			CreateColoniesProc	
				PatchesInRangeProc
				FoodsourcesInFlowerAndRangeProc
		UpdateColoniesProc		
			FoodsourcesInFlowerAndRangeProc	
			DieProc	
		UpdateColonyStoreBarsProc		
		CheckNumbersProc		
	NeedNectarPollenLarvaeTodayProc			
	ActivityProc			
		EgglayingProc		
			EggsParameterSettingProc	
		BroodIncubationProc		
		ForagingProc		

			Foraging_searchingProc	
			Foraging_collectNectarPollenProc	
			Foraging_costs&choiceProc	
				DieProc
				Foraging_PatchChoiceProc
			Foraging_unloadingProc	
	QueensLeavingNestProc			
	FeedLarvaeProc			
	QueenProductionDateProc			
	DevelopmentProc			
		Development_Mortality_AdultsProc		
			DieProc	
		Development_PupaeProc		
		Development_LarvaeProc		
		Development_EggsProc		
	MortalityBroodProc			
		DieProc		
	BadgersOnTheProwlProc			
		DieProc		
	OutputDailyProc			
		PlottingProc		
	DrawCohortsProc			

GO

The *Go* procedure, covering all processes of a single time step, is called one or several times by various run buttons (e.g. "Run", "1 day", "1 week" etc.) (Tab. 6b).

Daily update

After proceeding the time step by one day, all entities are updated (see *UpdateMorning_Proc*). The new date is set (*DateREP*), maximal hours of foraging for today is determined (see *Foraging_PeriodREP*) and *foodsources* are updated (*UpdateFoodsourcesProc*) by setting the nectar and pollen available to the maximal values during the flowering period or to 0 outside of the flowering period. In *UpdateSeasonalEventsProc* not-hibernating queens die at the end of the *species*-specific duration of the season (on day *seasonStop*), males are removed if all potential queens are in hibernation and on each 1st January (*Day* = 1), the number of hibernating queens is - if exceeded - reduced to its maximal value (*MaxHibernatingQueens*, default: 10000), to avoid undue computation time.

In *EmergenceNewQueensProc*, those queens emerging from hibernation today are addressed. Their *activity* changes from "hibernate" to "emerging", and their task thresholds for egg laying, nursing and nectar and pollen foraging are updated. As hibernating queens might have been implemented as cohorts while mother queens have to be implemented as individuals, a number of copies (clones) are created from an emerging queen, reflecting their cohort size and then their cohort size (*number*) is set to 1, i.e. they are now actual individuals.

Winter mortality for each freshly emerged queen is then determined on the basis of her (relative) weight (see *WintermortalityProbREP*). *Bees* that die are counted and removed in *DieProc*. Surviving queens then search for nests (see *NestSitesSearchingProc*). The daily probability to find a nest is independent of the habitat available and only depends on the *species*-specific variable *chanceFindNest*. If a queen finds a suitable nest site, a flower patch, suitable for nesting for this bumblebee *species*, is chosen (see *NestSiteFoodSourceREP*) and the nest is randomly located within the (theoretical) radius of that *foodsource* (Note: for simplicity, *foodsoures* are assumed to be round, i.e. they have a x- and y-coordinate, an area and a radius, calculated from the area. The underlying map serves to inform the user but is not used by the model itself). Queens that do not find a nest site might die, otherwise they will continue searching the next day.

Cuckoo bees (*Psithyrus*) are not searching for nest sites but for already established *colonies* of social queens (see *PsithyrusNestSearchProc*). They can invade any nest, irrespective of the host *species*. If they find a nest, they try to access it and, if they manage, might then be killed by the social queen or otherwise might kill the queen themselves. If they have successfully invaded a *colony*, they become a *colony* member and can then start to lay eggs.

Social queens that have found a nest site, can then create a *colony* (see *CreateColoniesProc*). To speed up the foraging procedures, various lists are then created to keep track of *foodsources* and "masterpatches" within foraging range, *foodsources* currently in flower, *foodsources* providing nectar or pollen etc. All *colonies* are then updated (see *UpdateColoniesProc*) which includes refreshing the lists of *foodsources* in flower, counting *bees* of various developmental stages and castes, checking for the switch point (when queen switches to lay haploid eggs) and competition point (when workers compete with the queen and no more eggs survive) and updating the *colony's* nectar and pollen store bars on the interface (see *UpdateColonyStoreBarsProc*). If colonies run out of energy, all *colony* members die. If no more adult bees are left in a *colony*, all brood dies. The morning update is completed after making sure that the number of *bees* present is identical to the number of initial bees plus all bees ever produced minus all *bees* that ever died (see *CheckNumbersProc*), otherwise the procedure *AssertionProc* is called, an error message pops up and the simulation run stops.

Tasks and activities

For each *colony*, the amount of nectar and pollen required today to feed the *larvae* is estimated (see *NeedNectarPollenLarvaeTodayProc*) and will affect the *colony's* foraging stimuli and hence the activities of the *bees*.

Bees can work (e.g. nurse brood) from *GetUpTime_s* till *CallItaDay_s*, which, under default setting, is ca. 24 hours a day. However, foraging can only be done after *Sunrise_s* (8:00 am) for *DailyForagingPeriod_s* seconds (default: 8 hours). As long as the current day time (*Daytime_s*) allows *bees* to work, the *bee* with the lowest *personalTime_s* (which defines the current day time) is determined, becomes active and might perform a task (see *ActivityProc*).

A bee engages in a task, if the stimulus in the *bee's colony* for this task is higher than the *bee's* threshold for this task. The *bee's activity* is then set to this task. Tasks are addressed in the following order: egg laying, nursing, pollen foraging and nectar foraging, where each task potentially overwrites the outcome of the previous task, i.e. a *bee* will only engage in egg laying, if none of the other three stimuli is above the corresponding threshold, whereas if the nectar foraging stimulus is above its threshold, the *bee* will certainly forage for nectar, irrespective of the stimulus-threshold relations of the other three tasks (Fig. 3a). If none of the task stimuli is above their threshold, the *bee* has a break of 30 minutes (*activity* set to "resting") and then might become active again.

Egg laying

If the *bee* ' s *activity* is "egglaying" one or more *bee* agents are produced (see *EgglayingProc*). If the mother's *colony* is "cohort based", then only one new bee agent is created but its cohort size (*number*) is set to the *species* batchsize. If the mother's *colony* is "individual based", then the *species'* batchsize determines how many *bee* agents are produced and *number* of each of these new *bees* is set to 1. New eggs are created as copies (clones) of their mother and then a number of variables has to be changed, e.g. *ploidy* is set to either 1 or 2, depending on whether the mother is a worker (results in ploidy 1) or a queen (results in ploidy 2 or, after the switch point, in ploidy 1), *stage* is set to "egg", brood and adult age is set to 0, their weight is set to the weight of an egg etc. (see *EggsParameterSettingProc*). If *ploidy* is 1, *caste* of the new egg is set to "males". If ploidy is 2, *caste* is usually set to "undefined" and will later (during the larval development) be changed to either "worker" or "queen". However, if a diploid egg is homozygous and the *bee's* alleles represent the sex locus (NetLogo switch *SexLocus?* on interface is "true") then *caste* is set to "male" (note: diploid males can develop into adults and mate with queens but these queens can't reproduce and are removed from the simulation). At last, the costs in terms of nectar and pollen consumption from the *colony's* stores are calculated. The *activity* "egglaying" lasts until the end of the day.

Nursing

If the *bee*'s *activity* is "nursing", then she spents 48 minutes with the brood to incubate it (see *BroodIncubationProc*). The amount of heat transfered to the brood depends on the *bee's* weight (and the number of individuals represented by the *bee* agent) and is equally distributed over all eggs, larvae and pupae and summed up for each of these receiving *bees* (to develop into the next stage, brood has to receive a certain amount of incubation within a certain time frame, otherwise it will die) (Fig. 2).

Nursing/brood incubation implicitly also covers feeding of larvae, although this is not a task in itself and is addressed only once each time step (see *FeedLarvaeProc*).

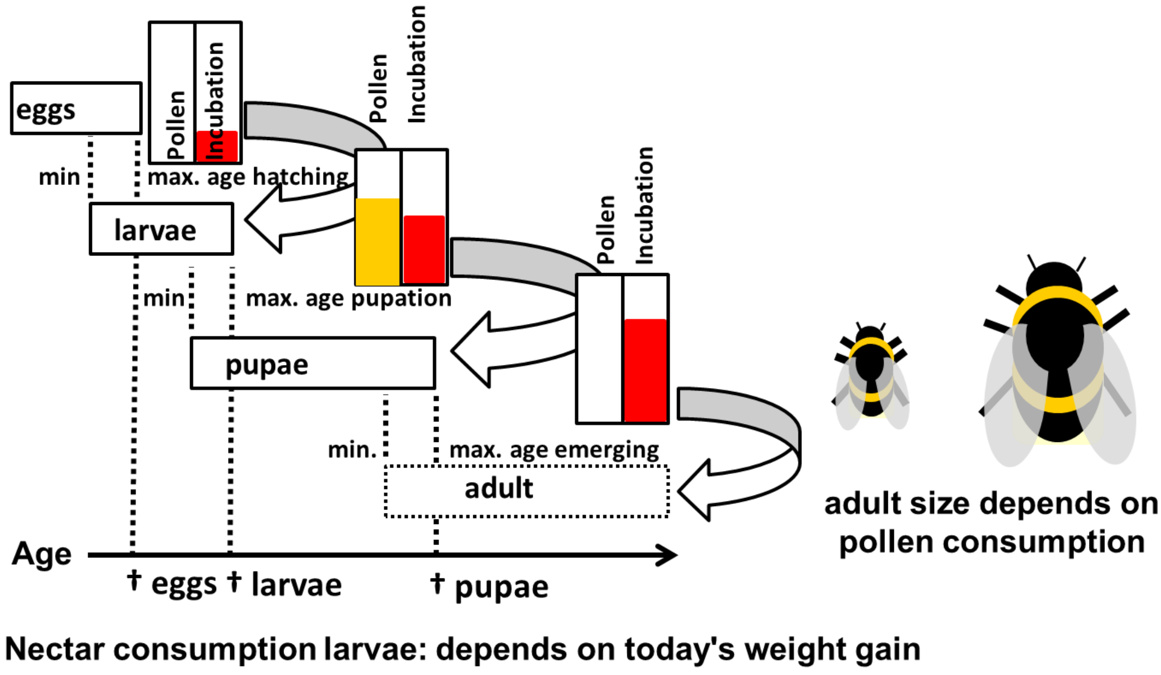

Fig. 2: To develop into the next stage, brood not only has to be of a minimum age, but also has to receive a certain amount of incubation (symbolised by the size of the red bar), which is provided by workers or the queen. Larvae also require feeding of nectar and pollen (symbolised by the the yellow bar). If the minimal requirements are not met within a certain time frame, the brood will die (†**)**. The amount of pollen consumed as larva defines the weight of the adult bee and hence also affects its proboscis length and the amount of nectar and pollen that can be collected per foraging trip.

Foraging

If the *bee*'s *activity* is "pollenForaging" or "nectarForaging", then the *bee* will leave the *colony* to collect food (see *ForagingProc*). Experienced *bees* typically know a number of flower patches, saved in two bee-specific lists for nectar and pollen patches (*knownMasterpatchesNectarList*, *knownMasterpatchesPollenList*) (a flower patch consists of one or several *foodsources*, each *foodsource* represents a single flower species). They usually also remember a single, relatively profitable *foodsource* for nectar (*nectarsourceToGoTo*) and one for pollen (*pollensourceToGoTo*). A foraging *bee* will then either go to the pollen *foodsource* she knows, if she is looking for pollen or to the nectar *foodsource* she knows, if she is looking for nectar. If she doesn't know such a *foodsource*, her *activity* is set to "searching", even if the *knownMasterpatches(Nectar or Pollen)List* is not empty (see *Foraging_searchingProc*) (Fig. 3b).

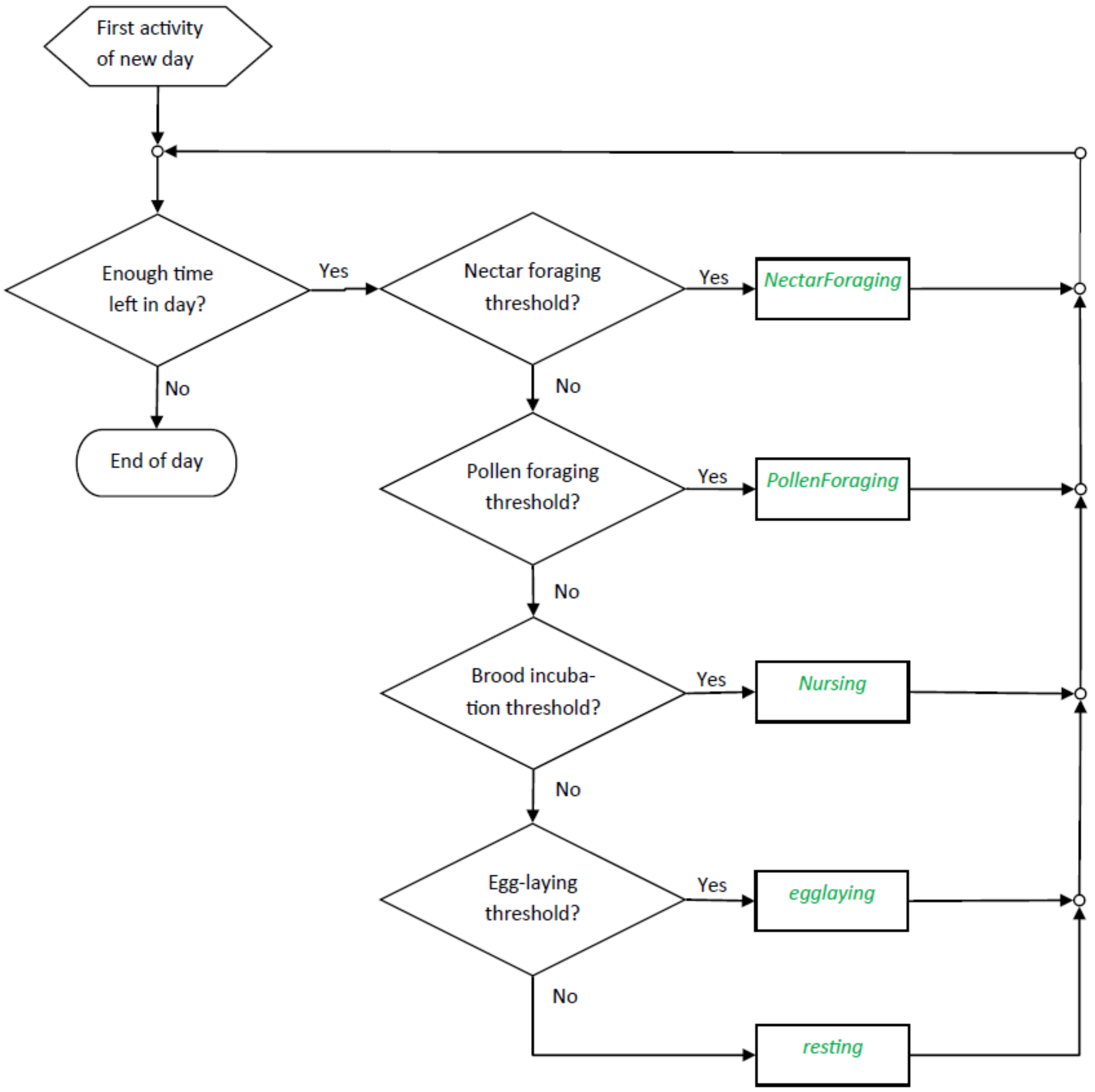

Fig. 3a: Overview of the decision making process: Bees perform a certain task, if the colony's stimulus for this activity is higher than the bee's threshold. If several stimuli exceed their threshold, the bee performs the task with the highest priority.

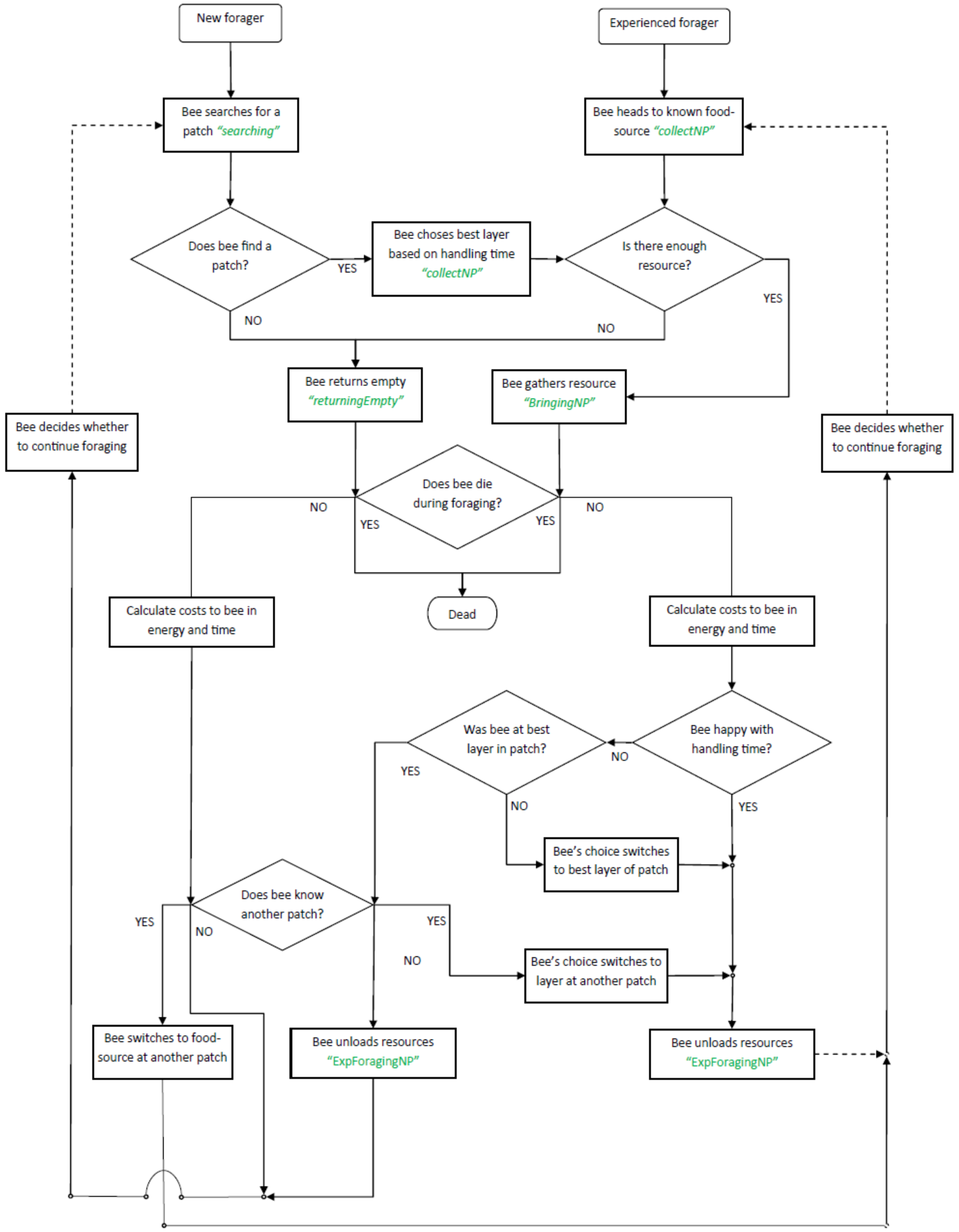

Fig. 3b: Overview of the decision making process of foraging bees.

Searching

To determine whether a flower patch is found, each entry of the *bees'* list of food patches currently offering the right type of forage within the *colony's* foraging range is addressed in a randomized order. For each of these flower patches, the detection probability is calculated, based on the distance between the *colony* and the flower patch (see *DetectionProbREP*), and it is randomly determined whether that flower patch was detected (if several flower patches are detected, only the last one is remembered by the *bee*). The *bee* then chooses the most profitable *foodsource* (flower species) of that flower patch (see *Foraging_bestLayerREP*), which is either based on the time to collect a pollen load or on the energetic efficiency for nectar foraging. This *foodsource* (i.e. a certain flower species at a certain flower patch) is then visited by the *bee*. If no flower patch at all was detected, the *bee* returns empty.

Collecting nectar and pollen

Once a foraging *bee* arrived at the *foodsource* it starts to collect nectar or pollen (see *Foraging_collectNectarPollenProc*). The amount of food removed depends on the *bee's* crop volume or the size of its pollen pellets, but cannot exceed what is actually still available at the *foodsource*. The amount of the forage load is then removed from the *foodsource* and the visit is counted.

Foraging costs, mortality and patch choice

Before a foraging *bee* returns to the *colony*, the costs of the trip in terms of time, energy and mortality are determined (see *Foraging_costs&choiceProc*). For *bees* returning empty, these costs are based on the constant, *species*-specific length of the scouting trip (*searchLength_m*), from which the duration and the energetic costs (taking the *bee's* weight into account) can be calculated. The mortality risk depends on the duration of the trip and the constant, global foraging mortality per second (*MortalityForager_per_s*) and the bee's survival is then randomly determined.

For successful foragers, the trip duration is calculated from the time needed to fly to and return from the patch plus the handling time (see *HandlingTime_s_REP*), i.e. how long it took to collect a full load of nectar or pollen. Energetic costs and foraging mortality are then determined in the same way as for *bees* returning empty.

Following Harder (1983), the calculation of handling time for nectar foragers is based on the *bee's* proboscis length, its weight, the corolla depth of the flower and the nectar volume available in a flower. Additionally, the time to fly from one flower to the next and the proportion of flowers already emptied is taken into account, e.g. if 50% of the nectar at a food source is already removed, the time to fly from one flower to the next and to test whether it has already been emptied is doubled (the actual ingestion time however remains unaffected). Lacking detailed data or existing models, the handling time for pollen foragers is based on poppy flowers and modified by the proportion of flowers already emptied. Hence, *foodsources* exploited by *bees* loose attractiveness as their handling time increases during the day with each visit.

Finally, the duration of the trip is added to the *bee's* personal time and then she reconsiders her current choice of *foodsource* (see *Foraging_PatchChoiceProc*) by comparing the duration of the current trip with the duration of previous trips. *Bees* are more likely to become "unhappy" with their current choice the longer the trip takes, in which case they again check for the best *foodsource* ("layer") of the current flower patch (see *Foraging_bestLayerREP*). If they are not at the best layer already, they will choose the newly determined best layer to be visited on their next foraging trip. If they already *are* at the best layer, then they will go to a new flower patch altogether, drawing on the flower patches they already know. In most cases

bees will go to a randomly chosen flower patch *closer* to the *colony* than the current one, but with a 10% probability they will visit a flower patch somewhat further away. If they do not know such a patch (because they are already foraging at the patch closest to or furthest from the *colony*) they will search for a new flower patch on their next trip.

Unloading

Successfully returning foragers unload their pollen or nectar load to the *colony*'s stores (see *Foraging_unloadingProc*), which again takes some time (*species*-specific variable *timeUnloading*).

Mating and hibernation of young queens

As soon as young queens have developed into adults, they leave their mother's *colony* to mate. (see *QueensLeavingNestProc*). If adult males are currently present in the simulation, one of them is randomly chosen. If no such male is present, mating with an *ad hoc* created male from outside may be allowed (if the NetLogo switch *UnlimitedMales?* is "true"), otherwise the queen is removed from the simulation. When mating, the queen receives the allele from the male and saves it in her spermatheca (*spermathecaList*). She then goes into hibernation and won't be active until she emerges again in the following year.

Feeding larvae and weight gain

Feeding of larvae takes place once a day (see *FeedLarvaeProc*). To grow, larvae need to consume pollen and energy from nectar. Based on the ratio of a *colony's* actual pollen store to a theoretical, "ideal" pollen store, the relative amount of pollen fed to the larvae is calculated. The same calculation is done for the nectar store. The amount of food actually fed is determined by the scarcer food type. For each larva it is then calculated how much pollen would be required for maximal growth (see *MaxWeightGainToday_mg_REP*), and a certain proportion - according to the relative stores - is then fed. E.g. if the nectar store is almost full and the pollen store is half-empty, larvae are fed half of the pollen they would like to consume. Based on the amount of pollen consumed and a *species*-specific assimilation factor (*pollenToBodymassFactor*), the new weight of the larva is calculated. From the total amount of pollen fed to all larvae it is then calculated how much energy from nectar was consumed, to assimilate the proteins from the pollen and the *colony's* stores are reduced accordingly.

Timing of queen and male production

Timing of queen and male production in the model is derived from data on *B. terrestris* by Duchateau and Velthuis (1988). At the beginning of the *colony* development, female larvae develop into workers, whereas later, they may develop into queens (see *QueenProductionDateProc*). The onset of queen production follows, with a few days of delay, the queen's switch from laying diploid eggs to haploid, male eggs. However, this also requires a sufficient number of workers relative to larvae in the *colony*. Diploid larvae of a certain age can then develop into queens instead of workers.

Development and mortality

Development procedures are called in the reversed order of the *bee's* developmental stages (i.e. starting with adults and ending with eggs) to avoid addressing those *bees* entering a new stage twice. On each time step, *bees* age by one day, which is either added to their *adultAge* or

their *broodAge* (see *DevelopmentProc*). Adult males and workers die when they reach a *caste*-specific maximal age (see *Development_Mortality_AdultsProc*). All adult bees can be subject to a constant, daily background mortality risk (see *MortalityAdultsBackground_daily*). However, as adult mortality in real colonies kept captive is negligible (Plowright and Jay 1968), we set the default value of this parameter to 0. Adult mortality outside the *colony* is covered in the model by foraging mortality or winter mortality for hibernating queens.
Also the task thresholds of queens are changing when the *colony* enters the social phase, so that she stops foraging and focuses on egg laying.

Pupae develop into adults, when they reached a *caste*- and *species*-specific minimum age of emerging and summed amount of incubation received (see *Development_PupaeProc*)(Fig. 2). Their stage is then set to "adult" and their task tresholds as well as crop size, pollen pellets, and proboscis lengths are set (for queens also their date of emerging from hibernation is determined).

Similar to pupae, larvae have to reach a certain *caste*- and *species*-specific age and summed incubation to pupate, but additionally, they also need to have a minimum weight (see *Development_LarvaeProc*). For diploid female larvae of undefined *caste*, their *caste* is determined at a specific larval age (see *DetermineCaste_REP*). The larva will develop into a worker, unless the *colony* is ready for queen production (*queenProduction?* is "true") and larvae has already reached a *species*-specific minimal weight.

Eggs only require a *species*-specific minimum age and a certain amount of summed incubation (both independent of *caste*) to hatch (see *Development_EggsProc*).

If *bees* are unable to proceed to the next developmental stage within a certain *caste*- and *species*-specific time frame, they die (see *MortalityBroodProc*). Furthermore, following Duchateau and Velthuis (1988), if a *colony* has passed its competition point, all eggs die, reflecting the reciprocal oophagy of workers and the queen.

Predation by badgers

Once in each time step, *badgers* are addressed to check if *colonies* within the foraging range (735m, Kruuk & Parish 1982) of a badger sett are destroyed (see *BadgersOnTheProwlProc*). If a *badger* comes across a *colony* (19% chance, Kowalczyk et al. 2006) and digs it up (10% chance) all *bees* in this *colony* are killed, resulting in the death of the *colony*.

Output and update of interface

At the end of each time step, some summary statistics are calculated, the plots on the user interface are updated (see *OutputDailyProc* and *PlottingProc*) and the weather signs (indicating today's foraging conditions) and representations of the *colonies'* cohorts on the simulated world are redrawn (see *DrawCohortsProc*).

PLEASE NOTE: Histograms on the model's interface to not take the cohort size into account, hence IBM colonies may be overrepresented in histograms!

4. DESIGN CONCEPTS

Basic principles

The model is agent based with an agent either representing a 1-day age cohort of bees or a single individual, depending on the setup of the *bee's colony*. This allows flexibility in terms of computation speed versus accuracy of output.
Growth and development as well as task performance are based on first principles. Bee weights depend on the amount of pollen consumed as larvae. Activities require energy consumption based on empirical data as well as time to perform them. Decision are made using a stimulus-threshold approach; task are ranked according to their importance for *colony* survival, and bees perform the highest ranked task, where the stimulus is above the threshold. Foraging decision aim to maximise pollen intake per unit time or energetic efficiency for nectar foraging, based on the *bee's* knowledge of food sources. Mortalities depend on the *bees' activity* or nutritional situation.

Emergence

Patterns can emerge at all organisational levels, and these are the primary outputs available:
At the individual level, the *activities* of *bees* and their foraging decisions (when and where to go in the landscape, which forage plants they exploit) as well as their lifespans emerge.
At the *colony* level *colony* dynamics, number and sex ratio of reproductives produced emerge.
At the population level the number of hibernating queens shaping the population dynamics, genetic diversity, and overall sex ratios emerge.
At the landscape level, the number of visits at the various food sources (flower patches and flower species), the locations where colonies produced males and queens and the *colony* densities emerge.

Adaption

Using a stimulus-threshold approach in the decision making process allows the *bees* to perform tasks flexibly and following the actual needs of the *colony*. *Bees* also react to the quality of food provided in the landscape and try to maximise their pollen intake per unit time or energetic efficiency for nectar foraging. With increasing handling time, as a consequence of the depletion of a food source visited by bees, the probability that *bees* switch to another, more profitable *foodsource* increases. Although *bees* possess alleles for one locus, these alleles are in the current model version not linked to a trait (though they may be intrepreted as sex alleles, prohibiting diploid males from reproduction). Linking alleles with bee-specific variables though could be easily added to the model to allow evolutionary processes to be studied.

Objectives

Decisions are made using a stimulus-threshold approach; task are ranked according to their importance for *colony* survival, and *bees* perform the highest ranked task, where the stimulus is above the threshold. Foraging decision aim to maximise pollen intake per unit time or energetic efficiency for nectar foraging, based on the *bee's* knowledge of foodsources.

Learning

Successful foragers memorize all food patches they have detected as well as their distances and the forage type they provide. They also remember the average duration of recent trips and compare it to the duration of the current trip to judge whether to stay at a current *foodsource* or to go to another one.

Prediction

Foraging *bees* remember the average duration of recent trips. The longer the current trip takes in comparison to previous trips, the higher is the probability of a forager to switch to another *foodsource*.

Sensing

Bees perceive time and they are able to sense the *colony* needs via stimuli for tasks (egg laying, nursing, nectar and pollen foraging). This implicitly or explicitly involves knowledge of nectar and pollen stores, of number, developmental stages and castes of nest mates and the demand of larvae for feeding. *Bees* also perceive food patches, can distinguish flowers of different species, find nectar and pollen, judge the sugar concentration of nectar and navigate in the landscape.

Interaction

There are direct interactions when queens mate with males or fight cuckoo *bees* entering the *colony*. Worker *bees* feed and incubate larvae. Indirect interaction takes place within a *colony* via task stimuli (see also ODD section "Sensing" above). *Bees* of different *colonies* (and possibly different *species*) also interact indirectly via collecting and hence reducing nectar and pollen at *foodsources*, leading to increased handling times.
Badgers interact with colonies they find by destroying them and killing all the *bees*.

Stochasticity

Agents (*bees*, *colonies* etc.) are addressed in a random order, Furthermore, a number of processes are based on or contain to some degree stochasticity:

- nest foundation and nest location
- mortality of queens during nest search, foraging mortality, winter survival of hibernating queens
- determining alleles of chromosomal DNA, mtDNA and in spermatheca of initial queens
- variation in time when bees get up in the morning
- date when queens emerge from hibernation
- badgers: finding (and destroying) a bumblebee *colony*
- cuckoo bees: finding a *colony*, entering it, killing the social queen or get killed, or dying during search for a nest
- determination of the queen production date
- switch of queen from diploid to haploid eggg laying
- foraging: finding a *foodsource*, becoming unhappy with current *foodsource*, decision of whether to go to a closer *foodsource* or one further away

Collectives

Bees belong to a certain *species*, where queens can only mate with males from the same *species*. They form *colonies*, founded by a single queen, with each worker and the mother queen contributing to the brood care and nectar and pollen stores. If a *colony* runs out of energy, all *colony* members die. *Colonies* can be invaded by cuckoo bees or destroyed by *badgers*.

Observation

The interface shows a map of the modelled landscape, location, size and type of habitat patches, location of *colonies* (distinguishing different *species*), *colony* age structures (# eggs, larvae, pupae, adults; workers, males, queens), location of badger setts, today's foraging conditions, locations where *colonies* produced males and queens in the past, and nectar and pollen visits of food patches. Five "generic" plots provide a number of output options like *colony* structures, number of *colonies*, number of queens of different *species*, nectar and pollen stores, nectar and pollen available in the landscape. Some of the output can also be created for a single, selected *colony* only.

5. INITIALISATION

The model is initialised in the procedure *Setup* (e.g. by pressing the "Setup" button) (see also Chapter 3 (Processes): Setup). The initial situation in the model is defined by the choices of the user made on the model interface (Tab. 7 and Supporting Information SI_04, sheet "GUI variables"), by parameters set in the procedure *ParametersProc*, and by data importet from input files (as specified by the user). Parameters defined as global variables are listed in Tab. 8. Variables on the interface can be set to the suggested default values by pressing the button "Default". The most important input on the interface are the number of initial bumblebee queens (including cuckoo bees) (*B_hortorum, B_hypnorum, B_lapidarius, B_pascuorum, B_pratorum, B_terrestris, and N_Psithyrus*), the number of badgers (*N_Badgers*) and which input files are loaded to define the landscape (number, location etc. of *foodsources*; *Input_File*), the flower species (nectar and pollen production etc. of forage plants; *FlowerspeciesFile*), and the bumblebee *species* (*SpeciesFilename*). More information for parameterisation and references can be found in Supporting Information SI_04).

In the default setting, 500 *B. terrestris* queens are initially present, but no other bumblebee *species* or badgers. This initial bee population is small, but grows rapidly over the next ca. 10 years to about 8000 hibernating queens.

Rand_Seed, which is not set in *DefaultProc*, defines the seed of the NetLogo pseudo-random number generator and hence the sequence of random numbers created during a model run. If *Rand_Seed* is 0, the seed is automatically set, based on the current date and time. In this case, the results of a run are <u>not</u> replicable.

Table 7 GUI input. Input options provided on interface

VARIABLENAME	Default value	DESCRIPTION
AbundanceBoost	1	factor to increase (or decrease) the amount of nectar and pollen at each *foodsource*
B_hortorum	0	number of initial Bombus hortorum queens
B_hypnorum	0	number of initial Bombus hypnorum queens
B_lapidarius	0	number of initial Bombus lapidarius queens
B_pascuorum	0	number of initial Bombus pascuorum queens
B_pratorum	0	number of initial Bombus pratorum queens
B_terrestris	500	number of initial Bombus terrestris queens
Backgroundcolour	5	colour of the 'matrix' (non-patch area) on the map
ChooseInputFile	"BBH-T_Suss1.txt"	a quick way to select a predefined filename as input for "Input_File" (when pressing "Apply!" button)
ChooseInputMap	"BBH-I_Suss1.png"	a quick way to select a predefined filename as input for "InputMap" (when pressing "Apply!" button)
Colonies_IBM	0	(maximal) number of colonies implemented as individual-based
FlowerspeciesFile	"BBH-Flowerspecies_Suss.csv"	input file with the specifications of flower species
FoodSourceLimit	25	approx. number of trips a *foodsource* must be able to supply with nectar or pollen, otherwise, foodsource might be removed
ForagingMortalityFactor	1	is multiplied by MORTALITY_FORAGER_PER_SEC to modify the foraging mortality
GenericPlot1	"SpeciesQueenabundance"	defines the graphs shown on associated plot
GenericPlot2	"speciesNcolonies"	defines the graphs shown on associated plot
GenericPlot3	"Foodavailable"	defines the graphs shown on associated plot
GenericPlot4	"Colonystructures"	defines the graphs shown on associated plot
GenericPlot5	"Speciesabundance"	defines the graphs shown on associated plot
Gridsize	500	distance [m] of gridlines, which can be shown on the map
Input_File	"BBH-T_Suss1.txt"	name of the text file read in to define number and specifications of foodsources
InputMap	"BBH-I_Suss1.png"	name of the image file read in to define the map, (supported formats: BMP, JPG, GIF, and PNG)
InspectTurtle	1	ID (who) of a turtle that can be addressed by the buttons "Inspect turtle" or Find!"
KeepDeadColonies?	TRUE	If false, dead colonies are removed after each year
Lambda_detectProb	-0.005	to calculate the the probability that a worker of a certain *colony* finds a certain foodsource, based on the distance [m] between foodsource and *colony* (detection probability is then e ^ (Lambda_detectProb * relevantDistance_m)). Use the BEESCOUT model to simulate detection probabilities and derive lambda.
MasterSizeFactor	1	affects the size of elements (turtles) displayed on the map
MaxHibernatingQueens	10000	maximal number of hibernating queens in the simulation. If exceeded, queens (irrespective of *species*) are randomly picked and removed
MinSizeFoodSources?	TRUE	If true foodsources offering less nectar or pollen as it is required for ca. than ca. "FoodSourceLimit" foraging trips have their nectar or pollen set to 0.

N_Badgers	0	number of badgers (badger's setts) in the simulation
N_Psithyrus	0	initial number of cuckoo bees
RAND_SEED	*[no default value for RAND_SEED defined]*	initial seed for the Netlogo pseudo-random number generator (if ≠ 0; otherwise (i.e if 0), random-seed is not set)
RemoveEmptyFoodSources?	TRUE	if true, foodsources that provide neither nectar nor pollen (e.g. because MinSizeFoodSources? is true) are removed during Setup
SexLocus?	FALSE	if true, homozygous diploid eggs will develop into males instead of workers or queens (diploid males can survive into adulthood and even mate but cannot reproduce)
ShowCohorts?	TRUE	if true, bee cohorts are shown on the map
ShowDeadCols?	FALSE	if true, dead colonies are shown on the map
ShowFoodsources?	TRUE	if true, foodsources are shown on the map
ShowGrid?	FALSE	if true, a grid is shown on the map
ShowInspectedColony?	TRUE	if true, certain "plotChoices" (e.g. "*Colony* structures") show the output of a single *colony*, defines by "inspectTurtle". If false, an average value of all colonies is calculated and shown in these plots
ShowMasterpatchesOnly?	FALSE	if true, non-masterpatches are hidden from the map
ShowNests?	TRUE	if true, colonies are shown on the map in the shape of a bumblebee of the respective *species*
ShowPlots?	TRUE	if true, "GenericPlots" are updated each time step
ShowQueens?	TRUE	if true, mated queens are shown on the map in the shape of a red circle
ShowSearchingQueens?	TRUE	if true, not-hibernating queens without a *colony* are shown on the map (in the very bottem left corner)
ShowWeather?	TRUE	if true, the weather symbols (sun/cloud) and the hours of foraging for the current day are shown
SpeciesFilename	"BBH-BumbleSpecies_2016-10-25_devQweight_mod.csv"	name of the input file that provides parameter values of the bumblebee *species*
UnlimitedMales?	TRUE	if true, queens are allowed to mate, even if no males are currently present in the simulation
Weather	"Constant8hrs"	defines the weather conditions as hours of foraging allowed on each day of the simulation
WinterMortality?	TRUE	if true, hibernating queens can die due to winter mortality in the procedure "EmergenceNewQueensProc"
X_Days	90	defines how many time steps the model proceeds, when the button "run X days" is pressed

Table 8 Parameters defined as global variables and their default values

VARIABLENAME	Default value	DESCRIPTION
CallItaDay_s	24 * 3600	time of day when bees stop working
CohortSymbolSize	0.75 * MasterSizeFactor	to calculate size of bee cohort graphics, when displayed on interface map
ColonySymbolSize	9 * MasterSizeFactor	to calculate size of *colony* graphics, when displayed on interface map
ColorIBM	24	color of *colony* graphics, representing individual-based colonies
DailyForagingPeriod_s	"Constant 8 hrs"	today's time allowance for foraging, set in Foraging_PeriodREP (identical for all BB *species*); in current version set to constant value (8 hrs per day)
DailySwitchProbability	0.13	daily probability of a *colony* to switch to laying aploid eggs (if larvae:workers ratio is sufficient)
EnergyFactorOnFlower	0.3	reduces energy spent on flying while a bee is in a flower patch (in Foraging_costs&choiceProc)
EnergyHoney_kJ/ml	22.67	energy content [kJ/ml] of stored nectar/honey
EnergySucrose_kJ/mymol	0.00582	energy content of sucrose [kJ/µmol]
EnergyRequiredForPollen Assimilation_kJ_per_g	6.2	energy [kJ] required to digest and assimilate proteins from pollen [g] consumed
FoodsourceSymbolSizeFactor	1.5 * MasterSizeFactor	to calculate size of foodsource graphics, when displayed on interface map
ForagingRangeMax_m	758	maximal foraging range [m] of the bumblebees (for all *species*)
GetUpTime_s	1	beginning of the working day
LarvaWorkerRatioTH	3	to determine a *colony*'s switch point and queen production date (larvaWorkerRatio needs to be smaller than LarvaWorkerRatioTH to switch /produce queens)
MaxLifespanMales	30	maximal lifespan [d] of male bumblebees
MetabolicRateFlight_W/kg	488.6	metabolic rate during flight
MinFoodSourceSymbolSize	2.5 * MasterSizeFactor	to calculate minimal size of foodsource graphics, when displayed on interface map
MortalityForager_per_s	0.00001	mortality risk per second outside the nest
N_ForeignAlleles	24	number of available alleles from which one is randomly drawn, if a queen mates when no males are present but "UnlimitedMales?" is set true
NestSearchTime_h	6	time spent by a queen searching a nest site to calculate the queen's mortality risk (i.e. does NOT affect the probability to find a nest site)
NotSetHigh	1E+15	auxiliary variable with a high value
NotSetLow	-1 * NotSetHigh	auxiliary variable with a low (i.e. negative) value
QueenDestinedEggsBeforeSP_d	5	First queen destined egg is laid ca. 5d before the *colony*'s switch point
QueenSymbolsize	2 * MasterSizeFactor	to calculate the size of queen graphics, when displayed on interface map
StepWidth	0.5 * MasterSizeFactor	cohort graphics on map move to the right each day by this distance
Sunrise_s	8 * 3600	time of day when first foraging may take place

6. INPUT DATA

Up to four input files can be loaded during setup:

Definition of bumblebee species: *SpeciesFilename*

In the default setting, *SpeciesFilename* is set to "BBH-BumbleSpecies_UK_01.csv", which provides the parameterisation for six common UK bumblebee species: *Bombus terrestris*, *B. lapidarius*, *B. pascuorum*, *B. hortorum*, *B. pratorum* and *B. hypnorum* and for an unspecific cuckoo bee (*Psithyrus*). The file is imported in the procedure *CreateSpeciesProc*.
SpeciesFilename refers to a file in the "csv"-format. The first line serves as header, each of the following lines represents a bumblebee *species*. More *species* can be added to this file.
It is important that the captions of each column is not changed, they have to be identical to the names of the *species*-specific variables they define (e.g. the column "batchsize" contains the the values for *batchsize* of each *species*). The first column needs to define the *species* name (format example: B_terrestris), the order of the other columns could be changed.
Tab. 9 lists the caption and a data example of the *SpeciesFilename* input file. *Species* and *names* are strings (but *do not* require double qoutes), *nestHabitatsList* is a list defining the habitat types (as strings, *with* double quotes) suitable for nesting.

Table 9: Caption and a data example of the *SpeciesFilename* input file

Variable name/caption	Format example
species	B_terrestris
name	B_terrestris
emergingDay_mean	91
emergingDay_sd	28
nestHabitatsList	["Grassland" "Garden" "Hedge" "Scrub" "Woodland" "Nestboxes"]
proboscis_min_mm	6.9
proboscis_max_mm	11.1
growthFactor	1.88
seasonStop	305
maxLifespanWorkers	60
batchsize	12
flightVelocity_m/s	5
searchLength_m	2500
timeUnloading	165
specMax_cropVolume_myl	173
specMax_pollenPellets_g	0.15
minToMaxFactor	2
devAgeHatchingMin_d	5
devAgePupationMin_d	12.9

devAgeEmergingMin_d	23.8
devWeightEgg_mg	1.5
devWeightPupationMin_mg	62.4
devWeightPupationMax_mg	249.5
pollenToBodymassFactor	1
dev_Q_DeterminationWeight_mg	0
devAge_Q_PupationMin_d	17
devWeight_Q_PupationMin_mg	590
devWeight_Q_PupationMax_mg	980
devAge_Q_EmergingMin_d	32
dailyNestSiteChance	0.2
dailyNestSiteChance	0.2

Definition of foodsources: *Input_File*

In the default setting, *Input_File* is set to "BBH-T_Suss1.txt", which provides the definitions of *foodsources*, derived from a 5 x 5km area in Sussex, UK. *Input_File* refers to a file in "txt"-format and is imported in the procedure *CreateFoodsourcesProc*.

The first line contains a single value, defining the edge length [m] of a grid cell forming the simulated landscape (i.e. the scale of a NetLogo "patch" in the NetLogo "world" shown on the interface), e.g. 25 means that a grid cell has the dimensions of 25 x 25m

The second line (optional) contains again a single number, defining the number of *foodsources* listed in the file.

The third line contains the header with the caption of each data column, each of the following lines defines one (or more) *foodsources* (**Tab. 10**).

Note that *patchType* and *info* are string variables in double quotes, *flowerSpeciesList* is a (nested) list, all other variables are numbers. If *flowerSpeciesList* is empty (i.e. []), the line represents a single *foodsource* (e.g. a crop). If *flowerSpeciesList* is not empty, it contains a number of sub-lists, each sub-list representing a flower species and its relative abundance, e.g. [["Bugle" 4.273] ["Burdock" 10] ["Ground_ivy" 1.52] ...]. (Relative abundance refers to the default nectar and pollen production per m^2 of this species, defined in the *FlowerspeciesFile*). Each flower species will be implemented as *foodsource* ("layer") (see also Chapter 2 (Entities): "Food patches" and Chapter 7 (Submodels): "CreateFoodsourcesProc" and "CreateLayersProc").

Bumble-BEEHAVE *Input_Files* can be created with the (updated) version of the BEEHAVE landscape module BEESCOUT.

Please note that *TotalMapArea_km2* has to be set by the user to the total area [km^2] of the simulated landscape (which might be smaller than the area of the whole NetLogo "world") .

Table 10: The *Input_File* text file defines the available food sources. Variables marked with * are not used by the model.

Header (3rd line)	first patch (4th line)	second patch (5th line)
id	0	1
patchType	"Crop_Maize"	"Scrub"
patchColour	55	125
xcor	102.582	131
ycor	200.6	205
size_sqm	68750	625
quantityPollen_g	51700	625
proteinPollenProp*	0.2	0.2
quantityNectar_l	0	0.625
concentration_mol/l	0	1.5
startDay	197	0
stopDay	210	0
corollaDepth_mm	5	5
nectarFlowerVolume_myl	4	4
intFlowerTime_s	2.5	2.5
flowerSpeciesList	[]	[["Bugle" 4.273] ["Burdock" 10] ["Ground_ivy" 1.52] ...]
info*	"no info"	"no info"

Definition of forage plant species: *FlowerspeciesFile*

In the default setting, *FlowerspeciesFile* is set to "BBH-Flowerspecies_Suss.csv", which provides the parameterisation for the 37 wildflower and crop species mapped in our example Sussex landscapes. *FlowerspeciesFile* refers to a file in "csv"-format and is imported in the procedure *CreateLayersProc* (Tab. 11).

Similar to *SpeciesFilename*, the first line serves as header, each of the following lines represents a forage plant species. *Flowerspecies* is a string variable in double quotes, all other variables are numbers, with *startDay* and *stopDay* being integers.

Table 11: The input file *FlowerspeciesFile* is a csv file, defining forage plant species that might serve as food sources.

Header (1st line)	1st species (2nd line)	2nd species (3rd line)
Flowerspecies	"Bugle"	"Burdock"
pollen_g/flower	0.00065	0.00043
nectar_ml/flower	0.00081	0.002289
proteinPollenProp	0.072104	0.11179
concentration_mol/l	0.824738	0.886487
startDay	120	181
stopDay	211	272
corollaDepth_mm	10	3.9

nectarFlowerVolume_myl	0.809667	2.289
intFlowerTime_s	0.6	0.6

Map of modelled area: *InputMap*

InputMap defines an image file representing a crop or habitat map of the simulated area. In the default setting it is set to "BBH-I_Suss1.png", showing the habitat types of one of the 5 x 5km areas we mapped in Sussex, UK. The supported file formats are BMP, JPG, GIF, and PNG. Loading a map is optional, the information is not used by the model and only serves as additional information for the user.

Please make sure that the map loaded corresponds to the *Input_File* and that *TotalMapArea_km2* (input field on interface) is set to the total area [km^2] of the simulated landscape (which might be smaller than the area of the whole NetLogo "world").

7. SUBMODELS

A description of standard NetLogo commands can be found on the NetLogo website: https://ccl.northwestern.edu/netlogo/docs/dictionary.html
For commands related to the csv extension (used for loading csv input files) see:
https://ccl.northwestern.edu/netlogo/docs/csv.html

Bumblebee biology, life cycle and general rationales

In this section we give a short, general overview of the life cycle of bumblebees and provide some justifications for the implementation the modelled processes. The model was developed with European/UK bumblebees in mind, but it should be adjustable with small changes to bumblebees elsewhere.

Hibernation and winter mortality

Biology:
Bumblebees, unlike honeybees, do not overwinter as a colony but instead only the (mated) queens survive the end of the season by burying themselves in the ground or under litter to depths of usually less than 10cm (Alford 1969a). Queens remain in their winter quarters for ca. 6-9 months (Alford 1975) until they emerge in spring.

During hibernation queens lose weight, largely due to spending energy resources stored in the fat body (Alford 1969b, Holm 1972). Heavier queens have higher probabilities to survive hibernation. For *B. terrestris* Beekman et al. (1998) found that queens with wet weight of less than 0.6g prior to diapause did not survive hibernation under laboratory conditions, independent of the temperature (-5 to +10C).

Implementation:

The model starts on 1st January with an initial number of queens being in hibernation. Queens are inactive in the model until they emerge from hibernation. On the day of their emergence, winter mortality is determined (*WintermortalityProbREP*), based on Beekman et al. (1998). We re-drew their Fig. 1B to calculate the weight-dependent survival probability from the proportion of survivors to survivors + non-survivors. We expected a sigmoid curve, however, survival probability dropped for very large queens. Beekman et al. provide the following explanation: "*One would expect that queens with the highest weight will survive diapause. It is therefore surprising that the initial weight distribution of dead queens exceeds that of the surviving queens (Figure 1B and 1C). However, in 1993 the average initial weight of the queens was highest and in this period the most severe diapause regimes (6 or 8 months) were started. Since the majority of the queens that were given a treatment with a length of 6 or 8 months died, the initial weight distribution of dead queens exceeds that of the surviving queens.*"
Assuming this drop of survival probability for heavy queens was an artifact, we fitted a sigmoid curve to the left side of the survival curve only. We then related the absolute wet weight to the minimal and maximal weight of a *B. terrestris* queen in the model. This approach allows us to determine the winter survival probability for queens of any *species*, based on their weight, relative to the minimal and maximal weight of queens of their *species*.

Main procedures covering these processes:

WintermortalityProbREP

Searching nests

Biology:
Bumblebee species differ in their preferences for nest sites and nesting habitats with some nesting overground, preferring rough, tussocky grassland and others nesting underground, using pre-existing holes, particularly abandoned nests of rodents (Alford 1975, Goulson 2010). Gardens and linear features like hedgerows or woodland edges show high nest densities (Osborne et al. 2008a). A queen spends a few days to several weeks on searching a suitable nest site (Alford 1975). McFrederick and LeBuhn (2006) find a correlation between the number of rodent holes and bee abundance, Heinrich (1979) reports of queens fighting for desirable nest sites, which indicates that nest sites could be a limiting factor.

Implementation:
The number of (potential) nest sites in reality can not be easily estimated. In most cases, their number will be (much) higher than the number of established colonies, particularly for species not relying on abandoned mammal nests. For this reason, we were not able to get useful estimates of potential nest site densities for different habitats and bumblebee species. We hence followed a very simplistic approach and assume that the number of nest sites is unlimited in any habitat, suitable for nesting. Whether or not a queen finds a nest site is determined by a constant, daily probability, independent of the area of available nesting habitat. Queens not finding a nest site are then subject to mortality, derived from an estimate of hours spent on searching and the bees' foraging mortality. As a result, densities of newly founded nests in the model could be very high (particularly in artificial landscapes), but due to competition for nectar and pollen and mortality of foraging queens, nest densities will soon decline.

Although this approach is a strong simplification of the actual processes and future versions of the model might come up with a more realistic implementation, it does not lead to unrealistically high colony densities, when modelling real landscapes.
The quality of a nest site (other than its location and hence distances to foraging patches) is not addressed in the model, not only because we do not have data on that but also because it would add considerably more complexity to the model, in terms of the decision making process of the queen when finding a nest of not very high quality, but nest quality should then also have effects on e.g. the energy requirements during brood incubation, possibly in interaction with ambient temperatures or maximal size of the brood nest in small cavities, etc.

Main procedures covering these processes:

NestSitesSearchingProc

Colony initiation

Biology:
After a queen has found a suitable nest site, she starts to store nectar and pollen in the nest (Alford 1975). She then lays a first batch of eggs on the pollen lump from which after ca. 4 days the larvae hatch. Depending on the way larvae are fed, bumblebees can be divided in two groups "pocket-makers" where larvae feed collectively and "pollen-storers" where larvae are fed individually, resulting in smaller size variation of workers than in pocket-makers (Goulson 2010). Once the first generation of larvae has pupated, the queen lays a second batch of eggs (Duchateau & Velthuis 1988). With the emergence of the first workers the colony enters the eusocial phase: the queen focuses on egg laying and the workers take over foraging and brood care (Duchateau & Velthuis 1988).

Implementation:
Calculations of task stimuli were set up in a way to replicate the behaviours described above. The queen first collects enough nectar and pollen before she lays a first batch of eggs, and, after their pupation, she then lays a second batch of eggs. Once workers are present, the queen stops foraging and specialises on egg laying. The *bees'* developmental stages are eggs, larvae, pupae and adults, development from one stage to the next is described in the section below. We did not distinguish between pocket-makers and pollen-storers as in most cases the vast majority of colonies modelled will be "cohort-based", i.e. a single bee agent in this case does not represent an individual but a group of bees of the same age. As the different feeding regimes of pollen-storers and pocket-makers result in different size variations *within* a batch of bees of the same age, it cannot be represented by a single agent, as this agent has the average weight or size of the cohort.

Main procedures covering these processes:

ActivityProc
StimEgglayingREP
StimNursingREP
StimForagingNectarREP
EgglayingProc

ThresholdLevelREP

Brood care and brood development

Biology:
Bumblee brood of all stages needs to be incubated, initially by the queen and later by workers (Heinrich 1979). Larvae additionally need to feed on pollen and nectar. The sizes attained by larvae are directionally proportional to the amount of food they are given (Goulson 2010) and high pollen quality accelerates the maturation of larvae (Moerman et al. 2015). The developmental times are approximately five days for eggs to hatch, fourteen days for larvae before pupating and another fourteen days until adult workers emerge, so in total roughly five weeks (Alford 1975). Reports on brood mortality range from ca. 5% (eggs to larvae mortality; Duchateau & Velthuis 1988) to about 66% (eggs to adults; Alford 1975).

Implementation:

Nursing *bees* in the model transfer heat to the brood nest. The energy transferred is calculated from the mass of incubating bees and distributed to all brood agents. Each brood agent sums up the energy from incubation (*cumulIncubationReceived_kJ*). Eggs, larvae or pupae can only proceed to the next developmental stage if they have received enough incubation (e.g. *devIncubationEmergingTH_kJ*: minimal energy received for an egg to hatch).
Furthermore, larvae need to consume nectar and pollen, where the amount of pollen consumed determines the larval growth. The pollen efficacy (i.e. how pollen consumption translates into weight gain) is set for each *species* in the input file defining the bumblebee *species*, we suggest a value of 1 (Moerman et al. 2017, Fig. 1: approx. average of mixed pollen diets). Finally, development into the next stage has to take place within a certain time frame, defined a *caste* specific minimal and maximal age (e.g. *devAgeHatchingMin_d* and *devAgeHatchingMax_d*), otherwise the bee dies. As brood mortality can be very low (Duchateau & Velthuis 1988), we did not implement an additional daily background mortality for brood.

Main procedures covering these processes:

BroodIncubationProc
DevelopmentProc
Development_PupaeProc
Development_LarvaeProc
Development_EggsProc
MortalityBroodProc

Production of males and queens

Biology:

In social bumblebees, reproductives are produced towards the end of the colony life cycle, when the queen switches from laying fertilized eggs developing into workers to laying unfertilized eggs, developing into males (Goulson 2010). This change is accompanied by

worker bees starting to lay (unfertilized) eggs themselves causing increased levels of aggression, including extensive mutual oophagy (Honk et al. 1981, Bloch & Hefetz 1999, Cnaani et al 2000a).

In *B. terrestris*, diploid larvae may develop into queens between the switch point (SP), when the mother queen lays haploid eggs, and the competition point (CP), after which almost all eggs are killed due to oophagy (though eggs laid before the the competition point will still develop into adults) (Duchateau & Velthuis 1988, Duchateau et al. 2004, Lopez-Vaamonde et al 2009).

Larvae developing into queens gain significantly more weight than worker destined larvae, particularly in pollen-storing bumblebee species (Goulson 2010). However, it is unclear whether the weight of a larva triggers the development into a queen or whether increased weight is the consequence of a larva's development into a queen (Ribeiro 1999, Ribeiro et al. 1999), though at least in *B. terrestris*, the latter seems to be the case (Cnaani et al. 1997, 2000b, Pereboom et al. 2003).

In some cases, when a bee is homozygous at the sex determining locus, fertilized eggs do not develop into workers, but males (Beye et al. 2003). Unlike in honeybees, diploid bumblebee males are viable (Duchateau et al. 1994). They can also mate with queens, however, these queens are not able to establish a colony (Duchateau & Marien 1995).

Young adult queens and males stay in their mother's nest for a few days and may take part in brood incubation before they finally leave (Alford 1975). The queens then mate with usually a single male only (Schmid-Hempel & Schmid-Hempel 2000), whereas males are able to mate multiple times (Tasei et al. 1998). Soon after mating, queens take up their winter quarters (Alford 1975).

Implementation:

We followed Duchateau & Velthuis (1988) to implement these processes:

They found that 50% of their colonies switched early, with the SP 9.8 ± 2.4 days after emergence of the first worker, but only when the larva/worker ratio was below 3 (their Fig.2). We estimated that these conditions were fulfilled for about 5 days, hence resulting in a daily probability to switch (when the L:W ratio is below 3) of ca. 13% (0.13^5 = 0.5). For reasons of simplicity we assume that the queen switches within one day from laying only diploid eggs to laying only haploid eggs, whereas Duchateau & Velthuis (1988) suggest that this transition takes several days.

They also found that first eggs to develop into queens were laid ca. 5d before SP (their Fig. 4) but again only if the L:W ratio was below 3. Based on these assumptions we can determine when the *colony* starts to produce queens (see *QueenProductionDateProc*).

From their Fig. 6 we calculate CP as 0.7 * onset of queen production + 15.5 [in days of the eusocial phase] with a maximum of 45d (see *CompetitionPointDateREP*). After CP, egg mortality in the model is 100%. We therefore ignored egg laying of workers as they won't contribute to the *colony*'s production of males (though this would be feasible by reducing the workers' threshold for egg laying after CP).

Due to lack of data, we used this mechanism to determine production of males and queens for all *species*, though in reality there might be considerable differences between *B. terrestris* and other species.

The production of diploid males is an option in the model. In this case, the *bee's* locus is assumed to be the sex locus and homozygous, diploid *bees* will develop into (adult) males. These males can mate with queens, in which case the queen is removed from the simulation as she won' be able to produce a *colony*.

The queens leave their mother's nest when they develop into adults. For simplification, they then immediately mate and go into hibernation. *Bees* in the model possess one locus and during mating, the queen receives and saves the male's allele.

Main procedures covering these processes:

UpdateColoniesProc
QueenProductionDateProc
CompetitionPointDateREP
QueensLeavingNestProc

Foraging

Biology:

Bumblebees forage for nectar and pollen and may collect either or both forage types during one trip (Free 1955a,b). Foraging decisions depend on the needs of the colony, with the presence of larvae increasing the collection of pollen (Free 1955b).
Bumblebees can forage under a wide range of weather conditions, including temperature close to or even below freezing (Alford 1975) and their foraging activities seem to be quite robust against temperature, wind speed and cloud cover (Peat & Goulson 2005), Nevertheless, based on a huge data set, Sanderson et al. (2015) found a positive impact of increasing air temperature and solar elevation and a negative impact of rainfall and windspeed of foraging activity.
There is evidence that bees are trying to maximise their foraging efficiency, with honeybees using energetic efficiency as currency for nectar foraging (Seeley 1994). Foraging efficiency can be increased by foraging closer or at food sources with shorter handling times. Nevertheless, bumbelebees do not forage very close (< 50m) to the colony (Dramstad et al. 1996) but can have a foraging range of several hundred to over 1.5 km (Knight et al. 2005, Osborne et al. 2008b).
Handling times for nectar depend on the flower sizes and shapes (like corolla depth) but also on the bees' sizes and tongue lengths (Brian 1957, Holm 1966, Inouye 1980, Harder 1985), as well as their experience (Peat & Goulson 2005). Some bumblebee species practice "nectar robbing" and access nectar without pollinating by biting holes in the corolla (Stout et al. 2000, Goulson 2010, Irwin et al. 2010).
Bumblebees tend to stay loyal to a forage patch (Dramstad et al. 1996) and they can establish traplines and visit food sources in a certain order (Lihoreau et al. 2012).
During foraging, bees are exposed to a considerable mortality risk, e.g. by crab spiders, robberflies or birds (Rodd 1980, Morse 1986, Schmid-Hempel & Heeb 1991, Stelzer et al. 2010).
In contrast to honeybees, successfully returning bumblebee foragers do not recruit nestmates to a specific food source, but they might stimulate other workers to leave the nest and search for food and possibly transfer information via scent (Dornhaus & Chittka 2001, 2004).

Implementation:
Depending on the needs of the *colony*, stimuli for nectar and pollen foraging are calculated, affecting the decision-making process of the *bees*. As weather conditions have relatively little

impact on the foraging activities, the maximal foraging hours per day (*Weather*) are set to a constant value.
Food sources are patches that can be composed of one or more flower species. For each flower species in each foraging patch, the amount of nectar and pollen still available today is kept track of. Individual flowers or the exact behaviour of a bee within a food source are not modelled. Choosing food sources is based on efficiency, either to minimise the total trip duration in pollen foragers or to maximise energetic efficiency in nectar foragers. The calculation of the nectar handling times are based on a model by Harder (1983), taking tongue length and corolla depths into account, for pollen foragers, due to lack of data, handling times are simply calculated on the basis of the food sources' depletion. Nectar robbing is currently not an option in the model. *Bees* have a high probability to return to the food patch they are currently exploiting but we did not include traplining as this activity would be below the spatial resolution of the model. Stimulation to forage is not considered as the model environment does not provide short-lasting foraging opportunities that would make this behaviour beneficial.

Main procedures covering these processes:

ForagingProc
Foraging_searchingProc
DetectionProbREP
Foraging_bestLayerREP
HandlingTime_s_REP
Foraging_SortKnownPatchesListREP
Foraging_collectNectarPollenProc
Foraging_costs&choiceProc
DieProc
Foraging_PatchChoiceProc
Foraging_bestLayerREP
Foraging_unloadingProc

SETUP

Purpose: sets up the model world in its inital state

Called by: "Setup" Button

Asking agents: none

Calling*:
ParametersProc
CreateFoodsourcesProc
CreateSpeciesProc
CreateBadgersProc
CreateInitialQueensProc
UpdateMorning_Proc
CreateSignsProc
OutputDailyProc

* Calls of *AssertionProc* are not listed here

For a complete scheduling of all procedures and reporter-procedures see Supporting Information SI_04.

Description

The *Setup* procedure sets the initial state of the model world, i.e. all agents are removed, all grid cells are set to their default initial values, all global variables are set to 0, plots and other output on the interface is cleared. The tick counter (NetLogo function to count the time steps) is re-set, and random-seed (to initialise NetLogo's pseudo-random number generator) is set - either to *RAND_SEED*, if *RAND_SEED* is not equal 0 or otherwise to a number automatically derived from the current date and time. Setting *RAND_SEED* to a value not equal 0 allows the user to exactly replicate a simulation run as the sequence of pseudo-random numbers will always be the same for the same seed.
Then procedures are called to set parameter values and create the initial agents (*foodsources*, initial queens etc.). If *ShowGrid?* is true a grid is shown on the map of the modelled world, with an edge length of *Gridsize* [m].

```
to Setup
  clear-all  ; combines the effects of clear-globals, clear-ticks, clear-turtles,
                       ; clear-patches, clear-drawing, clear-all-plots, and clear-output
  stop-inspecting-dead-agents ; closes all agent monitors
                            ;(ALL agents dead after clear-all!)
  reset-ticks  ; Resets the tick counter to zero, sets up all plots, then updates all plots
  if RAND_SEED != 0 [ random-seed RAND_SEED ] ; if RAND_SEED = 0: seed is based
                                               ; on date & time
  ParametersProc
  CreateFoodsourcesProc
  CreateSpeciesProc
  CreateBadgersProc
  CreateInitialQueensProc
  UpdateMorning_Proc
  CreateSignsProc
  if UpdateInterface? [ OutputDailyProc ]
  if ShowGrid? = true
  [
    ask patches with [ remainder pxcor round (Gridsize * Scaling_NLpatches/m) = 0 ]
      [ set pcolor black ]
    ask patches with [ remainder pycor round (Gridsize * Scaling_NLpatches/m) = 0 ]
      [ set pcolor black ]
    ask patch 290 5
      [ set  plabel-color black set plabel word Gridsize " m" ]
  ]
end
```

ParametersProc

Purpose: sets the values of global variables

Called by: *Setup*

Asking agents: none

Calling: none

Description

The procedure sets the initial values of global variables. References (and calculations) of parameter values are provided in Supporting Information SI_04.

```
to ParametersProc
  ; this procedure sets the GLOBAL parameters of the model
  set SpeciesList [] ; contains the BB species present in a run
  if B_terrestris > 0 [ set SpeciesList fput "B_terrestris" SpeciesList]
  if B_pascuorum > 0 [ set SpeciesList fput "B_pascuorum" SpeciesList]
  if B_lapidarius > 0 [ set SpeciesList fput "B_lapidarius" SpeciesList]
  if B_hortorum > 0 [ set SpeciesList fput "B_hortorum" SpeciesList]
  if B_hypnorum > 0 [ set SpeciesList fput "B_hypnorum" SpeciesList]
  if B_pratorum > 0 [ set SpeciesList fput "B_pratorum" SpeciesList]
  if N_Psithyrus > 0 [ set SpeciesList fput "Psithyrus" SpeciesList]

  set AssertionViolated false ; will be set true when an assertion is not met
  set CallItaDay_s 24 * 3600 ; [s]
  set ColorIBM 24 ; (24 = dark orange)
  set EnergyFactorOnFlower 0.3 ; for honeybees: Kacelnik et al 1986: 0.3
  set EnergySucrose_kJ/mymol 0.00582 ; [kJ/micromol]   342.3 g/mol     from BEEHAVE
  set GetUpTime_s 1 ; 8 * 3600   ; 8:00h a.m.
  set Sunrise_s 8 * 3600
  if ForagingMortalityModel = "high" [ set MortalityForager_per_s 1.0E-05 ]
   ;  (BEEHAVE VALUE: 0.00001, from Visscher&Dukas 1997 (Mort 0.036 per hour foraging)
  if ForagingMortalityModel = "intermediate" [ set MortalityForager_per_s 2.14E-06 ]
   ;  (Schmid-Hempel & Heeb 1991: mortality 30-40% per week (=>35%), survival rate per
   ; week: 0.65, assuming 8hrs foraging per day: 7 * 8 * 3600 = 201600 seconds,
   ; survival rate/s = 0.65^(1/201600) => mortality rate/s 2.14E-06
  if ForagingMortalityModel = "low" [ set MortalityForager_per_s 2.75E-07 ] ;  Stelzer et
   ; al. 2010 (doi:10.1111/j.1469-7998.2010.00709.x), Tab. 1 (from mean of loss rate %/h)
  set MortalityAdultsBackground_daily 0 ; Plowright & Jay 1968: negligible adult
                                        ; mortality in captive colonies (B. ternarius)
  set NotSetHigh 999999999999999  ; preliminary, high value, for a variable
  set NotSetLow -1 * NotSetHigh ; preliminary, low value, for a variable
  set QueenSymbolSize 2 * MasterSizeFactor ; relative size of queens displayed on GUI/world
  set ColonySymbolsize 9 * MasterSizeFactor ; .. rel. size of colonies...
  set FoodsourceSymbolSizeFactor 1.5 * MasterSizeFactor ; .. of foodsources etc.
  set MinFoodSourceSymbolSize 2.5 * MasterSizeFactor
  set CohortSymbolSize 0.75 * MasterSizeFactor
  set StepWidth 0.5 * MasterSizeFactor
  set EnergyRequiredForPollenAssimilation_kJ_per_g 6.2 ; Hrassnig, Crailsheim 2005
  set DailySwitchProbability 0.13  ; derived from Duchateau & Velthuis 1988
  set QueenDestinedEggsBeforeSP_d 5 ; Duchateau & Velthuis 1988 , Fig. 4
  set LarvaWorkerRatioTH 3 ; Duchateau & Velthuis 1988
  set N_ForeignAlleles 24 ; Duchateau et al. 1994
  set FoodsourcesInFlowerUpdate? false
  set NestSearchTime_h 6
  set QueensProducingColoniesList []
  set MetabolicRateFlight_W/kg 488.6 ; Wolf et al. 1999 (Tab. 1, Open air)
  set MaxLifespanMales 30  ; 30d of adult age;
      ; Duchateau & Marien 1995 Ins. Soc. 42:255-266 (1995): 30.48+-10.23;
      ; however: bees were kept in flight-cages hence most likely overestimating life span
end
```

CreateSpeciesProc

Purpose: creates the agents representing bumblebee *species*

Called by: *Setup*

Asking agents: none

Calling: none

<u>Description</u>

A "csv" input file, specifying the parameter values of potential bumblebee *species* is loaded and saved (as nested lists) in the local variable *speciesDataCSV*. The first line of this input file

(and hence the first item of *speciesDataCSV*) is the header of the table containing the names of the parameters and is saved as *header* (Note: in NetLogo lists, the first item of the list is counted as "item 0", the second as "item 1" etc.)
The procedure addresses all items of *speciesDataCSV*, each item representing one line of the original csv input file, containing the data of one bumblebee *species*. In each of these items (lines of the input file) the first (sub-)item (item 0) contains the information from the first cell of the current line in the original csv input file. This first column of the csv input file lists the bumblebee *species* name. If this sub-item is included in (i.e. is a "member" of) *SpeciesList* (a list containing all bumblebee *species* names that are included in the current run, based on the choices of the user and set in *ParametersProc*) then a new *species* is created.
The parameter values of this new *species* are taken from the current item ("?") which represents the data line of this *species* in the csv input file. The location of the correct value for a parameter to be set is determined from the position of the parameter's name in the *header* list.
For example, to set the *species*-specific parameter *emergingDay_mean*, the position of the string "emergingDay_mean" in the *header* list is determined. The value for *emergingDay_mean* is then at this very same position but not in the header but in the variable "?" which stores the line of data currently addressed. The advantage of this approach is that the order of the columns in the input file can be changed without having to change the code, with exception of the first column where the *species* names are defined (see also Chapter 6 (Input data): Definition of bumblebee *species*).

References (and calculations) of *species* parameter values are provided see Supporting Information SI_04.

```
to CreateSpeciesProc
 let speciesDataCSV csv:from-file SpeciesFilename ; a csv input file is loaded and saved
 let header item 0 speciesDataCSV  ; first line of the input file is the header
 foreach speciesDataCSV ; goes through all "lines" in ordered way
  [
    if member? item 0 ? SpeciesList ; if the species (i.e.first entry) of the current
       ; row is member of the SpeciesList (i.e. the list with those bee species added
       ; to the simulation, which was created in ParametersProc)
    [
      create-Species 1 ;
      [
        set name item (position "name" header) ?  ; checks in which column of
            ; the input data the species are listed and uses the value of the current row
        set maxLifespanWorkers item (position  "maxLifespanWorkers" header) ?
        set emergingDay_mean item (position "emergingDay_mean" header) ?
        set emergingDay_sd round (item (position "emergingDay_sd" header) ?)

        set batchsize item (position "batchsize" header) ?
        set flightVelocity_m/s item (position "flightVelocity_m/s" header) ?

        set flightCosts_kJ/m/mg MetabolicRateFlight_W/kg / flightVelocity_m/s
                   / (1000 * 1000 * 1000)
                   ; W/kg = J/s/kg; div. by speed => J/m/kg i.e. 0.001kJ/s/(1000000*mg)
        set searchLength_m item (position "searchLength_m" header) ?
        set seasonStop item (position "seasonStop" header) ?
        set timeUnloading item (position "timeUnloading" header) ?
        set specMax_cropVolume_myl item (position "specMax_cropVolume_myl" header) ?
        set specMax_pollenPellets_g item (position "specMax_pollenPellets_g" header) ?
        set nestHabitatsList []
        set nestHabitatsList read-from-string item (position "nestHabitatsList" header) ?
        set minToMaxFactor item (position "minToMaxFactor" header) ?
        set devAgeHatchingMin_d item (position "devAgeHatchingMin_d" header) ?
        set devAgePupationMin_d item (position "devAgePupationMin_d" header) ?
        set devAgeEmergingMin_d item (position "devAgeEmergingMin_d" header) ?
        set devWeightEgg_mg item (position "devWeightEgg_mg" header) ?
        set devWeightPupationMin_mg item (position "devWeightPupationMin_mg" header) ?
        set devWeightPupationMax_mg item (position "devWeightPupationMax_mg" header) ?
        set pollenToBodymassFactor item (position "pollenToBodymassFactor" header) ?
        set dev_Q_DeterminationWeight_mg
                         item (position "dev_Q_DeterminationWeight_mg" header) ?
        set devAge_Q_PupationMin_d
                         item (position "devAge_Q_PupationMin_d" header) ?
        set devWeight_Q_PupationMin_mg
```

```
                      item (position "devWeight_Q_PupationMin_mg" header) ?
    set devWeight_Q_PupationMax_mg
                      item (position "devWeight_Q_PupationMax_mg" header) ?
    set devAge_Q_EmergingMin_d item (position "devAge_Q_EmergingMin_d" header) ?
    set growthFactor item (position "growthFactor" header) ?
    set proboscis_min_mm item (position "proboscis_min_mm" header) ?
    set proboscis_max_mm item (position "proboscis_max_mm" header) ?
    set chanceFindNest   item (position "dailyNestSiteChance" header) ?
    set devQuotaIncubationToday_kJ 10 / (1.5 * batchsize)
    set devAgeHatchingMax_d devAgeHatchingMin_d * minToMaxFactor
    set devAgePupationMax_d devAgePupationMin_d * minToMaxFactor - devAgeHatchingMin_d
    set devAgeEmergingMax_d devAgeEmergingMin_d * minToMaxFactor -  devAgePupationMin_d
    set devIncubationHatchingTH_kJ devQuotaIncubationToday_kJ * devAgeHatchingMin_d
    set devIncubationPupationTH_kJ devQuotaIncubationToday_kJ * devAgePupationMin_d

    set devIncubationEmergingTH_kJ devQuotaIncubationToday_kJ * devAgeEmergingMin_d

    set dev_larvalAge_QueenDetermination_d   3
    set devAge_Q_PupationMax_d devAge_Q_PupationMin_d
                          * minToMaxFactor - devAgeHatchingMin_d
    set devAge_Q_EmergingMax_d devAge_Q_EmergingMin_d
                          * minToMaxFactor - devAge_Q_PupationMin_d
    set devIncubation_Q_PupationTH_kJ devQuotaIncubationToday_kJ
                          * devAge_Q_PupationMin_d
    set devIncubation_Q_EmergingTH_kJ devQuotaIncubationToday_kJ
                          * devAge_Q_EmergingMin_d ; * Incubation_Q_Factor

    ;Create list of foodSources as nest sites and calculate their total area
    set nestsiteFoodsourceList FoodSources with
               [ (member? patchtype [nestHabitatsList] of myself) AND masterPatch? ]
    set nestSiteArea sum [area_sqm] of nestsiteFoodsourceList

    set minPollenStore_g 0.5 * 0.001 * devWeightPupationMin_mg
                          * batchsize / pollenToBodymassFactor

   if count nestsiteFoodsourceList = 0 and name != "Psithyrus" [output-print (word name
                     " has no suitable nesting foodsources. No colonies will form")]
  ]
 ]
]
end
```

CreateFoodsourcesProc

Purpose: creates the agents representing food sources

Called by: *Setup*

Asking agents: none

Calling: *CreateLayersProc*

<u>Description</u>

If an input map defined by *InputMap* exists, it is loaded (supported image file formats: BMP, JPG, GIF, and PNG) and the colours (*pcolor*) of the grid cells (NetLogo "patches") is set accordingly. The colour of the grid cell is also saved in the variable *pcolorSave*, which allows changing colours to display certain information without losing the original map. This image file is optional and only serves to provide additional information for the user. If *InputMap* is "none" or the file does not exist, then the world's grid cells are shown in grey.

```
to CreateFoodsourcesProc
  ifelse ( file-exists? InputMap )
   [
    import-pcolors InputMap
    ask patches [ set pcolorSave pcolor ]
   ]
   [
```

```
ask patches
  [
    set pcolor 5 ; matrix colour if no map image is available; color 5  = grey
    set pcolorSave pcolor
   ]
]
```

The actual information about resources available in the landscape is saved in a text file (".txt"), specified by *Input_File*. If this file exists in the model's folder, it is opened. The first line of this input file contains a single value, defining the edge length [m] of a grid cell (NetLogo "patch") on the interface, e.g. 25 means that a grid cell has the dimensions of 25 x 25m and hence, *SCALING_NLpatches/m* is set to the reciprocal of this value. The second line (optional) contains again a single number, defining the number of foodsources listed in the file. The third line of the input file contains the header with the caption of each data column, each of the following lines defines one (or more) *foodsources* (see also Chapter 6 (Input data): Definition of foodsources). For each line (starting with line 4) a new *foodsource* is created and the parameter values are read in and saved in the corresponding *foodsource* specific variables. Some auxiliary patch variables are then calculated from the parameters (e.g. *radius* and *size*). *Foodsources* created in this procedure are "masterpatches" (i.e. *masterpatch?* is set true) and their variable *flowerSpeciesList* contains all flower species that occur at this food patch. As *foodsources* in the model only represent a single flower species, for each of the flower species listed in *flowerSpeciesList* a new foodsource needs to be created. This happens in *CreateLayersProc* which is called at the end of the procedure.

```
ifelse ( file-exists? Input_File )
  [
    file-open Input_File
    set SCALING_NLpatches/m precision (1 / file-read) 8
     ; CAUTION! Scaling in BEESCOUT: m/NLpatch <-> in Bumble-BEEHAVE: NLpatches per m
    let dustbin file-read-line
          ; N patches in old input file format or heading in new format
    if length dustbin <= 10 [ set dustbin file-read-line ] ; heading
    while [ not file-at-end? ]
    [
      create-Foodsources 1
      [
        ; imported file format:
        ; id patchType patchColour xcor ycor size_sqm quantityPollen_g quantityNectar_l
        ; concentration startDay stopDay corollaLength_mm nectarFlowerVolume_myl
        ; interFlowerTime_s patchInfo
        set id_Beescout file-read
        set patchType file-read
        set flowerSpecies_relativeAbundanceList (list patchType 1)
        let memoFoodpatchColour file-read
                 ; the colour of the food patch, as shown on the map
        set color memoFoodpatchColour - 1    ; the colour of the food source (= turtle),
                                  ; slightly darker then the food patch to be visible
        set colorMemo color  ; saves original color (for use in buttons)
        set xcor file-read
        set ycor file-read
        set area_sqm file-read  ; [m^2]
        set pollen_g ABUNDANCEBOOST * file-read ; [g]
        set pollenMax_g pollen_g
        set proteinPollenProp file-read
        set nectar_myl ABUNDANCEBOOST * file-read * 1000 * 1000
                 ; [quantityNectar_l: l * 1000 = ml; ml * 1000 = myl]
        set nectarMax_myl nectar_myl
        set nectarConcentration_mol/l file-read  ; [mol/l]
        set startDay file-read    ; day of year
        set stopDay file-read     ; day of year
        set corollaDepth_mm file-read    ; [mm]
        set nectarFlowerVolume_myl file-read  ; [microlitre]
        set interFlowerTime_s file-read        ; [s]
        set flowerSpeciesList file-read        ; [s]
        set patchInfo file-read-line  ; the rest of the line is now read in
        set radius_m sqrt (area_sqm / pi)    ; [m]
        set shape "circle"
        set size FoodsourceSymbolSizeFactor * radius_m * Scaling_NLpatches/m
        if size < MinFoodSourceSymbolSize [ set size MinFoodSourceSymbolSize ]
        ifelse ShowFoodsources? = false
          [ hide-turtle ]
          [ show-turtle ]
        set masterpatch? true
        set layersInPatchList (list who)
        set masterpatchID who
```

```
          ]
        ]
        file-close
      ]
      [
        user-message "There is no such Input_File in current directory!"
      ]
      if MergeHedges? = true  and count foodsources with [ patchType != "Hedgerow" ] > 0
        [MergeHedgesProc]
      CreateLayersProc   ; creates new foodsources from those foodsources with
                           ; multiple species (i.e. with flowerSpeciesList != [] )
      set TotalFoodSources count foodsources
end
```

MergeHedgesProc

Purpose: hedges are often represented by a large number of very small patches. If "MinSizeFoodSources?" (and "RemoveEmptyFoodSources?") are switched on, they may only contain one (Average willow) or very few foodsources. To avoid this, several small patches of hedges can be merged into a single, larger one (without loss of total area).

Called by: *CreateFoodsourcesProc*

Asking agents: none

Calling: none

<u>Description</u>

First, the closest non-hedge food patch for each hedge patch is determined. This information is saved in a list (*fieldsHedgeLinksList*) that contains *who* of the closest non-hedge field and *who* of the hedge.

```
to MergeHedgesProc
let fieldsHedgeLinksList [] ; to link hedges with their closest non-hedge field, format
    ; e.g. [[1 17] [5 29] [1 18]..] each sublist with 2 elements: 1st: who of closest non-
    ; hedge patch, 2nd: who of hedge
  let fieldsWithHedgesList [] ; contains who of all non-hedge patches that are closest
                              ; to at least one hedge patch
  ask foodsources with [ patchType = "Hedgerow" ]
  [
     let singleHedgeMatchList (list who)
     let myField min-one-of foodsources with [ patchType != "Hedgerow" ] [distance myself]
        ; myField saves the (non-hedge) foodsource closest to the current hedge patch
     set singleHedgeMatchList fput [who] of myField singleHedgeMatchList ; this is a 2 item
           ; list, 1st item: who of the hegde's closest non-hedge field, second item who
           ; of the hedge
     set fieldsWithHedgesList lput [who] of myField fieldsWithHedgesList
     set fieldsHedgeLinksList lput  singleHedgeMatchList fieldsHedgeLinksList
  ]
  set fieldsWithHedgesList remove-duplicates fieldsWithHedgesList  ; duplicates are removed
                                                                   ; from the list
```

Then a list (*shortSublist*) is created that only contains *who* of those hedge patches, sharing the same, closest non-hedge field:

```
  foreach fieldsWithHedgesList
  [
    let myFieldID ?
    let hedgesSublist filter [first ? = myFieldID] fieldsHedgeLinksList ; this sublist only
                           ; contains those elements where the current field is present
    let shortSublist []
    foreach hedgesSublist [ set shortSublist lput (item 1 ?) shortSublist ] ; this
       ; shortSublist only contains the who of those hedges, linked to the current field
    let masterHedgeID -1  ; will save who of the hedge patch that will increase in area
```

Finally, hedges sharing the same closest non-hedge patch are merged by summing up their areas in one of those hedge patches, the other hedge patches get an area of 0 and will be removed in *CreateLayersProc*.

```
      foreach shortSublist
      [
        ifelse masterHedgeID = -1 ; in this case, the foodsource is the first hedge at that
                                  ; field and will increase in size
         [ set masterHedgeID ? ]
         [  ; the areas of all other hedge patches are now added to the "master" hedge patch
           let areaToBeAdded_sqm [ area_sqm ] of foodsource ?
           let nectarToBeAdded_myl [nectarMax_myl]  of foodsource ?
           let pollenToBeAdded_g [pollenMax_g]  of foodsource ?
           ask foodsource masterHedgeID
            [
              set area_sqm area_sqm + areaToBeAdded_sqm
              set nectarMax_myl nectarMax_myl + nectarToBeAdded_myl ; if hedges are composed
                  ; of layers/several foodsources, this value will be overwritten
                  ; in CreateLayersProc
              set pollenMax_g pollenMax_g + pollenToBeAdded_g  ; if hedges are composed of
                 ; layers/several foodsources, this value will be overwritten
                 ; in CreateLayersProc
            ]
           ask foodsource ?
            [
              set area_sqm 0
              hide-turtle
            ]
         ]
       ]
     ]

    ask foodsources with [ patchType = "Hedgerow" ]
     [
       set radius_m sqrt (area_sqm / pi)    ; [m]
       set size FoodsourceSymbolSizeFactor * radius_m * Scaling_NLpatches/m
       ;; if size < MinFoodSourceSymbolSize [ set size MinFoodSourceSymbolSize ]
     ]

  end
```

CreateLayersProc

Purpose: adresses flower patches which are composed of at least two flower species and creates for each flower species a "layer", i.e. a *foodsource* with only a single flower species located at a certain flower patch represented by a single "masterpatch". Very small foodsources, providing hardly any nectar or pollen, may be removed.

Called by: *CreateFoodsourcesProc*

Asking agents: none

Calling: none

<u>Description</u>
The local variables *minNectSize_myl* and *minPolsize_g* define the least maximal amount of nectar and pollen a *foodsource* needs to provide, to not be removed, when the switches *MinSizeFoodSources?* and *RemoveEmptyFoodSources?* are both set true. They are calculated from an approximate average nectar and pollen load of a queen *bee*, multiplied by the minimum number of trips (*FoodSourceLimit*, set by user on interface) a *foodsource* is supposed to support. Removing those very small *foodsources* improves computation time but also *colony* performance, as handling times can rapidly increase there.
An input file in the "csv" format specified by *FlowerspeciesFile* contains information of flowering period, nectar and pollen production etc. for each flower species (see also Chapter 6

(Input data): Definition of forage plant species). The data of the csv file is accessed in the same way as described in more detail for *CreateSpeciesProc*. The file is opened and the content saved in the local variable *flowerspeciesDataCSV*:

```
to CreateLayersProc
; if the flowerSpeciesList of food source is not empty (i.e. it usually contains
; several plant species that might be in flower at different times) this procedure then
; creates a single flowerspecies food source (at the same location, area etc) for
; each flowerspecies of the original foodsource. At the end, the original food source
; is removed.

; Remove foodsources with low resource values
; If the switch MinSizeFoodSources? is ON, all foodsources with either nectarMax or
; pollenMax values under a certain threshold will
; have that resource set to 0. This is to prevent foragers from visiting low-resource
; flowers and having very high handling times, leading
; to poor colony performance. The thresholds for nectar and pollen are set below, each
; one being the amount of nectar/pollen an average Bterr
; queen can carry multiplied by the FoodSourceLimit interface variable. For example, if
; the variable is set to 20, the minimum nectar/pollen
; amount at a foodsource is enough for FoodSourceLimit trips by a queen Bterr with a
; crop size of 180myl and pollen pellets of 0.05g.
; If the switch RemoveEmptyFoodSources? is ON, all foodsources with BOTH nectarMax and
; pollenMax levels set to 0 by the above are removed from
; the model, which greatly improves the speed of the model.


 let foodsourcesRemoved false
 let minNectSize_myl FoodSourceLimit  * 180  ; minimum nectar for a foodsource to
                             ; not be removed when MinSizeFoodSources? is true
                             ; amount equal to "foodSourceLimit" number of trips by a
                             ; queen with a crop volume of 180myl
 let minPolsize_g FoodSourceLimit  * 0.05 ; minimum pollen for a foodsource is
                             ; amount equal to "foodSourceLimit" number of trips by a
                             ; queen with pollen pellet size of 0.05g

 ifelse ( file-exists? FlowerspeciesFile )
 [
   let flowerspeciesDataCSV csv:from-file FlowerspeciesFile ; reads flower species data
          ; from csv file and saves it in list, i.e. [[line 1][line 2]..[last line]]
   let header item 0 flowerspeciesDataCSV  ; saves header = first line of csv file
                                          ; (i.e. item 0 of list)
    let allFlowerspeciesList []
```

The names of all flower species present in the input file are then saved in *allFlowerspeciesList*:

```
   foreach but-first flowerspeciesDataCSV ; but-first: ignores header
   [
     let flowerSpec read-from-string item 0 ? ; gets the first value (= flower species)
                                            ; of each column (in actual order)
     set allFlowerspeciesList lput flowerSpec allFlowerspeciesList ;  the species is
                        ; now added to the list containing al possible flower species
   ]
```

Now *foodsources* with *flowerSpeciesList* not empty are addressed, i.e. these *foodsources* represent a flower patch that is composed of at least two flower species. For each of these flower species a new *foodsource* will be created, using the "hatch" command so that the new *foodsource* inherits all variables from its parent foodsource (i.e the one currently addressed):

```
  ask Foodsources with [ flowerSpeciesList != [] ]
   [
     let memoMasterpatchID -1
     foreach flowerSpeciesList  ; for each flowerspecues a new foodsource is created
                                ; (flowerSpeciesList is a Foodsources-own)
     [
       hatch 1
       [
```

Then those variables of the new foodsource are re-set that refer to flower species specifications, as defined in in the input file (*FlowerspeciesFile*). The variable *flowerSpecies_relativeAbundanceList* is a list (with 2 items) that contains a single flower species' name and its relative abundance in the current flower patch (e.g. ["Bugle" 0.236]) and the first item (item 0, i.e. the species name) is saved in the local variable *mySpecies*. If the species name contains the string "Margin" (as in e.g. "MarginRed_clover") the shape of the *foodsource* is set to "fieldmargin", which is a filled circle surrounded by a blue ring.

```
set flowerSpecies_relativeAbundanceList ?
let mySpecies item 0 flowerSpecies_relativeAbundanceList
if member? "Margin" mySpecies   [ set shape "fieldmargin" ] ; margins of
                     ; (crop) fields are presented on the map as a blue ring
```

The date for the current flower species are then copied from *flowerspeciesDataCSV* to the local variable *myDataLine*:

```
let myDataLine item (position mySpecies allFlowerspeciesList + 1)
   flowerspeciesDataCSV   ; myDataLine: the relevant line of the csv file for
         ; this particular flower species; position..+1 to account for header
```

The abundance (i.e. the second item of *flowerSpecies_relativeAbundanceList*, e.g. 0.236 for ["Bugle" 0.236]) is saved as *myRelativeAbundance*. *ABUNDANCEBOOST* is set to 1 per default but can be changed by the user to adjust the total amount of nectar and pollen available in the modelled landscape.

```
let myRelativeAbundance ABUNDANCEBOOST
             * (item 1 flowerSpecies_relativeAbundanceList) ; proportion of
                     ; patch area covered by this species

         ; pollen available at patch: pollen produced by this plant species per m2
; * total area of this foodsource * relative abundance of this flowerspecies
; in the habitat:
         set pollenMax_g area_sqm
                   * myRelativeAbundance
                   * (item (position "pollen_g/flower" header) myDataLine)
         set nectarMax_myl area_sqm
                   * myRelativeAbundance
                   * 1000  ; ul to ml
                   * (item (position "nectar_ml/flower" header) myDataLine)

         set nectarConcentration_mol/l
               (item (position "concentration_mol/l" header) myDataLine)  ; [mol/l]
               ; "position" determines the column with the relevant data
         set proteinPollenProp (item (position "proteinPollenProp" header) myDataLine)
         set startDay (item (position "startDay" header) myDataLine)
         set stopDay (item (position "stopDay" header) myDataLine)
         set corollaDepth_mm (item (position "corollaDepth_mm" header) myDataLine)
         set nectarFlowerVolume_myl
               (item (position "nectarFlowerVolume_myl" header) myDataLine)
         set interFlowerTime_s (item (position "intFlowerTime_s" header) myDataLine)
```

If the switch *MinSizeFoodSources?* is set true then the amount of nectar and/or pollen is set to 0, when it is below a minimal threshold:

```
  ;Set nectar / pollen levels to 0 if smaller than the minimum size
if MinSizeFoodSources? AND nectarMax_myl < minNectSize_myl
  [ set nectarMax_myl 0 ]
if MinSizeFoodSources? AND pollenMax_g < minPolsize_g
  [ set pollenMax_g   0 ]
```

If the switch *RemoveEmptyFoodSources?* is set true then *foodsources* offering neither nectar or pollen are removed:

```
; kill the foodSource if both nectar and pollen are below the respective
```

```
; minimum values and if RemoveEmptyFoodSources? is TRUE
         if nectarMax_myl = 0 AND pollenMax_g = 0 AND RemoveEmptyFoodSources?
           [
              set foodsourcesRemoved true
              die
           ]
```

If the masterpatch hasn't been defined yet for this flower patch (i.e. the local variable *memoMasterpatchID* is still set to -1) it is set now. Hence the first "layer" created will become the masterpatch for this flower patch and all foodsources (flower species) belonging belonging to this flower patch will have their *masterpatchID* set to the ID (*who*) of that first "layer". After a *foodsource* for each flower species is created, the original *foodsource* is no longer needed and is removed. Finally, to foodsources-own variable *layersInPatchList*, containing the ID of all *foodsources* of the current flower patch, is udated:

```
         ifelse memoMasterpatchID < 0  ; if the masterpatch hasn't been set yet..
         [
           set memoMasterpatchID who  ; ..the first foodsource/layer will be the
                                                              ; masterpatch
           set masterpatchID memoMasterpatchID ; (only masterpatchID has to be updated,
                                              ; as 'masterpatch?' is true by default)
         ]
         [
           set masterpatch? false    ; .. for all other 'layers' of the original
                                     ; foodsource, masterpatch? is set false
           set masterpatchID memoMasterpatchID ; set to the first 'layer' created at
                                                  ; this flower patch
           set layersInPatchList []  ; will be populated later
           if ShowMasterpatchesOnly = true [ hide-turtle ]
                ; non-masterpatches might be hidden
         ]
       ]
     ]   ; end of "foreach flowerSpeciesList" loop
     die ; the original foodsource is no longer needed and can be removed
   ]
 ]
 [ if FlowerspeciesFile != "No Input File"
   [ user-message "There is no such FlowerspeciesFile in current directory!" ]
 ]
 if foodsourcesRemoved = true [ output-print "One or more very small food sources
                      removed! To avoid, set RemoveEmptyFoodSources? 'false'!" ]

 ; set layersInPatchList to a list of all foodsources at same location:
 ask foodsources [set layersInPatchList sort [who] of foodsources-here ]

end
```

CreateBadgersProc

Purpose: creates the agents representing badgers

Called by: *Setup*

Asking agents: none

Calling: *DieProc*

<u>Description</u>
The habitat types suitable for *badgers* to burrow a sett are listed in *burrowHabitatsList* and the minimal distance between two setts is defined in *distanceLimit_m* (and *distance_patches* for this distance in grid cells). A list of potential habitat patches for badger setts is then created (*burrowFsSet*), listing the ID (*who*) of "masterpatch" *foodsources* of those habitat types specified in *burrowHabitatsList*.

N_Badgers badgers are created and moved to the location of a randomly picked habitat patch from the *burrowFsSet* list. Suggested numbers for *badgers* range from 0 to 3 for intermediate badger densities (Reilly & Courtenay 2007). After the creation of each *badger*, this list is updated and only takes those "masterpatch" *foodsources* of suitable habitat type into account which do not have any *badgers* within a daius of *distance_patches* grid cells. If no more more habitat patches suitable for *badger* setts are available, the remaining *badgers* die:

```
 to CreateBadgersProc

  let burrowHabitatsList  ["Scrub"]  ; habitats badgers can nest in
  let distanceLimit_m 300  ; badgers cannot be created within this distance
    ; of a current sett (Kruuk 1978, J. Zool., Lond.184, 1-19; Fig. 2)
  let memoX 0
  let memoY 0
  ; convert distance to netlogo patches:
  let distance_patches    distanceLimit_m * SCALING_NLpatches/m
  ; agentset of suitable foodsources:
  let burrowFsSet FoodSources with [ (member? patchtype burrowHabitatsList) AND
                                     masterPatch? ]
  create-badgers N_Badgers ; create the badgers
  [
    ifelse count burrowFsSet > 0 ; check for suitable foodsource
    [
      let chosenFs     one-of burrowFsSet
      ask chosenFs     [ set memoX pxcor set memoY pycor ]
      setxy            memoX memoY
      set size         9 * MasterSizeFactor
      set shape        "Badger"
      ; recreate the agentset, only taking masterpatches without any badgers
       ; in a certain radius into account
      set burrowFsSet  FoodSources with [ (member? patchtype burrowHabitatsList) AND
                                         masterPatch? AND
                                         count badgers-here = 0 AND
                                         count badgers in-radius distance_patches = 0
                                       ]
    ]
    [ DieProc "Badger: not enough habitat!" ]
          ; no badgers if there is no habitat for their burrows!
  ]

end
```

CreateInitialQueensProc

Purpose: creates the agents representing the initial queen *bees*

Called by: *Setup*

Asking agents: none

Calling: *ThresholdLevelREP*, *ProboscisLengthREP*

Description

For each item in the *SpeciesList* (containing the *bee species* present), a number of intial queens (specified by the interface input *B_lapidarius*, *B_pascuorum*, etc.) are created and their initial *bee* parameters are set. As these initial queens are in hibernation (*activity* = "hibernate"), their age is set to 180d (note: adult age of queens has no effect). Alleles are set to random float numbers (with 2^53 possible numbers as output). *Activity* thresholds are set by calling the reporter-procedure *ThresholdLevelREP*. The queen's emerging date is randomly determined, based on a normal distribution with *emergingDay_mean* as mean and

emergingDay_sd as standard deviation. Emerging date needs to be larger than 0 and smaller than the *species*-specific end of the season. The queen's weight is also determined randomly with mean and standard deviation being derived from the minimal and maximal queen pupation weight of this *species*, which then allows to calculate its proboscis length (in the reporter-procedure *ProboscisLengthREP*) as well as *cropvolume_myl* and *pollenPellets_g* (in *CropAndPelletSizeREP*).

```
to CreateInitialQueensProc
  let newQueens 0
  foreach SpeciesList ; lists bee species present
  [
   if ? = "B_lapidarius" [ set newQueens B_lapidarius ] ; numbers specified on interface
   if ? = "B_pascuorum" [ set newQueens B_pascuorum ]
   if ? = "B_terrestris" [ set newQueens B_terrestris ]
   if ? = "B_hortorum" [ set newQueens B_hortorum ]
   if ? = "B_hypnorum" [ set newQueens B_hypnorum ]
   if ? = "B_pratorum" [ set newQueens B_pratorum ]
   if ? = "Psithyrus" [ set newQueens N_Psithyrus ]
   let modelledSpecies ?
   create-bees newQueens  ; newQueens = number of new queens created here
   [
     set shape "circle"
     if ShowQueens? = false [ hide-turtle ]
     set size QueenSymbolSize
     set adultAge 180  ; queens have hibernated (exact age doesn't matter)
     set broodAge 36  ; (exact age doesn't matter)
     set color red
     set brood? false
     set caste "queen"
     set mated? true
     set number 1
     set ploidy 2
     set mtDNA random-float 139.9 ; i.e. within the range of Netlogo colours
     set allelesList list (random-float 139.9) (random-float 139.9)
     set spermathecaList [] ;list (allele)
     set spermathecaList fput (random-float 139.9) spermathecaList
     set colonyID -1 ; i.e. does not belong to any colony yet
     let speciesIDmemo -1
     let speciesNameMemo "noName"
     ask one-of Species with [ name = modelledSpecies ]
       [
         set speciesIDmemo who
         set speciesNameMemo name
       ]
     set speciesID speciesIDmemo
     set speciesName speciesNameMemo
     set stage "adult"
     set thEgglaying ThresholdLevelREP "eggLaying" "QueenInitiationPhase"
     set thForagingNectar ThresholdLevelREP "nectarForaging" "QueenInitiationPhase"
     set thForagingPollen ThresholdLevelREP "pollenForaging" "QueenInitiationPhase"
     set thNursing ThresholdLevelREP "nursing" "QueenInitiationPhase"
     set activity "hibernate"
     set activityList [ ]
     set personalTime_s random (2 * 3600) + (GetUpTime_s - 3600) ; = Start_time_s +- 1hr
                                                            ;(i.e. between 7:00 and 9:00 am)
     let yearEndSeason [seasonStop] of OneSpecies speciesID ; prevent bees from setting
                                                    ; emergingDate past the end of season
     while [ emergingDate <= 0 OR emergingDate >= yearEndSeason]
       [ set emergingDate
             round random-normal [ emergingDay_mean ]
                   of OneSpecies speciesID [emergingDay_sd] of OneSpecies speciesID ]
    ; emerging from hibernation next year on day "emergingDay_mean" (+- s.d.)

     set currentFoodsource -1
     set nectarsourceToGoTo -1
     set pollensourceToGoTo -1
     set pollenForager? false
     set knownMasterpatchesNectarList [ ]
     set knownMasterpatchesPollenList [ ]

     ; determination of the queen's weight:
     let minWeight_mg [ devWeight_Q_PupationMin_mg ] of oneSpecies speciesID
     let maxWeight_mg [ devWeight_Q_PupationMax_mg ] of oneSpecies speciesID
     let meanWeight_mg (maxWeight_mg + minWeight_mg) / 2
     let sd_weight (maxWeight_mg - minWeight_mg) / 4 ; mean +- 2xSD > 95%

     set weight_mg random-normal meanWeight_mg sd_weight
     if weight_mg > maxWeight_mg [ set weight_mg maxWeight_mg ]
     if weight_mg < minWeight_mg [ set weight_mg minWeight_mg ]
     set glossaLength_mm ProboscisLengthREP
     set cropvolume_myl   CropAndPelletSizeREP "nectar"
     set pollenPellets_g  CropAndPelletSizeREP "pollen"
   ]
```

```
  ]
end
```

ThresholdLevelREP

Purpose: reports the thresholds of the four tasks, for *bees* in different *stages*

Asking agents: *bees*

Called by: *QueensLeavingNestProc*, *CreateInitialQueensProc*, *PsithyrusNestSearchProc*, *Development_Mortality_AdultsProc*, *Development_PupaeProc*, *EggsParameterSettingProc*, *EmergenceNewQueensProc*

Calling: none

Input: *thType*, *situation*

Description

The input variable *thType* defines the task (egg laying, pollen or nectar foraging, nursing) a *bee* is asking its threshold for and the input variable *situation* describes the developmental *stage*, *caste* or *species* of the *bee*. Based on these two inputs, the reported result (local variable *th*) is set. Tasks that will not be performed are set to the very high value *NotSetHigh*:

```
to-report ThresholdLevelREP [ thType situation ]
  ;TYPES: eggLaying pollenForaging nectarForaging nursing
  ;SITUATIONS: egg worker youngQueen QueenInitiationPhase QueenSocialPhase Psith

  let th  -1
  ;Egg Laying
  if thType = "eggLaying"
  [
    if situation = "egg"                  [set th NotSetHigh]
    if situation = "worker"               [set th NotSetHigh]
    if situation = "youngQueen"           [set th NotSetHigh]
    if situation = "QueenInitiationPhase" [set th 0.1]
    if situation = "QueenSocialPhase"     [set th 0]
    if situation = "Psith"                [set th 0.2]
  ]

  ; Pollen foraging
  if thType = "pollenForaging"
  [
    if situation = "egg"                  [set th NotSetHigh]
    if situation = "worker"               [set th 0.9]
    if situation = "youngQueen"           [set th NotSetHigh]
    if situation = "QueenInitiationPhase" [set th 0.7]
    if situation = "QueenSocialPhase"     [set th NotSetHigh]
    if situation = "Psith"                [set th NotSetHigh]
  ]

  ; Nectar foraging
  if thType = "nectarForaging"
  [
    if situation = "egg"                  [set th NotSetHigh]
    if situation = "worker"               [set th 0.9]
    if situation = "youngQueen"           [set th NotSetHigh]
    if situation = "QueenInitiationPhase" [set th 0.7]
    if situation = "QueenSocialPhase"     [set th NotSetHigh]
    if situation = "Psith"                [set th NotSetHigh]
  ]

  ; Nursing
  if thType = "nursing"
  [
    if situation = "egg"                  [set th NotSetHigh]
    if situation = "worker"               [set th 0.9]
    if situation = "youngQueen"           [set th NotSetHigh]
    if situation = "QueenInitiationPhase" [set th 0.5]
    if situation = "QueenSocialPhase"     [set th 0.9]
    if situation = "Psith"                [set th NotSetHigh]
  ]
```

```
 if th = -1 [ AssertionProc "Assertion violated in ThresholdLevelREP: TH not set!" ]
 report th
end
```

CropAndPelletSizeREP

Purpose: calculates a *bee's* crop volume and size of its pollen pellets based on the weight of the *bee*.

Asking agents: *bees*

Calling: none

Input: *forage*

Description

As crop volume and size of pollen pellets will change with the size of the *bee*, we calculate *cropvolume_myl* and *pollenPellets_g* from the *bee's weight_mg*. Calculation of the crop volume is based on data from Ings et al. 2006 (described below). We then infer from the crop volume the size of the pollen pellet, using the factor *beehaveCropToPelletFactor_ul-to-g*. This factor is derived from the BEEHAVE model (Becher et al. 2014), dividing the value for pollen pellets weight (0.015g) by the crop volume (50µl) of honeybees.

Calculations of crop and pellet sizes

Fig. 2 of Ings et al. 2006 was re-drawn (using the "Point" tool of the image-processing software ImageJ (https://imagej.nih.gov/ij/)). The values were converted from foraging-load-per-hour to foraging-load-per-trip with the average trip duration (for each series) as presented in their Fig. 1c. Then they were converted from mg to myl using a conversion factor from Schmickl & Crailsheim (2007): *Nectar(myl) = Nectar(mg) * 0.72*
All data series were combined in a plot of foraging load per trip (mg) vs bee weight (g) and a linear trend line was fitted: *foragingLoadNectar/trip(mg) = 402.32* beeWeight(g)*
As we haven't been able to find comparable data for pollen gathering, we assume that the relation of nectar load to pollen load in bumblebees is the same as in honeybees, i.e. crop volume honeybees: 50µl (Nuñez 1966, 1970, Schmid-Hempel et al. 1985), pollen load: 0.015g (Schmickl & Crailsheim 2007 from Seeley 1995).

```
to-report CropAndPelletSizeREP [ forage ]
 ; bee crop and pollen capacity based on weight using (1) linear formula
 ; (2) pollen:crop ratio from HBs in BEEHAVE, with both
 ; having an upper limit set by species-own variables.
  let beeWeightToLoadFactor 0.402 ; 0.402: derived from Ings et al 2006 for nectar loads
  let beehaveCropToPelletFactor_ul-to-g 0.015 / 50  ; nectar load BEEHAVE: 50ul
         ; (Winston (1987), Nuñez (1966, 1970), Schmid-Hempel et al. (1985)
         ; POLLENLOAD  0.015 [g]  (from HoPoMo, Schmickl Crailsheim 2007,
         ; based on Seeley 1995)

  let result 0
  let maxCropVol_myl [ specMax_cropVolume_myl ] of oneSpecies speciesID
  let maxPollen_g  [ specMax_pollenPellets_g ] of oneSpecies speciesID

  if forage = "nectar"
    [ set result min list (maxCropVol_myl) (weight_mg * beeWeightToLoadFactor) ] ; result
                                                       ; is the lower of these two values
  if forage = "pollen"
    [ set result min list (maxPollen_g)  (weight_mg *
          beeWeightToLoadFactor * beehaveCropToPelletFactor_ul-to-g) ]  ; result is the
                                                         ; lower of these two values

  if result = 0 [  AssertionProc "Assertion violated: Error in CropAndPelletSizeREP" ]
```

```
  report result  ; units: ul for nectar, g for pollen!
end
```

CreateSignsProc

Purpose: creates agents used as "signs" on the interface to inform the user about today's foraging conditions or the location of an agent.

Called by: *Setup*

Asking agents: none

Calling: none

Description

Creates a single agent in the shape of "sun", "cloud" and "circletarget". The agents can be shown or hidden, depending on the users choice.

```
 to CreateSignsProc
  create-Signs 1  ; Weather symbol: Sun
  [
    setxy max-pxcor - 6 max-pycor - 16
    set shape "sun"
    set size 11
    set color 44.2
    hide-turtle
    set colonyID -1
  ]
  create-Signs 1  ; Weather symbol: Cloud
  [
    setxy max-pxcor - 10 max-pycor - 17
    set shape "cloud"
    set size 11
    set color grey - 2
    hide-turtle
    set colonyID -1
  ]
  create-Signs 1  ;  Symbol for FIND-Button
  [
    set color red
    set shape "circletarget"
    set size 30
    hide-turtle
  ]
end
```

GO

Purpose: represents all processes of a single time step (day)

Called by: Buttons ("Run", "1 day", "1 week", "1 year", "run X days")

Asking agents: none

Calling:
UpdateMorning_Proc
NeedNectarPollenLarvaeTodayProc

NextActiveBeeREP
ActivityProc
QueensLeavingNestProc
FeedLarvaeProc
QueenProductionDateProc
DevelopmentProc
MortalityBroodProc
BadgersOnTheProwlProc
OutputDailyProc
DrawCohortsProc

Description

If an assertion was violated during the previous time step, the simulation run stops, the modelled world turns red and a message pops up to inform the user.

Then it is made sure that *InspectColony* refers to a *colony* and the Netlogo time step counter tick is increased by 1 (day)

The procedure *UpdateMorning_Proc* is called to updates those variables that count or represent events on a daily or seasonal basis.

For each *colony* its estimated today's need of nectar and pollen for the larvae is calculated in the procedure *NeedNectarPollenLarvaeTodayProc*, which will affect the foraging stimuli in the *colony*.

While the local variable *continueWorking* is true and active (i.e. not "hibernating") workers or queens are present, these active *bees* can perform tasks (specified in the procedure *ActivityProc*). Activities include "nestConstruction", "resting", "egglaying", "nursing", and various activities related to foraging (for nectar or pollen).

```
to GO
 if AssertionViolated = true
    [
      user-message "Assertion violated!"
      ask patches [ set pcolor red ]
      stop
     ]
 ; make sure, InspectColony refers to a colony:
 if (count Bees with [ colonyID = InspectColony ] = 0 and count Colonies > 0)
  [ set InspectColony [ who ] of one-of colonies ]
 if any? turtles with [ who = InspectColony ] and count Colonies > 0
 [
   if ([ breed ] of turtle InspectColony != Colonies)
   [ set InspectColony [ who ] of one-of colonies ]
 ]
let continueWorking true

while [ continueWorking = true ; still some time left today to do some work..
         and count Bees with [ (caste = "worker" or caste = "queen")
        and (activity != "hibernate") and stage = "adult" ] > 0 ; there are actually (active)
      bees, that can work
         and count colonies > 0 ]
  [
    set ActiveBee NextActiveBeeREP
    ask Bee ActiveBee
    [
      ifelse personalTime_s > CallItaDay_s
        [ set continueWorking false ]
        [
```

```
        set Daytime_s personalTime_s ; day time based on personal time of current bee
        ifelse (floor (remainder personalTime_s 3600) / 60) >= 10
        ; adds current personal time to activityList (hh:mm)
          [ set activityList lput ( word floor (personalTime_s / 3600) ":"
            floor ((remainder personalTime_s 3600) / 60)) activityList ]
          [ set activityList lput ( word floor (personalTime_s / 3600) ":0"
            floor ((remainder personalTime_s 3600) / 60)) activityList ]
        ActivityProc
      ]
    if colonyID = -1 [ set personalTime_s CallItaDay_s + 1 ]  ; if queen hasn't founded
                           ; a colony yet, it won't be active for the rest of the day
  ]
]
```

The *bee* addressed is the one with the lowest value for *personalTime_s* (determined in *NextActiveBeeREP*), hence *bees* performing a short task can earlier engage in a new task than *bees* performing a longer task. *PersonalTime_s* of the current bee *also* represents the current time (*Daytime_s*) and is saved in the bees *activityList*, a list that logs all *activities* a *bee* performs during a day.

If *personalTime_s* of all active *bees* exceeds *CallItADay_s* (24 * 3600s =24hrs), *continueWorking* is set false, no more tasks are performed and all bees are "resting" (except of hibernating queens and males).

Then a number of procedures is called and finally the program stops if no *bees* or *colonies* are left:

```
  ask Bees with [ stage = "adult" and activity != "hibernate" and caste != "male" ]
  [
    set activity "resting"
    set activityList lput "End" activityList
  ]
  QueensLeavingNestProc ; young queens leave the nest to mate & hibernate
  FeedLarvaeProc
  QueenProductionDateProc
  DevelopmentProc
  MortalityBroodProc
  BadgersOnTheProwlProc
  if UpdateInterface? [ OutputDailyProc ]
  if ShowCohorts? = true [ DrawCohortsProc ]
  if count Colonies + count Bees = 0 and StopExtinct? = true [ stop ]
end
```

NextActiveBeeREP

Purpose: determines which is the next bee to become active (faster than using Netlogo's *min-one-of* command)

Called by: *Go*

Asking agents: none

Calling: none

<u>Description</u>

The list *ActiveBeesSortedList* (set up in *UpdateMorning_Proc*) contains the ID's (*who*) of all *bees* who can perform a task today. The list is ordered according to the personal time of a *bee* (*personalTime_s*). The first item in this list refers to a *bee* that either just had been active and/or will be active next and its position in the list needs to be updated. The first item of the updated list will then be the next active bee (an alternative approach would be use the NetLogo command *min-one-of* to determine the *bee* with the lowest *personalTime_s*. However, the procedure we describe here runs considerably faster).

ActiveBee is set to this first item of *ActiveBeesSortedList* and the personal time of this "active bee" is then saved in the local variable *persTime_activeBee*. The local variables *minPosition* and *maxPosition* describe the range of positions in *ActiveBeesSortedList* where the active bee should be placed. They are set to the first and last postition of the list to start with and then approach each other until finally the correct position is determined. The local variable *currentPosition* is set to the position just in the middle of the list:

```
to-report NextActiveBeeREP
  ; determines which is the next bee to become active

  set ActiveBee first ActiveBeesSortedList  ; this refers to the bee
    ; that JUST HAD BEEN active! (but not e.g.if the previously active bee just had died!)
  let persTime_activeBee [ personalTime_s ] of bee ActiveBee
  ; the actual position is somewhere between the minimal and the maximal position:
  let minPosition 0 ; counting of items in list start with 0
  let maxPosition length ActiveBeesSortedList - 1
     ; -1, as counting of items in listst start with 0
  let currentPosition round (maxPosition / 2) ; don't know where the final position will be
                                ; so currentPosition  is set right into the middle
```

If *personalTime_s* of the *bee* at *currentPosition* is larger than *persTime_activeBee* then the correct position of the active *bee* will be in the first half of the list, hence *maxPosition* can now be set to *currentPosition* and the new *currentPosition* is then set into the middle of *minPosition* and the previous *currentPosition*. Otherwise (i.e. if *personalTime_s* of the *bee* at *currentPosition* was smaller than *persTime_activeBee*) the correct position of the active bee will be in the second half of the list, hence *minPosition* is set to *currentPosition* and the new current position is set to the middle of the previous *currentPosition* and *maxPosition*. This process is repeated, while the difference between *maxPosition* and *minPosition* is larger 1:

```
  ; now the correct position is determined:
  while [ maxPosition - minPosition > 1 ]
  [
    ifelse [ personalTime_s ] of bee item currentPosition ActiveBeesSortedList
                                                                  > persTime_activeBee
      [
        set maxPosition currentPosition
        set currentPosition round ((currentPosition + minPosition) / 2)
      ]
      [
        set minPosition currentPosition
        set currentPosition round ((maxPosition + currentPosition) / 2)
      ]
  ]
```

The correct position of the active *bee* has now been determined and two sublists of *ActiveBeesSortedList* are created, *beginningList* (from the beginning of *ActiveBeesSortedList* to *currentPosition* (excluding)) and *endList* from *currentPosition* (including) to the end of *ActiveBeesSortedList*:

```
  ; beginningList is activeList to currentposition & endList is currentPosition
  ; to end of list:
  let beginningList sublist ActiveBeesSortedList 0 currentPosition
                                        ; beginning to (excluding) currentPosition
  let endList sublist ActiveBeesSortedList currentPosition length ActiveBeesSortedList
                                                ; from (including) currentPosition to end
```

The active *bee* is now removed from its original position. If more than one *bee* is active (*ActiveBeesSortedList* contains at least two items), the first item of *beginningList* is removed, if only one *bee* is active, the first item of endlist is removed (as *beginningList* is empty in this case):

```
  ;if the number of bees in list is greater than 1, the first bee from the beginningList
; is removed, as this is the activeBee duplicated ; if number of bees in list is 0,
; the first bee from the endList is removed, as this is the activeBee duplicated
  ifelse length ActiveBeesSortedList > 1
   [ set beginningList but-first beginningList ] ; first item of beginningList is removed
   [ set endlist but-first endlist ]        ; first item of endList is removed
```

Finally, *ActiveBeesSortedList* is re-set again by merging *beginningList* and *endList* and *ActiveBee*, which is the ID (*who*) of the active *bee*, is then copied to the correct position, either at the very end or between *beginningList* and *endList* (note that *beginningList* might be empty here so that the previously active *bee* can end up in the first position again). *ActiveBeesSortedList* is now updated and its first item will be reported as the next active *bee*:

```
 ifelse (length endList = 1
          and persTime_activeBee > [ personalTime_s ] of bee item 0 endList )
   [ set ActiveBeesSortedList (sentence beginningList endList ActiveBee) ]
   [ set ActiveBeesSortedList (sentence beginningList ActiveBee endList) ]
  report first ActiveBeesSortedList
end
```

UpdateMorning_Proc

Purpose: updates those variables that count or represent events on a daily or seasonal basis

Called by: *Setup, Go*

Asking agents: none

Calling:

DateREP
Foraging_PeriodREP
UpdateFoodsourcesProc
UpdateSeasonalEventsProc
EmergenceNewQueensProc
UpdateColoniesProc
UpdateColonyStoreBarsProc
CheckNumbersProc

<u>Description</u>

If an assertion (i.e. a statement in the code that needs to be true) is violated, the user message "Assertion violated!" pops up:

```
if AssertionViolated [ ask patches [ set pcolor red ]
                       user-message "Assertion violated!" ]
```

The current day of the year, *Day*, is calculated on the basis of the time step (ticks) and the date is set:

```
set Day round (ticks mod 365.00000001)
set Date DateREP
```

Today's foraging period (reflecting the weather conditions) are calculated:

```
if Day > 0 [ set DailyForagingPeriod_s Foraging_PeriodREP ]
```

Procedures to update *foodsources* and to incorporate seasonal events are called and hibernating queen may emerge:

```
UpdateFoodsourcesProc
UpdateSeasonalEventsProc
EmergenceNewQueensProc
```

PersonalTime_s of not hibernating adult queens and workers is set to *GetUpTime_s* (1s) plus a random timespan (local variable: *randomTimeToGetUp_s*) ranging from 0 - 1800s and the *activityList* of the bees is emptied from the entries of the previous day:

```
 set ActiveBeesSortedList []
 ask Bees with [ (caste = "worker" or caste = "queen")
     and (activity != "hibernate") and stage = "adult" ]
  [
    set personalTime_s GetUpTime_s + random randomTimeToGetUp_s
    set activityList [ ]
    set ActiveBeesSortedList fput who ActiveBeesSortedList
  ]
  set ActiveBeesSortedList sort-by [[personalTime_s] of bee ?1
                            < [personalTime_s] of bee ?2] ActiveBeesSortedList
```

The variable *ActiveBeesSortedList* keeps track of all potentially active adult bees (i.e. workers and not hibernating queens), sorted by their *personalTime_s*. The first *bee* in this list is the next one to become active (see also *NextActiveBeeREP*).

Finally, three more procedures are called (*UpdateColoniesProc*, *UpdateColonyStoreBarsProc* and *CheckNumbersProc*) to update the *colonies* and assert that the total numbers of *bees* is correct.

DateRep

Purpose: calculates the current date

Called by: *UpdateMorning_Proc*

Asking agents: none

Input: none

Description

This procedure determines the date (day, month, year) based on the current time step ("ticks"). The first date after pressing the Setup button (with ticks = 0) is "31 December 0". Leap years are not considered.

```
to-report DateREP
  let month-names (list "January" "February" "March" "April" "May" "June" "July" "August"
                        "September" "October" "November" "December")
  let days-in-months (list 31 28 31 30 31 30 31 31 30 31 30 31)
  let year floor (ticks / 365.01) + 1
  if ticks = 0 [ set year 0 ]
  let month 0
  let dayOfYear remainder ticks 365
  if dayOfYear = 0 [ set dayOfYear 365 ]
  let dayOfMonth 0
  let sumDaysInMonths 0
  while [ sumDaysInMonths < dayOfYear ]
  [
    set month month + 1
    set sumDaysInMonths sumDaysInMonths + item (month - 1) days-in-months
    set dayOfMonth dayOfYear - sumDaysInMonths + item (month - 1) days-in-months
  ]
  let result ""
  if month > 0
    [ set result (word dayOfMonth "  " (item (month - 1) month-names) " " year )]
  report result
end
```

Foraging_PeriodREP

Purpose: calculates today's foraging period

Called by: *UpdateMorning_Proc*

Asking agents: none

Calling: none

Input: none

<u>Description</u>

This reporter procedure determines how much time [s] *bees* can spend on foraging today. The result is based on the "weather" condition, chosen by the user via the Netlogo "chooser" *Weather*. Most options refer to constant daily foraging conditions, e.g. "Constant 8 hrs" results in (up to) 8 hours of foraging every day. The option "foragingHoursExample" provides an arbitrary example of a list with 365 items, defining the hours of foraging for each day of the year. The value for the current *Day* is then picked and reported.

```
to-report Foraging_periodREP
  let foragingPeriod_s -1
  let foragingHoursList [ ]
  let foragingHoursExample [ 0  3.1  0  0  0  1.5  0  0.1  0  0  1.7  1.6  0  0  0  0  0  0
      0 0  1.5  5  0  3.2  0  0  0  0.2  0  0  0  0.1  0.9  5.9  3.5  6.9  1.3  7.7  2.3
      4.6  2.2 0.5 9.2  0  8  3.2 4.1  0  9  9.1  7.3  5.7  4.9  0  12.1  6.5  7.9  7.9
      11.1  2.8  0  2.8  6  5.7  0  4  10.1  2.9  10.1  0  11.4  6.3  9.9  4.4  7.5  8
      12.3  8.7  10.3  3.7  11.3  13.2  14  4.2  7.7  8.2  7.2  9.2  5  13.1  10.5  3.5
      11.1  13.6  6.2  8.4  7.8  8.5  9.8  6.5  4.1  10.8  12.5  15.1  10.1  4.3  7  9.4
      8.9  7.5  7.8  6.6  11.4  12.1  12.4  11.9  10.1  14.7  7.8  13.1  3.3  16.6  14.8
      17.9  5.7  0.2  2.9  10  14.7  16.2  15.8  5.3  5.8  2.5  6  15.2  1.3  13.1  11.2  2
      12.9  9.7  2.1  17.3  5.7  8.5  13.1  18.5  1.7  6.7  13.8  0.5  0.8  15.7  4.9  11.4
      11.9  3.8  11.7  7.1  21.2  17.7  1.8  12.3  15.7  16.9  16.8  9.9  3.6  20.4  13
      5.1  0.6  11.7  2.1  4.7  13.9  13.8  1.4  0.3  18.4  14.8  12.8  3.7  13.5  4.7  0.3
      5.5  4  17.5  1.7  0.3  14.9  12.4  11.6  8.5  4.5  11.1  16  13.2  13.8  0.7  7.1
      14.3  3.4  2.2  5.6  10.6  3.4  15.5  15.6  12.8  15  14  5.9  15.5  9.1  2  1  3.2
      9.3  3  3.1  14  10.2  1  9.7  8.8  3.8  1.9  11.9  9.3  6.5  6.6  8.4  4.3  7.2  1.5
      11.4  10.4  13.5  1.2  6  4.4  13.5  12.4  8  9.3  5.9  0.9  6.8  5.9  9.1  10.5  6
      7.9  2.3  0.8  0  7.9  11  1.3  8.7  6.5  6.6  7.6  0  0  9.4  7.1  6.4  4  6.6  0
```

```
    2.7  0  0  7.8  0  8.7  0.3  2  4.8  1.8  0.9  0  0  7.2  5.8  6.5  0  1.1  0  0  0.7
    6.3  1.3  0  5.5  1.4  2.8  0  0  0  4  0  1.4  5.1  0  0  2.1  0  0.5  0  1  0  0
    2.3  0  0  0  1.4  0.6  0  0  0  0  0.8  0  0  1  0.9  0  0  0  0  0  0  2.3  0  0
    1.9  1.4  0  0  0  1.5  0  0  0  1  1.9  0  0  3.4  0  0  1  0  0  0  0  0  0  1.6 ]

  if Weather = "foragingHoursExample"
   [
    set foragingHoursList foragingHoursExample
    set foragingPeriod_s (item (day - 1) foragingHoursList) * 3600
   ]
  if Weather = "Constant 24 hrs" [ set foragingPeriod_s 24 * 3600 ]
  if Weather = "Constant 20 hrs" [ set foragingPeriod_s 20 * 3600 ]
  if Weather = "Constant 16 hrs" [ set foragingPeriod_s 16 * 3600 ]
  if Weather = "Constant 12 hrs" [ set foragingPeriod_s 12 * 3600 ]
  if Weather = "Constant 11 hrs" [ set foragingPeriod_s 11 * 3600 ]
  if Weather = "Constant 10 hrs" [ set foragingPeriod_s 10 * 3600 ]
  if Weather = "Constant 9 hrs" [ set foragingPeriod_s 9 * 3600 ]
  if Weather = "Constant 8 hrs" [ set foragingPeriod_s 8 * 3600 ]
  if Weather = "Constant 7 hrs" [ set foragingPeriod_s 7 * 3600 ]
  if Weather = "Constant 6 hrs" [ set foragingPeriod_s 6 * 3600 ]
  if Weather = "Constant 5 hrs" [ set foragingPeriod_s 5 * 3600 ]
  if Weather = "Constant 4 hrs" [ set foragingPeriod_s 4 * 3600 ]
  if Weather = "Constant 3 hrs" [ set foragingPeriod_s 3 * 3600 ]
  if Weather = "Constant 2 hrs" [ set foragingPeriod_s 2 * 3600 ]
  if Weather = "Constant 1 hrs" [ set foragingPeriod_s 1 * 3600 ]

  report foragingPeriod_s
end
```

UpdateSeasonalEventsProc

Purpose: addresses seasonal events such as the *species*-specific end of the season

Called by: *Updates_Proc*

Asking agents: none

Calling: *DieProc*

Description

If *Day* equals the *species*-specific *seasonStop* date, for all *bees* of this *species* (except for hibernating queens) the procedure *DieProc* is called in which these bees and their *colonies* are removed. If all queens are in hibernation and no brood is present, any adult males still alive are removed to save computing time, as they won't be able to mate. To prevent the model to run too slowly, on first of January (*Day* = 1) the number of hibernating queens may be reduced to *MaxHibernatingQueens* (input set on interface, default: 10000) or slightly less, depending on whether the queens are from cohort-based colonies or IBM-colonies.

```
to UpdateSeasonalEventsProc
  ask Species
    [
      let whoSpec who
      if Day = seasonStop
      [
        ask Bees with [ speciesID = whoSpec and activity != "hibernate" ]
          [
            let memoNumber number
            if brood?
         [ ask colony colonyID
         [ set broodDeathEndSeason  broodDeathEndSeason + memoNumber ] ]
            DieProc "End of season"
          ]
      ]
    ]

  if TotalHibernatingQueens = TotalQueens and (TotalEggs + TotalLarvae + TotalPupae = 0)
                             and TotalMales > 0   ; i.e. kill males in autumn if
                            ; all queens are in hibernation and no brood is left
```

```
      [
        ask bees with [ caste = "male" ][ DieProc "Males: all queens in hibernation!" ]
      ]

  if Day = 1
      [
        with-local-randomness [ ask bees with [ caste = "queen" and activity = "hibernate" ]
             [ setxy 0 0 ] ]  ; hibernating queens are moved to bottem left corner
             ; to distinguish this year's and last years queens; with-local-randomness: to
             ; not change sequence of random numbers, results of "Version test", 2017-04-21
        let queensToKill TotalHibernatingQueens - MaxHibernatingQueens
        if queensToKill > 0
          [
             set TotalHibernatingQueensEverRemoved TotalHibernatingQueensEverRemoved
                                                + queensToKill
             output-print "Reduced number of hibernating queens to
                                                    no more than MaxHibernatingQueens!" ]
        while [ queensToKill > 0 ]
        [
          ask one-of bees with [ caste = "queen" and activity = "hibernate" ]
             [
               set queensToKill queensToKill - number
               DieProc "max. number of hibernating queens"
             ]
        ]
      ]

  end
```

DieProc

Purpose: calls the "die" command for all biologically relevant agents and keeps track of all their deaths

Called by: *UpdateSeasonalEventsProc*

Asking agents: *Bees* with [*speciesID* = whoSpec and *activity* != "hibernate"]

Calling: none

Input: *causeOfDeath*

<u>Description</u>

If the calling agent is a *bee*, the number of all *bees* ever died (*TotalBeesEverDied*) is increased by the *bees* cohort size, the *bee* is removed from the *ActiveBeesSortedList* (a list that keeps track of all *bees* that can perform a task, ordered by the time they finish their current *activity*) and the *bee* then dies:

```
to DieProc [ causeOfDeath ] ; calls the actual "die" command for all biologically
                            ; relevant agents and keeps track of all their deaths
  if breed = Bees
   [
     set TotalBeesEverDied TotalBeesEverDied + number
     if number < 1
        [ show ticks AssertionProc "Less than 1 bee in bee agent (CheckNumbersProc)" ]

     ; Remove dying bees from the ActiveBeesSortedList:
     if member? who ActiveBeesSortedList
       [ set ActiveBeesSortedList filter [? != who] ActiveBeesSortedList ]

       die
   ]
```

If the calling agent is a *colony*, then some *colony* statistic may be printed in the model's output area and the breed changes from "colonies" to "deadCols", shown as white circles, if the switch *showDeadCols?* is on. This allows to archive *colonies* to later access the stored data:

```
if breed = Colonies
  [
    if eusocialPhaseDate +  switchPointDate + competitionPointDate < NotSetHigh
     [
        output-type "ticks id 1stWorker SP CP QPD death #Q #M: "
        output-type ticks output-type " "
        output-type who output-type " "
        output-type eusocialPhaseDate output-type " "
        output-type switchPointDate output-type " "
        output-type competitionPointDate output-type " "
        output-type queenproductiondate output-type " "
        output-type ticks output-type " "
        output-type totalQueensProduced output-type " "
        output-type totalMalesProduced output-print " "
     ]

    ; instead of removing colony, change breed to deadCol
    ; also kill store bars and change agent into a small white dot on the 2D view
    ask storebars with [ storeColonyID = [who] of myself ] [ die ]
    set breed deadCols
    set size 1
    set label ""
    set color white
    set shape "circle"
    set colonyDeathDay ticks
    if not showDeadCols? [ ht ]

 ]
```

Also *badgers* can die during *Setup*, when they can't find a suitable location for their sett (determined in *CreateBadgersProc*):

```
if breed = badgers
 [
   output-show causeOfDeath
   die
 ]
if breed != deadCols [ AssertionProc "Zombie alarm in DieProc" ]
       ; only dead colonies are supposed to survive DieProc

end
```

UpdateFoodsourcesProc

Purpose: the amounts of available nectar and pollen *foodsources* are re-set

Called by: *Updates_Proc*

Asking agents: none

Calling: none

<u>Description</u>

The total amount of nectar and pollen available today (i.e. before foraging starts) is calculated. All *foodsources* are addressed and their nectar and pollen supply is summed up in the global variables *NectarAvailableTotal_l* and *PollenAvailableTotal_kg*.

If the current day is the first or last day of the flowering period (*startDay*, *stopDay*) of at least one *foodsource*, then the global variable *FoodsourcesInFlowerUpdate?* is set true. (In this case, the colonies will need to update their list of *foodsources* in flower, which happens in the procedure *FoodsourcesInFlowerAndRangeProc* called by *UpdateColoniesProc*. For details, see descriptions of these procedures).

Flower patches do not provide nectar or pollen outside their flowering period. It is assumed that *foodsources* are not in flower at the turn of the year, i.e. *startDay* has to be smaller than *stopDay*, otherwise *AssertionProc* will be called and the program stops.

```
to UpdateFoodsourcesProc
  ; updating FOODSOURCES (nectar & pollen):
  set PollenAvailableTotal_kg 0
  set NectarAvailableTotal_l 0
  set FoodsourcesInFlowerUpdate? false
  ask Foodsources
  [
    if startDay > StopDay
     [ AssertionProc "Foodsource: startDay > StopDay! (UpdateFoodsourcesProc)"  ]
    if day = startDay or day = stopDay [ set FoodsourcesInFlowerUpdate? true ]
    ifelse day >= startDay and day < StopDay
    [
       set nectar_myl nectarMax_myl
       set pollen_g pollenMax_g
    ]
    [
       set nectar_myl 0
       set pollen_g 0
    ]
    set NectarAvailableTotal_l NectarAvailableTotal_l + (nectar_myl / (1000 * 1000))
    set PollenAvailableTotal_kg PollenAvailableTotal_kg + (pollen_g / 1000)
  ]
end
```

EmergenceNewQueensProc

Purpose: new queens may die over winter or otherwise emerge from hibernation and can found a new *colony*

Called by: *UpdateMorning_Proc*

Asking agents: none

Calling:

ThresholdLevelREP
WintermortalityProbREP
DieProc
NestSitesSearchingProc
PsithyrusNestSearchProc
CreateColoniesProc

<u>Description</u>

The task thresholds of *bees* emerging today (i.e. *emergingDate* = *ticks*) are updated, as defined in the reporter-procedure *ThresholdLevelREP*. As queens from a "cohort based" *colony* are still represented as cohorts, they now have to be re-implemented as individuals, before they can found a new *colony*. This is done by creating cohort-size (*number*) - 1 copies of the current *bee* and setting the new cohort-size (*number*) to 1. (Turtles created with the NetLogo command "hatch" inherit all variables from its parent).

```
ask Bees with [emergingDate = ticks]
[

  ; EMERGING
  ...
  ; thresholds are updated:
  set activity "emerging"
  set thEgglaying ThresholdLevelREP "eggLaying" "QueenInitiationPhase"
  set thForagingNectar ThresholdLevelREP "nectarForaging" "QueenInitiationPhase"
  set thForagingPollen ThresholdLevelREP "pollenForaging" "QueenInitiationPhase"
  set thNursing ThresholdLevelREP "nursing" "QueenInitiationPhase"

  ; HATCHING INDIVIDUALS

  ; cohort based queens become individuals:
  let hatchlings number - 1
     ; for cohort based queens: bee needs to be "cloned" cohortsize - 1 times!
  set number 1         ; new queens are individuals now (not cohorts)
  hatch hatchlings
     ; the "clones" of the originally cohort-based queenagent are created
]
```

Then these newly emerged *bees* (with *activity* = "emerging") are addressed to randomly determine whether or not they died over winter. The propability to die is calculated in the reporter-procedure *WintermortalityProbREP*, based on the weight of the queen. (Winter mortality can be switched off on the interface with the switch ("*WinterMortality?*")

```
ask bees with [activity = "emerging"]
[
  ...

  ; WINTER MORTALITY

  if WinterMortality? = true and random-float 1 > WintermortalityProbREP
    [ DieProc "winter mortality"]

  ; AFTER SURVIVAL

  set activity "resting"
  set colonyID -1   ; queens haven't found a nest site yet nor started a colony
  ifelse ShowQueens? = true
    [show-turtle]
    [hide-turtle]
]
```

Those queens that have emerged from hibernation but still are without a nest a nest are then addressed. If no habitat suitable for nesting exists for the queen in the simulated landscape, it dies immediately. Otherwise, the queen can find a nest site, which is determined in the procedures *NestSitesSearchingProc* for social bumblebees and *PsithyrusNestSearchProc* for cuckoo bumblebees (the *activity* of a successful, social queen is then changed to "nestConstruction" in *NestSitesSearchingProc*).
Finally the procedure *CreateColoniesProc* is called if there is at least one bee with the *activity* "nestConstruction".

```
if count bees with [ caste = "queen" and colonyID = -1
              and activity != "hibernate" ] > 0
 [
  ; queens without a colony search for nest sites
  ask bees with [ caste = "queen" and colonyID = -1 and activity != "hibernate" ]
  [
    ifelse count [nestsiteFoodsourceList] of onespecies speciesID > 0
    [
      ifelse speciesName != "Psithyrus"
        [ NestSitesSearchingProc  ]  ; social BB
        [ PsithyrusNestSearchProc ]  ; cuckoo BB
    ]
    [
      DieProc (word "no suitable foodsources for nesting exist for " speciesname)
     ;kill off bees with no chance of finding a nest site
    ]
  ]
```

```
    ; if successful, they build a new nest:
    if count bees with [ activity = "nestConstruction" ] > 0
     [ CreateColoniesProc ]
   ]
```

WintermortalityProbREP

Purpose: determines the probability of a hibernating queen to survive the winter, based on its weight

Called by: *EmergenceNewQueensProc*

Asking agents: *bees* with [*activity* = "emerging"]

Calling: none

Description

The weight of the queen is expressed as a relative weight in comparison to the minimal and maximal queen weights of this *species*. Based on this relative weight (*myRelativeWeight*) the survival probability of the queen is calculated. We derived this equation from Fig. 1B in Beekman et al. (1998) (for details see Chapter 7, Bumblebee biology, life cycle and rationales: Hibernation and winter mortality).

```
to-report WintermortalityProbREP
 let minWeightSpecies_mg [ devWeight_Q_PupationMin_mg ] of oneSpecies speciesID
 let maxWeightSpecies_mg [ devWeight_Q_PupationMax_mg ] of oneSpecies speciesID
 let myRelativeWeight (weight_mg - minWeightSpecies_mg)
                      / (maxWeightSpecies_mg - minWeightSpecies_mg)
 let survivalProb 0.64 / (1 + e ^ (-22 * (myRelativeWeight - 0.32)))
 report survivalProb
end
```

NestSitesSearchingProc

Purpose: determines if a (social) bumblebee queen finds a nest site

Called by: *EmergenceNewQueensProc*

Asking agents: *Bees* (non-Psithyrus queens (not hibernating) without a *colony*)

Calling:

DieProc

NestSiteFoodSourceREP

Description

Based on a species-specific probability (*chanceFindNest*) it is randomly determined whether or not a queen finds a nest site. (Note that this probability is constant and not affected by the area of suitable nesting habitat or density of already established colonies).

If the queen is successful, the *foodsource*, providing suitable nest habitat, is determined in the reporter-procedure *NestSiteFoodSourceREP*. The nest is then build on a randomly chosen grid

cell (NetLogo "patch") within the (theoretical) area of the *foodsource* (food sources in the model are assumed to have a circular shape, with the (theoretical) radius being calculated from the (actual) area of the food source). The *bee* then moves to the location of the nest site and changes its *activity* to "nestConstruction".
Bees that have not found a nest might die during searching. The probability to die is based on the foraging mortality per second (*MortalityForager_per_s*) and the time they spent searching (*NestSearchTime_h*) (both global variables).

```
to NestSitesSearchingProc
  let memoX 0
  let memoY 0
  let memoSpecies    oneSpecies speciesID
  let nestSiteFound  false
  let memoFoodSource nobody
  let dailyChance    [chanceFindNest] of memoSpecies ; chance is species-own variable

 ; Decide if queen finds a nest today
  if random-float 1 <= dailyChance
  [

    ; this food source is found and will be used as nesting habitat:
    set memoFoodSource NestSiteFoodSourceREP memoSpecies

    ; determine loaction of the nest (= a NetLogo patch (gridcell)):
    ask memoFoodSource
    [
      ask one-of patches with
        [ distance myself < ([radius_m] of myself * SCALING_NLpatches/m) ]
      [
        set memoX pxcor
        set memoY pycor
      ]
    ]
    set nestSiteFound true
  ]


  ifelse nestSiteFound = true
  [
    setxy memoX memoY
    set activity "nestConstruction"
  ]
  ;If nest site not found, queen has probability of dying based on foraging mortality
per sec multiplied by seconds searching for nest site
  [
    if random-float 1 <if random-float 1
       < 1 - ((1 - MortalityForager_per_s) ^ (NestSearchTime_h * 60 * 60))
       ; 1 - MortalityForager_per_s: prob. to survive 1s
       ; ^ (NestSearchTime_h * 60 * 60): prob to survive the searching period
       ; 1 - prob. to survive = prob. to die
    [
      DieProc "Queen: died while searching nest site"
    ]
  ]
end
```

NestSiteFoodSourceREP

Purpose: determines which *foodsource* was found by a searching queen to be used as nesting habitat

Called by: *NestSitesSearchingProc*

Asking agents: *Bees* (non-Psithyrus queens (not hibernating) without a *colony*)

Calling: none

Input: *memoSpecies*

Description

The *species*-specific variable *nestsiteFoodsourceList* lists all *foodsources* suitable for nesting for the queen while *nestSiteArea* is the total area of suitable nesting habitat. The probablity for a certain *foodsource* to be chosen for nesting is then the area of this *foodsource* divided by the total area of suitable nesting habitat. This probabilities are summed up (*probsSummedUp*) for all *foodsources* in *nestsiteFoodsourceList* in an ordered way (starting with the first) until a randomly determined threshold probability *p* ([0..1[) is reach. The *foodsource* currently addressed is then chosen for nesting (*chosenFoodSource*) and reported.

```
to-report NestSiteFoodSourceREP [ memoSpecies ]
  let chosenFoodSource  nobody
  let foodSourceList shuffle sort [nestsiteFoodsourceList] of memoSpecies
       ; Randomise order of species-suitable foodSources
  let foodSourceArea    [nestSiteArea] of memoSpecies
       ; total area of species-suitable foodSources
  let p                 random-float 1  ; this is the threshold probability
  let probsSummedUp     0
  let foodCounter       0    ; keeps track of the food source currently addressed
  let fsFound?          FALSE ; so far, no food source (= nesting site) has been found

  while [not fsFound?] ; go through all food sources in the list
  [
   ; the probability of the current food source to be found:
   let probs          [area_sqm / foodSourceArea] of (item foodCounter foodSourceList)
   ; these probabilities are then summed up for each food source in the list..
   set probsSummedUp  probs + probsSummedUp
   ; until the sum is larger then p:
   if probsSummedUp > p
   [
     set chosenFoodSource  (item foodCounter foodSourceList)
       ; current food source is chosen for nesting
     set fsFound?       TRUE   ; yes, suitable food source/nesting site has been found
   ]
   set foodCounter foodCounter + 1
  ]
  ...
  report chosenFoodSource
end
```

PsithyrusNestSearchProc

Purpose: determines if a cuckoo *bee* finds a suitable host *colony* and can enter it successfully

Called by: *EmergenceNewQueensProc*

Asking agents: *Bees* (Psithyrus queens (not hibernating) without a *colony*)

Calling:

DieProc
ThresholdLevelREP

Description

First the probabilities to find a host nest (*findSingleNestProb*), to get access to the nest (*getAccessProb*), to get killed by the host queen (*getKilledProb*) and to kill the host queen (*killQueenProb*) are defined (please note that these suggested probabilities are arbitrary and not yet based on empirical studies).

It is randomly determined if a nest is found with the probability based on the probability to find a single nest (*findSingleNestProb*) and number of nests now available (cuckoo bees in the model can invade any nest, irrespective of the host *species*). It is then randomly determined whether the cuckoo *bee* gets access to the nest (*getAccessProb*) and if it gets killed by the host

queen (*getKilledProb*). Successful cuckoo *bees* then move to a randomly chosen nest and become a member of the *colony* by setting their variable *colonyID* to the ID (*who*) of the host *colony*. They are now shown on the map as a black circle around the infested *colony*. The Psithyrus queens then update their *activity* threshold as defined in *ThresholdLevelREP*. The host queen might then get killed by the cuckoo bee (*killQueenProb*). If both queens survive, they won't fight again.
If the Psithyrus queen is not successful in invading a host nest, it might die during searching, similar as social queens in *NestSitesSearchingProc*.

```
to PsithyrusNestSearchProc
  ;  determines if a cuckoo bee finds a suitable host colony and can enter it successfully
  let memoColID -1
  let findSingleNestProb 0.05
  let getAccessProb 0.25
  let getKilledProb 0.25
  let killQueenProb 0.5
  let succesful false
  let myWho who
  ; probablility to find any host nest:
  let findAnyNestProb 1 - ((1 - findSingleNestProb) ^ count colonies)

  if random-float 1 < findAnyNestProb ; is a host nest found?
  [
    if random-float 1 < getAccessProb ; if yes, does cuckoo bee get access to it?
    [
      ifelse random-float 1 < getKilledProb ; if so, is it killed by host queen?
      [ DieProc "Psithyrus: killed by Bombus queen" ]
      [
        set succesful true
        set color black
        set size size * 8
        set shape "circleSingle"
        ask one-of colonies [ set memoColID who ] ; the host colony is randomly chosen
        set colonyID memoColID ; cuckoo bee becomes a member of the new host colony
        move-to colony colonyID ; cuckoo bee moves to its new host colony
        set thForagingNectar ThresholdLevelREP "nectarForaging" "Psith"
        set thForagingPollen ThresholdLevelREP "pollenForaging" "Psith"
        set thNursing ThresholdLevelREP "nursing" "Psith"
        set thEgglaying ThresholdLevelREP "eggLaying" "Psith"
      ]
    ]
  ]

  ifelse succesful = true ; if cuckoo bee was successful - which colony was invaded?
  [
    if count bees with [colonyID = memoColID and caste = "queen" and mated? = true] > 0
    [
      ask bees with [colonyID = memoColID and caste = "queen" and mated? = true
                    and who != myWho ]    ; host queen might be killed by Psithyrus
      [
        if random-float 1 < killQueenProb
        [
          DieProc "Queen killed by cuckoo bee!"
        ]
      ]
    ]
  ]
  ; if no host nest was invaded, cuckoo bee might die:
  [
   if random-float 1 <
                   1 - ((1 - MortalityForager_per_s) ^ (NestSearchTime_h * 60 * 60))
   [
     DieProc "Psithyrus: died while searching nest"
    ]
  ]
end
```

CreateColoniesProc

Purpose: creates and sets up initial values of a newly founded *colony*

Called by: *EmergenceNewQueensProc*

Asking agents: none

Calling:

PatchesInRangeProc
FoodsourcesInFlowerAndRangeProc

Description

After determining the number of new *colonies* needed (*nNewColonies*) by counting the *bees* with *activity* = "nestConstruction", two *storebars* are created, to display the relative amount of nectar and pollen stored in the the *colony* on the interface:

```
create-storebars 2 * nNewColonies
[
  set shape "halfline"
  set heading 90
  set size 10 * MasterSizeFactor
  set maxSize size
  set storeColonyID whoColony
]
```

Then the *colonies* are created at the current locations of the founding queens. The date of foundation is saved (*colonyFoundationDay*).
Nectar stores are represented by the amount of energy stored (*energyStore_kJ*) which is set to a small inital value, corresponding to 100µl of nectar with a sucrose concentration of 1.5M.

Some variables defining the developmental phase of the *colony* (*switchPointDate, competitionPointDate, eusocialPhaseDate, queenProductionDate*) are set to a very high value (*NotSetHigh* = 9999999999999999). They may be re-set in the further course of the simulation.

The variable *cohortBased?* determines if *colony* members are created in the model as cohorts or as individuals. If the current number of *colonies* that are individual based (*cohortBased?* = false) is smaller than the maximal number of individual based *colonies* allowed in the model (*COLONIES_IBM*), then the new *colony* is implemented as individual based (*cohortBased?* = false), i.e. each individual bumblebee is represented as an agent. Otherwise the *colony* is implemented as cohort based (*cohortBased?* = true), in this case, all bumblebees laid in the same batch are represented by only one *bee* agent.

```
create-Colonies nNewColonies
 [
   set whoColony who ; the ID of the colony
   set colonyFoundationDay ticks
   ask one-of bees with [ activity = "nestConstruction" ]
   [
     set xcol xcor  ;  x and y coordinates of the queen are saved, so that the nest can
be located where the queen is
     set ycol ycor
     set colonyID whoColony ; queen gets the ID of the colony..
     set memoSpeciesID SpeciesID ; and saves her species-type for the colony
     set activity "resting"      ; as the colony is created now, the queen rests
     set speciesShape speciesName   ; saves the species of the queen so that the colony
can be displayed in the according shape
     if ShowQueens? = true [show-turtle]
   ]

   set queenProduction? false   ; no production of queens yet
   set switchPointDate NotSetHigh ; queen won't lay haploid eggs until switchPointDate
is re-se
   set competitionPointDate NotSetHigh
   set eusocialPhaseDate NotSetHigh
   set queenProductionDate NotSetHigh
   set speciesIDcolony memoSpeciesID ; colony gets species-type from queen
```

```
    if ShowNests? = false [ hide-turtle ]
    set xcor xcol      ; the colony is placed at the location of queen
    set ycor ycol
    set queenright? true  ; queen is still alive
    set shape speciesShape ; colony is displayd on the interface as a bumblebee, showing
 the species of the queen
    set heading 0
    set color 33  ; dark brown
    set size ColonySymbolsize
    set energyStore_kJ 100 * EnergySucrose_kJ/mymol * 1.5
     ; i.e. 0.873kJ (= 100 microliter of 1.5M nectar (i.e. ca. 1 crop))
    set colonysize 1  ; i.e. the queen
    set cohortBased? true
    if count Colonies with [ cohortBased? = false ] < COLONIES_IBM
    [
      set cohortBased? false
      set color ColorIBM
      set InspectColony Who
    ]
```

The procedures *PatchesInRangeProc* and *FoodsourcesInFlowerAndRangeProc* are called to determine which *foodsources* are within the foraging range of a *colony* and which of those are currently in flower.

Then the *storeBars* are moved to the location just below their *colonies* and are assigned to either represent pollen stores (orange) or nectar stores (yellow). Finally the global variable *TotalColoniesEverProduced*, keeping track of the number of *colonies* ever produced, is increased by 1.

```
  let barX 3.5
    let barY 5
    ; a nectar and a pollen storebar is now assigned to the new colony
    ask one-of storebars with [ storeColonyID = -1 ]
    [
      ifelse xcol - barX > min-pxcor and ycol - barY > min-pycor
        [ setxy xcol - barX ycol - barY ]
        [ hide-turtle ]
      set storeColonyID whoColony
      set store "Nectar"
      set color yellow
    ]

    set barY barY - 1
    ask one-of storebars with [ storeColonyID = -1 ]
    [
      ifelse xcol - barX > min-pxcor and ycol - barY > min-pycor
        [ setxy xcol - barX ycol - barY ]
        [ hide-turtle ]
      set storeColonyID whoColony
      set store "Pollen"
      set color orange - 0.5
    ]
    set TotalColoniesEverProduced  TotalColoniesEverProduced + 1
  ]
end
```

PatchesInRangeProc

Purpose: creates 2 lists, containing the *who* of all foodsources and masterpatch-foodsources within the foraging range of the *colony*

Called by: *CreateColoniesProc*

Asking agents: (newly created) colonies

Calling: none

Description

All *foodsources* within the foraging range, defined by the global variable *ForagingRangeMax_m* (i.e. independent of the bee *species*), are addressed and their ID (*who*) is saved in the *colonies'* list *allPatchesInRangeList*. All of these *foodsources* for which *masterpatch?* is true, also become member of the *colonies'* list *masterpatchesInRangeList*.

```
to PatchesInRangeProc
 let allPatches []
 let allMasterPatches []
 let xcol xcor
 let ycol ycor
 set allPatchesInRangeList []
 set masterpatchesInRangeList []
; food sources within the colonies' foraging range are addressed..
 ask foodsources with [ distancexy xcol ycol <= (ForagingRangeMax_m
                                                  * Scaling_NLpatches/m )  ]
 [
   set allPatches fput who allPatches
   ; and their ID (who) is saved
   if masterpatch? = true [ set allMasterPatches fput who allMasterPatches ]
   ; they they are masterpatches, their ID (who) is also saved in another list
 ]
 set allPatchesInRangeList allPatches
   ; list of all food sources within the colonies foraging range
 set masterpatchesInRangeList allMasterPatches
  ; list of the masterpatch food sources within the colonies foraging range
end
```

FoodsourcesInFlowerAndRangeProc

Purpose: creates 5 lists, containing the *who* of *foodsources* or masterpatches offering nectar, offering pollen or offering either nectar or pollen within the foraging range

Called by: *CreateColoniesProc*

Asking agents: (newly created) colonies

Calling: none

Description

All *foodsources* within the foraging range of a *colony* are addressed, using the *colonies'* list *allPatchesInRangeList* (created in *FoodsourcesInFlowerAndRangeProc*).
If they contain pollen, their ID (*who*) is added to the *colonies'* list *pollenInFlowerAndRangeList* and their masterpatches are added to *masterpatchesWithPollenlayersInFlowerAndRangeList*.
This is then repeated for *foodsources* offering nectar within foraging to create the lists *nectarInFlowerAndRangeList* and *masterpatchesWithNectarlayersInFlowerAndRangeList*.
The two lists *pollenInFlowerAndRangeList* and *nectarInFlowerAndRangeList* are then combined in the list *allSourcesInFlowerAndRangeList* with duplicates being removed. These lists allow to easily address nectar or pollen patches accessible to a *colony* in the foraging procedures.

```
to FoodsourcesInFlowerAndRangeProc
 ; called by a colony; creates 5 lists, containing the who of foodsources or
 ; masterpatches offering nectar, offering pollen or offering either nectar or pollen
 ; within the foraging range

 set pollenInFlowerAndRangeList []
 set nectarInFlowerAndRangeList []
 set allSourcesInFlowerAndRangeList []
 let pol []
 let polM []
 let nec []
 let necM []


 foreach allPatchesInRangeList ; all patches within foraging range are addressed
 [
   ask foodsource ?
   [
     if pollen_g > 0 ; if they contain pollen..
      [
        set pol lput who pol ; .. their ID is added to the list pol
        set polM lput masterpatchID polM ; .. and their masterpatch is added to the
                                             list polM
      ]
     if nectar_myl > 0  ; similar for nectar
      [
        set nec lput who nec
        set necM lput masterpatchID necM
      ]
    ]
  ]

 set polM remove-duplicates polM ; make sure, a masterpatch occurs only once in the
                                  ; polM list
 set necM remove-duplicates necM ; ditto for necM


 set pollenInFlowerAndRangeList pol
 set masterpatchesWithPollenlayersInFlowerAndRangeList polM
 set nectarInFlowerAndRangeList nec
 set masterpatchesWithNectarlayersInFlowerAndRangeList necM
 set allSourcesInFlowerAndRangeList remove-duplicates
       (sentence pollenInFlowerAndRangeList nectarInFlowerAndRangeList)
; combines the nectar and pollen list into a single list
end
```

UpdateColoniesProc

Purpose: daily update of the colonies' statistics, labels and signs, removal of dead colonies

Called by: *UpdateMorning_Proc*

Asking agents: none

Calling:

FoodsourcesInFlowerAndRangeProc
DieProc
CompetitionPointDateREP

<u>Description</u>

Colonies that run out of energy or are without adult *bees* are removed. The total number of *colonies* and the number of adult males and queens ever produced in a gridcell are saved as patches-own variables (*nColonies*, *nMalesProduced*, *nQueensProduced*).

If *FoodsourcesInFlowerUpdate?* was set true in *UpdateFoodsourcesProc*, then *FoodsourcesInFlowerAndRangeProc* is called to update which *foodsources* in the foraging range of the *colony* are in flower.
All worker *bees* and the old mother queen die after the *colony's* competition day as soon as no more brood is left in the *colony*.
All *colony* members die due to starvation, when its nectar stores (*energyStore_kJ*) are depleted.
If no adult bees are left in the *colony*, the brood dies.

```
to UpdateColoniesProc
 ask colonies
 [
   let whoCol who
   let countBroodMort_NA 0 ; count number of brood dying through no adults left
   let countBRoodMort_ES 0 ; count number of brood dying through energy stores
                           ; being empty

   if FoodsourcesInFlowerUpdate? = true
     [ FoodsourcesInFlowerAndRangeProc ]   ; updated, if some foodsources started
                                           ; or stopped flowering today

   if ticks > competitionPointDate ; death of colony after competition point
      and allEggs + allLarvae + allPupae = 0
      [ ask Bees with [ colonyID = whoCol and adultAge > 10
                 and (caste = "worker"  or caste = "queen")]  ;  as males are outside
                  ; the colony they are killed separately in UpdateSeasonalEventsProc
                 [ DieProc "Colony death after CP!" ] ]

   if energyStore_kJ <= 0 ; death of colony due to starvation
     [
       ask Bees with [ colonyID = whoCol ]
       [
         if brood? = TRUE [set countBRoodMort_ES countBRoodMort_ES + number]
         DieProc "Colony's energy store depleted!"
       ]
     ]

   if (sum [ number ] of Bees with [ colonyID = whoCol and brood? = false ] = 0)
      [ ask Bees with [ colonyID = whoCol ] ; brood dies, if no adults are left
        [
          if brood? = TRUE [set countBroodMort_NA countBroodMort_NA + number]
          DieProc "No adult bees left!"
        ]
      ]
   ; keeping track of dying brood & reason of death:
   set broodDeathsNoAdults        broodDeathsNoAdults + countBroodMort_NA
   set broodDeathsEnergyStores    broodDeathsEnergyStores + countBRoodMort_ES
   set summedIncubationToday_kJ   0 ; so far, no incubation has taken place today
```

Additionally, some *colony* statistics are updated, to keep track of the number of *bees* in different developmental *stages* (and similar for larvae, pupae, adults, workers, adult queens (hibernating or active), active adult queens (i.e. not in hibernation), adult males and the total *colony* size (i.e. all *Bees* of the *Colony*, including brood (but not offspring queens or males that already have left the *colony*):

```
set allEggs sum [ number ] of Bees with [ colonyID = whoCol and stage = "egg" ]
set allLarvae sum [ number ] of Bees with [ colonyID = whoCol and stage = "larva" ]
set allPupae sum [ number ] of Bees with [ colonyID = whoCol and stage = "pupa" ]
set allAdults sum [ number ] of Bees with [ colonyID = whoCol and stage = "adult" ]
set allAdultWorkers sum [ number ] of Bees
              with [ colonyID = whoCol and caste = "worker" and stage = "adult" ]
set allAdultQueens sum [ number ] of Bees
              with [ colonyID = whoCol and caste = "queen" and brood? = false ]
set allAdultActiveQueens sum [ number ] of Bees
              with [ colonyID = whoCol and caste = "queen" and activity != "hibernate"
                                         and brood? = false ]
set allAdultMales sum [ number ] of Bees
              with [ colonyID = whoCol and caste = "male" and brood? = false ]
set colonysize sum [ number ] of Bees with [ colonyID = whoCol ]
ifelse allAdultWorkers > 0 ; calculate larvaWorkerRatio if adult workers are present
     [ set larvaWorkerRatio allLarvae / allAdultWorkers ]
     [ set larvaWorkerRatio NotSetHigh ]
set colonyWeight_mg sum [number * weight_mg] of Bees with [ colonyID = whoCol ]
```

The queen switches to lay haploid instead of diploid eggs on the day of the *colonies'* switch point (Duchateau & Velthuis 1988). *Colonies* may switch with a daily probability (*DailySwitchProbability*) if they are in the eusocial phase (i.e. worker bees are or were present) and the ratio of larvae:worker *bees* is below a certain threshold (*LarvaWorkerRatioTH*):

```
if switchPointDate = NotSetHigh  ; i.e. the colony/queen hasn't switched to lay
                                 ; haploid eggs
 [
  if eusocialPhaseDate < NotSetHigh  ;.. but adult workers are (or were) present
  and larvaWorkerRatio < LarvaWorkerRatioTH ;.. and enough worker bees are present
                                            ; rel. to larvae
  [

    if random-float 1 <= DailySwitchProbability ; then the colony may switch to
    [ set switchPointDate ticks ]  ; the produce haploid eggs
  ]
 ]
```

The *colonies'* competition point (when worker start to lay eggs themselves but also eat eggs produced by nestmates resulting in very high brood mortality) is determined in the reporter-procedure *CompetitionPointDateREP*, if 1.) it wasn't determined already, 2.) the *colony* is in the eusocial phase and 3.) the queen production day (i.e. when diploid larvae are raised as queens) is already set. *Colonies* beyond the competition point are shown topsy-turvy:

```
if competitionPointDate = NotSetHigh ; i.e. if CP is not determined yet..
    and eusocialPhaseDate    < NotSetHigh ; but colony is in eusoc. phase
    and queenProductionDate  < NotSetHigh ; and queen product. day is defined
  [ set competitionPointDate CompetitionPointDateREP ]
if ticks >= competitionPointDate [ set heading 180 ]
   ; colony symbol is turned on its head after CP
```

Finally the *colony signs* and labels, providing information on the interface on size and state of the *colony*, are updated. Before *colonies* without any *bees* are removed (*die*), the grid cell (NetLogo "patch") they are located updates its records on number of males (*nMalesProduced*), queens (*nQueensProduced*) and *colonies* (*nColonies*) ever produced at this location. If the switch *KeepDeadColonies?* is set false (default: true), then *DeadCols* are removed from the simulation. (This has no effect on the model outcome - except of changing the series of pseudo-random numbers - nor does it strongly affect the computation time).

```
; LABELS & SIGNS:
    set label colonysize
    if count Bees with [ colonyID = whoCol and caste = "queen" and mated? = true ] = 0
      [ set queenright? false ]
    ifelse count Bees with [ colonyID = whoCol ] = 0
      [
        ; to diplay the production of reproductives on the map..
        let malesHere totalMalesProduced   ; ... the numer of adult males..
        let queensHere totalQueensProduced  ; .. and adult queens ever produced by
                                           ; this dying colony..
        ask patch-here ; (info saved in NetLogo patch = grid cell)
         [
          set nMalesProduced nMalesProduced + malesHere     ; .. is added to the total
                                                          ; number of males..
          set nQueensProduced nQueensProduced + queensHere  ; and queens ever produced
                                                          ; here at this Netlogo patch
          set nColonies nColonies + 1      ; ..and the total colonies here
         ]
        let EndSeasonDate [seasonStop] of onespecies speciesIDcolony
        ifelse day >= EndSeasonDate
          [ set ColonyDeathsEndSeason  ColonyDeathsEndSeason + 1 ]
          [ set ColonyDeathsNoBees     ColonyDeathsNoBees + 1 ]

        DieProc "Colony: No adults or brood left in this colony!"
       ; colony dies, as no bees are left
      ]
      [
        set colonyAge colonyAge + 1 ; surviving colonies age by 1 day
      ]
```

```
  ]
  if KeepDeadColonies? = false and Day = 1 [ ask DeadCols [ die ] ]
       ; dead colonies can be removed from the simulation with the new year
end
```

CompetitionPointDateREP

Purpose: determines the date of a *colonies'* competition point

Called by: *UpdateColoniesProc*

Asking agents: *colonies*

Calling: none

Description

The competition point is calculated, following Duchateau & Velthuis 1988 with the equation derived from their Fig. 6. It is driven by the production of queens and happens at latest 45 days after emergence of the first worker bees.

```
to-report CompetitionPointDateREP
  ; determines the date of a colonies' competition point
 let compDate NotSetHigh
 let x queenProductionDate - eusocialPhaseDate
 let y 0.7 * x + 15.5   ; from Duchateau & Velthuis 1988, Fig. 6
 let latestCPafter_d 45

 set compDate round (eusocialPhaseDate + y)
 if compDate - eusocialPhaseDate > latestCPafter_d
    [ set compDate eusocialPhaseDate + latestCPafter_d ]

 report compDate
end
```

UpdateColonyStoreBarsProc

Purpose: daily update of the *colonies'* nectar and pollen store display

Called by: *UpdateMorning_Proc*

Asking agents: none

Calling: none

Description

The *storeBars* are located on the interface underneath the *colony* they belong to and their length (*size*) represents the relative amount of nectar and pollen stored in comparison to a theoretical ideal store. A size factor is calculated as current amount of food stored divided by an "ideal" amount of food stored:

```
to UpdateColonyStoreBarsProc
```

```
 ask storeBars
  [
    let nectarSizeFactor 0
    let pollenSizeFactor 0
    ifelse colony storeColonyID = nobody
     [ die ]  ; storeBars die here (and not in DieProc as not a biological agent)
     [
       ask colony storeColonyID
       [
         set nectarSizeFactor energyStore_kJ / (idealEnergyStore_kJ + 0.00001)
               ; + 0.00001 to avoid division by zero
         set pollenSizeFactor pollenStore_g / (idealPollenStore_g + 0.00001)
         if nectarSizeFactor > 1 [ set nectarSizeFactor 1 ]
         if pollenSizeFactor > 1 [ set pollenSizeFactor 1 ]
       ]
     ]
    if store = "Nectar"
     [ set size maxSize * nectarSizeFactor ]
    if store = "Pollen"
     [ set size maxSize * pollenSizeFactor ]
  ]
end
```

The "ideal" values are calculated in the reporter procedures *StimForagingNectarREP* and *StimForagingPollenREP* and are based on the amount of food that is approximately required within a certain number of days.

NeedNectarPollenLarvaeTodayProc

Purpose: calculates for each *colony* how much nectar and pollen is required today to feed the larvae

Called by: *Go*

Asking agents: none

Calling: *MaxWeightGainToday_mg_REP*

<u>Description</u>

All *bees* with *stage* = "larva" in each *colony* are addressed. Their need for pollen is calculated from the maximal weight they can gain today (determined in the reporter-procedure *MaxWeightGainToday_mg_REP*) times a *pollenToBodymassFactor* factor times the cohort size (*number*). This is summed up in the local variable *pollenNeedMyColony_g* over all larvae cohorts of the *colony* and saved in the *colony* variable *pollenNeedLarvaeToday_g*. The *colonies'* energy needs for the larvae (*energyNeedToday_kJ*) are then the product of the pollen needs and the global variable *EnergyRequiredForPollenAssimilation_kJ_per_g*.

```
to NeedNectarPollenLarvaeTodayProc ; calculates how much nectar and pollen is
                                ; approximately required today to feed the larvae
 ask colonies
 [
  let myColony who
  let pollenNeedMyColony_g 0 ; no pollen need so far

  ; address the larvae of this colony:
  ask bees with [ stage = "larva" and colonyID = myColony ]
    [ set pollenNeedMyColony_g pollenNeedMyColony_g ; pollen need summed up here..
                              + number             ; calculated from cohort size ..
                              * ((MaxWeightGainToday_mg_REP who)
                                    ; times max. possible gain in weight..
```

```
                              / ([pollenToBodymassFactor] of OneSpecies speciesID))
                                    ;..translated into pollen
                              / 1000 ]      ; units: mg -> g
  set pollenNeedLarvaeToday_g pollenNeedMyColony_g
  set energyNeedToday_kJ
           pollenNeedLarvaeToday_g * EnergyRequiredForPollenAssimilation_kJ_per_g
 ]
end
```

MaxWeightGainToday_mg_REP

Purpose: calculates a larva's maximal weight gain during 24 hrs

Called by: *FeedLarvaeProc*, *NeedNectarPollenLarvaeTodayProc*

Asking agents: *Bees* (larva)

Calling: none

Input: *myID*

Description

The weight gain of a single larva is calculated by multiplying its current weight with a *species*-specific weight gain factor minus the current weight. Larvae reaching a *caste* specific maximal weight stop growing:

```
to-report MaxWeightGainToday_mg_REP [ myID ]
 let maxWeightGain_mg 0 ; actual max. weight gain not determined yet
 let memoQPupationMax [ devWeight_Q_PupationMax_mg ] of OneSpecies speciesID
  ; the max. pupal weight of a queen
 let memoWPupationMax [ devWeightPupationMax_mg ] of OneSpecies speciesID
   ; the max. pupal weight of a worker
 ask bee myID
 [
  let myGrowthFactor [ growthFactor ] of OneSpecies speciesID
         ; growth factor depends on the species
  set maxWeightGain_mg (weight_mg * myGrowthFactor) - weight_mg
         ; i.e. a larva's max. weight gain today

    ; if the maximum new weight is greater than the pupation max, reduce maxWeightGain
   ; to difference between pupation max and current weight:
  if caste = "queen" and weight_mg + maxWeightGain_mg > memoQPupationMax
      [ set maxWeightGain_mg   memoQPupationMax - weight_mg ]  ; for queens

  ; .. and for worker (or still undefined) larvae:
  if (caste = "worker" or caste = "undefined" or caste = "male")
         and weight_mg + maxWeightGain_mg > memoWPupationMax
      [ set maxWeightGain_mg   memoWPupationMax - weight_mg ]
 ]
 report maxWeightGain_mg
end
```

ActivityProc

Purpose: determines the *activity* (resting, egg laying, nursing, nectar or pollen foraging) of a worker or queen bee

Called by: *Go*

Asking agents: *bees* (workers and non-hibernating queens)

Calling:

StimEgglayingREP
StimForagingNectarREP
StimForagingPollenREP
StimNursingREP
EgglayingProc
BroodIncubationProc
ForagingProc

Description

In this procedure, the *bees* repeatedly decide on the tasks they perform during the day. The main *activities* are "egglaying", "nursing", "nectarForaging" and "pollenForaging". Decisions are made via a stimulus-threshold approach in combination with ranking the task according to their importance for the *colony*. The *activity* of *bees* not engaging in a task is set to "resting". There are some more activities, which are not part of this procedure like "hibernate" (set in *QueensLeavingNestProc*) when young queens overwinter, "nestConstruction" (set in *NestSitesSearchingProc*) when freshly emerged queens have found a nesting site and are about to create a new *colony* and a number of foraging activities, defining the sub-tasks (like "searching", "collectNectar", "bringingNectar" etc

At the beginning, the *activity* of a *bee* is set to "resting". Then the *bees*' *colony* is addressed to determine the stimuli in this *colony* for egg laying, nectar foraging, pollen foraging and nursing (each calculated in a reporter procedure: *StimEgglayingREP*, *StimForagingNectarREP*, *StimForagingPollenREP*, *StimNursingREP*).

```
to ActivityProc
  let break_s 0.5 * 3600 ; time a bee spents resting before potentially becoming
                         ; active again
  set activity "resting"
  if colonyID >= 0  ; only colony members can angage in tasks
  [
   ask Colony colonyID  ; the stimuli in a bees' colony are determined
     [
       set stimEgglaying StimEgglayingREP
       set stimNectarForaging StimForagingNectarREP
       set stimPollenForaging StimForagingPollenREP
       set stimNursing StimNursingREP
     ]
```

If a stimulus is higher than the *bees*' threshold for this task, her *activity* is set to this task. The order in which stimuli and threshold are compared is based on the rank of the task. Tasks are ranked from lowest to highest priority, starting with the ones that have the least impact on the *colony*. *Activity* can be re-set immediately afterwards to another task if the stimulus is higher than the threshold and therefore tasks of lower rank will only be performed when the thresholds for the higher priority tasks are not met.

Order:
1. egg laying (eggs are less valuable than larvae or pupae)
2. nursing (lack of nursing will delay development but not quickly kill brood)
3. pollen foraging (lack of pollen would kill larvae)
4. nectar foraging (depleted nectar stores would kill the whole *colony*)

```
; if a colony-specific stimulus exceeds the individual threshold,
; "activity" of the bee is set to this particular task,
; tasks are ordered by their importance:
if [ stimEgglaying ] of Colony colonyID > thEgglaying [ set activity "egglaying" ]
if [ stimNursing ] of Colony colonyID > thNursing [ set activity "nursing" ]
if [ stimPollenForaging ] of Colony colonyID > thForagingPollen
                                          [ set activity "pollenForaging" ]
if [ stimNectarForaging ] of Colony colonyID > thForagingNectar
                                          [ set activity "nectarForaging" ]
```

As an example, if the stimuli for nursing and nectar foraging both exceeded the *bees'* thresholds, than her *activity* would be "nectarForaging".

The *activity* of a *bee* is then written in her *activityList* ("REST", "EGG", "NURSE", "P-FOR", "N-FOR") and the procedure for this task (*EgglayingProc*, *BroodIncubationProc*, or *ForagingProc* (for nectar and pollen) is called. Note that cuckoo *bees* neither engage in foraging nor in brood care:

```
  if speciesName = "Psithyrus" and (activity = "nursing" or activity = "pollenForaging"
                              or  activity = "nectarForaging")
     [ set activity "resting" ]  ; cuckoo bees don't work!

  if activity = "resting" [ set activityList lput "REST" activityList
                            set personalTime_s personalTime_s + break_s
                          ]
  if activity = "egglaying" [ set activityList lput "EGG" activityList
                              EgglayingProc
                            ]
  if activity = "nursing" [ set activityList lput "NURSE" activityList
                            BroodIncubationProc
                          ]
  if activity = "pollenForaging"
                          [ set activityList lput "P-FOR" activityList
                            ForagingProc
                          ]
  if activity = "nectarForaging"
                          [ set activityList lput "N-FOR" activityList
                            ForagingProc
                          ]
end
```

If none the stimuli exceeded the *bee's* threshold, then the *activity* remains "resting", no task related procedure is called and the local variable *break_s* is added to the *bees*' *personalTime_s*, reflecting the time a *bee* is resting.

StimEgglayingREP

Purpose: calculates the stimulus for egg laying within a *colony*

Called by: *ActivityProc*

Asking agents: *Colonies*

Input: none

<u>Description</u>
The stimulus for egg laying is set to 0, but can be changed to 1, if either enough pollen is present but no eggs or larvae or if at least one worker *bee* is present:

```
to-report StimEgglayingREP
  let egglayingStim 0  ; egg stimulus is 0, unless..
  ; ..pollen stores are ok and no eggs or larvae are present..
```

```
    if ((pollenStore_g > [ minPollenStore_g ] of oneSpecies speciesIDcolony
         and (allEggs + allLarvae) = 0))
       or allAdults > 1 ; ..or if at least 1 worker is present
     [ set egglayingStim  1 ] ; ..then egg stimulus is 1
    report egglayingStim
  end
```

StimForagingNectarREP

Purpose: calculates the stimulus for nectar foraging within a *colony*

Called by: *ActivityProc*

Asking agents: *Colonies*

Input: none

<u>Description</u>

The stimulus to forage nectar (*nectarStim*) results from a comparison of the actual energy store with an "ideal" energy store (*idealEnergyStore_kJ*) which takes the estimated energy consumption of larvae (*energyNeedToday_kJ*), a number of days the stores are supposed to last (*storeSize_d*), a minimal energy store (*minNectarStore_kJ*) and a correction factor (*idealEnergyFactor*) into account. Depending on whether or not the stimulus exceeds *nectarStimTH*, it is then either set to 1 or 0.
As there is little understanding about the decision making of real bees, the procedures to determine stimuli and thresholds do not actually reflect biological processes but instead are the result of a heuristic approach, testing the model with various versions and parameterisations to maximise the population size. The chosen implementation leads to reasonable *colony* growth and population dynamics.

```
  to-report StimForagingNectarREP ; asked by colony
    let storeSize_d 5
    let minNectarStore_kJ 20
    let nectarStimTH  0.005 ; heuristically determined
    let idealEnergyFactor 6 EnergyFactorOnFlower

    set idealEnergyStore_kJ IdealEnergyFactor * energyNeedToday_kJ
                                               * storeSize_d + minNectarStore_kJ
    let nectarStim (idealEnergyStore_kJ - energyStore_kJ) / idealEnergyStore_kJ

  ; if the stimulus is low, it is set to 0, otherwise to 1:
    ifelse nectarStim > NectarStimTH
      [ set nectarStim 1 ]
      [ set nectarStim 0 ]

    ; foraging only during daytime:
    if (Daytime_s < Sunrise_s)
        or (Daytime_s > Sunrise_s + DailyForagingPeriod_s)
      [ set nectarStim 0 ]
    report nectarStim
   end
```

StimForagingPollenREP

Purpose: calculates the stimulus for pollen foraging within a *colony*

Called by: *ActivityProc*

Asking agents: *Colonies*

Input: none

Description

The stimulus to forage pollen (*pollenStim*) results from a comparison of the actual pollen store with an "ideal" pollen store (*idealPollenStore_g*) which takes the estimated pollen consumption of larvae (*pollenNeedLarvaeToday_g*), a number of days the stores are supposed to last (*storeSize_d*) and a minimal pollen store (*minPollenStore_g*) into account. Depending on whether or not the stimulus exceeds *pollenStimTH*, it is then either set to 1 or 0.

```
to-report StimForagingPollenREP ; asked by colony
  let storeSize_d 5
  let pollenStimTH  0.005 ; heuristically determined
  set idealPollenStore_g pollenNeedLarvaeToday_g * storeSize_d
               + [ minPollenStore_g ] of oneSpecies speciesIDcolony
  if idealPollenStore_g < 0
       [ AssertionProc "Negative idealPollenStore_kJ! (StimForagingPollenREP)" ]
  let pollenStim (idealPollenStore_g - pollenStore_g) / idealPollenStore_g
  ifelse pollenStim > pollenStimTH
    [ set pollenStim 1 ]
    [ set pollenStim 0 ]
  if (Daytime_s < Sunrise_s) or (Daytime_s > Sunrise_s + DailyForagingPeriod_s)
                               ; foraging only during daytime
    [ set pollenStim 0 ]
  report pollenStim
 end
```

StimNursingREP

Purpose: calculates the stimulus for nursing within a *colony*

Called by: *ActivityProc*

Asking agents: *Colonies*

Input: none

Description

If the energy required for incubation today (*incubationRequiredToday_kJ*), which is the average energy required per day for an individual (*devQuotaIncubationToday_kJ*) times the brood (*allEggs* + *allLarvae* + *allPupae*) is larger than the incubation actually received today (*summedIncubationToday_kJ*), then the stimulus to nurse (*nursingStim*) is set to 1 or otherwise to 0.

```
to-report StimNursingREP
 let nursingStim 0
 let incubationRequiredToday_kJ [ devQuotaIncubationToday_kJ ]
                                 of Onespecies speciesIDcolony
       * (allEggs + allLarvae + allPupae) ; approx. incubation required for
                                          ; whole brood nest today
 set nursingStim 0
 if incubationRequiredToday_kJ > summedIncubationToday_kJ [ set nursingStim 1 ] ; bees
       ; will try to incubate brood nest, until requirements for today are fulfilled
 report nursingStim
end
```

EgglayingProc

Purpose: creates a batch of new eggs

Called by: *ActivityProc*

Asking agents: *Bees* (workers and non-hibernating queens)

Calling: *EggsParameterSettingProc*

Description

After defining some local variables, the number of cohorts (local variable *newCohorts* representing the new eggs) and their cohort size is determined. If the *colony* variable *cohortBased?* is true, then only one cohort is produced and the number of *bees* in this cohort (*beesInCohort*) is set to *batchsize* of the *species*. If *cohortBased?* is false (i.e. it is an individual-based *colony*), then the number of cohorts is set to *batchsize* of the *species* and *beesInCohort* is 1.

```
to EgglayingProc
  let mother caste   ; to distinguish queen and worker laid eggs
  let newCohorts 1   ; for cohort based cols (will later be changed in case of IBM cols)
  let beesInCohort [ batchsize ] of OneSpecies speciesID  ; for cohort based cols (will
                                                         ; later be changed in case of IBM cols)
  let eggWeight [devWeightEgg_mg] of OneSpecies speciesID
  let pollenToMass [pollenToBodymassFactor] of OneSpecies speciesID

  if [ cohortBased? ] of colony colonyID = false   ; i.e. IBM colonies..
    [
      set newCohorts [ batchsize ] of OneSpecies speciesID  ; .. number of "cohorts" =
        ; batchSize, as each "cohort" contains only a single bee (as IBM colony)..
      set beesInCohort 1   ; .. with only 1 bee in each
    ]
```

If enough energy and pollen is stored in the colony, new *bees* (N = *newCohorts*) are created, using the NetLogo *hatch* command, where the new agent inherits all variables from its "parent" (in this case the egg laying *bee*).

If the mother is the queen, then the eggs can be haploid or diploid. If the *colony* has reached the switching point to produce reproductives then queen laid eggs are haploid, and will develop into males otherwise her eggs are diploid. Diploid *bees* may develop into workers or queens, depending on their individual development and the state of the *colony*. However, if they are homozygous and *SexLocus?* is true, then they become males (see *Development_LarvaeProc* and *DetermineCaste_REP*). Worker laid eggs are always haploid. Then the procedure *EggsParameterSettingProc* is called, which re-sets the *bees*' variables:

```
if [ pollenStore_g ] of colony colonyID > pollenCost_g and
    [ energyStore_kJ ] of colony colonyID > energyCost_kJ
[   ; eggs can only be laid, if enough energy and pollen is present!

  hatch newCohorts ; "hatch" command, as "create" is not possible in a turtle context
  [
    ifelse mother = "queen"
      [ ; queens can produce male and female offspring:
        ifelse ticks > [ switchPointDate ] of colony colonyID
               ; after the switch point, only males are produced
          [ set ploidy 1 ]    ; 1: haploid male
          [ set ploidy 2 ]    ; 2: diploid bee (worker, queen or diploid male)
      ]
      [ ; workers can only produce male offspring:
        set ploidy 1          ; 1: haploid male
      ]
    EggsParameterSettingProc beesInCohort ; calls the procedure
```

```
                 ; EggsParameterSettingProc and transfers the local variable beesInCohort
    ]
```

Finally, costs in terms of energy and pollen consumption are calculated and the queen's *personalTime_s* is updated:

```
    ; Pollen cost is total mass of laid eggs * the conversion of pollen to bee body mass
    ; Energy cost is amount needed by female to facilitate replacement lost pollen
    let pollenCost_g   beesInCohort * eggWeight * pollenToMass / 1000
    let energyCost_kJ  pollenCost_g * EnergyRequiredForPollenAssimilation_kJ_per_g

    ; Remove costs from store
    ask colony colonyID
    [
     set pollenStore_g  pollenStore_g - pollenCost_g
     set energyStore_kJ energyStore_kJ - energyCost_kJ
    ]
  ]
  set personalTime_s personalTime_s + 24 * 3600  ; i.e. no further
                 ; action on that day, personalTime_s will be reset on next morning!

end
```

EggsParameterSettingProc

Purpose: sets parameter values for new eggs

Called by: *EgglayingProc*

Input: *beesInCohort* (i.e. *batchsize* of the addressed bumblebee *species*)

Asking agents: *Bees* (workers and non-hibernating queens)

Calling: *ThresholdLevelREP*

Description

Those parameters that are different in an egg from its adult mother are reset here (remember that eggs, created with the "hatch" command, inherited all parameter values of their mother). The *allelesList*, which represents a *bee's* genome, receives randomly one (of the two) alleles of its mother's *allelesList*. Diploid eggs, laid by a mated queen, also receive the (single) allele present in the *spermathecaList* of their mother. Thresholds for nectar and pollen foraging, egg laying and nursing are set to values defined in *ThresholdLevelREP*.

```
to EggsParameterSettingProc [ beesInCohort ]
  ; sets parameter values for new eggs

  ; alleles of the egg:
  let shiftDrawnCohorts 1  ; (1) to show cohorts above the colony
  let myAllelesList []
  set myAllelesList fput one-of allelesList myAllelesList   ; egg gets one allele from
                                                            ; its mother
  set allelesList myAllelesList
  set caste "undefined"   ; castes: "undefined", "queen", "worker", "male"
  ifelse ploidy = 1  ; haploid males
    [
      set color violet
      set caste "male"
    ]
    [                     ; females and diploid males:
      ...
      set color blue
```

```
      set allelesList fput one-of spermathecaList allelesList  ; diploid bees get
                                              ; another allele from their father/spermatheca
      if  SexLocus? = true  ; if alleles refer to the sex locus..
      and item 0 allelesList = item 1 allelesList  ; .. and bee is homozygous..
      [ set caste "male" ]   ; .. it becomes a diploid male!
    ]
  set spermathecaList [] ; eggs haven't mated yet..
  set size CohortSymbolSize
  set shape "halfline"
  __set-line-thickness 0.5
  set heading 0
  set number beesInCohort
  set TotalBeesEverProduced TotalBeesEverProduced + number
  set activity "resting"
  set adultAge 0
  set brood? true
  set broodAge          0   ; set to 0 as eggs are created with the "hatch" command
  set cumulTimeEgg_d    0   ; Set to 0, not mother's value
  set cumulTimeLarva_d  0   ; Set to 0, not mother's value
  set cumulTimePupa_d   0   ; Set to 0, not mother's value
  set cropVolume_myl    0   ; now based on weight, has to be set on emergence
  set pollenPellets_g   0   ; now based on weight, has to be set on emergence
  set currentFoodsource -1  ; not set yet
  set nectarsourceToGoTo -1 ; not set yet
  set pollensourceToGoTo -1 ; not set yet
  set stage "egg"   ; egg, larva, pupa, adult
  set mated? false
  set thEgglaying       ThresholdLevelREP "eggLaying" "egg"
  set thForagingNectar  ThresholdLevelREP "nectarForaging" "egg"
  set thForagingPollen  ThresholdLevelREP "pollenForaging" "egg"
  set thNursing         ThresholdLevelREP "nursing" "egg"
  set activityList [ ]
  set knownMasterpatchesNectarList [ ]
  set knownMasterpatchesPollenList [ ]
  set weight_mg [ devWeightEgg_mg ] of OneSpecies speciesID
  ;;; set colonyID ...                  no need to re-set!
  set cumulIncubationReceived_kJ 0
  set emergingDate NotSetLow
  set expectation_NectarTrip_s 0
  set expectation_PollenTrip_s 0
  set glossaLength_mm 0
  ;;; set mtDNA ...                     no need to re-set!
  set nectarLoadSquadron_kJ 0
  set personalTime_s 0
  set pollenForager? false
  set pollenLoadSquadron_g 0
  ;;; set speciesID  ...               no need to re-set!
  ;;; set speciesName ...               no need to re-set!

  ; location of egg cohort on the interface is relative to its colony's location:
  set xcor [ xcor ] of Colony colonyID
      - [ devAgeEmergingMin_d / 10 ] of OneSpecies speciesID
  set ycor [ ycor ] of Colony colonyID + shiftDrawnCohorts
  ifelse ShowCohorts? = false
    [ hide-turtle ]
    [ show-turtle ]
  ask colony colonyID [set totalEggsProduced  totalEggsProduced + beesInCohort]

end
```

BroodIncubationProc

Purpose: determines how much energy for brood incubation is provided by a "nursing" *bee*

Called by: *ActivityProc*

Asking agents: *bees* (workers and non-hibernating queens)

Calling: none

<u>Calculating incubation:</u>
We derived the duration of a single heating activity from Heinrich (1979). His Fig. 5.2 is showing 144 temperature recordings (each lasting 10 minutes) of a single, brood incubating queen (*B. vosnesenskii*) over 24 hrs. We identified 20 heating periods covered by ca. 96

recordings. Hence, the total total time spent on incubation was 96 * 10 min. = 960 minutes per day or 960 * 60 / 20 = 2880s of incubation per heating period. Silvola (1984) suggests a *B. terrestris* queen spends about 10 kJ/day on incubation. Assuming the mean weight of a *B. terrestris* queen is 0.8g (Beekman et al. 1998) then the energy provided from heating bees is 10kJ / 0.8g = 12.5 kJ/g. Assuming 960 minutes incubation per day, then 2.17 * 10^{-7} kJ of heat can be produced by 1 mg heating *bees* per second.

Description

Based on the mass (*weight_mg*) of the incubating *bee* (cohort) and the time spent on incubation (*heatingPeriod_s*), it is calculated how much energy the brood receives from this incubation event in total (*heatProvided_kJ*).
This incubation energy is then equally distributed over the brood (*heatProvidedPerBrood_kJ*) *HeatProvidedPerBrood_kJ* is summed up over time in each brood cohort (*cumulIncubationReceived_kJ*), which is one factor that determines when this individual (or cohort) advances into the next developmental stage. *SummedIncubationToday_kJ* keeps track of the total investment of a *colony* in brood incubation. The energy spent on incubation is then subtracted from the *colony's* energy store and *personalTime_s* for the incubating *bee* is increased by *heatingPeriod_s*.

```
to BroodIncubationProc
 let heatingPeriod_s 2880  ; time spent on incubation - ca. 48 min. between foraging
flights of incubating queen, Heinrich, p. 92, Fig. 5.2
 let incubationEnergy_kJ_per_mg_s 0.000000217013888 ; kJ per mg heating bee-mass per
                                                     ; second (calculation see above)
 let heatProvided_kJ heatingPeriod_s * incubationEnergy_kJ_per_mg_s
                                     * weight_mg * number ; [kJ] energy released by
                                     ; heating bee (cohort) during an incubation phase
 let heatProvidedPerBrood_kJ 0
 if [ allEggs + allLarvae + allPupae ] of  Colony colonyID > 0
  [ ; amount of energy brood receives per individual:
    set heatProvidedPerBrood_kJ
        heatProvided_kJ
        / [ allEggs + allLarvae + allPupae ] of  Colony colonyID ]
 let memoColonyID colonyID
 ask bees with [ colonyID = memoColonyID
          and (stage = "egg" or stage = "larva" or stage = "pupa") ]
  ; the brood now receives the energy, which is summed up in cumulIncubationReceived_kJ
  [ set cumulIncubationReceived_kJ
                cumulIncubationReceived_kJ + heatProvidedPerBrood_kJ ]

; energy spent for heating is subtracted from colonies' energy stores
 ask Colony colonyID
 [
   set summedIncubationToday_kJ summedIncubationToday_kJ + heatProvided_kJ
   set energyStore_kJ energyStore_kJ - heatProvided_kJ
 ]
 set personalTime_s personalTime_s + heatingPeriod_s  ; heating takes some time..
end
```

ForagingProc

Purpose: records the foraging activities and calls the other foraging procedures

Called by: *ActivityProc*

Asking agents: *bees* (workers and non-hibernating queens)

Calling:
Foraging_searchingProc
Foraging_collectNectarPollenProc

Foraging_costs&timeProc
Foraging_unloadingProc

<u>Description</u>

The global variable *TotalForagingTripsToday* keeps track of a day's total number of foraging trips from all colonies.
If a *bee*'s *activity* is "pollenForaging" then *pollenForager?* is set true, and *currentFoodsource* (the *foodsource* the *bee* will be visiting) is set *pollensourceToGoTo* (determined in *Foraging_PatchChoiceProc*).
If a *bee's activity* is "nectarForaging" then *pollenForager?* is set false, and *currentFoodsource* is set *nectarsourceToGoTo*.

The *activity* of *bees* without a *foodsource* to go (i.e. if *currentFoodsource* < 0) is set to "searching", otherwise the *bee*'s *activity* is to either "collectPollen" or "collectNectar". All changes of their *activity* is logged in their *activityList*.

Then the procedures dealing with the detailed processes of foraging are called: *Foraging_searchingProc*, *Foraging_collectNectarPollenProc*, *Foraging_costs&timeProc*, and *Foraging_unloadingProc*.

```
to ForagingProc
 set TotalForagingTripsToday TotalForagingTripsToday + number
 ifelse activity = "pollenForaging"    ; if bee decided to collect pollen..
  [
    set pollenForager? true     ; .. it becomes a pollen forager..
    set currentFoodsource pollensourceToGoTo
  ]
  [
    set pollenForager? false   ; or otherwise a nectar forager
    set currentFoodsource nectarsourceToGoTo
  ]

 ifelse currentFoodsource < 0 ; i.e. currentFoodsource  does not refer to
                              ; an existing food source
  [
    set activity "searching"
    set activityList lput "S" activityList
   ]
  [
    ifelse pollenForager? = true
      [
        set activity "collectPollen"
        set activityList lput "cP" activityList
      ]
      [
        set activity "collectNectar"
        set activityList lput "cN" activityList
      ]
  ]

 Foraging_searchingProc    ; unexperienced foragers search new flower patch

 set activityList lput (word "(" currentFoodsource ")"  ) activityList
 Foraging_collectNectarPollenProc  ; succesful scouts and experienced Foragers
                                   ; gather nectar
 Foraging_costs&choiceProc  ; energy costs for flights and trip duration
 Foraging_unloadingProc  ; ..and unload their crop & increase colony's honey store

 if (pollenLoadSquadron_g + nectarLoadSquadron_kj) > 0
    [ AssertionProc "Bee carries pollen or nectar after unloading! (ForagingProc)"]

end
```

Foraging_searchingProc

Purpose: determines if a scout bee (*activity* = "searching") finds a *foodsource*

Called by: *ForagingProc*

Asking agents: *bees* (workers and non-hibernating queens)

Calling:

DetectionProbREP
Foraging_bestLayerREP
Foraging_SortKnownPatchesListREP

Description

Only *bees* with *activity* = "searching" are addressed in this procedure. After defining some local variables, the location of the *bee*'s *colony* is saved (local variables *xcol*, *ycol*).
Depending on whether the *bee* is searching for nectar or pollen, *masterpatchesWith(Nectar or Pollen)layersInFlowerAndRangeList* is saved in the local variable *myMasterpatchesWithFoodList*. This list contains the ID/who of "masterpatches" representing a flower patch where at least one flower species (*foodsource*) currently provides the forage type the *bee* is after.

```
 to Foraging_searchingProc
; foragers with activity = "searching" may find a food source, other foragers
; (activity: collect nectar or pollen) don't do anything here

  if activity = "searching"
  [
    let chosenMasterpatch -1 ; bee hasn't found a food source yet
    let myMasterpatchesWithFoodList []
    let xcol NotSetHigh  ; saves the location of the bee's colony to determine the
                         ; detection probability
    let ycol NotSetHigh
    let pollenFor false
    if pollenForager? = true [ set pollenFor true ]

    ask colony colonyID
    [
      set xcol xcor
      set ycol ycor

      ifelse pollenFor = true
        [ set myMasterpatchesWithFoodList
              masterpatchesWithPollenlayersInFlowerAndRangeList ]
              ; only masterpatches are considered, otherwise, detection prob. would
      increase with the number of flowerspecies at a patch!
        [ set myMasterpatchesWithFoodList
              masterpatchesWithNectarlayersInFlowerAndRangeList ]
    ]
```

The list of flower patches offering the wanted forage (*myMasterpatchesWithFoodList*) is then shuffled and for each item of the list (ID/who of a "masterpatch") it is determined randomly whether or not its flower patch is detected by the *bee*. The probability to be detected is calculated in *DetectionProbREP*, based on the distance of the patch to the *colony*. In case of a detection, the local variable *chosenMasterpatch* is set to the current item (i.e. to *who* (the ID) of the "masterpatch" detected). As all items of the list are addressed, several detections may occur but only the flower patch detected last is then the chosen one:

```
; (if activity = "searching"...):
    foreach shuffle myMasterpatchesWithFoodList ; shuffled only once, not every time a
                                                ; new item is addressed!
    [
      if random-float 1 <  DetectionProbREP ? xcol ycol   ; all items in list are
   ; addressed, hence chosenMasterpatch may be set several times - only last patch
   ; detected is the patch chosen!
      [ set chosenMasterpatch ? ]             ; this is a masterpatch that has at least
                      ; 1 layer currently providing the forage the bee is searching for
```

```
            ]
```

If a flower patch was detected (i.e. if *chosenMasterpatch* >= 0) then the *bee* is going to exploit the most profitable *foodsource* ("layer") within this flower patch. This is determined in *Foraging_bestLayerREP*, and the *bee*'s *currentFoodsource* is set to *who* (ID) of this most profitable *foodsource*.
The *bee*'s *knownMasterpatchesPollen(Nectar)List* is then updated by adding chosenMasterpatch, its *activity* is set to "collectPollen" ("collectNectar") and logged in its *activityList*:

```
ifelse chosenMasterpatch >= 0  ; if the bee has found a patch:
[
 set currentFoodsource Foraging_bestLayerREP chosenMasterpatch  ; the bees new
    ; food source is then the best layer at that patch (based on handling time)
    ; (only sources actually providing the food the bee is after are considered)
 ifelse currentFoodsource >= 0
 [
   ifelse pollenForager? = true
   [
     set knownMasterpatchesPollenList
          fput chosenMasterpatch knownMasterpatchesPollenList
          ; food source is added to the list of known pollen patches
     set knownMasterpatchesPollenList
          Foraging_SortKnownPatchesListREP knownMasterpatchesPollenList
          ; the list is now sorted again by distances,
          ; with duplicates being removed
     set activity "collectPollen"
     set activityList lput "cP" activityList        ]
   [
       set knownMasterpatchesNectarList
          fput chosenMasterpatch knownMasterpatchesNectarList
          ; food source is added to the bees' list of known nectar patches
       set knownMasterpatchesNectarList
          Foraging_SortKnownPatchesListREP knownMasterpatchesNectarList
          ; the bees' list is now sorted again by distance,
          ; with duplicates being removed
     set activity "collectNectar"
     set activityList lput "cN" activityList
   ]
 ]
```

In (usually) rare cases, it may happen that a bee finds a "masterpatch" but no nectar or pollen is offered anymore at any of the "layers" (*foodsources* belonging to this flower patch), as it was visited by (other) *bees* and already depleted earlier this day. In this case - but also when no "masterpatch" was found in the first place - the *bee* will return empty (*activity* = "returningEmpty") with *currentFoodsource* = -1:

```
 [
   ; bee found a patch but with 0 nectar or pollen (because this foodsource
  ; was visited by bees and depleted today)
   set activity "returningEmpty"
   set activityList lput "rE0" activityList ; rE0: to distiguish from unsuccessful
          ; scout who haven't found a masterpatch in the first place
 ]
]
[
 ; otherwise, if bee does not find a patch at all:
   set activity "returningEmpty"
   set activityList lput "rE" activityList
]
]
if activity =  "searching"
     [ AssertionProc "Bee is still searching! (Foraging_searchingProc)"  ]
end
```

Foraging_SortKnownPatchesListREP

Purpose: removes duplicates in list of known *foodsources* and sorts it by distances of masterpatches and the bee's *colony*

Asking agents: *bees* with *activity* = "searching"

Called by: Foraging_searchingProc

Input: *knownPatchesList*

Description

This reporter-procedure receives a list of *foodsources* (*knownPatchesList*). Duplicates (i.e. *foodsources* represented several times) in the list are removed and then the list is ordered by the distances of the *foodsources* to the *colony* of the scout *bee*, starting with the closest *foodsource*.

```
to-report Foraging_SortKnownPatchesListREP [ knownPatchesList ]
; removes duplicates in list and sorts it  by distances of masterpatches
; and the bee's colony

 let newList []
 set knownPatchesList remove-duplicates knownPatchesList ; duplicates are removed
 ask Colony colonyID ; this is the colony of the scouting bee
   [ set newList sort-by [distance (Foodsource  ?1) < distance (Foodsource ?2)]
        knownPatchesList ] ; (division by SCALING_NLpatches/m is not necessary here!)
 report newList
end
```

DetectionProbREP

Purpose: calculates the detection probability of a patch, based on its distance to the *colony* of the searching *bee*.

Asking agents: *bees*

Called by: *Foraging_searchingProc*

Input: *patchWho, xcol, ycol*

Description

The distance between the *colony* location and the location of the centre of the *foodsource* is calculated (unit: NetLogo gridcells) and divided by *SCALING_NLpatches/m* to translate it into meter of real distance (*dist_m*). This distance is then reduced by the radius (*radius_m*) of the *foodsource* and saved as *relevantDistance_m* (>= 0m). The detection proability (*detProb*) is then calculated as suggested by Becher et al. (2016), with *Lambda_detectProb* being an input set by the user. This allows the user to make detection probabilities dependent on the structure of the landscape. If the *foodsource* is beyond the maximal foraging range (global variable *ForagingRangeMax_m*) it can't be detected (*detProb* = 0):

```
to-report DetectionProbREP [ patchWho xcol ycol ]
  let dist_m 0
  let patchRadius_m -999

  ; calculation of the distance between Foodsource and Colony:
  ask Foodsource patchWho
    [
      set dist_m (distancexy xcol ycol) / SCALING_NLpatches/m
      set patchRadius_m radius_m
     ]
  ; calculation of the detection probability, based on the distance
```

```
  ; (see BEESCOUT model, Becher et al. 2016, Ecological Modelling):

  let relevantDistance_m dist_m - patchRadius_m
       ; the "relevant" distance is the distance to the edge of the field,
       ; i.e. dist_m (to centre) - patchRadius_m
  if relevantDistance_m < 0 [ set relevantDistance_m 0 ] ; no negative distances!
  let detProb e ^ (Lambda_detectProb * relevantDistance_m)

  if relevantDistance_m > ForagingRangeMax_m
       [ set detProb 0 ] ; patch is beyond the colonies foraging range
  report precision detProb 10
end
```

Foraging_collectNectarPollenProc

Purpose: collection of nectar or pollen at a certain patch by a foraging bee

Called by: *ForagingProc*

Asking agents: *bees* (workers and non-hibernating queens)

Calling: none

<u>Description</u>

Bees with *activity* "collectPollen" (i.e. successful scouts) or *activity* "expForagingP" (i.e. experienced pollen foragers continuing to exploit a *foodsource* previously visited) will gather a pollen load from the *foodsource*, their *activity* is changed to "bringingPollen" and their *activityList* is updated. The amount of pollen collected is removed from the *foodsource* and the visit is counted in the *foodsource* variable *cumulPollenVisits*. If not enough pollen for a full pollen load is left at the patch, the *bees* will only collect as much pollen as available. If no pollen at all is left, their *activity* is set to "returningEmpty":

```
to Foraging_collectNectarPollenProc
; foragers with activity = "collectPollen" or activity = "expForagingP" OR
; activity = "collectNectar" or activity = "expForagingN" can gather food from
; a food source. No other bees are addressed
  if activity = "collectPollen" or activity = "expForagingP"
  [

    ; does patch still have any pollen?:
    ifelse [ pollen_g ] of Foodsource currentFoodsource > 0
      [ ; the forager will then be bringing pollen:
        set pollenLoadSquadron_g min list ; takes the smaller value of an
                                          ; ad hoc created
;list with two items: 1st: max. pollen bee (cohort) can carry, 2nd: pollen left at patch.
          (pollenPellets_g * number)      ; 1st: max. pollen bee (cohort) can carry
          ([ pollen_g ] of Foodsource currentFoodsource) ; 2nd: pollen left at patch
        set activity "bringingPollen"
        set activityList lput "P" activityList  ; activity log
        ; amount of pollen at the patch is reduced
        let memoNumber number
        let memoPollenLoad pollenLoadSquadron_g
        ask Foodsource currentFoodsource  ; visited food source is addressed
          [
            set pollen_g pollen_g - memoPollenLoad ; and the pollen removed
            set cumulPollenVisits cumulPollenVisits + memoNumber
               ; all pollen visits at patch, ever
          ]
      ]
      [
        set activity "returningEmpty"
```

```
                set activityList lput "Ep" activityList ; activity log
              ]
           ]
```

In a similar way, gathering nectar takes place. If nectar is present at the *foodsource*, the *bee* fills her crop with either the maximal volume she can carry or the amount of nectar left at the *foodsource* (*nectar_myl*), whatever is lower. The energy (*nectarLoadSquadron_kJ*) of the collected nectar is calculated from the volume (*cropvolume_myl* multiplied by *number*, the number of bees in the cohort) and the sugar concentration (*nectarConcentration_mol/l*). *Activity* is set to "bringingNectar" and the *activityList* is updated, the nectar volume collected is removed from the *foodsource*, and the visit is counted (*cumulNectarVisits*) at the patch. If no nectar is available at the patch anymore, the bees return empty:

```
   if activity = "collectNectar" or activity = "expForagingN"
    [
      ; does patch still have any nectar?:
      ifelse [ nectar_myl ] of Foodsource currentFoodsource > 0
        [ ; the forager will then be bringing nectar:
          let nectarRemoved min list  ; takes the smaller value of an ad hoc created list
                                      ; with two items: 1st: max. nectar a bee (cohort)
                                       ; can carry, 2nd: nectar left at patch.
            (cropvolume_myl * number)  ; 1st item: max. nectar a bee (cohort) can carry
            ([ nectar_myl ] of Foodsource currentFoodsource)   ; 2nd item: max. pollen
                                                               ; a bee (cohort) can carry

          set nectarLoadSquadron_kJ nectarRemoved * EnergySucrose_kJ/mymol
                * [ nectarConcentration_mol/l ] of Foodsource currentFoodsource
            ; set the nectar energy load with the amount removed

          set activity "bringingNectar"
          set activityList lput "N" activityList
          ; amount of nectar at the patch is reduced:
          let memoNumber number
          ask Foodsource currentFoodsource
           [
             set nectar_myl nectar_myl - NectarRemoved
             set cumulNectarVisits cumulNectarVisits + memoNumber
                 ; all nectar visits at patch, ever
           ]
        ]
        [
          ; if NECTAR foodsource is EMPTY:
          set activity "returningEmpty"
          set activityList lput "En" activityList  ; "Empty nectar"
        ]
     ]
  end
```

Foraging_costs&choiceProc

Purpose: calculates mortality, duration and energetic costs of foraging trip

Called by: *ForagingProc*

Asking agents: *bees* (workers and non-hibernating queens)

Calling:

DieProc
Foraging_PatchChoiceProc

Description

Mortality risk and energetic costs of a foraging trip depends on the duration or covered distance of the trip. For *bees* returning empty, the distance covered by an (unsuccessful)

search trip is set to the *species*-specific value *searchLength_m*. The energetic costs are not paid by the foraging *bee* but by its *colony* instead and are calculated from the search length multiplied with the *species*' energetic flight costs per meter (*flightCosts_kJ/m*) and the number of bees represented by this cohort (*number*). The duration is calculated from the distance covered and the flight speed (*flightVelocity_m/s*). The probability of a *bee* to survive the foraging trip is the probability to survive a single second of foraging (1 - *MortalityForager_per_s*) to the power of the trip duration [s]. The risk of death is then 1 - the probability of survival. *ForagingMortalityFactor* (set on GUI, default value: 1) allows the user to easily modify the foraging mortality.

```
to Foraging_costs&choiceProc ; costs in time, energy and mortality
; first bees with activity = "returningEmpty" and then bees with  activity =
; "bringingNectar" or activity = "bringingPollen" are addressed to calculate time
; and energy spent on the trip. Finally Foraging_PatchChoiceProc is called, as
; the future patch/flowerspecies choice is based on the time spent on the trip.

 let persTimeSave personalTime_s ; the current time
 let saveNumber number   ; number of individuals, this cohort/forager agent represents
 let saveWeight_mg weight_mg     ; save variable for weight of individual/s
 let saveFlightCosts_kJ/m/mg  [ flightCosts_kJ/m/mg ] of OneSpecies speciesID
 ; Test for errors:
 if not member? caste ["worker" "queen"]
    [ AssertionProc (word stage " " caste " " who " shouldn't be foraging
                     (Foraging_costs&timeProc)")]
 if saveFlightCosts_kJ/m/mg < 0
    [ AssertionProc "saveFlightCosts local variable not set (Foraging_costs&timeProc)"]

; EMPTY BEES:
 if activity = "returningEmpty"
  [
    ; nectar store in the colony is reduced to reflect the energy consumed
    ; during the trip:
    let tripDuration_s 0
    ask Colony colonyID
    [
       set energyStore_kJ energyStore_kJ
               - ( [ searchLength_m ] of OneSpecies speciesIDcolony
               * saveFlightCosts_kJ/m/mg * saveNumber * saveWeight_mg )
       set tripDuration_s [ searchLength_m ] of OneSpecies speciesIDcolony
           / [ flightVelocity_m/s ] of OneSpecies speciesIDcolony
    ]
    set personalTime_s personalTime_s + tripDuration_s    ; some time has passed..
    ; a Bee dies during the foraging trip, unless she survives every single second:
    let survivalChance (1 - MortalityForager_per_s * ForagingMortalityFactor)
       ; probability to survive a single second of the foraging trip
       ;    ^ tripDuration_s        ; ... to survive EACH second of the trip
    let mortalityRisk 1 - survivalChance   ; risk to die = 1 - probability to survive
    if random-float 1 < mortalityRisk [ DieProc "foraging: empty" ] ; does bee die?
  ]
```

If *bees* were successful in collecting nectar or pollen, then the energetic costs are calculated by doubling the distance to the *foodsource* they visited (multiplied by flight costs per meter) and adding the energy spent within the patch. This energy spend within a flower patch is calculated from the handling time multiplied by the flight speed (i.e. resulting in a distance) and a factor (*EnergyFactorOnFlower*) which reduces the energy as bees spend some time sitting on flowers. Again, cohort size (*number*) is taken into account. The *bees' personalTime_s* is updated, and the survival of the *bees* is determined as described above. Finally, *Foraging_PatchChoiceProc* is called to determine if the *bees* switch to another *foodsource*.

```
; SUCCESSFUL FORAGERS:
 ; energy consumption of successful foragers:
 let handlingTime_s 0
 if activity = "bringingNectar" or activity = "bringingPollen"
  [
    if activity = "bringingNectar"
      [
        set handlingTime_s HandlingTime_s_REP currentFoodsource pollenForager?
        set activity "expForagingN"
        set activityList lput "Xn" activityList
      ]
```

```
        if activity = "bringingPollen"
          [
            set handlingTime_s HandlingTime_s_REP currentFoodsource pollenForager?
            set activity "expForagingP"
            set activityList lput "Xp" activityList
          ]
        let memoPatch currentFoodsource
        let tripDuration_s 0
        ask Colony colonyID
        [
          set energyStore_kJ energyStore_kJ -
               (
                 2 * distance (Foodsource memoPatch)
                   / SCALING_NLpatches/m ; bees have to fly to the patch and back,
                                         ; distance [NLpatches] / Scaling = [m]
                                         ; plus distances they fly within the patch,
                                         ; but reduced by rests on flowers
                 + handlingTime_s
                   * [ flightVelocity_m/s ] of OneSpecies speciesIDcolony
                   * EnergyFactorOnFlower    ; [kJ] = [m * kJ/m + kJ/m * s * m/s]
                )
                * saveFlightCosts_kJ/m/mg * saveWeight_mg  ; flight costs (kJ) per m,
                                                           ; dependent on the bees' weight
                * saveNumber                ; multiplied by number of bees in the cohort
           set tripDuration_s (2 * distance (Foodsource memoPatch)
                                  / SCALING_NLpatches/m
                                  / [ flightVelocity_m/s ] of OneSpecies speciesIDcolony)
                                 + handlingTime_s
        ]
        set personalTime_s personalTime_s + tripDuration_s    ; some time has passed..
        ; MORTALITY:
       ; probability to survive a single second of the foraging trip:
        let survivalChance (1 - MortalityForager_per_s * ForagingMortalityFactor)
                           ^ tripDuration_s    ; ... to survive EACH second of the trip

        ; ForagingMortalityFactor (set on GUI) allows to easliy modify foraging mortality
        let mortalityRisk 1 - survivalChance   ; risk to die = 1 - probability to survive
        if random-float 1 < mortalityRisk [  DieProc "foraging: N or P forager" ]
        set activityList lput (word "HT:" precision handlingTime_s 1) activityList
      ]

      if personalTime_s - persTimeSave <= 0
         [ AssertionProc "No time - or negative time - passed for this bee!
                          (Foraging_costs&timeProc)" ]
      Foraging_PatchChoiceProc personalTime_s - persTimeSave ; the bee makes a choice about
                                                             ; where to forage based on the trip duration
    end
```

Foraging_PatchChoiceProc

Purpose: determines if *bees* are still happy with their current *foodsource* (based on the duration of the trip). If not, they will either switch to the best "layer" (flower species) at their current flower patch or - if they already forage at the best "layer" (flower species), they will switch to another flower patch ("layergroup") they know or search for a new one

Called by: *Foraging_costs&choiceProc*

Asking agents: *bees* (workers and non-hibernating queens)

Calling: *Foraging_bestLayerREP*

Input: *currentTripDuration_s*

<u>Rationale & terminology:</u>
In the ODD protocol we speak of real world forage habitats as "flower patches" and implement them in the model via *foodsources*. As each *foodsource* represents only a single flower species, semi-natural habitat patches with a large number of different flower species have to be implemented by several *foodsources*. In this case, the first *foodsource* of such a semi-natural habitat patch created in the *Setup* procedure will be used to store some statistics

concerning the flower patch as a whole. This first *foodsource* is hence called a "masterpatch". We sometimes refer to *foodsources* belonging to semi-natural habitat patch also as "layers" (as they can be imagined as being stacked up on each other at the location of the flower patch). When scouting *bees* find a flower patch they detect the whole flower patch (i.e. all *foodsources* at this location) and memorise the ID of the respective masterpatch (saved in their *knownMasterpatchesNectar(Pollen)List* list). However, for the actual nectar and pollen collection, they only exploit a specific *foodsource* within this flower patch, representing a specific flower species. *Bees* can switch between foraging trips (but not within) from one *foodsource* to another at the same flower patch, but they can also move to a flower patch at a different location.
The ID (*who*) of "masterpatches" saved in the *bee*'s *knownMasterpatchesNectar(Pollen)List* are ordered by the distance of the food patch to the *bee*'s *colony*, i.e. the first item in the list refers to a flower patch closer to the *colony* as the second item in the list etc.. We assume that the foraging distance results from a trade-off between foraging close enough to the *colony* to save time and energy and far enough to access high quality and quantity resources (as closer patches might have already been depleted by the *colony*). When a *bee* quits exploiting her current flower patch and decides to re-visit another, but already known flower patch, she will pick a flower patch either closer to the *colony* than the current patch (i.e. any of those listed in *knownMasterpatchesNectar(Pollen)List* before her current patch) or the flower patch that is just a bit further away (i.e. the one that is listed just behind her current patch). If no such flower patch exists, the bee will search for new *foodsources* during the following trip. The decision of whether or not the current *foodsource* is abandoned depends on the actual duration of the foraging trip in comparison to the expectation of the bee.

Description

When *Foraging_PatchChoiceProc* is called by *Foraging_costs&choiceProc*, it gets the duration of the calling *bee*'s foraging trip as input (*currentTripDuration_s*). At the beginning of the procedure, some local variables are defined:

```
to Foraging_PatchChoiceProc [ currentTripDuration_s ]
   ; determines if bees are still happy with their current food source (based on the
   ; duration of the trip). If not, they will either switch to the best
   ; layer/flowerspecies at the
   ; current patch or - if they already forage at the best layer/flowerspecies, they
   ; will switch to another patch/'layergroup' they know or search for a new one
   ; (note: expectation_Nectar/PollenTrip_s is 0 for a novice forager, hence they are
   ; likely to search new patches until they get more experienced)
   ; Ref: Wiegmann et al 2003, Physiology & Behavior 79 (2003) 561- 566

  let preferenceClosePatchesProb 0.9 ; heuristically determined to result in
                                     ; highest numbers of hibernating queens
  let happy? true ; defines whether or not a bee is still happy with
                  ; her current food source
  let gotoNewLayergroup? false ; whether or nat the bee is going to exploit
                               ; a different flower patch
  let bestLayer -1 ; ID (who) of best food source in the current flower patch.
    ; As long as the bee is happy, it doesn't matter which foodsource is the best layer
  let myExpectation_s expectation_NectarTrip_s ; expectation of a bee about
                                               ; the duration of the trip
  let myKnownMasterpatchesList knownMasterpatchesNectarList
  let searchProbBase 0.1
  if pollenforager? = true
   ; some changes if a bee is a pollen forager and not a nectar forager:
   [
     set myExpectation_s expectation_PollenTrip_s
     set myKnownMasterpatchesList knownMasterpatchesPollenList
   ]
  let myMasterpatchID -1
  let myCurrentPatchPosition -1
```

Bees returning empty will always search a new flower patch (i.e. *gotoNewLayergroup?* is set *true*), whereas *bees* who were able to collect nectar or pollen base their decision about continuing with their current *foodsource* on the duration of the foraging trip. This decision is made randomly, with the probability being the sum of a base probability to search (*searchProbBase*) and a degree of "unhappiness" with the current *foodsource*, which results from a comparison of the trip duration with the expected duration of the trip. The new expectation for the duration of the next trip are then calculated as the mean of the current expectation and the current trip duration.

```
; the longer a trip takes in comparison to a bees' expectation, the higher is the
; probability to become unhappy. Expectations are then recalculated as the mean of the
; duration of current trip and the previous expectation
 ifelse activity = "returningEmpty"
   [ set gotoNewLayergroup? true] ; unsuccessful bees always search for
                                       ; a new flower patch/'layergroup'
   [
     set myMasterpatchID [ masterpatchID ] of foodsource currentFoodsource
     if position myMasterpatchID myKnownMasterpatchesList = false
        [ AssertionProc ("No number for myMasterpatchID (Foraging_PatchChoiceProc)") ]
     ; the "position" (in the list) of the currently used 'layergroup'
     ; in the myKnownMasterpatchesList:
     set myCurrentPatchPosition position myMasterpatchID myKnownMasterpatchesList
     ; NeLogo command "position": "On a list, reports the first position of item
     ; in list, or false if it does not appear."
     if myCurrentPatchPosition  = false [ set myCurrentPatchPosition -1 ] ; to avoid an
                 ; error if myMasterpatchID is not part of the myKnownMasterpatchesList
     ; determine whether or not a bee becomes unhappy with her current foodsource:
     let unhappyProb (currentTripDuration_s - myExpectation_s) / currentTripDuration_s
         ;  bee compares her expectations on trip duration with the acutal duration
     if unhappyProb < 0 [ set unhappyProb 0 ]    ; no negative probability
       ; the probability to search a new patch then depends on searchProbBase
       ; and her degree of unhappiness:
     if random-float 1 < (searchProbBase + unhappyProb)
                         [ set happy? false ] ;
     let newExpectation (myExpectation_s + currentTripDuration_s) / 2
       ; new expectation take duration of current and previous trips into account
     ifelse pollenforager? = true  ; the new expectations are saved..
      [ set expectation_PollenTrip_s newExpectation ] ; as expect. for pollen trips
      [ set expectation_NectarTrip_s newExpectation ]  ; .. or for nectar trips
   ]
```

If bees are no longer happy with their current *foodsource* (i.e. if *happy?* is *false*) the *bee*'s *activity* is set to "returningUnhappyP(N)" and it may switch to a more profitable foodsource ("layer") within this flower patch. The "best" (most profitable) *foodsource* is determined in the reporter-procedure *Foraging_bestLayerREP*. If the bee is not currently using the best *foodsource* of the flower patch (*bestLayer*), she then switches to the best (i.e. *currentFoodsource* is set to *bestLayer*). If it turns out that is already foraging at the best *foodsource* of that flower patch (or "layergroup"), then *gotoNewLayergroup?* is set *true* and the *bee* will abandon the current flower patch (though she still remembers it and might return to it some time later).

```
 if happy? = false
    [
      set activityList lput ":(" activityList  ; sad smiley added to activityList
      ifelse pollenforager? = true
        [ set activity "returningUnhappyP" ]
        [ set activity "returningUnhappyN" ]
      set bestLayer Foraging_bestLayerREP currentFoodsource
      ifelse bestLayer = currentFoodsource
        [ set gotoNewLayergroup? true ] ; if bee is already foraging from
       ; the best layer, it will search for a completely now patch/'layergroup'
        [ set currentFoodsource bestLayer ]     ; .. otherwise it will stay
       ; at the current patch but switch to the best foodsource/flowerspecies here
    ]
```

If a *bee* decides to abandon its current flower patch and re-visit another, already known flower patch, it is determined whether she goes to a patch closer to the *colony* as the current patch (with the probability *preferenceClosePatchesProb*) or one (a little) further away. *MyCurrentPatchPosition* defines the location where the ID (*who*) of the current flower patch

(or more precisely, of the "masterpatch" of the layergroup, representing this flower patch) is stored in the list *myKnownMasterpatchesList*. The items of this list are ordered by the distance of the flower patches they are representing to the *colony*, starting with the closest ones (note that the first item in the list has the position 0, the second 1 etc.). If the bee goes to a patch closer to the *colony*, a random position (between -1 and *myCurrentPatchPosition* - 1) is chosen. The item at this position defines the ID of the new flower patch. If the position is -1, no flower patch is chosen and the bee will become a scout and search for a new patch in her next foraging trip.
If the *bee* goes to a flower patch further away from the *colony*, the position on the *myKnownMasterpatchesList* is not randomly picked but is *myCurrentPatchPosition* + 1. If *myCurrentPatchPosition* already is the last item of the list, no flower patch is chosen and the bee will also become a scout in her next foraging trip.

```
        if gotoNewLayergroup? = true
          [
            set activityList lput "..." activityList
            let newPatchPosition -999
           ; the new patch has a similar distance to the colony as the old patch,
           ; but patches closer to the colony are preferred (preferenceClosePatchesProb = 0.6)
            ifelse random-float 1 < preferenceClosePatchesProb
             [ set newPatchPosition random (myCurrentPatchPosition + 1) - 1 ]
               ; a random position < current position, includ. 0 and -1 (-1 results in search
               ; of a new flower patch. Prob. decreases, the more patches are known)
             [ set newPatchPosition myCurrentPatchPosition + 1 ]
            ifelse newPatchPosition < 0 or newPatchPosition >= length myKnownMasterpatchesList
             [
               set currentFoodsource -1    ; bees will search for a
                                            ;completely new flower patch
             ]
             [ set currentFoodsource
                      Foraging_bestLayerREP item newPatchPosition myKnownMasterpatchesList ]
                        ; bee goes to a flower patch it already knows and chooses
                        ; the best foodsource (layer) there
          ]
         ifelse pollenforager? = true
           [ set pollensourceToGoTo currentFoodsource ] ; the (new) current foodsource will
                                                      ; be used for the next pollen foraging trip
           [ set nectarsourceToGoTo currentFoodsource ] ; the (new) current foodsource will
                                                        ; be used for the next nectar foraging trip
       end
```

<u>For example:</u>
Let *pollenforager?* be *true* and *activity* be "expForagingP"
Let *knownMasterpatchesPollenList* be [7 21 15 9 12 26] and hence the local variable *myKnownMasterpatchesList* will then also be [7 21 15 9 12 26]
Let *myMasterpatchID* be 9 and *currentFoodsource* be 10.
Let *expectation_PollenTrip_s* and hence also *myExpectation_s* be 100 [s]
Let *currentTripDuration_s* be 150 [s].

This describes a sucessful pollen forager who knows six flower patches where pollen is available. She currently exploits *foodsource* 10 wich belongs to a flower patch represented by the "masterpatch" 9. As her *activity* is not "returningEmpty" she might continue exploiting *foodsource* 10 in future. *MyCurrentPatchPosition* is 3 (9 (= the masterpatch ID) is the 4th item in *myKnownMasterpatchesList* hence its position is 3 (as counting in Netlogo lists starts with 0)). *UnhappyProb* is (150 - 100) / 150 = 0.333.
Let the random number be smaller than *searchProbBase* (0.1) + 0.333 and hence *happy?* is set *false*. *NewExpectation* (and hence *expectation_PollenTrip_s*) is then (100 + 150) / 2 = 125 [s].
The *bee*'s new *activity* is now "returningUnhappyP" and she might switch to another flower species (i.e. to another *foodsource*) at the same flower patch. Assuming *bestLayer*, determined

in *Foraging_bestLayerREP*, is 10 - i.e. the *foodsource* the bee is already exploiting - then *gotoNewLayergroup?* is set *true* and the *bee* will switch to another flower patch.
To determine her new flower patch, a random integer number between [-1..2] is drawn. If the result is 1, then the ID (*who*) of the "masterpatch" representing the flower patch is 21 (item 1 i.e. the second item in *myKnownMasterpatchesList* [7 21 15 9 12 26]). Assuming there a 5 flower species available at this flower patch then it is represented by 5 *foodsources* (with *who* = 21, 22, 23, 24 and 25). The resulting most profitable foodsource, determined in *Foraging_bestLayerREP*, may be 23, then the *bee*'s *currentFoodsource* and *pollensourceToGoTo* is set to 23 and this will be the foodsource visited on her next pollen foraging trip.
If the decision of the bee would be to visit a flower patch further away, then it would go to one of the *foodscources* represented by masterpatch 12.

Foraging_bestLayerREP

Purpose: reports most profitable *foodsource* ("layer") within the currently used flower patch, based on minimal handling time

Asking agents: *bees* (workers and non-hibernating queens)

Called by: *Foraging_searchingProc*, *Foraging_PatchChoiceProc*

Calling: *HandlingTime_s_REP*

Input: *myCurrentFoodsource*

<u>Description</u>

For each *foodsource* ("layer") of the flower patch currently used by the *bee*, it is checked whether it offers the forage type (nectar or pollen) the *bee* is searching. If it does, the current handling time is calculated in the reporter-procedure *HandlingTime_s_REP* (note that handling time increases with depletion of the *foodsource*). The *foodsource* with the shortest handling time is memorised (*memoBestPatch*) and then reported:

```
to-report Foraging_bestLayerREP [ myCurrentFoodsource ]
; reports most profitable foodsource ("layer") within the currently used flower patch,
; based on minimal handling time

 let memoBestHandlingtime notSetHigh ; to store the shortest handling time so far
 let memoBestEEF notSetLow
 let memoBestPatch -1
 let myBeeID who
 let distanceColonyFoodpatch_m 0  ;; distance (same for all layers!) will be set now:
 ask colony colonyID
  [ set distanceColonyFoodpatch_m ; distance between the colony and the food patch
        distance (Foodsource myCurrentFoodsource) ; the distance in NetLogo patches
        / SCALING_NLpatches/m ] ; div. by scling => distance in m
 foreach [ layersInPatchList ]  of foodsource myCurrentFoodsource ; for each foodsource
        ; of the bees flower patch, the handling time is calculated
  [
     let currentLayer ?
     if pollenforager? = true and [ pollen_g ]  of foodsource currentLayer > 0
                         ; only patches that actually provide pollen are considered
     [
       ask bee myBeeID
       [
         ; handling time is determined:
         let handlingTime_s HandlingTime_s_REP currentLayer pollenforager?
         if handlingTime < memoBestHandlingtime ; and if it is the shortest so far..
         [
           set memoBestPatch currentLayer  ; the ID of this foodsource..
```

```
            set memoBestHandlingtime handlingTime  ; and the handling time are saved
          ]
        ]
       ]

       if pollenforager? = false and [ nectar_myl ] of foodsource currentLayer > 0
                         ; only patches that actually provide nectar are considered
       [
        ask bee myBeeID
        [
          ; handling time is determined:
          let handlingTime_s HandlingTime_s_REP currentLayer pollenforager?
          let energyCostsThisLayer_kJ ; energy needed to exploit this layer:
             (

              2 * distanceColonyFoodpatch_m ; bees fly to and return from food patch
                 ; plus distances they fly within the patch,
                 ; but reduced by rests on flowers:
              + handlingTime_s
                * [ flightVelocity_m/s ] of OneSpecies speciesID ; [s] * [m/s] = [m]
                * EnergyFactorOnFlower
             )
             * [ flightCosts_kJ/m/mg ] of OneSpecies speciesID * weight_mg
                       ; flight costs (kJ) per m, dependent on the bees' weight[mg]
             * number   ; [m] * [kJ/m/mg] * [mg] => [kJ]

          ; energy gained when exploiting this layer:
          let energyGainThisLayer_kJ cropvolume_myl
                  * EnergySucrose_kJ/mymol ; [ul] * [kJ/umol] => [kJ/mol * l]
                  * [ nectarConcentration_mol/l ] of Foodsource currentLayer
                        ; [kJ/mol * l] * [mol/l] => [kJ]
          ; energetic efficiency of exploiting this layer:
          let eef (energyGainThisLayer_kJ - energyCostsThisLayer_kJ)
                   / energyCostsThisLayer_kJ
          if eef > memoBestEEF   ; if it is the energetically best so far..
          [
            set memoBestPatch currentLayer          ; ..the ID of this foodsource..
            set memoBestHandlingtime handlingTime   ; ..and the handling time are saved
          ]
        ]
       ]
     ]
    report memoBestPatch ; this might be negative (-1), if no foodsource was found!
   end
```

HandlingTime_s_REP

Purpose: calculates the time a bee needs to spend in a food patch to collect a full nectar load

Asking agents: *bees* (workers and non-hibernating queens)

Called by: *Foraging_costs&timeProc*

Calling: none

Input: *myPatch, pollenPatch?*

<u>Description & Rationale</u>

The calculation of the handling time for nectar collection mainly follows a model by Harder (Harder 1983). It is based on the weight [mg] of the bee, its glossa length [mm] and the corolla depth [mm] and nectar volume [µl] of the flower.
The only change made to Harder's model was to also take into account that with the *bees* using a flower patch, more and more flowers will be depleted. We hence consider the degree of depletion. As this may result for almost completely emptied patches in unrealistically high handling times, we also define a maximal handling time (*maxHandlingTime_s*).

When the reporter-procedure is called, two variables are transferred, the first one (*myPatch*) defining the ID (*who*) of the *foodsource* and the second one (*pollenPatch?*) whether the handling time for nectar or for pollen is asked for.
First, some local variables are created. *MaxHandlingTime_s* is derived from honeybees (and hence likely overestimating the maximal handling time for bumblebees). *FillingLevel* describes the current amount of the forage available at the patch relative to the possible maximum on that day. If *pollenPatch?* is false, *handlingTime_s* will be calculated on the basis of nectar availability, flower shape, and the *bees'* proboscis length and hence *fillingLevel* is set to *nectar_myl* divided by *nectarMax_myl*:

```
to-report HandlingTime_s_REP [ myPatch pollenPatch? ] ; called by bee
; calculates the time [s] to gather of full load of nectar or pollen
; for nectar: based on Harder 1983: Oecologia 57:274-280

 let maxHandlingTime_s 60 * 60 ; approx. max. from Ings et al. 2006, Fig. 1; Journal of
       Applied Ecology, 43,940-948; also comparable to data from Fig. 6 in Stelzer et al 2010,
       PloS One, 5(3), e9559
 let handlingTime_s -999  ; will be re-set to correct value below
 let fillingLevel 0 ; amount of food (nectar or pollen) currently at the patch relative
                       ; to its max. value for today, (correct value calculated below)
  ifelse pollenPatch? = false
 ; NECTAR FORAGING:
 [
  ask foodsource myPatch
   [
     if nectarMax_myl > 0
       [ set fillingLevel  nectar_myl / nectarMax_myl ]
   ]
```

Then the equations provided by Harder (1983) in his Fig. 4 are implemented. First, local variables are created for the parameters required, with the first letters of the parameter name referring to the respective identifier in Harder 1983 (where applicable). *V_nectarVolume_myl* is the amount of nectar available in a single flower in microlitre. *Ta_accessTime_s* describes the time [s] required to enter and leave a flower (Harder 1983, eq. 7). *Ti_ingestionTime_s* is the time required to consume the nectar provided by the flower (Harder 1983, eq. 8).

```
  ; Harder 1983, Fig. 4:
  let W_beeWeight_g weight_mg / 1000
  let G_lengthGlossa_mm glossaLength_mm
  let C_CorollaDepth_mm [ corollaDepth_mm ] of Foodsource myPatch
  let V_nectarVolume_myl [ nectarFlowerVolume_myl ] of Foodsource myPatch
  let Ta_accessTime_s 0.3 + 0.04 * C_CorollaDepth_mm ; time to access a flower
  let numerator log (V_nectarVolume_myl + 1) 10
  let num 0.3 * W_beeWeight_g ^ 0.3333 * G_lengthGlossa_mm
  let base (1.41 - C_CorollaDepth_mm / G_lengthGlossa_mm)
  if base < 0.001 [ set base 0.001 ]    ; as 0 ^ -0.4 is not valid
         ; (in calculation of local variable den, see below)
  let den (base ^ -0.4) - 0.3 * Ta_accessTime_s
  let denominator log (num / den + 1) 10
  let Ti_ingestionTime_s numerator / denominator
```

Harder calculates the total probing time as the sum of access time and ingestion time. However, this describes only how long it takes a bee to empty a single flower and does neither take the density of flowers, nor the proportion of already emptied flowers, or the *bees'* crop size into account. We therefore calculate *handlingTimePerFlower_s* the following way: we add the average time to fly from one flower to the next (*interFlowerTime_s*, a *foodsource* specific parameter provided in the input file *FlowerspeciesFile*) to *Ta_accessTime_s* and then divide by *fillingLevel* to account for the proportion of flowers already emptied. Then *Ti_ingestionTime_s*, the time to take up the nectar in the flower, is added:

```
  let handlingTimePerFlower_s maxHandlingTime_s  ; handling time set to maximal value..
  if fillingLevel > 0  ; avoid division by 0     ; .. unless there is nectar available,
```

```
      ; then it is recalculated (if the new value is lager than maxHandlingTime_s,
      ; it will be set back to maxHandlingTime_s at the end of this procedure)
 [
   set handlingTimePerFlower_s (
                                (
                                 [ interFlowerTime_s ] of Foodsource myPatch
                                          ; the time to travel to the next flower
                                 + Ta_accessTime_s
                                          ; + the time to test whether it contains nectar
                                )
                               / fillingLevel      ; divided by the filling level to
                                                   ; account for depletion of the patch
                              )
                              + Ti_ingestionTime_s    ; + time to actually load the nectar,
                                                      ; once a filled flower is found
 ]
```

The total time spent in the flower patch is then *handlingTimePerFlower_s* times the number of flowers needed to to fill the *bees'* crop:

```
 let flowersVisited 1 ; at least one flower has to be visited..
 if V_nectarVolume_myl < cropvolume_myl ; but ususally more than one flower is needed:
              [ set flowersVisited (cropvolume_myl / V_nectarVolume_myl) ]
 set handlingTime_s handlingTimePerFlower_s * flowersVisited  ; the time to find a flower and
      ; empty it is then multiplied by the number of flowers, needed to fill the crop
]
```

If *pollenPatch?* is true, *handlingTime_s* is calculated on the basis of the pollen availability at the *foodsource*. As to our knowledge no theoretical method is available to calculate handling times for pollen collection which takes bee- and flower species into account, this procedure is very simplified. Parameterisation is based on poppy flowers (Raine & Chittka 2007) and is likely to be different for other flower species. The proportion of flowers still offering pollen on that day (*FillingLevel*) is set to *pollen_g* divided by *pollenMax_g*. The calculation of the actual handling time is then equivalent to the one for nectar collection, as described above.

```
 ; POLLEN FORAGING:
 [

 let timeInFlowers_s 257.4  ; time  bee spents in flower(s) to collect 1 pollen load, derived
         ;(for poppy flowers) from Raine & Chittka 20072007,
         ; Tab. 1, "Number of flowers visited"
         ; times "Mean flower handling time/ s" (mean of all three bouts)
 let flowersNeededForPollenLoad 58  ; Raine & Chittka 2007, Tab. 1
                                    ; "Number of flowers visited" (mean of all 3 bouts)

 ask foodsource myPatch    ; get the filling level for this foodsource:
  [
    ifelse pollenMax_g > 0
     [ set fillingLevel  pollen_g / pollenMax_g ]
     [ set fillingLevel  0 ] ; (this should actually never be the case)
  ]

 ifelse fillingLevel > 0
 [
   set handlingTime_s [ interFlowerTime_s ] of Foodsource myPatch ; the time to travel
                                                                  ; to the next flower
                       * flowersNeededForPollenLoad    ; times the number of flowers
                                                       ; needed to be visited
                       / fillingLevel              ; divided by the filling level
                                                   ; to account for depletion of the patch
                    + timeInFlowers_s       ; plus the time to actually collect the
                                            ; pollen, once a flower with pollen is found

 ]
 [ set handlingTime_s  maxHandlingTime_s ]

 ] ; end: if pollen forager

if handlingTime_s > maxHandlingTime_s [ set handlingTime_s maxHandlingTime_s ]
report handlingTime_s
end
```

Foraging_unloadingProc

Purpose: bees store their nectar or pollen loads in the *colony*

Called by: *ForagingProc*

Asking agents: *Bees* (workers and non-hibernating queens)

Calling: none

Description

Successful nectar foragers (i.e. *bees* with *activity* "expForagingN" or "returningUnhappyN") transfer the energy they are carrying to a *colony's* nectar stores (*energyStore_kJ*), successful pollen foragers (i.e. *bees* with *activity* "expForagingP" or "returningUnhappyP") add their load to the *colony's* pollen store (*pollenStore_g*). The loads of the *bees* is then set to 0 and their *activityList* and personal time are updated:

```
to Foraging_unloadingProc
  ; successful foragers (irrespective whether they are happy or not) unload
  ; their nectar or pollen load
  ifelse activity = "expForagingN" or activity = "expForagingP"
       or activity = "returningUnhappyN" or activity = "returningUnhappyP"
  [
    let nectarIncrease nectarLoadSquadron_kJ
    let pollenIncrease pollenLoadSquadron_g

    ask Colony colonyID    ; load is added to the colony's stores:
    [
     set energyStore_kJ energyStore_kJ + nectarIncrease
     set pollenStore_g pollenStore_g + pollenIncrease
    ]
    ifelse activity = "expForagingN" or activity = "returningUnhappyN"
      [  set activityList lput (word "N+:" precision nectarIncrease 2) activityList ]
      [  set activityList lput (word "P+:" precision pollenIncrease 4) activityList ]
    set nectarLoadSquadron_kJ 0
    set pollenLoadSquadron_g 0
    set personalTime_s personalTime_s + [ timeUnloading ] of OneSpecies speciesID
  ]
  [ ; make sure bees with other activities don't carry nectar or pollen:
    if nectarLoadSquadron_kJ + pollenLoadSquadron_g > 0
      [ AssertionProc "Bee did not unload nectar or pollen in Foraging_unloadingProc!"
  ]
 ]
end
```

QueensLeavingNestProc

Purpose: young queens leave the *colony*, mate and hibernate

Called by: *Go*

Asking agents: none

Calling: *DieProc*

Description

Young, adult queens that haven't left their *colony* yet (i.e. *bees* with *stage* "adult", *caste* "queen", mated? false and *colonyID* not set to -1) leave the *colony* to mate with a single male and then hibernate immediately.
The young queen mates with a single, randomly chosen adult, haploid or diploid male of the same *species* and saves his *allelesList* in her *spermathecaList* (Note that in bumblebees (unlike e.g. honeybees), diploid male brood can develop into adults (Duchateau et al. 1994), however, queens mated with a diploid male are not able to establish a colony (Duchateau & Marien 1995). We hence remove queens mated with diploid males during hibernation (in *QueensLeavingNestProc*)). If no adult males are available, the queen still mates (if the switch *UnlimitedMales?* is set true (default)), but her *spermathecaList* is set to a random, negative integer number between -1 and -*nForeignAlleles*, representing a male from outside the simulated world. The *queen's colonyID* is set to -1 as she has left her mother *colony* and hasn't founded her own *colony* so is currently not a member of any *colony*. *Mated?* is set true and *activity* becomes "hibernate", i.e. she won't be involved in any activities until she emerges next spring. If *UnlimitedMales?* is false queens die if no males are available for mating on that day.

```
to QueensLeavingNestProc
 ; young queens leave the colony, mate and hibernate:
  ask Bees with [ stage = "adult" and caste = "queen" ; young (unmated), adult queens
  and mated? = false and colonyID != -1 ]             ; still in a colony..
  [
    let memoSpecies speciesID
    ifelse count bees with [ caste = "male" and stage = "adult"
               and speciesID = memoSpecies ] > 0   ; if suitable males are present..
     [
      let newAlleleList []  ; .. the queen will mate with one..

      ask one-of bees with [ caste = "male" and stage = "adult"
                             and speciesID = memoSpecies ] ;   mating with a haploid
                             ; or diploid(!) adult male of the same species,
        [ set newAlleleList allelesList ]   ;
      set spermathecaList newAlleleList ; male alleles are saved in the spermatheca
     ]
     [ ; if no males present queen mates with a male from "outside":
      if UnlimitedMales? = false [ DieProc "Queen: no mating" ]
                                ; if queen's can't mate, they are removed
      let foreignAllele -1 * (random N_ForeignAlleles) - 1
               ; random integer number: -1, -2, ... -N_ForeignAlleles
      set spermathecaList fput foreignAllele spermathecaList
      if length spermathecaList > 1
       [ AssertionProc "Assertion violated in QueensLeavingNestProc:
                        too many alleles here!" ]
     ]
    set mated? true    ; queen is now mated
    set thEgglaying ThresholdLevelREP "eggLaying" "QueenInitiationPhase"
       ; queen is now ready to lay eggs (though she won't before hibernation!)
    set size QueenSymbolSize
    set shape "circle"
    set color red
    set activity "hibernate"
       ; queen hibernates and be active untile she emerges in spring
    set colonyID -1   ; queen is no longer member of a colony
    if length spermathecaList = 2 [ DieProc "Queen: mating with diploid male" ]
      ; queens mating with diploid male are removed from the simulation as they are
      ; not able to establish a colony (Duchateau & Marien 1995)
  ]
end
```

FeedLarvaeProc

Purpose: determines how much nectar and pollen is fed to larvae in each *colony*, calculates the resulting weight gain of the larvae and updates the *colony* stores

Called by: *Go*

Asking agents: none

Calling: *MaxWeightGainToday_mg_REP*

Description

We assume that larvae have a diet that balances the intake of protein (pollen) and carbohydrate (nectar) (e.g. Simpson & Raubenheimer 2006, Pirk et al. 2010, Stabler et al. 2015). We first calculate the pollen consumption to determine the weight gain of a larva and then derive the amount of energy required to assimilate the consumed proteins.

The procedure is called after all of today's foraging has been taking place and before the development of brood and adults.
First the relative amounts of nectar and pollen that the worker *bees* are prepared to feed to the brood (*relativePollenToBeFed*, *relativeEnergyToBeFed*) is calculated by dividing actual nectar and pollen store by the "ideal" nectar and pollen stores of the *colony* (*idealPollenStore_g*, *idealEnergyStore_kJ*). These "ideal" values are calculated in the reporter procedures *StimForagingNectarREP* and *StimForagingPollenREP* and are based on the amount of food that is approximately required within a certain number of days. The resulting "relative" amounts of food to be fed have to be between 0 and 1.

```
to FeedLarvaeProc
  ask colonies
  [

   let myColony who
   ; RELATIVE AMOUNTS TO BE FED:
   let relativePollenToBeFed 0 ; may be updated below
   let relativeEnergyToBeFed 0

   ; This will be set based on how large the stores are relative to the ideal stores
   ; (these have already been filled through foraging today)
   if idealPollenStore_g * idealEnergyStore_kJ > 0   ; i.e. if both > 0
   [
     set relativePollenToBeFed pollenStore_g / idealPollenStore_g
     set relativeEnergyToBeFed energyStore_kJ / idealEnergyStore_kJ
      ; set values to be bound by 0 1. Added bound by 0 because values can be lower
      ; if the energyStore is negative (this is okay, because
      ; the colony will die at the start of the next tick). Negative values lead to
      ; energy being taken from the larvae and added back to the store.
     set relativePollenToBeFed median (list 0 1 relativePollenToBeFed)
     set relativeEnergyToBeFed median (list 0 1 relativeEnergyToBeFed)

   ]
```

As pollen and nectar consumption of the larvae are not independent of each other, we define the limiting factor (*growthLimitingFactor*) as *relativePollenToBeFed* or *relativeEnergyToBeFed*, whatever is smaller.

```
   ; So set the growth limiting factor as the lowest of either relativeEnergy
   ; or relativePollen based on Liebig's law of the minimum, larval growth is assumed
   ; to be limited by only one factor:
   let growthLimitingFactor relativePollenToBeFed
   if relativeEnergyToBeFed < relativePollenToBeFed  ; amount of nectar fed is adjusted to
                                                     ; the amount of pollen fed
     [ set growthLimitingFactor relativeEnergyToBeFed ]
```

The actual amount of pollen fed to an individual larva is then the amount of pollen it would need for maximal growth multiplied by *growthLimitingFactor*. This maximal weight gain is calculated in the reporter-procedure *MaxWeightGainToday_mg_REP* and depends on the larva's current weight and a *species*-specific factor that describes how efficient a *bee* is to transform pollen into body mass (*pollenToBodymassFactor*):

```
    ; ACTUAL FEEDING OF EACH INDIVIDUAL LARVA:
    let totalPollenFedToday_g 0  ; sums up the total amount of pollen a colony feeds
                                 ; to the larvae
    ask bees with [ stage = "larva" and colonyID = myColony ]
    [
      ; Calculate pollen gained based on conversion to max weight gain adjusted by
      ; limiting factor
      ; amount of pollen fed to a single larva (no "number" here as it refers to amount
      ; an individual larva gets)
      let pollenReceivedToday_mg growthLimitingFactor * ((MaxWeightGainToday_mg_REP who)
                               / ([pollenToBodymassFactor] of OneSpecies speciesID))

    if pollenReceivedToday_mg > [ pollenStore_g ] of colony myColony * 1000
        ; to avoid negative pollen stores
        [
          set pollenReceivedToday_mg [ pollenStore_g ] of colony myColony * 1000
          if pollenReceivedToday_mg < 0 [ set pollenReceivedToday_mg 0 ] ; in case of negative
                                                ; pollen stores, larvae are not fed at all!
        ]
```

Then the weight of the larva is increased, according to the amount of pollen consumed and its *pollenToBodymassFactor*. The amount of pollen consumed in the *colony* is summed up, taking the cohort size (*number*) of each agent representing a larva into account:

```
      ; Update the larva's weight
      let oldWeight_mg weight_mg
      set weight_mg weight_mg + pollenReceivedToday_mg
                        * [pollenToBodymassFactor] of OneSpecies speciesID
      if weight_mg < 0 [ AssertionProc "BUG in FeedPOLLENProc" ]
      if weight_mg < oldWeight_mg [ AssertionProc "BUG in FeedPOLLENProc: WeightLoss" ]
      ; Update the total pollen to be taken from the store by the number of indivuals
      ; in the cohort
      set totalPollenFedToday_g totalPollenFedToday_g
                          + (number * ((weight_mg - oldWeight_mg)
                          / [pollenToBodymassFactor] of OneSpecies speciesID))
                          / 1000
        ; multiplied by "number" here as it refers to the total costs for the colony
    ]
```

Finally, it is calculated how much energy is required by the larvae to assimilate to pollen they have consumed and then the colonies pollen and nectar stores are updated:

```
    ; Update the total energy required to assimilate th pollen consumed:
    let totalEnergyFedToday_kJ totalPollenFedToday_g
                          * EnergyRequiredForPollenAssimilation_kJ_per_g
    ; REMOVING RESOURCES FROM THE STORE
    set pollenStore_g pollenStore_g - totalPollenFedToday_g
    if pollenStore_g < 0 [ type "negative pollen store! Ticks: " show ticks ]
    set energyStore_kJ energyStore_kJ - totalEnergyFedToday_kJ
    ; (negative energy store wouldn't matter as it results in the death
    ; of the colony the next morning (in UpdateColoniesProc))
  ]
  ]
end
```

QueenProductionDateProc

Purpose: determines for each *colony* the date when it starts to produce queens

Called by: *Go*

Asking agents: none

Calling: none

Description

The procedure determines if the *colony* variable *queenProduction?* is set true and - if this is the case - calculates the date (*queenProductionDate*) when the first eggs destined to become queens (may) have been laid. Once *queenProduction?* is true, female larvae of a certain age (*dev_larvalAge_QueenDetermination_d*) may develop into queens instead of workers. As *dev_larvalAge_QueenDetermination_d* refers only to the time a *bee* spends as larva and does not take the egg period into account, these larvae may differ in their *broodAge*. Following Duchateau & Velthuis 1988, we assume that queen-destined eggs are not laid earlier than *QueenDestinedEggsBeforeSP_d* (5d) before the *colony*'s switch point (*switchPointDate*). If today is the first time step when female larvae may develop into queens instead of workers (i.e. if *queenProduction?* is going to be set from *false* to *true* today), then the day when the first batch of queen-destined eggs have been laid is today's time step minus the age of these larvae. If actually larvae of the right larval-age are present today their age (*broodAge*) can be directly accessed and *queenProductionDate* can be accurately calculated. However, to determine *queenProductionDate* even if no larvae of the right age are present, an average development time for eggs (*averageCumulTimeEgg_d*) needs to be assumed and *timeEggToLarvalAgeAtQueenDetermination* is then calculated as *averageCumulTimeEgg_d* plus *dev_larvalAge_QueenDetermination_d*. Hence, *queenProduction?* may be set true, if today's time step (*ticks*) minus *timeEggToLarvalAgeAtQueenDetermination* is larger or equal the *colony*'s switch point date (*switchPointDate*) minus *QueenDestinedEggsBeforeSP_d*.

However, also sufficient worker *bees* relative to the the number of larvae need to be present in the *colony* (i.e. *larvaWorkerRatio* has to be smaller than *LarvaWorkerRatioTH* (set to 3) to allow queen production. Only if both criteria are fullfilled, *queenProduction?* is set true, and *queenProductionDate* is set to the date when these queen-destined eggs have been laid.

```
to QueenProductionDateProc
  ; for B. terrestris, based on Duchateau & Velthuis 1988 - no data for other species!
  ask colonies
  [
    let memoColony who
    let averageCumulTimeEgg_d 7 ; average duration of egg phase (in the model!): 6-7d
                                ; set to 7 as this results in better sex ratio
    let timeEggToLarvalAgeAtQueenDetermination        ; time as egg + time as larvae
            averageCumulTimeEgg_d
            + [ dev_larvalAge_QueenDetermination_d ] of OneSpecies speciesIDcolony ;  i.e.
                                                     ; ca. 7+3=10d for B. terrestris
            ; (only) if larvae of the right age are present,
            ; timeEggToLarvalAgeAtQueenDetermination can be directly determined from their
            ; brood age (in this case, the previous value is overwritten):
    if any? bees with [ stage = "larva"
                        and colonyID = memoColony
                        and cumulTimeLarva_d = [ dev_larvalAge_QueenDetermination_d ]
                                             of OneSpecies speciesID ]
       [ set timeEggToLarvalAgeAtQueenDetermination
               max [ broodAge ] of bees
                    with [ stage = "larva"
                    and colonyID = memoColony
                    and cumulTimeLarva_d = [ dev_larvalAge_QueenDetermination_d ]
                                         of OneSpecies speciesID ]]
             ; asking for "max" in case there are 2 larval cohorts of
             ; dev_larvalAge_QueenDetermination_d age but different broodAges
             ; (because younger cohort has developed quicker as eggs).
             ; This should not happen in the current version,
             ; but might be the case in a future version.

      if queenProduction? = false
       and ticks - timeEggToLarvalAgeAtQueenDetermination  ; this is the date when
                             ; the larvae which are today at the queen determination stage
                             ; were laid as eggs
           >= switchPointDate - QueenDestinedEggsBeforeSP_d  ; "queen eggs" are laid
                     ; QueenDestinedEggsBeforeSP_d (5d) before switchpoint at earliest
       and larvaWorkerRatio < LarvaWorkerRatioTH
         ; ..but also the L:W ratio on that day has to be below LarvaWorkerRatioTH (= 3)
      [
        set queenProduction? true ; female larvae can now develop into queens
        set queenProductionDate ticks - timeEggToLarvalAgeAtQueenDetermination ; ..these
                                ; larvae were laid (as eggs) on the day queenProductionDate
      ]
```

```
  ]
end
```

DevelopmentProc

Purpose: ageing and development of brood stages and adults

Called by: *Go*

Asking agents: none

Calling:
Development_Mortality_AdultsProc
Development_PupaeProc
Development_LarvaeProc
Development_EggsProc

Description
The *bees* age by one day (increasing *broodAge* for brood or *adultAge* for adults) and their graphic representations on the interface move one step to the right. Details of the development are covered by sub-procedures, depending on the developmental stage of the *bee*:
Adults bees undergo a behavioural development in *Development_Mortality_AdultsProc*, pupae are dealt with in *Development_PupaeProc*, larvae in *Development_LarvaeProc* and eggs in *Development_EggsProc*).

```
to DevelopmentProc
 ask bees
 [
   let whoCol colonyID
   ifelse stage = "adult"
    [
      set adultAge adultAge + 1
      if adultAge > 700 [AssertionProc "Assertion violated: Bee with 2 hibernations!"]
      if brood? = true [AssertionProc "Assertion violated (DevelopmentProc)" ]
      Development_Mortality_AdultsProc
    ]
    [
      set broodAge broodAge + 1
      if brood? = false  [ "Assertion violated(DevelopmentProc)"]
      if stage =  "pupa" [ Development_PupaeProc ]
      if stage = "larva" [ Development_LarvaeProc whoCol ]
      if stage =  "egg"  [ Development_EggsProc ]
    ]

  if xcor + StepWidth < max-pxcor and mated? = false
       ; move graphic representation of bees on GUI
       [ set xcor xcor + StepWidth ]
 ]
end
```

Development_Mortality_AdultsProc

Purpose: Worker bees die whenreaching their maximal age and queens update the *activity* thresholds when the *colony* enters the social phase.

Called by: *DevelopmentProc*

Asking agents: *bees* (with *stage* = "adult")

Calling:

DieProc
ThresholdLevelREP

<u>Description</u>

Adult worker *bees* and males die, when they reach their maximal age (*MaxLifespanWorkers* , *MaxLifespanMales*). The thresholds for nectar foraging, pollen foraging, nursing and egg laying of mother queens change, when their *colony* enters the social phase. The new thresholds are set in *ThresholdLevelREP*. A daily mortality risk (*MortalityAdultsBackground_daily*) can be applied to all adult *bees*, but under default conditions its value is set to 0, as adult mortality within the nest is negligible in real bumblebees (Plowright and Jay 1968) and adult mortality outside the nest is addressed elsewhere in the model.

```
to Development_Mortality_AdultsProc
 if caste = "worker" ; behavioural development workers
 [
   if adultAge > [ maxLifespanWorkers ] of OneSpecies speciesID
       [ DieProc "Worker: adultAge > maxLifespanWorkers" ]
 ]

 if caste = "male" ; death of adult males after max lifespan
 [
   if adultAge > MaxLifespanMales [ DieProc "Male: adultAge > MaxLifespanMales" ]
 ]

 if caste = "queen" and mated? = true and colonyID >= 0
    and [ allAdultWorkers ] of colony colonyID > 0 ; if colony is in the social phase
 [
    set thForagingNectar ThresholdLevelREP "nectarForaging" "QueenSocialPhase"
    set thForagingPollen ThresholdLevelREP "pollenForaging" "QueenSocialPhase"
    set thNursing ThresholdLevelREP "nursing" "QueenSocialPhase"
    set thEgglaying ThresholdLevelREP "eggLaying" "QueenSocialPhase"
 ]
 if  MortalityAdultsBackground_daily > 0 ; MortalityAdultsBackground_daily = 0
    and random-float 1 > MortalityAdultsBackground_daily
    [ DieProc "Adult bee: mortality in colony" ]
end
```

Development_PupaeProc

Purpose: determines if pupae develop into adults and emerge

Called by: *DevelopmentProc*

Asking agents: *bees* (with *stage* = "pupa")

Calling:

CropAndPelletSizeREP
ThresholdLevelREP
ProboscisLengthREP

<u>Description</u>

The procedure addresses bees separately with *caste* = "worker", "male" or "queen". If the *caste* specific criteria to develop into adults are fulfilled (i.e. pupae have a certain age and sufficient incubation received (*devAgeEmergingMin_d* and *devIncubationEmergingTH_kJ* for workers and males, or *devAge_Q_EmergingMin_d* and *devIncubation_Q_EmergingTH_kJ* for queens), then they develop into adults. In this case, *stage* is set to "adult", *brood?* to false, and the thresholds for the four tasks are re-set (*ThresholdLevelREP*) in workers and queens.
The proboscis length (*glossaLength_mm*) is determined in *ProboscisLengthREP* and *cropvolume_myl* and *pollenPellets_g* in *CropAndPelletSizeREP*.

```
to Development_PupaeProc ; procedure checks if pupae develop into adults

  set cumulTimePupa_d cumulTimePupa_d + 1  ;  ; potential ouput (time spent as pupa)
  if caste = "worker"
  [
    ; Development factors pupae: age & incubation:
    if cumulIncubationReceived_kJ >= [ devIncubationEmergingTH_kJ ]
                                       of OneSpecies speciesID
         and broodAge >= [ devAgeEmergingMin_d ] of OneSpecies speciesID
         [
           set stage "adult"
           set brood? false
           set color black
           let newWorkers number ; saves the cohort size
           set TotalAdultsEverProduced TotalAdultsEverProduced + number
           ask colony colonyID [set totalAdultsProduced  totalAdultsProduced
                                                        + newWorkers]
           ask colony colonyID [set totalWorkersProduced  totalWorkersProduced
                                                        + newWorkers]
           set cropvolume_myl    CropAndPelletSizeREP "nectar"
           set pollenPellets_g   CropAndPelletSizeREP "pollen"
           set thEgglaying       ThresholdLevelREP "eggLaying" "worker"
           set thForagingNectar ThresholdLevelREP "nectarForaging" "worker"
           set thForagingPollen ThresholdLevelREP "pollenForaging" "worker"
           set thNursing         ThresholdLevelREP "nursing" "worker"
           set glossaLength_mm   ProboscisLengthREP
         ]
  ]
```

Similarly for males, but no crop volume, size of pollen pellets or proboscis length are calculated:

```
  ; NOTE: in bumblebees (B. terrestris) diplod males develop into (sterile)
  ; adults (Duchateau et al. 1994)
  ;  (dipl. males can also mate but these queens are not able to establish a colony
  ;(Duchateau & Marien 1995) and are removed (in QueensLeavingNestProc)
  if caste = "male" ; MALE PUPAE - might develop into adult males
  [
     if cumulIncubationReceived_kJ >= [ devIncubationEmergingTH_kJ ]
                                       of OneSpecies speciesID
          and broodAge >= [ devAgeEmergingMin_d ] of OneSpecies speciesID
          [
             set brood? false
             set stage "adult"
             set color green
             let newMales number
             set TotalAdultsEverProduced TotalAdultsEverProduced + number
             set TotalAdultMalesEverProduced TotalAdultMalesEverProduced + number
             ask colony colonyID
             [
               set totalAdultsProduced totalAdultsProduced + newMales
               set totalMalesProduced  totalMalesProduced + newMales
             ]
          ]
   ]
```

For queens, the date of emergence from hibernation (*emergingDate*) is determined randomly around a *species*-specific mean (*emergingDay_mean*) ± standard deviation (*emergingDay_sd*) but be within the coming season.
The global variable *QueensProducingColoniesList* keeps track of all colonies that ever produced queens and is updated, as well as the statistics of the *bee*'s *colony* (*totalQueensProduced, totalAdultsProduced*).

```
if caste = "queen" ; QUEEN PUPAE - might develop into adult queens
[
   if cumulIncubationReceived_kJ >= [ devIncubation_Q_EmergingTH_kJ ]
                                    of OneSpecies speciesID
      and broodAge >= [ devAge_Q_EmergingMin_d ] of OneSpecies speciesID
        [
         let yearEndSeason (365 * ceiling (ticks / 365))
             + [seasonStop] of OneSpecies speciesID ; prevent bees from setting
                                   ; emergingDate past the end of season
         let yearStartSeason (365 * ceiling (ticks / 365))
             ;start season is 365 * the current year
         while [ emergingDate <= yearStartSeason OR emergingDate > yearEndSeason]
                     ; add start season to the while statement
           [ set emergingDate  (365 * ceiling (ticks / 365)) ; emerging from
             ; hibernation next year on day "emergingDay_mean" (+- s.d.)
              + round random-normal [ emergingDay_mean ] of OneSpecies speciesID
                               [emergingDay_sd]  of  OneSpecies  speciesID]  ;  SD

         set stage "adult"
         set brood? false
         set color red

         let newQueensProduced number
         if not member? colonyID QueensProducingColoniesList
           [ set QueensProducingColoniesList
                     lput colonyID QueensProducingColoniesList ]
         set TotalAdultsEverProduced TotalAdultsEverProduced + number
         set TotalAdultQueensEverProduced TotalAdultQueensEverProduced + number
         ask colony colonyID
         [
           set totalQueensProduced totalQueensProduced + newQueensProduced
           set totalAdultsProduced  totalAdultsProduced + newQueensProduced
         ]
          set cropvolume_myl   CropAndPelletSizeREP "nectar"
          set pollenPellets_g  CropAndPelletSizeREP "pollen"
          set thEgglaying      ThresholdLevelREP "eggLaying" "youngQueen"
          set thForagingNectar ThresholdLevelREP "nectarForaging" "youngQueen"
          set thForagingPollen ThresholdLevelREP "pollenForaging" "youngQueen"
          set thNursing        ThresholdLevelREP "nursing" "youngQueen"
          set glossaLength_mm ProboscisLengthREP
        ]
 ]

 if caste = "undefined"
   [ AssertionProc "Assertion violated: undefined caste! (Development_PupaeProc)" ]
end
```

ProboscisLengthREP

Purpose: calculate the length of the proboscis from the weight of the bee

Asking agents: *bees*

Called by: *CreateInitialQueensProc*, *Development_PupaeProc*

Calling: none

Input: none

Description

The length [mm] of a proboscis is calculated from a *bee's* weight [mg], assuming a linear relationship between weight and proboscis length. Minimal and maximal weights (*devWeightPupationMin_mg*, *devWeight_Q_PupationMax_mg*) and minimal and maximal proboscis lengths (*proboscis_min_mm*, *proboscis_max_mm*) are *species*-specific parameters (note that the minimal weight refers to a worker *bee* but the maximal weight to a queen). The *slope* is calculated as the difference between maximal and minimal proboscis length divided

by the difference of maximal and minimal weight. The actual proboscis length is then the minimal length plus the difference between actual bee weight and minimal weight times the *slope*.

```
to-report ProboscisLengthREP
  let minWeight_mg [ devWeightPupationMin_mg ] of oneSpecies speciesID
  let maxWeight_mg [ devWeight_Q_PupationMax_mg ] of oneSpecies speciesID
  let minLength_mm [ proboscis_min_mm ] of oneSpecies speciesID
  let maxLength_mm [ proboscis_max_mm ] of oneSpecies speciesID
  let slope (maxLength_mm - minLength_mm) / (maxWeight_mg - minWeight_mg)
  let proboscisLength_mm minLength_mm + (weight_mg - minWeight_mg) * slope
  if weight_mg < minWeight_mg or weight_mg > maxWeight_mg
       [ AssertionProc ("Wrong bee weight in ProboscisLengthREP Min") ]
  report proboscisLength_mm
end
```

Development_LarvaeProc

Purpose: determines if larvae develop into a pupae

Called by: *DevelopmentProc*

Asking agents: *bees* (with *stage* = "larva")

Calling: *DetermineCaste_REP*

Input: *whoCol* (*who* of the calling *bee's colony*)

Description

Larvae increase the duration of their larval age (*cumulTimeLarva_d*) by one day. If their larval age reaches *dev_larvalAge_QueenDetermination_d* and their *caste* is still "undefined", the reporter-procedure *DetermineCaste_REP* is called to determine on basis of the *larva*'s weight and the situation of its *colony* whether it develops into a worker or a queen.
If a *larva* meets all the *caste* and *species*-specific requirements for pupation (incubation received, age and weight), *stage* is set "pupa" and *totalPupaeProduced* is updated by the cohort size of the *bee*.

```
to Development_LarvaeProc [ whoCol ]
  ; procedure checks if larvae develop into pupa.  Development factors larva: age,
  ; incubation and weight - option to develop into queen!

  set cumulTimeLarva_d cumulTimeLarva_d + 1 ; time spent as larva is increased by 1d
  if caste = "undefined" and cumulTimeLarva_d = [ dev_larvalAge_QueenDetermination_d ]
                                     of OneSpecies speciesID ; age of determination is
                                                 ; independent of of time spent as egg

  [
    set caste DetermineCaste_REP whoCol ; this reporter-procedure determines the caste
    if caste = "queen" [ set color orange ]
    if caste = "undefined"
       [AssertionProc "Assertion violated: undefined caste! (Development_LarvaeProc)"]
  ]

  if caste = "worker" or caste = "male"  ; larvae develop into pupae as soon as they
     ; 1) received enough incubation, and 2) they are old enough and 3.) heavy enough
     and cumulIncubationReceived_kJ >= [ devIncubationPupationTH_kJ ]
                                              of OneSpecies speciesID
     and broodAge >= [ devAgePupationMin_d ] of OneSpecies speciesID
     and weight_mg >= [ devWeightPupationMin_mg ] of OneSpecies speciesID
  [
```

```
    set stage "pupa"
    set color brown
    if ploidy = 1 [ set color grey - 2 ]
    let memoNumber number
    ask colony colonyID [set totalPupaeProduced  totalPupaeProduced + memoNumber]
  ]

  if caste = "queen" ;
     and cumulIncubationReceived_kJ >= [ devIncubation_Q_PupationTH_kJ ]
                                        of OneSpecies speciesID
     and broodAge >= [ devAge_Q_PupationMin_d ] of OneSpecies speciesID
     and weight_mg >= [ devWeight_Q_PupationMin_mg ] of OneSpecies speciesID
  [
    set stage "pupa"
    set color red
    let memoNumber number
    ask colony colonyID [set totalPupaeProduced  totalPupaeProduced + memoNumber]
  ]

end
```

DetermineCaste_REP

Purpose: determines if a female larvae develops into a worker or a queen

Asking agents: *bees* (with *stage* = "larva" and caste = "undefined")

Called by: *Development_LarvaeProc*

Input: *whoCol*

Description

Female larvae develop into workers, unless both an individual and a *colony* criterion to develop into a queen, are fulfilled: 1) the larvae needs to have a certain, minimal weight (*dev_Q_DeterminationWeight_mg*) and the *colony* needs to be ready for queen production (i.e. *queenProduction?* is true). (Note that *dev_Q_DeterminationWeight_mg* is set to 0 (i.e. is always fulfilled) as, at least in *B. terrestris*, increased feeding is not the cause but the consequence of a larvae developing into a queen (Pereboom et al. 2003) while for other *species* we are lacking data).

```
to-report DetermineCaste_REP [ whoCol ]
 let mycaste "worker" ; bee will develop into a worker, unless it becomes a queen
 ; it will be a queen if individual weight (1) and colony conditions (2) for becoming
 ; a queen are both fulfilled:
 if (weight_mg >= [ dev_Q_DeterminationWeight_mg ] of OneSpecies speciesID ; (1)
   and [ queenProduction? ] of colony whoCol = true)                       ; (2)
   [ set mycaste "queen" ]
 report mycaste
end
```

Development_EggsProc

Purpose: determines if eggs develop into a larvae

Called by: *DevelopmentProc*

Asking agents: *bees* (with *stage* = "egg")

Calling: none

<u>Description</u>

A larva hatches from an egg (i.e. *stage* is set to "larva") when it has received at least a certain *species*-specific amount of energy from incubation (*devIncubationHatchingTH_kJ*) and is of *devAgeHatchingMin_d* days age or older. The *colony* variable *totalLarvaeProduced* keeps track of all larvae produced in this *colony* as a potential output of simulation runs.

```
to Development_EggsProc  ;  procedure checks if eggs develop into larvae
 if cumulIncubationReceived_kJ >= [ devIncubationHatchingTH_kJ ] of OneSpecies speciesID
    and broodAge >= [ devAgeHatchingMin_d ] of OneSpecies speciesID
    [
      set stage "larva"
      set color white
      if ploidy = 1 [ set color yellow ]  ; male larvae are represented by yellow bars
                                          ; on the interface
      let memoNumber number
      ask colony colonyID [set totalLarvaeProduced  totalLarvaeProduced + memoNumber]
    ]
end
```

MortalityBroodProc

Purpose: determines mortality of brood stages

Called by: *Go*

Asking agents: none

Calling: *DieProc*

<u>Description</u>

If brood does not develop into the next stage within a certain time frame (e.g. because of lack of incubation) it dies.
The *species*-specific maximal ages are: for eggs: *devAgeHatchingMax_d*, for larvae *devAgeEmergingMax_d* (or *devAge_Q_EmergingMax_d* for queen larvae) and *devAgeEmergingMax_d* for pupae (or *devAge_Q_EmergingMax_d* for queen pupae). We assume that worker and male brood show a similar development (e.g. Cnaani et al. 2002 for *B. impatiens*).
Eggs die, if their age (*broodAge*) exceeds the *species*-specific maximal age for hatching (*devAgeHatchingMax_d*):

```
to MortalityBroodProc

 ask bees
 [
   let memoNumber number

   if stage = "egg" and broodAge > [ devAgeHatchingMax_d ] of OneSpecies speciesID
   [
     ask colony colonyID [set eggDeathsIncubation  eggDeathsIncubation + memoNumber]
     DieProc "Egg: broodAge  > devAgeHatchingMax_d"
```

```
        ]
```

Queen larvae die, if they are older than *devAge_Q_PupationMax_d*, all other larvae die after the age of *devAgePupationMax_d*. To determine the cause of larval mortality as a potential output of a simulation run, the *larvae*'s relative weight and relative amount of incubation it has received is calculated, with the lower one of both being the cause of its death. The number of larvae died due to insufficient weight (*larvaDeathsWeight*) or insufficient incubation (*larvaDeathsIncubation*) is tracked by the *colonies*.

```
if stage = "larva"
  [
   if ((caste = "worker" or caste = "male" or caste = "undefined")
                  and  broodAge > [ devAgePupationMax_d] of OneSpecies speciesID )
        or (caste = "queen" and  broodAge > [ devAge_Q_PupationMax_d]
                                              of OneSpecies speciesID )
    [
     ; Get relative incubation and weights (relative to minimum target required
           ; for developing into the next stage).
     let relativeIncub  (cumulIncubationReceived_kJ
                          / [ devIncubationPupationTH_kJ ] of OneSpecies speciesID)
     let relativeWeight -1
     ifelse caste = "queen"
       [
        set relativeWeight (weight_mg / [ devWeight_Q_PupationMin_mg ]
                                              of OneSpecies speciesID)
        set relativeIncub  (cumulIncubationReceived_kJ
                  / [ devIncubation_Q_PupationTH_kJ ] of OneSpecies speciesID)
       ]
       [
        set relativeWeight (weight_mg / [ devWeightPupationMin_mg ]
                                              of OneSpecies speciesID)
        set relativeIncub  (cumulIncubationReceived_kJ
                        / [ devIncubationPupationTH_kJ ] of OneSpecies speciesID)
       ]
     ; Record the outputs: number of bees that die due to
    ; relative weight/incubation received is less than 1
     if relativeWeight < 1 AND relativeWeight < relativeIncub
      [ ask colony colonyID
         [set larvaDeathsWeight  larvaDeathsWeight + memoNumber]
      ]
     if relativeIncub  < 1 AND relativeIncub < relativeWeight
      [ ask colony colonyID
         [set larvaDeathsIncubation  larvaDeathsIncubation + memoNumber] ]
     if relativeWeight >= 1 AND relativeIncub >= 1 [assertionProc "Neither Weight
                          or Incubation reason for death: MortalityBroodProc (1)"]
     DieProc "Larva: broodAge  > max. pupation age"
    ]
  ]
```

Worker or male pupae die, if their age is above *devAgeEmergingMax_d* and queen pupae die if they are older than *devAge_Q_EmergingMax_d* (there are no pupae with "undefined" *caste*). Again, the causes of pupal deaths (*pupaDeathsWeight*, *pupaDeathsIncubation*) are tracked by the colonies:

```
if stage = "pupa"
  [
    if caste = "undefined"
            [ assertionProc "Pupa with undefined caste (MortalityProc)!" ]
    if ((caste = "worker" or caste = "male")
            and  broodAge  > [ devAgeEmergingMax_d ] of OneSpecies speciesID )
       or (caste = "queen" and  broodAge  > [ devAge_Q_EmergingMax_d ]
                                      of OneSpecies speciesID )
    [
     ask colony colonyID ;as pupae are not fed, they died due to lack of incubation
       [ set pupaDeathsIncubation  pupaDeathsIncubation + memoNumber ]
     DieProc "Pupa: broodAge  > max. emerging age"
    ]
  ]
```

After a *colony* passed its competition point we assume that all eggs die as they are consumed by worker bees (Duchateau & Velthuis 1988):

```
  if colonyID >= 0 and ticks > [ competitionPointDate ] of Colony colonyID
; development of eggs into larvae only possible before CP! (Duchateau & Velthuis 1988)
  [
    if stage = "egg"
```

```
          [
            ask colony colonyID [set broodDeathsCP  broodDeathsCP + memoNumber]
            DieProc "Egg: CP!"
          ]
      ]
    ]
  end
```

BadgersOnTheProwlProc

Purpose: determines if a *colony* is dug up and killed by a badger

Called by: *Go*

Asking agents: none

Calling: *DieProc*

<u>Description</u>
Bumblebee *colonies* within the home range (*foragingRange_m*) of a badger may be destroyed and all bees killed. The probability for this to happen is calculated from the daily probability a badger comes across the nest (*encounterProb*) and the probability that the nest is then detected an dug up (digUpProb) by the badger. The nectar and pollen stores of the *colony* are then set to 0 and all *bees* die:

```
to BadgersOnTheProwlProc
  let foragingRange_m 735 ; estimated from Kruuk & Parish, J.Zool.,Lond.(1982) 196,31-39
                          ; Tab. 1: territory: ca. 170ha, hence radius ca. 735m
  let encounterProb  0.19 ; probability to come across the nest; Kowalczyk et al 2006,
      ; Wildlife Biology 12(4):385-391. 2006
      ; Tab1, DR% (daily range as % of total home range: 19+-18;
  let digUpProb 0.1 ; probability to perceive nest and dig it up - ARBITRARY VALUE
  ask Badgers
  [
   let memoX xcor
   let memoY ycor
   ask colonies with [ distancexy memoX memoY < SCALING_NLpatches/m * foragingRange_m ]
   [
     if random-float 1 < encounterProb * digUpProb
     [

       set energyStore_kJ 0
       set pollenStore_g 0
       set color red
       let victimColonyID who
       let memobroodDeaths 0
       ask bees with [ colonyID = victimColonyID ]
       [

         if brood? [set memobroodDeaths memobroodDeaths + number]
         DieProc "Colony killed by badger!"
       ]
       set broodDeathBadger broodDeathBadger + memobroodDeaths
     ]
   ]
  ]
end
```

OutputDailyProc

Purpose: Updates plots and weather symbols

Called by: *Go*

Asking agents: none

Calling: *PlottingProc*

Description

Some global variables keeping track of the number of queens, *foodsources*, *colonies* etc. to be shown on NetLogo "monitors" on the interface are updated.
For each 'generic' plot on the interface, the procedure *PlottingProc* is called. *PlottingProc* requires as input the plot addressed (e.g. "plot 1") and which output is to be shown on the plot. The output is specified by the user via a Netlogo "Chooser" on the interface associated to the plot (e.g. "N colonies").
Then the weather symbols, showing today's foraging period are updated: the *sign* with shape = "sun" is only shown, if foraging is possible (i.e. if *DailyForagingPeriod_s* > 0), the *sign* with shape = "cloud" is shown, if the daily foraging period is less than 4 hours.

```
to OutputDailyProc
 with-local-randomness    ; allows changing switching off plots without
                           ; changing the sequence of random numbers
 [
  random-seed ticks       ; local random seed, only valid within this procedure


  set TotalIBMColonies count colonies with [ cohortBased? = false ]
  set TotalQueens sum [ number ] of Bees with [ caste = "queen" ]
  set TotalMatedQueens sum [ number ] of Bees with [ caste = "queen" and mated? = true ]
  set TotalUnmatedQueens sum [ number ] of Bees with [ caste = "queen" and mated? = false ]
  set TotalHibernatingQueens sum [ number ] of Bees with [ activity = "hibernate"]
  set TotalColonies count colonies
  set TotalBeeAgents count bees
  set TotalMales sum [ number ] of Bees with [ caste = "male" ]
  set TotalActiveBees length ActiveBeesSortedList

  ifelse count bees with [brood? = false and caste = "worker"] > 0
    [ set meanWorkerWeight_mg mean [ weight_mg ] of bees
                                with [brood? = false and caste = "worker"] ]
    [ set meanWorkerWeight_mg   0 ]
  ifelse count bees with [brood? = false and caste = "queen"] > 0
    [ set meanQueenWeight_mg   mean [ weight_mg ] of bees
                                with [brood? = false and caste = "queen"] ]
    [ set meanQueenWeight_mg   0 ]
  ifelse count bees with [brood? = false] > 0
    [ set meanAdultWeight_mg   mean [ weight_mg ] of bees with [brood? = false] ]
    [ set meanAdultWeight_mg   0 ]

  set ColonyDensity_km2      TotalColonies /
                             (
                               (((max-pycor - EdgeTop) - (min-pycor + EdgeBottom))
                                 * ((max-pxcor - EdgeRight) - (min-pxcor + EdgeLeft)))
                                 / (Scaling_NLpatches/m * Scaling_NLpatches/m * 1000000)
                             )

  if ShowPlots?  = true
  [
    PlottingProc "plot 1" GenericPlot1   ; PlottingProc is called repeatedly..
    PlottingProc "plot 2" GenericPlot2
    PlottingProc "plot 3" GenericPlot3
    PlottingProc "plot 4" GenericPlot4
    PlottingProc "plot 5" GenericPlot5
  ]
  if ShowWeather? = true
  [
    ask Signs with [ shape = "sun"]
    [
```

```
      ifelse DailyForagingPeriod_s > 0
        [ show-turtle set label precision (DailyForagingPeriod_s / 3600) 1 ]
        [ hide-turtle set label " " ]
    ]   ; "sun" sign is shown, whenever there is an opportunity to forage

    ask Signs with [ shape = "cloud"]
    [
      ifelse DailyForagingPeriod_s < (4 * 3600)
        [ show-turtle ]
        [ hide-turtle ]
    ]   ; "cloud" sign is shown, whenever there is less than 4 hrs of foraging possible
  ]
 ]
end
```

PlottingProc

Purpose: Plotting output as specified by the user

Called by: *OutputDailyProc*

Input: *plotname, plotChoice*

Asking agents: none

Calling: none

Description

The specified (*plotname*) plot is addressed and the output chosen is shown. The procedure lists all output options and then calculates the graphs.

```
to PlottingProc [ plotname plotChoice ]

  set-current-plot plotname
  if plotChoice = "Foodsources sizes (histogram)"
    [
      set-plot-x-range 0 10
      create-temporary-plot-pen "N  "
      set-plot-pen-mode 1 ; 1: bars
      set-plot-pen-color black
      set-plot-pen-interval 1
      histogram [ size ] of Foodsources
    ]

  if plotChoice = "Matrilines (histogram)" ; NOTE: this plot does NOT correct
      ; for "number" (cohort size), hence IBM colonies will be overrepresented!
    [
      set-plot-x-range 0 140
      create-temporary-plot-pen "mtGene"
      set-plot-pen-mode 1 ; 1: bars
      set-plot-pen-color black
      set-plot-pen-interval 0.1
      histogram [ mtDNA ] of bees with [ caste = "queen" ]
    ]

  if plotChoice = "Genepool (histogram)" ; NOTE: this plot does NOT correct
      ; for "number" (cohort size), hence IBM colonies will be overrepresented!
    [
      let genepool []
      ask bees with [ caste = "queen" ]
       [
         foreach allelesList
           [ set genepool fput ? genepool ]
         foreach spermathecaList
           [ set genepool fput ? genepool ]
       ]
      set-plot-x-range 0 140
      create-temporary-plot-pen "alleles"
```

```
    set-plot-pen-mode 1 ; 1: bars
    set-plot-pen-color black
    set-plot-pen-interval 0.1
    histogram genepool ;
  ]

if plotChoice = "Colony sizes (histogram)" and count Colonies > 0 ; NOTE: this plot does
    ; NOT correct for "number" (cohort size), hence IBM colonies will be overrepresented!
  [
    if (max [colonysize] of Colonies > 0)
    [
      set-plot-x-range 0 10
      set-plot-x-range  0 max [colonysize] of Colonies
      create-temporary-plot-pen "N  "
      set-plot-pen-mode 1 ; 1: bars
      set-plot-pen-color black
      set-plot-pen-interval 20
      histogram [ colonysize ] of Colonies
    ]
  ]

 if plotChoice = "Bee weights [mg] (histogram)"  ; NOTE: this plot does NOT correct
    ; for "number" (cohort size), hence IBM colonies will be overrepresented!
  [
    create-temporary-plot-pen "queens"
    set-plot-pen-color red
    set-plot-x-range  0 1500
    set-plot-y-range  0 40
    set-plot-pen-mode 1 ; 1: bars
    set-plot-pen-interval 50
    histogram [ weight_mg ] of bees with [brood? = false and caste = "queen"]

    create-temporary-plot-pen "workers"
    set-plot-pen-color black
    set-plot-pen-mode 1 ; 1: bars
    set-plot-pen-interval 50
    histogram [ weight_mg ] of bees with [brood? = false and caste = "worker"]

    create-temporary-plot-pen "males"
    set-plot-pen-color green
    set-plot-pen-mode 1 ; 1: bars
    set-plot-pen-interval 50
    histogram [ weight_mg ] of bees with [brood? = false and caste = "male"]
  ]

if plotChoice = "N colonies"
 [
   set-plot-x-range 0 10
   create-temporary-plot-pen "Cols"
   plotxy ticks count Colonies
 ]

if plotChoice = "Species N colonies"
  [
    set-plot-x-range 0 10

    create-temporary-plot-pen "B_terrestris"
    set-plot-pen-color yellow
    plotxy ticks count colonies with [shape = "b_terrestris"]

    create-temporary-plot-pen "B_lapidarius"
    set-plot-pen-color black
    plotxy ticks count colonies with [shape = "b_lapidarius"]

    create-temporary-plot-pen "B_pascuorum"
    set-plot-pen-color brown
    plotxy ticks count colonies with [shape = "b_pascuorum"]

    create-temporary-plot-pen "B_hortorum"
    set-plot-pen-color green
    plotxy ticks count colonies with [shape = "b_hortorum"]

    create-temporary-plot-pen "B_pratorum"
    set-plot-pen-color orange
    plotxy ticks count colonies with [shape = "b_pratorum"]

    create-temporary-plot-pen "B_hypnorum"
    set-plot-pen-color blue
    plotxy ticks count colonies with [shape = "b_hypnorum"]

  ]


if plotChoice = "Foraging period max. [hrs]"
  [
   set-plot-x-range 0 10
   create-temporary-plot-pen "max. foraging"
   plotxy ticks DailyForagingPeriod_s / 3600
```

```
  ]

if plotChoice = "Foraging trips daily"
[
 ; set-plot-x-range 0 10
  create-temporary-plot-pen "N trips total"
  plotxy ticks TotalForagingTripsToday
]

if plotChoice = "Food available"
  [
  ; set-plot-x-range 0 10
   create-temporary-plot-pen "Nectar_l"
     set-plot-pen-color yellow
     plotxy ticks NectarAvailableTotal_l
   create-temporary-plot-pen "Pollen_kg"
     set-plot-pen-color red
     plotxy ticks PollenAvailableTotal_kg
  ]

if plotChoice = "Total adults"
[
  set-plot-x-range 0 10
  create-temporary-plot-pen "Adults"
  plotxy ticks TotalAdults
]

if plotChoice = "Species total adults"
  [
   set-plot-x-range 0 10
   create-temporary-plot-pen "B_terrestris"
     set-plot-pen-color yellow
     plotxy ticks sum [ number ] of bees
            with [ speciesName = "B_terrestris" and brood? = false and colonyID > 0 ]

   create-temporary-plot-pen "B_lapidarius"
     set-plot-pen-color black
     plotxy ticks sum [ number ] of bees
            with [ speciesName = "B_lapidarius" and brood? = false and colonyID > 0 ]

   create-temporary-plot-pen "B_pascuorum"
     set-plot-pen-color brown
     plotxy ticks sum [ number ] of  bees
           with [ speciesName = "B_pascuorum" and brood? = false and colonyID > 0 ]

   create-temporary-plot-pen "B_hortorum"
     set-plot-pen-color green
     plotxy ticks sum [ number ] of  bees
           with [ speciesName = "B_hortorum" and brood? = false and colonyID > 0 ]

   create-temporary-plot-pen "B_pratorum"
     set-plot-pen-color orange
     plotxy ticks sum [ number ] of  bees
           with [ speciesName = "B_pratorum" and brood? = false and colonyID > 0 ]

   create-temporary-plot-pen "B_hypnorum"
     set-plot-pen-color blue
     plotxy ticks count bees
           with [ speciesName = "B_hypnorum" and brood? = false and colonyID > 0 ]

   create-temporary-plot-pen "Psithyrus"
     set-plot-pen-color red
     plotxy ticks sum [ number ] of bees
           with [ speciesName = "Psithyrus" and brood? = false and colonyID > 0 ]
  ]

if plotChoice = "Species total adult queens"
  [
   set-plot-x-range 0 10
   create-temporary-plot-pen "B_terrestris"
     set-plot-pen-color yellow
     plotxy ticks sum [ number ] of bees
           with [ speciesName = "B_terrestris" and brood? = false and caste = "queen" ]

   create-temporary-plot-pen "B_lapidarius"
     set-plot-pen-color black
     plotxy ticks sum [ number ] of bees
           with [ speciesName = "B_lapidarius" and brood? = false and caste = "queen" ]

   create-temporary-plot-pen "B_pascuorum"
     set-plot-pen-color brown
     plotxy ticks sum [ number ] of bees
           with [ speciesName = "B_pascuorum" and brood? = false and caste = "queen" ]

   create-temporary-plot-pen "B_hortorum"
     set-plot-pen-color green
     plotxy ticks sum [ number ] of bees
           with [ speciesName = "B_hortorum" and brood? = false and caste = "queen" ]
```

```
   create-temporary-plot-pen "B_pratorum"
     set-plot-pen-color orange
     plotxy ticks sum [ number ] of bees
           with [ speciesName = "B_pratorum" and brood? = false and caste = "queen" ]

   create-temporary-plot-pen "B_hypnorum"
     set-plot-pen-color blue
     plotxy ticks sum [ number ] of bees
           with [ speciesName = "B_hypnorum" and brood? = false and caste = "queen" ]

   create-temporary-plot-pen "Psithyrus"
     set-plot-pen-color red
     plotxy ticks sum [ number ] of  bees
           with [ speciesName = "Psithyrus" and brood? = false and caste = "queen" ]
  ]


if plotChoice = "Species hibernating queens"
[
  set-plot-x-range 0 10

  create-temporary-plot-pen "B_terrestris"
  set-plot-pen-color yellow
  plotxy ticks sum [ number ] of  bees
          with [ speciesName = "B_terrestris" and brood? = false
          and caste = "queen" and activity = "hibernate" ]

  create-temporary-plot-pen "B_lapidarius"
  set-plot-pen-color black
  plotxy ticks sum [ number ] of  bees
          with [ speciesName = "B_lapidarius" and brood? = false
          and caste = "queen"  and activity = "hibernate"  ]

  create-temporary-plot-pen "B_pascuorum"
  set-plot-pen-color brown
  plotxy ticks sum [ number ] of  bees
          with [ speciesName = "B_pascuorum" and brood? = false
          and caste = "queen"  and activity = "hibernate"  ]

  create-temporary-plot-pen "B_hortorum"
  set-plot-pen-color green
  plotxy ticks sum [ number ] of  bees
          with [ speciesName = "B_hortorum" and brood? = false
          and caste = "queen"  and activity = "hibernate"  ]

  create-temporary-plot-pen "B_pratorum"
  set-plot-pen-color orange
  plotxy ticks sum [ number ] of  bees
          with [ speciesName = "B_pratorum" and brood? = false
          and caste = "queen"  and activity = "hibernate"  ]

  create-temporary-plot-pen "B_hypnorum"
  set-plot-pen-color blue
  plotxy ticks sum [ number ] of  bees
          with [ speciesName = "B_hypnorum" and brood? = false
          and caste = "queen"  and activity = "hibernate"  ]

  create-temporary-plot-pen "Psithyrus"
  set-plot-pen-color red
  plotxy ticks sum [ number ] of  bees
          with [ speciesName = "Psithyrus" and brood? = false
          and caste = "queen"  and activity = "hibernate"  ]
]

if plotChoice = "Hibernating queens"
[
  set-plot-x-range 0 10
  create-temporary-plot-pen  "N  "
  plotxy ticks sum [ number ] of bees with [ activity = "hibernate"]
]

if plotChoice = "Egg laying"
  [
   set-plot-x-range 0 10
   create-temporary-plot-pen "period"
   ifelse ShowInspectedColony? = true
        [

          ifelse count colonies with [ who = InspectTurtle ] = 1
            [ plotxy ticks [allEggs] of Colony InspectTurtle]
            [ clear-plot ]
         ]
        [ plotxy ticks (TotalEggs) ]
  ]

if plotChoice = "Stores: honey [ml] & pollen [g]"
  [
```

```
   set-plot-x-range 0 10
   create-temporary-plot-pen "honey"
   set-plot-pen-color yellow

   ifelse count Colonies = 0
     [ plotxy ticks 0 ]
     [
       ifelse ShowInspectedColony? = true
       [
         ifelse count colonies with [ who = InspectTurtle ] = 1
           [ plotxy ticks [energyStore_kJ] of Colony InspectTurtle / EnergyHoney_kJ/ml ]
           [ clear-plot ]
       ]
       [ plotxy ticks (mean [ energyStore_kJ ] of Colonies) / EnergyHoney_kJ/ml ]

     ]
   create-temporary-plot-pen "pollen"
   set-plot-pen-color orange
   ifelse count Colonies = 0
     [ plotxy ticks 0 ]
     [
       ifelse ShowInspectedColony? = true
       [
         ifelse count colonies with [ who = InspectTurtle ] = 1
          [ plotxy ticks [pollenStore_g] of Colony InspectTurtle ]
          [ clear-plot ]
       ]
       [ plotxy ticks (mean [ pollenStore_g ] of Colonies) ]
     ]
  ]

if plotChoice = "Colony structures"
[
 ifelse ShowInspectedColony? = true
 [
   ifelse count colonies with [ who = InspectTurtle ] = 1
   [
      set-plot-x-range 0 10
      create-temporary-plot-pen "Eggs"
        set-plot-pen-color blue
        plotxy ticks [allEggs] of Colony InspectTurtle
      create-temporary-plot-pen "Larvae"
        set-plot-pen-color yellow
        plotxy ticks [allLarvae] of Colony InspectTurtle
      create-temporary-plot-pen "Pupae"
        set-plot-pen-color brown
        plotxy ticks [allPupae] of Colony InspectTurtle
      create-temporary-plot-pen "Workers"
        set-plot-pen-color black
        plotxy ticks [allAdultWorkers] of Colony InspectTurtle
      create-temporary-plot-pen "Males"
        set-plot-pen-color green
        plotxy ticks [allAdultMales] of Colony InspectTurtle
      create-temporary-plot-pen "Queens"
        set-plot-pen-color red
        plotxy ticks [allAdultQueens] of Colony InspectTurtle
   ]
   [ clear-plot ]  ; plot is cleared after the previous 'inspected colony' has died
]

 [  ; if  ShowInspectedColony? = FALSE:
   set-plot-x-range 0 10
      create-temporary-plot-pen "Eggs"
        set-plot-pen-color blue
        plotxy ticks TotalEggs
      create-temporary-plot-pen "Larvae"
        set-plot-pen-color yellow
        plotxy ticks TotalLarvae
      create-temporary-plot-pen "Pupae"
        set-plot-pen-color brown
        plotxy ticks TotalPupae
      create-temporary-plot-pen "Workers"
        set-plot-pen-color orange
        plotxy ticks TotalAdultWorkers
      create-temporary-plot-pen "Males"
        set-plot-pen-color green
        plotxy ticks TotalAdultMales
      create-temporary-plot-pen "Queens"
        set-plot-pen-color black
        plotxy ticks TotalAdultQueens
   ]
  ]

if plotChoice = "Switchpoints"
[
  set-plot-x-range 0 50
  create-temporary-plot-pen "SP"
  set-plot-pen-color black
```

```
    set-plot-pen-mode  1
    histogram [ switchPointDate - eusocialPhaseDate ] of Colonies
              with [ eusocialPhaseDate + switchPointDate < NotSetHigh ]
  ]

  if plotChoice = "Sex ratio"
  [
    set-plot-y-range 0 1
    create-temporary-plot-pen "M:F"
    set-plot-pen-color black
    set-plot-pen-mode  0
    if TotalAdultQueens > 0
    [
      plot TotalAdultMales / TotalAdultQueens
    ]
  ]

end
```

DrawCohortsProc

Purpose: Shows the cohorts of each *colony* on the interface

Called by: *Go*

Asking agents: none

Calling: none

Description

For each *colony*, the number of *bees* of the same age (local variable *cohortSize*) is determined (with exception of the mother queen). These *bees* are presented as lines (*shape* = "halfline") on the interface and the length of the line (*size*) reflects the number of *bees* in this age group:

```
to DrawCohortsProc
 ask colonies
 [
   let whoCol who  ; saves colony ID
   if count Bees with [ colonyID = whoCol and shape = "halfline" ] > 0
    ; make sure there are some bees that can be addressed
    [
     let currentAge 0 ; defines which age cohort is now addressed
     let maxAge 1 + [ broodAge  + adultage ] of
                max-one-of Bees with [ colonyID = whoCol and shape = "halfline" ]
                                      [ broodAge  + adultage ]
     repeat maxAge ; to address all cohorts in this colony
     [
       let cohortSize sum [number] of Bees
                      with [ broodAge  + adultage = currentAge
                      and  colonyID = whoCol
                      and shape = "halfline" ] ; i.e. not the mother queen
       ask Bees with [ broodAge  + adultage = currentAge
                and colonyID = whoCol
                and shape = "halfline"
                ]
          [ set size cohortSize * CohortSymbolSize ] ; length of line reflects cohort size
       set currentAge currentAge + 1 ; the next cohort to be addressed is 1 day older
     ]
   ]
 ]
end
```

AssertionProc

Purpose: causes a simulation run to stop once an assertion in the code has been violated

Called by: only called, if an assertion is violated. Assertions are placed in a large number of procedures and reported-procedures.

Asking agents: none

Calling: none

Input: *message*

Description

The procedure is called whenever an assertion is violated. In this case, a message is shown in the NetLogo "Command center" on the interface, specifying what caused the error. This message is then also saved in the global variable *AssertionMessage*, to be accessible when running Bumble-BEEHAVE in combination with the R software. *AssertionViolated* is then set true which will cause a user message to pop up and stop the simulation run at the beginning of the next time step.

```
to AssertionProc [ message ]
  show message
  set AssertionMessage  message
  set AssertionViolated true
end
```

ActivityListButton

Purpose: Shows the logged activities of all adult *bees* as output in the "Command Center" (Interface)

Called by: Button ("Activity List")

Asking agents: none

Calling: none

Description

All adult *bees* are addressed, sorted by their *species* (*speciesID*), ID (*who*) of their *colony* and their own ID (*who*). For each *bee*, a line is printed on the "Command Centre" (Netlogo Interface), showing the *species*, the *caste*, the ID's of the *colony* and the bee, the age of the *bee*, and then the list of the logged activities.

```
to ActivityListButton
type "day: " type day print " (species caste colony bee age activities) "
; adult bees within a colony are sorted by "speciesID " then "colonyID" and then by "who":
foreach sort-on [ speciesID * 1000000000000000 + colonyID * 10000000 + who ]
                bees with [ stage = "adult" and colonyID >= 0 ]
  [ ask ?
     [
       type speciesName type " "
```

```
        if caste = "worker"  [ type "W" type " " ]
        if caste = "queen" and mated? = true [ type "Q" type " " ]
        if caste = "queen" and mated? = false [ type "q" type " " ]
        if caste = "male" and mated? = false [ type "M" type " " ]
        type colonyID type " "
        type who type " "
        type adultAge type " "
        print activityList ] ]
 end
```

DefaultProc

Purpose: Sets the input options on the interface to the default parameter values

Called by: Button ("Default")

Asking agents: none

Calling: none

Description

Sets all input options on the interface to the default parameter values, except for *RAND_SEED*, which remains unchanged.

```
to DefaultProc
  ;;; set RAND_SEED 1
  set AbundanceBoost 1
  set B_hortorum 0
  set B_hypnorum 0
  set B_lapidarius 0
  set B_pascuorum 0
  set B_pratorum 0
  set B_terrestris 500
  set Backgroundcolour 5
  set ChooseInputFile "BBH-T_Suss1.txt"
  set ChooseInputMap "BBH-I_Suss1.png"
  set COLONIES_IBM 0
  set FlowerspeciesFile "BBH-Flowerspecies_Suss.csv"
  set FoodSourceLimit 25
  set ForagingMortalityFactor 1
  set ForagingMortalityModel "high"
  set GenericPlot1 "Species total adult queens"
  set GenericPlot2 "Species N colonies"
  set GenericPlot3 "Food available"
  set GenericPlot4 "Colony structures"
  set GenericPlot5 "Species total adults"
  set Gridsize 500
  set INPUT_FILE "BBH-T_Suss1.txt"
  set InputMap "BBH-I_Suss1.png"
  set InspectTurtle 1
  set KeepDeadColonies? true
  set Lambda_detectProb -0.005
  set MapAreaIncluded "complete"
  set MasterSizeFactor 1
  set MaxHibernatingQueens 10000
  set MinSizeFoodSources? TRUE
  set N_Badgers 0
  set N_Psithyrus 0
  set RemoveEmptyFoodSources? TRUE
  set SexLocus? false ; true
  set ShowCohorts? true
  set ShowDeadCols? false
  set ShowFoodsources? true
  set ShowGrid? false
  set ShowInspectedColony? false
  set ShowMasterpatchesOnly? false
  set ShowNests? true
```

```
  set ShowPlots? true
  set ShowQueens? true
  set ShowSearchingQueens? true
  set ShowWeather? true
  set SpeciesFilename "BBH-BumbleSpecies_UK_01.csv"
  set StopExtinct? true
  set UnlimitedMales? true
  set Weather "Constant 8 hrs"
  set WinterMortality? true
  set X_Days 90
end
```

VersionTestProc

Purpose: runs the model under defined conditions to check for changes made to the code

Called by: Button ("Version Test")

Asking agents: none

Calling:

DefaultProc
Setup

Description

After calling *DefaultProc*, the initial number of badgers and initial queens for all bumblebee *species* (including Psithyrus) is set to a value larger 0. The *Setup* is called and the model runs for 2 years. The local variable *testValue* is calculated from a number of *bees* and *foodsources*, and compared to the value of the local variable *expectedValue*, which is set to the result expected. A user message then pops up to inform the user whether the code (or one of the input files) has or probably has not changed. Note that only changes resulting in different numbers of *bees* (or *foodsources*) after two years can be detected.

```
to VersionTestProc ; to test whether the model was changed
 ; value expected, if the model hasn't changed:
 let expectedValue 8560

 DefaultProc

 set RAND_SEED 1
 set B_hortorum 20
 set B_hypnorum 20
 set B_lapidarius 20
 set B_pascuorum 20
 set B_pratorum 20
 set B_terrestris 100
 set N_Psithyrus 20
 set N_Badgers 5

 Setup
 repeat 2 * 365
  [
    Go
    if AssertionViolated = true
     [
      ask patches [ set pcolor red ]
      stop
     ]
  ]
  let testValue TotalBeesEverProduced + TotalHibernatingQueens
                 + TotalMales + TotalAdultWorkers + TotalFoodSources
  type testValue type "    Difference: " print testValue - expectedValue
  ifelse testValue = expectedValue
```

```
    [ user-message
          "No deviation detected from the pulished version of Bumble-BEEHAVE (2017)" ]
    [ user-message "CHANGES MADE TO THE MODEL OR INPUT FILES!"]
end
```

REFERENCES

Alford, D. V. "A study of the hibernation of bumblebees (Hymenoptera: Bombidae) in southern England." *The Journal of Animal Ecology* (1969a): 149-170.

Alford, D. V. "Studies on the fat-body of adult bumble bees." *Journal of Apicultural Research* 8.1 (1969b): 37-48.

Alford, D. V. (1975). Bumblebees, Davis-Poynter, London.

Becher, M. A., Grimm, V., Thorbek, P., Horn, J., Kennedy, P. J., & Osborne, J. L. (2014). BEEHAVE: a systems model of honeybee colony dynamics and foraging to explore multifactorial causes of colony failure. Journal of Applied Ecology, 51(2), 470-482.

Becher, M. A., Grimm, V., Knapp, J., Horn, J., Twiston-Davies, G., & Osborne, J. L. (2016). BEESCOUT: A model of bee scouting behaviour and a software tool for characterizing nectar/pollen landscapes for BEEHAVE. Ecological Modelling, 340, 126-133.

Beekman, M., Stratum, P., & Lingeman, R. (1998). Diapause survival and post-diapause performance in bumblebee queens (Bombus terrestris). *Entomologia experimentalis et applicata*, *89*(3), 207-214.

Beye, M., Hasselmann, M., Fondrk, M. K., Page, R. E., & Omholt, S. W. (2003). The gene csd is the primary signal for sexual development in the honeybee and encodes an SR-type protein. *Cell*, *114*(4), 419-429.

Bloch, G., & Hefetz, A. (1999). Regulation of reproduction by dominant workers in bumblebee (Bombus terrestris) queenright colonies. *Behavioral Ecology and Sociobiology*, *45*(2), 125-135.

Brian, A. D. (1957). Differences in the flowers visited by four species of bumble-bees and their causes. *The Journal of Animal Ecology*, 71-98.

Cnaani, J., Borst, D. W., Huang, Z. Y., Robinson, G. E., & Hefetz, A. (1997). Caste determination in Bombus terrestris: differences in development and rates of JH biosynthesis between queen and worker larvae. *Journal of Insect Physiology*, *43*(4), 373-381.

Cnaani, J., Robinson, G. E., Bloch, G., Borst, D., & Hefetz, A. (2000a). The effect of queen-worker conflict on caste determination in the bumblebee Bombus terrestris. *Behavioral Ecology and Sociobiology*, *47*(5), 346-352.

Cnaani, J., Robinson, G. E., & Hefetz, A. (2000b). The critical period for caste determination in Bombus terrestris and its juvenile hormone correlates. *Journal of Comparative Physiology A*, *186*(11), 1089-1094.

Cnaani, J., Schmid-Hempel, R., & Schmidt, J. O. (2002). Colony development, larval development and worker reproduction in Bombus impatiens Cresson. *Insectes Sociaux, 49*(2), 164-170.

Dornhaus, A., Chittka, L. (2001). Food alert in bumblebees (Bombus terrestris): possible mechanisms and evolutionary implications. Behavioral Ecology and Sociobiology, 50(6), 570-576.

Dornhaus, A., Chittka, L. (2004). Information flow and regulation of foraging activity in bumble bees (Bombus spp.). Apidologie, 35(2), 183-192.

Duchateau, M. J., & Marien, J. (1995). Sexual biology of haploid and diploid males in the bumble bee Bombus terrestris. *Insectes Sociaux*, *42*(3), 255-266.

Duchateau, M. J., & Velthuis, H. H. W. (1988). Development and reproductive strategies in Bombus terrestris colonies. *Behaviour*, *107*(3), 186-207.

Duchateau, M. J., Velthuis, H. H., & Boomsma, J. J. (2004). Sex ratio variation in the bumblebee Bombus terrestris. *Behavioral Ecology*, *15*(1), 71-82.

Duchateau, M. J., Hoshiba, H., & Velthuis, H. H. W. (1994). Diploid males in the bumble bee Bombus terrestris. *Entomologia experimentalis et applicata*, *71*(3), 263-269.

Dramstad, W. E. (1996). Do bumblebees (Hymenoptera: Apidae) really forage close to their nests?. *Journal of Insect Behavior*, *9*(2), 163-182.

Free, J. B. (1955a). The collection of food by bumblebees. *Insectes Sociaux*, *2*(4), 303-311.

Free, J. B. (1955b). The division of labour within bumblebee colonies. *Insectes sociaux, 2*(3), 195-212

Goulson, D. (2010). *Bumblebees: behaviour, ecology, and conservation*. Oxford University, Oxford.

Grimm, V., Berger, U., Bastiansen, F., Eliassen, S., Ginot, V., Giske, J. et al. (2006) A standard protocol for describing individual-based and agent-based models. Ecological Modelling, 198, 115–126.

Grimm, V., Berger, U., DeAngelis, D.L., Polhill, G., Giske, J. & Railsback, S.F. (2010) The ODD protocol: a review and first update. Ecological Modelling, 221, 2760–2768.

Harder, L. D. (1983). Flower handling efficiency of bumble bees: morphological aspects of probing time. Oecologia, 57(1-2), 274-280)

Harder, L. D. (1985). Morphology as a predictor of flower choice by bumble bees. *Ecology, 66*(1), 198-210.

Heinrich, Bernd. Bumblebee economics. Harvard University Press, 1979.

Holm, S. N. (1966). The utilization and management of bumble bees for red clover and alfalfa seed production. *Annual review of entomology*, *11*(1), 155-182.

Holm, S. N. (1972). Weight and life length of hibernating bumble bee queens (Hymenoptera: Bombidae) under controlled conditions. *Insect Systematics & Evolution, 3*(4), 313-320.

Honk, C. V., Röseler, P. F., Velthuis, H. H. W., & Hoogeveen, J. C. (1981). Factors influencing the egg laying of workers in a captive Bombus terrestris colony. *Behavioral Ecology and Sociobiology, 9*(1), 9-14.

Ings, T. C., Ward, N. L., & Chittka, L. (2006). Can commercially imported bumble bees out-compete their native conspecifics?. Journal of Applied Ecology, 43(5), 940-948.

Inouye, D. W. (1980). The effect of proboscis and corolla tube lengths on patterns and rates of flower visitation by bumblebees. *Oecologia*, *45*(2), 197-201.

Irwin, R. E., Bronstein, J. L., Manson, J. S., & Richardson, L. (2010). Nectar robbing: ecological and evolutionary perspectives. *Annual Review of Ecology, Evolution, and Systematics*, *41*, 271-292.

Knight, M. E., Martin, A. P., Bishop, S., Osborne, J. L., Hale, R. J., Sanderson, R. A., & Goulson, D. (2005). An interspecific comparison of foraging range and nest density of four bumblebee (Bombus) species. Molecular Ecology, 14(6), 1811-1820.

Kowalczyk, R., Zalewski, A. & Bogumiła, J (2006). "Daily movement and territory use by badgers Meles meles in Białowieża Primeval Forest, Poland." Wildlife Biology 12(4): 385-391.

Kruuk, H, & Parish, T. (1982) Factors affecting population density, group size and territory size of the European badger, Meles meles." Journal of Zoology 196(1), 31-39.

Lihoreau, M., Raine, N. E., Reynolds, A. M., Stelzer, R. J., Lim, K. S., Smith, A. D., ... & Chittka, L. (2012). Radar tracking and motion-sensitive cameras on flowers reveal the development of pollinator multi-destination routes over large spatial scales. PLoS Biol, 10(9), e1001392.

Lopez-Vaamonde, C., Raine, N. E., Koning, J. W., Brown, R. M., Pereboom, J. J. M., Ings, T. C., ... & Bourke, A. F. G. (2009). Lifetime reproductive success and longevity of queens in an annual social insect. *Journal of Evolutionary Biology*, *22*(5), 983-996.

Moerman, R., Vanderplanck, M., Roger, N., Declèves, S., Wathelet, B., Rasmont, P., ... & Michez, D. (2015). Growth rate of bumblebee larvae is related to pollen amino acids. *Journal of Economic Entomology*, 109, 25–30.

Moerman, R., Vanderplanck, M., Fournier, D., Jacquemart, A. L., & Michez, D. (2017). Pollen nutrients better explain bumblebee colony development than pollen diversity. *Insect Conservation and Diversity*. doi: 10.1111/icad.12213

Morse, D. H. (1986). Predatory risk to insects foraging at flowers. *Oikos,* 46(2), 223-228.

Núñez, J. A. (1966). Quantitative Beziehungen zwischen den Eigenschaften von Futterquellen und dem Verhalten von Sammelbienen. Journal of Comparative Physiology A: 53(2), 142-164.

Núñez, J. (1970). The relationship between sugar flow and foraging and recruiting behaviour of honey bees (Apis mellifera L.). Animal Behaviour, 18, 527-538.

Osborne, J. L., Martin, A. P., Shortall, C. R., Todd, A. D., Goulson, D., Knight, M. E., ... & Sanderson, R. A. (2008a). Quantifying and comparing bumblebee nest densities in gardens and countryside habitats. *Journal of Applied Ecology, 45*(3), 784-792.

Osborne, J. L., Martin, A. P., Carreck, N. L., Swain, J. L., Knight, M. E., Goulson, D., ... & Sanderson, R. A. (2008b). Bumblebee flight distances in relation to the forage landscape. *Journal of Animal Ecology*, *77*(2), 406-415.

Peat, J., & Goulson, D. (2005). Effects of experience and weather on foraging rate and pollen versus nectar collection in the bumblebee, Bombus terrestris. *Behavioral Ecology and Sociobiology*, *58*(2), 152-156.

Pereboom, J. J. M., Velthuis, H. H. W., & Duchateau, M. J. (2003). The organisation of larval feeding in bumblebees (Hymenoptera, Apidae) and its significance to caste differentiation. *Insectes Sociaux*, *50*(2), 127-133.

Plowright, R. C., & Jay, S. C. (1968). Caste differentiation in bumblebees (Bombus Latr.: Hym.) I.—The determination of female size. Insectes Sociaux, 15(2), 171-192.

Raine, N. E., & Chittka, L. (2007). Pollen foraging: learning a complex motor skill by bumblebees (Bombus terrestris). Naturwissenschaften, 94(6), 459-464.

Reilly, L. A., & Courtenay, O. (2007). Husbandry practices, badger sett density and habitat composition as risk factors for transient and persistent bovine tuberculosis on UK cattle farms. Preventive Veterinary Medicine, 80(2), 129-142.

Ribeiro, M. D. F., Velthuis, H. H. W., Duchateau, M. J., & Van der Tweel, I. (1999). Feeding frequency and caste differentiation in Bombus terrestris larvae. *Insectes Sociaux*, *46*(4), 306-314.

Rodd, F. H., Plowright, R. C., & Owen, R. E. (1980). Mortality rates of adult bumble bee workers (Hymenoptera: Apidae). *Canadian Journal of Zoology*, *58*(9), 1718-1721.

Sanderson, R. A., Goffe, L. A., & Leifert, C. (2015). Time-series models to quantify short-term effects of meteorological conditions on bumblebee forager activity in agricultural landscapes. *Agricultural and Forest Entomology*, *17*(3), 270-276.

Schmickl, T., & Crailsheim, K. (2007). HoPoMo: A model of honeybee intracolonial population dynamics and resource management. Ecological Modelling, 204(1), 219-245.

Schmid-Hempel, P., & Heeb, D. (1991). Worker mortality and colony development in bumblebees, Bombus lucorum (L.)(Hymenoptera, Apidae). Mitt. Schweiz. Entomol. Ges, 64, 93-108.

Schmid-Hempel, P., Kacelnik, A., & Houston, A. I. (1985). Honeybees maximize efficiency by not filling their crop. Behavioral Ecology and Sociobiology, 17(1), 61-66.

Schmid-Hempel, R., & Schmid-Hempel, P. (2000). Female mating frequencies in Bombus spp. from Central Europe. *Insectes Sociaux*, *47*(1), 36-41.

Seeley, T.D. (1994) Honey bee foragers as sensory units of their colonies. Behavioral Ecology and Sociobiology, 34, 51-62.

Seeley, T. D. (1995). The wisdom of the hive. Cambridge.

Silvola, J. (1984). Respiration and energetics of the bumblebee Bombus terrestris queen. *Holarctic ecology*, 7, 177-181.

Simpson, S. J., & Raubenheimer, D. (2012). The nature of nutrition: a unifying framework. Australian Journal of Zoology, 59(6), 350-368.

Stelzer, R. J., Chittka, L., Carlton, M., & Ings, T. C. (2010). Winter active bumblebees (Bombus terrestris) achieve high foraging rates in urban Britain. PLoS One, 5(3), e9559.

Stelzer, R. J., Raine, N. E., Schmitt, K. D., & Chittka, L. (2010). Effects of aposematic coloration on predation risk in bumblebees? A comparison between differently coloured populations, with consideration of the ultraviolet. *Journal of Zoology*, *282*(2), 75-83.

Stout, J. C., Allen, J. A., & Goulson, D. (2000). Nectar robbing, forager efficiency and seed set: bumblebees foraging on the self incompatible plant Linaria vulgaris (Scrophulariaceae). *Acta Oecologica*, *21*(4), 277-283.

Tasei, J. N., Moinard, C., Moreau, L., Himpens, B., & Guyonnaud, S. (1998). Relationship between aging, mating and sperm production in captive Bombus terrestris. *Journal of Apicultural Research*, *37*(2), 107-113.

Pirk, C. W., Boodhoo, C., Human, H., & Nicolson, S. W. (2010). The importance of protein type and protein to carbohydrate ratio for survival and ovarian activation of caged honeybees (Apis mellifera scutellata). Apidologie, 41(1), 62-72.

Stabler, D., Paoli, P. P., Nicolson, S. W., & Wright, G. A. (2015). Nutrient balancing of the adult worker bumblebee (Bombus terrestris) depends on the dietary source of essential amino acids. Journal of Experimental Biology, 218(5), 793-802.

Winston, M. L. (1987). The biology of the honey bee. Harvard University Press.
