# Supplementary material for: Bumble‐BEEHAVE: A systems model for exploring multifactorial causes of bumblebee decline at individual, colony, population and community level
Source: J Appl Ecol. 2018 May 22;55(6):2790–801. doi: 10.1111/1365-2664.13165 (PMC6221040; doi:10.1111/1365-2664.13165)
Supplement: Supplementary file 8 [file JPE-55-2790-s008.pdf]

CHANGES to Bumble-BEESCOUT

Overview main changes:

- new option to create input files for Bumble-BEEHAVE
- 5 new colour options for the crop map are provided
- additional specification of flowers (corolla depth, nectar volume, protein content pollen) and inter flower flight time of foragers
- option to define the crop map via a text file instead of an image file
- option to have multiple flower species within the same patch/habitat, defined via a new input file (HabitatsInput). For details, see Supporting Information of the Bumble-BEEHAVE publication (Becher et al. 2017, Ecology Letters): "SI_07_Methodology for calculating BEESCOUT 2.0 Habitats input file and Bumble-BEEHAVE" and the Bumble-BEEHAVE manual (SI_02).

CHANGES IN DETAIL:

new on interface:

Setup map:

chooser:
"TextMap"
(new option on chooser "InputFile": "TextMap")
button:
"Bumble-BEEHAVE Example"

Resolution:

switch:
"HighResolution" (high resolution not available for TextMaps!)
buttons:
"translate to High Res"
"translate to Low Res"

Display

button:
"Habitats"

Create Bumble-BEEHAVE files:

button:
"create Bumble-BEEHAVE files"
input:
"BumbleworldImage_Filename"

"Bumbleworld_Inputfile"
<u>switch:</u>
"AutoName"

Definition food patches:

<u>New colours:</u>
Brown (BR), Turquoise (T), Violet (V), Magenta (M), Pink (P)

<u>new input:</u>
"Corolla_.." (for each colour) (corolla depth in mm)
"FlowerVol_.." (for each colour) (nectar volume in flower in µl)
"intFlowerT_.." (for each colour) (time to fly from one flower to the next in s)
"Protein_.." (for each colour) (proportion of protein in pollen)
"HabitatsInput" (input file defining flower species and abundancies in each habitat type)

<u>new buttons:</u>
"DefineHabitatProc"
"Apply!"

<u>new monitors:</u>
"FlowerSpeciesList_.." (for each colour)
"N ... gridcells" (for each colour)

<u>new chooser:</u>
"ChooseHabitatsInput"

New variables:

new extension used:
csv

new bees-own variable:
distanceFromColony_m

new patches-own variable:
habitatTypePatch

new patchStatistics-own variables:
corollaDepth_mm
nectarFlowerVolume_myl
interFlowerTime_s
flowerSpeciesList
patchInfo
proteinPollenProp

new global variables:

ColourCodeList
HabitatDataCSV
FlowerSpeciesList_R
FlowerSpeciesList_Y
FlowerSpeciesList_G
FlowerSpeciesList_B
FlowerSpeciesList_BR
FlowerSpeciesList_T
FlowerSpeciesList_V
FlowerSpeciesList_M
FlowerSpeciesList_P

Changes to Procedures:

New procedures:

ReadMapFromTextProc
DefineHabitatsProc
BumbleworldOutProc
BumbleworldSetFlowerSpeciesProc

Setup

If the high resolution option is chosen, the size of the Netlogo world is increased from 300 x 210 grid cells to 1500 x 1050. (Note that high resolution can considerably slow down the setup and only makes sense if the InputMap refers to am image file, not to a text file).

```
to Setup
  __clear-all-and-reset-ticks

   ; new for Bumble-BEESCOUT
  ifelse HighResolution = false ;
   [
     resize-world 0 300 0 210  ; min-pxcor max-pxcor min-pycor max-pycor
     set-patch-size 5
   ]
   [
     set-patch-size 1
     resize-world 0 1500 0 1050
   ]
  if AutoName = true
  [
    ifelse InputFile = "TextMap"
    [
      let name1 substring TextMap 0 (length TextMap - 4)
      set BumbleworldImage_Filename (word name1 "_View.png")
      set Bumbleworld_Inputfile (word name1 "_Patches.txt")
    ]
    [
      let name1 substring InputFile 0 (length InputFile - 4)
      set BumbleworldImage_Filename (word name1 "_View.png")
      set Bumbleworld_Inputfile (word name1 "_Patches.txt")
    ]
  ]
...
```

FlowerSpeciesLists are defined as empty lists:

```
  set FlowerSpeciesList_R []
  set FlowerSpeciesList_Y []
  set FlowerSpeciesList_G []
```

```
  set FlowerSpeciesList_B []
  set FlowerSpeciesList_BR []
  set FlowerSpeciesList_T []
  set FlowerSpeciesList_V []
  set FlowerSpeciesList_M []
  set FlowerSpeciesList_P []
```

the new colours can be added to PatchColoursList:

```
  if TurquoisePatches = true [ set PatchColoursList fput Turquoise PatchColoursList ]
  if VioletPatches = true [ set PatchColoursList fput Violet PatchColoursList ]
  if BrownPatches = true [ set PatchColoursList fput Brown PatchColoursList ]
  if MagentaPatches = true [ set PatchColoursList fput Magenta PatchColoursList ]
  if PinkPatches = true [ set PatchColoursList fput Pink PatchColoursList ]
```

The crop map can either be created from a text file or from an image file:

```
  ifelse InputFile != "No input file"
    [
      ifelse InputFile = "TextMap"
      [
        ReadMapFromTextProc
        if count patches with [ originalColor = BorderColor ] > 0 [ DetermineBordersProc ]
      ]
      [
        ImportMapProc                    ;;loads the crop map
        if count patches with [ originalColor = BorderColor ] > 0 [ DetermineBordersProc ]
      ]
     ]
```

DetermineBordersProc

minor change in the way, borders are calculated:

```
set RightBorder max-pxcor + 1
  if count patches with [ (pxcor > (max-pxcor / 2)) and (originalColor = BorderColor)] > 0
   [ set RightBorder min [ pxcor ] of patches with [(pxcor > (max-pxcor / 2))
     and (originalColor = BorderColor)] ]

set LeftBorder min-pxcor - 1
if count patches with [(pxcor < (max-pxcor / 2)) and (originalColor = BorderColor)] > 0
    [ set LeftBorder  max [ pxcor ] of patches with [(pxcor < (max-pxcor / 2))
      and (originalColor = BorderColor)]]
if RightBorder - LeftBorder <= 2
     [
       set RightBorder max-pxcor + 1   ; i.e. outside the "world"
       set LeftBorder min-pxcor - 1
     ]
```

ReadMapFromTextProc

This is a completely new procedure to import crop maps from text files:

```
to ReadMapFromTextProc
let nextColourType -1
file-open TextMap
let ncols read-from-string file-read-line   ; reads in number of columns
let nrows read-from-string file-read-line   ; reads in number of rows
set Scaling read-from-string file-read-line ; reads in the scaling of the map (size [m]
                                            ; of each grid cell)
set Scale_X1 1
set Scale_X2 ncols
set ScaleDistance_m Scaling * (Scale_X2 - Scale_X1)
set ColourCodeList read-from-string file-read-line
if ncols > max-pxcor or nrows > max-pycor
```

```
   [ user-message "TextMap does not match size of BEESCOUT world" ]

; place map in the centre:
let leftMargin (max-pxcor - ncols) / 2
let bottomMargin (max-pycor - nrows) / 2

foreach sort patches [
  ask ?
  [
    if (pxcor >= leftMargin and pxcor < ncols + leftMargin) and (pycor >= bottomMargin
        and pycor < nrows + bottomMargin) and file-at-end? = false
    [
      set nextColourType file-read
      ifelse nextColourType > 0
       [

         set habitatTypePatch nextColourType
         set pcolor item (nextColourType - 1) colourCodeList
       ]

       [ set pcolor grey ]
    ]
  ]
]
file-close

ask patches
  [
    set originalColor pcolor  ;; the color of the (grid) patch in the original file
    set plabel-color black
    set flowerPatchID -1
    set patchColor pcolor  ;; saves the color after rounding
   ]

end
```

ImportMapProc

New colour options and "if..(pcolor != BorderColor)" added:

```
    if remainder pcolor 10 > White_th [ set pcolor white ]
    if (pcolor > Red_min) and (pcolor <= Red_max) and (pcolor != BorderColor)
        [ set pcolor red ]
    if (pcolor > Yellow_min) and (pcolor <= Yellow_max) and (pcolor != BorderColor)
        [ set pcolor yellow ]
    if (pcolor > Green_min) and (pcolor <= Green_max) and (pcolor != BorderColor)
        [ set pcolor green ]
    if (pcolor > Blue_min) and (pcolor <= Blue_max) and (pcolor != BorderColor)
        [ set pcolor Blue ]
    if (pcolor > Turquoise_min) and (pcolor <= Turquoise_max) and (pcolor != BorderColor)
        [ set pcolor Turquoise ]
    if (pcolor > Violet_min) and (pcolor <= Violet_max) and (pcolor != BorderColor)
        [ set pcolor Violet ]
    if (pcolor > Brown_min) and (pcolor <= Brown_max) and (pcolor != BorderColor)
        [ set pcolor Brown ] ;Woodland
    if (pcolor > Magenta_min) and (pcolor <= Magenta_max) and (pcolor != BorderColor)
        [ set pcolor Magenta ]
    if (pcolor > Pink_min) and (pcolor <= Pink_max) and (pcolor != BorderColor)
        [ set pcolor Pink ]
    set patchColor pcolor
```

AnalyseProc

To speed up analysis of the map, only patches in the vicinity of the current patch are addressed:

```
  foreach sort patches
  [
   ...
   if flowerPatchID = -1 ;; if patch is not identified yet
   [
    let search-patches patches in-radius Repetitions
    repeat Repetitions
```

```
    [
    ...
    ask search-patches with [(pcolor = currentColor) and (flowerPatchID = currentPatchID)]
       ;; connected (nlogo-)patches around the firstPatchOfFlowerpatch are searched defined
       ;; as part of this flower patch

            [
               ...
```

CreatePatchStatisticsProc

When the patchstatistics are created, the new colours and the new patchstatistic-own variables are taken into account:

```
create-PatchStatistics Npatches
     [
       set size 2  ; changed: previous version: size = 1
       set shape "circle"
       set currentWho Who
       set patchInfo "no info"
       set xcor mean [ pxcor ] of patches with [ flowerPatchID = currentWho ]
       set ycor mean [ pycor ] of patches with [ flowerPatchID = currentWho ]
       set currentColor patchcolor

       if currentWho != flowerPatchID
         [
           ask min-one-of patches with [ flowerPatchID = currentWho ] [distance myself]
           [

...

set flowerSpeciesList []
if patchColor = red
[
  set patchType PATCHTYPE_R
  set concentration Conc_R
  set nectarGathering_s t_Nectar_R
  set pollenGathering_s t_Pollen_R
  set startDay Start_R
  set stopDay Stop_R
  set corollaDepth_mm Corolla_R
  set nectarFlowerVolume_myl FlowerVol_R
  set interFlowerTime_s intFlowerT_R
  set proteinPollenProp Protein_R
]
```

(and similar for the other colours)

DetermineSizeProc

During the process of determining sizes of flower patches and their distances to the colony, the user is updated on the progress under the high resolution setting:

```
 foreach sort patchStatistics
 [ ask ?
   [
   if HighResolution = true
    [ type "Progress in DetermineSizeProc [%]: "
      print precision (100 * who / count patchStatistics) 1 ]

...
```

Again, code is modified to take the new colour options into account:

```
if color = Turquoise - 1 ; Grassland Pasture
     [
       set quantityNectar_l (Nectar_T / 1000) * areaSqm  ; (Nectar_T: ml)
       set quantityPollen_g Pollen_T * areaSqm
     ]
```

(and similar for the other new colours)

DefineHabitatsProc

This is a completely new procedure that loads a csv file to define flower species and abundance in the various crop/habitat types:

```
to DefineHabitatsProc
 set HabitatDataCSV csv:from-file HabitatsInput  ; the csv file contains information about
which habitat types are represented by which colour and the relative abundance of flower
species
 let headerList item 0 HabitatDataCSV   ; the header of the csv table
 let line 0

 foreach but-first HabitatDataCSV ; goes through all 'lines' (except of header) in ordered way
  [
    set line line + 1
    let currentFlowerList []
    let habitatType "none"
    let column 0
    let nameOfCurrentList first ?
    foreach but-first ? ; goes through all 'columns' of this 'line'
    [
      set column column + 1
      let content ? ; the content of a single 'cell'
      ifelse is-string? content = true ;
       [ set habitatType ? ] ; i.e. ? is a string with the habitat name
       [
         if content > 0
         [
           set content precision content 3  ; content here represents the relative
                       ; abundance of a flower species in the current habitat type
           let speciesNameString (word "[" "\"" item column headerList "\"" )
           set currentFlowerList
               lput (word speciesNameString " " content "]") currentFlowerList
         ]
       ]
     ]

    if nameOfCurrentList = "FlowerSpeciesList_R"
       [
         set FlowerSpeciesList_R currentFlowerList
         if habitatType != "undefined" [ set Patchtype_R (word "\"" habitatType "\"") ]
       ]
    if nameOfCurrentList = "FlowerSpeciesList_Y"
      [
        set FlowerSpeciesList_Y currentFlowerList
        if habitatType != "undefined" [ set Patchtype_Y (word "\"" habitatType "\"") ]
      ]
    if nameOfCurrentList = "FlowerSpeciesList_G"
      [
        set FlowerSpeciesList_G currentFlowerList
        if habitatType != "undefined" [ set Patchtype_G (word "\"" habitatType "\"") ]
      ]
    if nameOfCurrentList = "FlowerSpeciesList_B"
      [
        if habitatType != "undefined" [ set Patchtype_B (word "\"" habitatType "\"") ]
        set FlowerSpeciesList_B currentFlowerList
      ]
    if nameOfCurrentList = "FlowerSpeciesList_BR"
      [
        set FlowerSpeciesList_BR currentFlowerList
        if habitatType != "undefined" [ set Patchtype_BR (word "\"" habitatType "\"") ]
      ]
    if nameOfCurrentList = "FlowerSpeciesList_T"
     [
       set FlowerSpeciesList_T currentFlowerList
       if habitatType != "undefined" [ set Patchtype_T (word "\"" habitatType "\"") ]
     ]
    if nameOfCurrentList = "FlowerSpeciesList_V"
     [
```

```
      set FlowerSpeciesList_V currentFlowerList
      if habitatType != "undefined" [ set Patchtype_V (word "\"" habitatType "\"") ]
    ]
   if nameOfCurrentList = "FlowerSpeciesList_M"
    [
      set FlowerSpeciesList_M currentFlowerList
      if habitatType != "undefined" [ set Patchtype_M (word "\"" habitatType "\"") ]
    ]
   if nameOfCurrentList = "FlowerSpeciesList_P"
    [
      set FlowerSpeciesList_P currentFlowerList
      if habitatType != "undefined" [ set Patchtype_P (word "\"" habitatType "\"") ]
    ]
  ]
end
```

MoveBeesProc

The new bees-own variable "distanceFromColony_m" is calculated

```
; actual MOVEMENT:
 ask bees with [ nTrips <= MaxTrips ]
  [
    ...
    set distanceFromColony_m (distancexy Col_X Col_Y) * Scaling
```

BumbleworldSetFlowerSpeciesProc

This is a new procedure to update the flowerspeciesLists of each each patchStatistics:

```
to BumbleworldSetFlowerSpeciesProc
ask patchStatistics
[
  if patchColor = Red [ set flowerSpeciesList FlowerSpeciesList_R ]
  if patchColor = Yellow [ set flowerSpeciesList FlowerSpeciesList_Y ]
  if patchColor = Green  [ set flowerSpeciesList FlowerSpeciesList_G ]
  if patchColor = Blue  [ set flowerSpeciesList FlowerSpeciesList_B ]
  if patchColor = Brown [ set flowerSpeciesList FlowerSpeciesList_BR ]
  if patchColor = Turquoise  [ set flowerSpeciesList FlowerSpeciesList_T ]
  if patchColor = Violet [ set flowerSpeciesList FlowerSpeciesList_V ]
  if patchColor = Magenta [ set flowerSpeciesList FlowerSpeciesList_M ]
  if patchColor = Pink [ set flowerSpeciesList FlowerSpeciesList_P ]
]
end
```

BumbleworldOutProc

This is a new procedure to create input files for the bumblebee model Bumble-BEEHAVE:

```
to BumbleworldOutProc
 BumbleworldSetFlowerSpeciesProc
 ask (turtle-set Patchstatistics bees hives) [ hide-turtle]
 export-view  BumbleworldImage_Filename
 if file-exists? Bumbleworld_Inputfile [ file-delete Bumbleworld_Inputfile ]
 file-open Bumbleworld_Inputfile
 file-print Scaling
 file-print count patchStatistics
 file-print "id patchType patchColour xcor ycor size_sqm quantityPollen_g proteinPollenProp
quantityNectar_l concentration_mol/l startDay stopDay corollaDepth_mm nectarFlowerVolume_myl
intFlowerTime_s flowerSpeciesList info"

 foreach sort patchStatistics
```

```
 [
   ask ?
   [
     file-type who file-type " "
     file-type patchType file-type " "
     file-type color + 1 file-type " "
     file-type precision xcor 3 file-type " "
     file-type precision ycor 3 file-type " "
     file-type precision areaSqm 1 file-type " "
     file-type precision quantityPollen_g 0 file-type " "
     file-type precision proteinPollenProp 3 file-type " "
     file-type precision quantityNectar_l 3 file-type " "
     file-type concentration file-type " "
     file-type startDay file-type " "
     file-type stopDay file-type " "
     file-type corollaDepth_mm file-type " "
     file-type nectarFlowerVolume_myl file-type " "
     file-type interFlowerTime_s  file-type " "
     file-type flowerSpeciesList  file-type " "

     file-write patchInfo ; printed as string i.e. with " "
     file-print (" ")
   ]
 ]
 file-close
end
```

Default_ButtonProc

The new, user-defined variables are added to Default_ButtonProc, so that they are to their default values when the button "Default (Honeybees)" is pressed:

```
  set   AutoName  TRUE
  set   Bumbleworld_Inputfile  "No input _Patches.txt"
  set   BumbleworldImage_Filename  "No input _View.png"
  set   Corolla_B  5
  set   Corolla_BR  5
  set   Corolla_G  5
  set   Corolla_M  5
  set   Corolla_P  5
  set   Corolla_R  5
  set   Corolla_T  5
  set   Corolla_V  5
  set   Corolla_Y  5
  set   FlowerVol_B  4
  set   FlowerVol_BR  4
  set   FlowerVol_G  4
  set   FlowerVol_M  4
  set   FlowerVol_P  4
  set   FlowerVol_R  4
  set   FlowerVol_T  4
  set   FlowerVol_V  4
  set   FlowerVol_Y  4
  set   HabitatsInput  "BS-Habitats_Herts.csv"
  set   HighResolution FALSE
  set   IntFlowerT_B  1.54   ; Pyke (1978): inter flower time: 1.54s, (Oecologia 34, 255-266)
  set   IntFlowerT_BR  1.54
  set   IntFlowerT_G  1.54
  set   IntFlowerT_M  1.54
  set   IntFlowerT_P  1.54
  set   IntFlowerT_R  1.54
  set   IntFlowerT_T  1.54
  set   IntFlowerT_V  1.54
  set   IntFlowerT_Y  1.54
  set   Protein_B  0.2   ; Hrassnig & Crailsheim 2005: assume 20% protein in pollen,
                          ; (Apidologie 36, 255-277)
  set   Protein_BR  0.2
  set   Protein_G  0.2
  set   Protein_M  0.2
  set   Protein_P  0.2
  set   Protein_R  0.2
  set   Protein_T  0.2
  set   Protein_V  0.2
  set   Protein_Y  0.2
  set   TextMap  "BS-Map-T_Herts1.txt"
```

Complete code (tracked changes)

```
extensions [ matrix csv ]

breed [hives hive]

breed [bees bee]

breed [patchStatistics patchStatistic ]

bees-own
[
  destinationList
  flightPhase
  furthestDistance_m
  furthestLocationList
  knownPatchesList
  lastDestinationList
  lastLocationList
  nowDetectedPatchesList
  nTrips
  time_s

  distanceFromColony_m  ; new ***MB*** 2015-10-06 for BEESCOUT 2.0
]


patches-own
```

[
flowerPatchID
firstPatchOfFlowerpatch
mapDisplay
originalColor
patchColor
habitatTypePatch
satelliteColor
visits
]

patchStatistics-own
[
areaPx
areaSqm
calculatedDetectionProb_per_trip
closestDistance_m
concentration
detects1000List
firstDetection_s
maxDetectProb_per_s
meanDistance_m
modelledDetectionProb_per_s
modelledDetectionProb_per_trip
nectarGathering_s
nNectarVisits
nPollenVisits
patchType
pollenGathering_s
quantityNectar_l
quantityPollen_g
startDay
stopDay

```
  timeMaxDetectProb_s
  totalDetections
  visitColor

  corollaDepth_mm ; ***MB*** new for BEESCOUT 2.0
  nectarFlowerVolume_myl ; ***MB*** new for BEESCOUT 2.0
  interFlowerTime_s ; ***MB*** new for BEESCOUT 2.0
  flowerSpeciesList ; ***MB*** new for BEESCOUT 2.0
  patchInfo ; ***MB*** new for BEESCOUT 2.0, short string with additional information for the user
  proteinPollenProp ; ***MB*** new for BEESCOUT 2.0
]


globals
[
  BackgroundColor
  BorderColor
  BottomBorder
  ColourCodeList
  ForagingFileList
  Gap_s
  LakeColor
  LeftBorder
  MaxHiveDisplAllBees_m
  MixedStratCol
  MixedStratInd
  NewTimestepForaging
  Npatches
  OutputFileList
  OutputWordResult
  PatchColoursList
  Repetitions
  RightBorder
```

```
  Scaling
  TopBorder
  TotalTrips
  WriteToFile

  ; new ***MB*** 2015-10-06 for BEESCOUT 2.0:
  HabitatDataCSV
  FlowerSpeciesList_R FlowerSpeciesList_Y FlowerSpeciesList_G FlowerSpeciesList_B FlowerSpeciesList_BR FlowerSpeciesList_T FlowerSpeciesList_V FlowerSpeciesList_M FlowerSpeciesList_P

]

;; ==============================================================================================================================================
```

to Setup

```
 ; Setup Procedure  ; RUN A PROCEDURE


  __clear-all-and-reset-ticks
 ;if RandSeed > 0 [ random-seed RandSeed ]

   ; ***MB*** new for BEESCOUT 2.0
  ifelse HighResolution = false ;
  [
     resize-world 0 300 0 210  ; min-pxcor max-pxcor min-pycor max-pycor
     set-patch-size 5
  ]
  [
     set-patch-size 1
```

```
    resize-world 0 1500 0 1050
  ]
  if AutoName = true
  [
    ifelse InputFile = "TextMap"
    [
      let name1 substring TextMap 0 (length TextMap - 4)
      set BumbleBeehaveImage_Filename (word name1 "_View.png")
      set BumbleBeehave_Inputfile (word name1 "_Patches.txt")
      if substring BumbleBeehave_Inputfile 0 9 = "BS-Map-T_"
      [
        set BumbleBeehaveImage_Filename remove "BS-Map-T_" BumbleBeehaveImage_Filename
        set BumbleBeehaveImage_Filename remove "_View" BumbleBeehaveImage_Filename
        set BumbleBeehaveImage_Filename (word "BBH-I_" BumbleBeehaveImage_Filename)
        set BumbleBeehave_Inputfile remove "BS-Map-T_" BumbleBeehave_Inputfile
        set BumbleBeehave_Inputfile remove "_Patches" BumbleBeehave_Inputfile
        set BumbleBeehave_Inputfile (word "BBH-T_" BumbleBeehave_Inputfile)
      ]
      if substring BumbleBeehave_Inputfile 0 15 = "SI_07_BS-Map-T_"
      [
        set BumbleBeehaveImage_Filename remove "SI_07_BS-Map-T_" BumbleBeehaveImage_Filename
        set BumbleBeehaveImage_Filename remove "_View" BumbleBeehaveImage_Filename
        set BumbleBeehaveImage_Filename (word "SI_07_BBH-I_" BumbleBeehaveImage_Filename)
        set BumbleBeehave_Inputfile remove "SI_07_BS-Map-T_" BumbleBeehave_Inputfile
        set BumbleBeehave_Inputfile remove "_Patches" BumbleBeehave_Inputfile
        set BumbleBeehave_Inputfile (word "SI_07_BBH-T_" BumbleBeehave_Inputfile)
      ]
    ]
    [
      let name1 substring InputFile 0 (length InputFile - 4)
      set BumbleBeehaveImage_Filename (word name1 "_View.png")
      set BumbleBeehave_Inputfile (word name1 "_Patches.txt")
    ]
```

```
 ]
 ; END  ***MB*** new for BEESCOUT 2.0

 if RandSeed != 0 [ random-seed RandSeed ]
 set OutputFileList []  ;; list, in which the results for the output file will be written
 set Gap_s 3 ; 3s: gap between 2 recordings of a bee in radar data Rothamsted - and hence also duration of a "tick" (timestep)
 set MixedStratInd false
 set MixedStratCol false

 set LakeColor cyan
 set BorderColor 125.12345678987654 ; a unique color most likely not occuring on the map (~magenta)
 set OutputWordResult ""
 create-hives 1
   [
     set color 33
     set shape "square"
     set size 4
     setxy Col_X Col_Y
   ]
 ask patches [ set pcolor BorderColor ] ; pcolor set to a unique color, to determine the borders of the map
 set PatchColoursList []
 set FlowerSpeciesList_R []
 set FlowerSpeciesList_Y []
 set FlowerSpeciesList_G []
 set FlowerSpeciesList_B []
 set FlowerSpeciesList_BR []
 set FlowerSpeciesList_T []
 set FlowerSpeciesList_V []
 set FlowerSpeciesList_M []
 set FlowerSpeciesList_P []
 if RedPatches = true [ set PatchColoursList fput red PatchColoursList ]
 if GreenPatches = true [ set PatchColoursList fput green PatchColoursList ]
 if YellowPatches = true [ set PatchColoursList fput yellow PatchColoursList ]
```

```
 if BluePatches = true [ set PatchColoursList fput blueBlue PatchColoursList ]
 if TurquoisePatches = true [ set PatchColoursList fput Turquoise PatchColoursList ] ; Grassland Pasture
 if VioletPatches = true [ set PatchColoursList fput Violet PatchColoursList ]; Gardens
 if BrownPatches = true [ set PatchColoursList fput Brown PatchColoursList ] ; Woodland
 if MagentaPatches = true [ set PatchColoursList fput Magenta PatchColoursList ] ; Semi-natural Scrub
 if PinkPatches = true [ set PatchColoursList fput Pink PatchColoursList ] ; Semi-natural Grassland
 set Scaling ScaleDistance_m / (Scale_X2 - Scale_X1) ;; real distance [m] divided by distance of grid points

  ifelse InputFile != "No input file"
   [
     ImportMapProc                ;;loads the crop map    ; CALL A PROCEDURE
     ifelse InputFile = "TextMap"
     [
       ReadMapFromTextProc
       if count patches with [ originalColor = BorderColor ] > 0 [ DetermineBordersProc ]  ; CALL A PROCEDURE
     ]
     [
       ImportMapProc                ;;loads the crop map    ; CALL A PROCEDURE
       if count patches with [ originalColor = BorderColor ] > 0 [ DetermineBordersProc ]  ; CALL A PROCEDURE
     ]
    ]
   [
     ask patches [   ; ELSE (if there is no input file):
              set pcolor grey
              set patchColor grey
              set originalColor grey
             ]
   ]



 if SatelliteFile != "No satellite image"
   [
```

```
      import-pcolors SatelliteFile
      ask patches [
                   set satelliteColor pcolor
                   set pcolor patchcolor
                ]
    ]

  set WriteToFile false

  AnalyseProc   ; CALL A PROCEDURE
  ask patches [set pcolor grey ]
  ask patches with [  member? patchColor PatchColoursList = true] [ set pcolor patchColor ]
  if Lakes = true [ ask patches with [ patchColor = LakeColor ] [ set pcolor LakeColor ] ]
  if count patches with [ pcolor = LakeColor ] / (max-pxcor * max-pycor) > 0.5
    [ user-message "There are a lot of lakes on your map!" ]

end

;;
==================================================================================================================================================================
```

to DetermineBordersProc

```
  set TopBorder min [ pycor ] of patches with [(pycor > (max-pycor / 2)) and (originalColor = BorderColor)]      ; determine the borders of the world: top ..
  set BottomBorder max [ pycor ] of patches with [(pycor < (max-pycor / 2)) and (originalColor = BorderColor)]    ;   ...and bottom
  if TopBorder - BottomBorder <= 2
     [
      set TopBorder max-pycor + 1      ; i.e. outside the "world"
      set BottomBorder min-pycor - 1
     ]

  set RightBorder max-pxcor + 1
```

```
  if count patches with [ (pxcor > (max-pxcor / 2)) and (originalColor = BorderColor)] > 0      ; determine the borders of the world: right ..
   [ set RightBorder min [ pxcor ] of patches with [(pxcor > (max-pxcor / 2)) and (originalColor = BorderColor)]    ; determine the borders of the world: right
..]

  set LeftBorder min-pxcor - 1
  if count patches with [(pxcor < (max-pxcor / 2)) and (originalColor = BorderColor)] > 0
    [ set LeftBorder max [ pxcor ] of patches with [(pxcor < (max-pxcor / 2)) and (originalColor = BorderColor)])]]      ;   ...and left
  if RightBorder - LeftBorder <= 2
    [
     set RightBorder max-pxcor + 1   ; i.e. outside the "world"
     set LeftBorder min-pxcor - 1
    ]

  ask patches with [(pycor = TopBorder) or (pycor = BottomBorder) or (pxcor = LeftBorder) or (pxcor = RightBorder)]
    [ set originalColor BorderColor ]

  ask patches with [ originalColor = BorderColor ]  [ set pcolor BorderColor ]
end

;;
==================================================================================================================================================================================

to ReadMapFromTextProc
 ; let colourCodeList [ brown magenta violet pink white turquoise white red yellow green ]
;let colourCodeList [ pink  white  turquoise  brown  white  white  brown  white  pink  white  pink  green  white  white  white  brown  brown  brown  brown
violet  pink  magenta  turquoise  yellow  white  red  white  white  white  white  white  ]

let nextColourType -1

file-open TextMap
let ncols read-from-string file-read-line   ; reads in number of columns
let nrows read-from-string file-read-line   ; reads in number of rows
```

```
set Scaling read-from-string file-read-line ; reads in the scaling of the map (size [m] of each grid cell)
set Scale_X1 1
set Scale_X2 ncols
set ScaleDistance_m Scaling * (Scale_X2 - Scale_X1)
set ColourCodeList read-from-string file-read-line
if ncols > max-pxcor or nrows > max-pycor [ user-message "TextMap does not match size of BEESCOUT world" ]

; ***MB*** 2016-07-21
; place map in the centre:
let leftMargin (max-pxcor - ncols) / 2
let bottomMargin (max-pycor - nrows) / 2


foreach sort patches [
 ask ?
 [
  if (pxcor >= leftMargin and pxcor < ncols + leftMargin) and (pycor >= bottomMargin and pycor < nrows + bottomMargin) and file-at-end? = false
  [
   set nextColourType file-read
   ifelse nextColourType > 0
   [
    set habitatTypePatch nextColourType
    set pcolor item (nextColourType - 1) colourCodeList
   ]

   [ set pcolor grey ]
  ]
 ]
]
file-close

ask patches
 [
```

```
    set originalColor pcolor  ;; the color of the (grid) patch in the original file
    set plabel-color black
    set flowerPatchID -1
    set patchColor pcolor  ;; saves the color after rounding
  ]

end

;; ==========================================================================
```

to ImportMapProc

```
;;loads the crop map, corrects colours    ; RUN A PROCEDURE

  import-pcolors InputFile
  ask patches
  [
    set originalColor pcolor  ;; the color of the (grid) patch in the original file
    set plabel-color black
    set flowerPatchID -1
    if remainder pcolor 10 < Black_th [ set pcolor black ]
    if remainder pcolor 10 > White_th [ set pcolor white ]
    if (pcolor > Red_min) and (pcolor <= Red_max) and (pcolor != BorderColor) [ set pcolor red ]
    if (pcolor > Yellow_min) and (pcolor <= Yellow_max) and (pcolor != BorderColor) [ set pcolor yellow ]
    if (pcolor > Green_min) and (pcolor <= Green_max) and (pcolor != BorderColor) [ set pcolor green ]
    if (pcolor > Blue_min) and (pcolor <= Blue_max) and (pcolor != BorderColor) [ set pcolor blueBlue ] ; Rothamsted
    if (pcolor > Turquoise_min) and (pcolor <= Turquoise_max) and (pcolor != BorderColor) [ set pcolor Turquoise ] ;Grassland Pasture
    if (pcolor > Violet_min) and (pcolor <= Violet_max) and (pcolor != BorderColor) [ set pcolor Violet ] ;Gardens
    if (pcolor > Brown_min) and (pcolor <= Brown_max) and (pcolor != BorderColor) [ set pcolor Brown ] ;Woodland
    if (pcolor > Magenta_min) and (pcolor <= Magenta_max) and (pcolor != BorderColor) [ set pcolor Magenta ] ;Semi-natural Scrub
    if (pcolor > Pink_min) and (pcolor <= Pink_max) and (pcolor != BorderColor) [ set pcolor Pink ] ;Semi-natural Grassland
    set patchColor pcolor  ;; saves the color after rounding
  ]
end
```

;;
===

to AnalyseProc

```
;; determination of flower patches, calls procedures to create scout bees, and exploration of landscape   ; RUN A PROCEDURE
  clear-turtles
  let currentColor 0
  let currentPatchID -1
  let flowerPatchCounter 0
  set Repetitions round (MaxPatchRadius_m / Scaling)
    ; # of repetition depends on the scale of the landscape

  foreach sort patches
  [
    ask ?   ;; determines flowerpatches: coloured (r,b,y,g) (nlogo-)patches with ID -1 are searched, it gets a new ID and all connected (nlogo-)patches with the same colour get the same ID
     [
      if member? patchColor PatchColoursList
      [
        if flowerPatchID = -1 ;; if patch is not identified yet
        [
          set flowerPatchID (flowerPatchCounter)
          set currentColor pcolor ;; colour of the flower patch is the colour of the "firstPatchOfFlowerpatch"
          set firstPatchOfFlowerpatch true
          set currentPatchID flowerPatchID
          let search-patches patches in-radius Repetitions  ;; NEW ***VG***
          repeat Repetitions
          [
           ; ask patches  with [(pcolor = currentColor) and (flowerPatchID = currentPatchID)] ;; connected (nlogo-)patches around the firstPatchOfFlowerpatch are searched defined as part of this flower patch
```

```
      ; NEW ***VG*** NEW:
       ask search-patches with [(pcolor = currentColor) and (flowerPatchID = currentPatchID)] ;; connected (nlogo-)patches around the firstPatchOfFlowerpatch are searched defined as part of this flower patch
       [
        ask neighbors with [(pcolor = currentColor) and (flowerPatchID = -1)]
          [ set flowerPatchID currentPatchID ]
        ]
      ]
      set flowerPatchCounter flowerPatchCounter + 1
      ]
     ]
     set pcolor lime  ; to show progress of map analysis
   ]
 ]
 set Npatches currentPatchID + 1  ; as 1st patch has id 0
 CreatePatchStatisticsProc ;; creates "patchStatistics" (turtles) to store data of the flower patches   ; CALL A PROCEDURE
 create-hives 1
  [
   set color 33
   set shape "square"
   set size 4
   setxy Col_X Col_Y
  ]
 CreateBeesProc   ;; creates scouts (turtles)   ; CALL A PROCEDURE
 DetermineSizeProc                              ; CALL A PROCEDURE
 if Lakes = true
  [
   ask patches with [patchColor = black ]
     [ set patchColor LakeColor ]
  ]

 if count patchstatistics > 0
```

```
  [
    if Plot1 > count patchstatistics - 1
      or Plot2 > count patchstatistics - 1
      or Plot3 > count patchstatistics - 1
      or Plot4 > count patchstatistics - 1
      [
        set Plot1 min [ who ] of patchstatistics
        set Plot2 round ((mean [ who ] of patchstatistics + min [ who ] of patchstatistics) / 2)
        set Plot3 round ((mean [ who ] of patchstatistics + max [ who ] of patchstatistics) / 2)
        set Plot4 max [ who ] of patchstatistics
      ]
  ]

end

;;
==============================================================================================================
==========================================
```

to CreatePatchStatisticsProc

```
;; "patchStatistics": turtles, containing the relevant infos of the flowerPatches, slightly different colour, located within the flowerPatch   ; RUN A PROCEDURE
let currentXcor 0
let currentYcor 0
let currentWho 0
let currentColor 0
create-PatchStatistics Npatches
    [
      set size 12
      set shape "circle"
      set currentWho Who
```

```
; new ***MB*** 2015-10-06 for BEESCOUT 2.0:
set patchInfo "no info"
set xcor mean [ pxcor ] of patches with [ flowerPatchID = currentWho ]
set ycor mean [ pycor ] of patches with [ flowerPatchID = currentWho ]
set currentColor patchcolor

if currentWho != flowerPatchID
 [
   ask min-one-of patches with [ flowerPatchID = currentWho ] [distance myself]
   [
     set currentXcor pxcor
     set currentYcor pycor
     set currentColor patchcolor
   ]
   setxy currentXcor currentYcor
 ]

set color currentColor - 1
set label-color white
;if color = black [ set label-color white]
set label who
set modelledDetectionProb_per_s 0
set modelledDetectionProb_per_trip 0
set totalDetections 0
set timeMaxDetectProb_s 0
set detects1000List []
set maxDetectProb_per_s 0
set flowerSpeciesList []
if patchColor = red
 [
  set patchType PATCHTYPE_R
  set concentration Conc_R
  set nectarGathering_s t_Nectar_R
```

```
        set pollenGathering_s t_Pollen_R
        set startDay Start_R
        set stopDay Stop_R
        set corollaDepth_mm Corolla_R   ; ***MB*** new for BEESCOUT 2.0
        set nectarFlowerVolume_myl FlowerVol_R  ; ***MB*** new for BEESCOUT 2.0
        set interFlowerTime_s intFlowerT_R      ; ***MB*** new for BEESCOUT 2.0
        set proteinPollenProp Protein_R         ; ***MB*** new for BEESCOUT 2.0

       ]
      if patchColor = blueBlue
       [
        set patchType PATCHTYPE_B
        set concentration Conc_B
        set nectarGathering_s t_Nectar_B
        set pollenGathering_s t_Pollen_B
        set startDay Start_B
        set stopDay Stop_B
        set corollaDepth_mm Corolla_B   ; ***MB*** new for BEESCOUT 2.0
        set nectarFlowerVolume_myl FlowerVol_B  ; ***MB*** new for BEESCOUT 2.0
        set interFlowerTime_s intFlowerT_B      ; ***MB*** new for BEESCOUT 2.0
        set flowerSpeciesList FlowerSpeciesList_B    ; ***MB*** new for BEESCOUT 2.0
        set proteinPollenProp Protein_B         ; ***MB*** new for BEESCOUT 2.0
       ]
      if patchColor = yellow
       [
        set patchType PATCHTYPE_Y
        set concentration Conc_Y
        set nectarGathering_s t_Nectar_Y
        set pollenGathering_s t_Pollen_Y
        set startDay Start_Y
        set stopDay Stop_Y
        set corollaDepth_mm Corolla_Y   ; ***MB*** new for BEESCOUT 2.0
        set nectarFlowerVolume_myl FlowerVol_Y  ; ***MB*** new for BEESCOUT 2.0
```

```
        set interFlowerTime_s intFlowerT_Y      ; ***MB*** new for BEESCOUT 2.0
        set proteinPollenProp Protein_Y         ; ***MB*** new for BEESCOUT 2.0

      ]
      if patchColor = green
      [
        set patchType PATCHTYPE_G
        set concentration Conc_G
        set nectarGathering_s t_Nectar_G
        set pollenGathering_s t_Pollen_G
        set startDay Start_G
        set stopDay Stop_G
        set corollaDepth_mm Corolla_G   ; ***MB*** new for BEESCOUT 2.0
        set nectarFlowerVolume_myl FlowerVol_G  ; ***MB*** new for BEESCOUT 2.0
        set interFlowerTime_s intFlowerT_G      ; ***MB*** new for BEESCOUT 2.0
        set flowerSpeciesList FlowerSpeciesList_G    ; ***MB*** new for BEESCOUT 2.0
        set proteinPollenProp Protein_G         ; ***MB*** new for BEESCOUT 2.0
      ]
      if patchColor = Turquoise ; Grassland Pasture
      [
        set patchType PATCHTYPE_T
        set concentration Conc_T
        set nectarGathering_s t_Nectar_T
        set pollenGathering_s t_Pollen_T
        set startDay Start_T
        set stopDay Stop_T
        set corollaDepth_mm Corolla_T   ; ***MB*** new for BEESCOUT 2.0
        set nectarFlowerVolume_myl FlowerVol_T  ; ***MB*** new for BEESCOUT 2.0
        set interFlowerTime_s intFlowerT_T      ; ***MB*** new for BEESCOUT 2.0
        set proteinPollenProp Protein_T         ; ***MB*** new for BEESCOUT 2.0

      ]
      if patchColor = Violet ;Gardens
```

```
    [
      set patchType PATCHTYPE_v
      set concentration Conc_V
      set nectarGathering_s t_Nectar_V
      set pollenGathering_s t_Pollen_V
      set startDay Start_V
      set stopDay Stop_V
      set corollaDepth_mm Corolla_V  ; ***MB*** new for BEESCOUT 2.0
      set nectarFlowerVolume_myl FlowerVol_V ; ***MB*** new for BEESCOUT 2.0
      set interFlowerTime_s intFlowerT_V     ; ***MB*** new for BEESCOUT 2.0
      set proteinPollenProp Protein_V        ; ***MB*** new for BEESCOUT 2.0

    ]
   if patchColor = Brown ;Woodland
    [
      set patchType PATCHTYPE_BR
      set concentration Conc_BR
      set nectarGathering_s t_Nectar_BR
      set pollenGathering_s t_Pollen_BR
      set startDay Start_BR
      set stopDay Stop_BR
      set corollaDepth_mm Corolla_BR  ; ***MB*** new for BEESCOUT 2.0
      set nectarFlowerVolume_myl FlowerVol_BR ; ***MB*** new for BEESCOUT 2.0
      set interFlowerTime_s intFlowerT_BR     ; ***MB*** new for BEESCOUT 2.0
      set proteinPollenProp Protein_BR        ; ***MB*** new for BEESCOUT 2.0

    ]
   if patchColor = Magenta ;Semi-natural Scrub
    [
      set patchType PATCHTYPE_M
      set concentration Conc_M
      set nectarGathering_s t_Nectar_M
      set pollenGathering_s t_Pollen_M
```

```
        set startDay Start_M
        set stopDay Stop_M
        set corollaDepth_mm Corolla_M   ; ***MB*** new for BEESCOUT 2.0
        set nectarFlowerVolume_myl FlowerVol_M  ; ***MB*** new for BEESCOUT 2.0
        set interFlowerTime_s intFlowerT_M      ; ***MB*** new for BEESCOUT 2.0
        set proteinPollenProp Protein_M         ; ***MB*** new for BEESCOUT 2.0

      ]
      if patchColor = Pink
      [
        set patchType PATCHTYPE_P
        set concentration Conc_P
        set nectarGathering_s t_Nectar_P
        set pollenGathering_s t_Pollen_P
        set startDay Start_P
        set stopDay Stop_P
        set corollaDepth_mm Corolla_P   ; ***MB*** new for BEESCOUT 2.0
        set nectarFlowerVolume_myl FlowerVol_P  ; ***MB*** new for BEESCOUT 2.0
        set interFlowerTime_s intFlowerT_P      ; ***MB*** new for BEESCOUT 2.0
        set proteinPollenProp Protein_P         ; ***MB*** new for BEESCOUT 2.0

      ]
    ]
end

;;
==============================================================================================================================================================
```

to-report CalculateDetectProbREP

```
;; calculates the detection probability of a flower patch based on distance; see Simulation 2 in the BEESCOUT publication  ; RUN A PROCEDURE
  let lambda -0.00073 ; see BEESCOUT publication, Tab. 1, fit to "random location" search mode
```

```
  report e ^ (lambda * meanDistance_m)
end

;;
==================================================================================================
=================================================
```

to DetermineSizeProc

```
 ;; determines the size and distance to colony of the flower patches, writes data in short list and in output list   ; RUN A PROCEDURE
 let currentWho 0
 let shortList []
 ;;ask patchStatistics [
 foreach sort patchStatistics [ ask ? [
   if HighResolution = true [ type "Progress in DetermineSizeProc [%]: " print precision (100 * who / count patchStatistics) 1 ] ;  ; ***MB*** new for
BEESCOUT 2.0

  let sumDistance 0
  let currentClosestDistanceM 999999 ;; Creation of LIST for OUTPUT file:
  set currentWho Who
  set areaPx (count patches with [flowerPatchID = currentWho])
  ask patches with [flowerPatchID = currentWho]
   [
    set sumDistance sumDistance + distancexy Col_X Col_Y
    if distancexy Col_X Col_Y < currentClosestDistanceM
      [ set currentClosestDistanceM distancexy Col_X Col_Y ]
   ]
  set areaSqm round(areaPx * Scaling * Scaling)

  if color = red - 1
     [
      set quantityNectar_l (Nectar_R / 1000) * areaSqm  ; (Nectar_R: ml)
```

```
        set quantityPollen_g Pollen_R * areaSqm
      ]
  if color = green - 1
      [
        set quantityNectar_l (Nectar_G / 1000) * areaSqm  ; (Nectar_G: ml)
        set quantityPollen_g Pollen_G * areaSqm
      ]
  if color = yellow - 1
      [
        set quantityNectar_l (Nectar_Y / 1000) * areaSqm  ; (Nectar_Y: ml)
        set quantityPollen_g Pollen_Y * areaSqm
      ]
  if color = blueBlue - 1
      [
        set quantityNectar_l (Nectar_B / 1000) * areaSqm  ; (Nectar_B: ml)
        set quantityPollen_g Pollen_B * areaSqm
      ]
  if color = Turquoise - 1 ; Grassland Pasture
      [
        set quantityNectar_l (Nectar_T / 1000) * areaSqm  ; (Nectar_T: ml)
        set quantityPollen_g Pollen_T * areaSqm
      ]
  if color = Violet - 1 ; Gardens
      [
        set quantityNectar_l (Nectar_V / 1000) * areaSqm  ; (Nectar_V: ml)
        set quantityPollen_g Pollen_V * areaSqm
      ]
  if color = Brown - 1 ; Woodland
      [
        set quantityNectar_l (Nectar_BR / 1000) * areaSqm  ; (Nectar_W: ml)
        set quantityPollen_g Pollen_BR * areaSqm
      ]
  if color = Magenta - 1 ; Semi-natural Scrub
```

```
    [
       set quantityNectar_l (Nectar_M / 1000) * areaSqm  ; (Nectar_M: ml)
       set quantityPollen_g Pollen_M * areaSqm
    ]
  if color = Pink - 1 ; Semi-natural Grassland
    [
       set quantityNectar_l (Nectar_P / 1000) * areaSqm  ; (Nectar_P: ml)
       set quantityPollen_g Pollen_P * areaSqm
    ]
  set meanDistance_m precision((sumDistance / areaPx) * Scaling) 1  ;; [m]
  set closestDistance_m precision (currentClosestDistanceM * Scaling) 1  ;; [m]
  if closestDistance_m < 0.1 [ set closestDistance_m 0.1 ]  ; to avoid division by 0
  set calculatedDetectionProb_per_trip CalculateDetectProbREP

  ;; Headline (added in the "WriteFromListToFileProc"):  1. who 2. xcor 3. ycor 4. patchType 5. closestDistanceM 6. areaSqm 7. modelledDetectProb
  set shortList lput Who shortList              ;; 1st column (who)
  set shortList lput xcor shortList             ;; 2nd column (xcor)
  set shortList lput ycor shortList             ;; 3rd column (ycor)
  set shortList lput patchType shortList        ;; 4th column (patchType)
  set shortList lput closestDistance_m shortList  ;; 5th column (closestDistance)
  set shortList lput areaSqm shortList          ;; 6th column (area)
  ;;                                            ;; 7th column: modelledDetectionProb - is added in the "WriteFromListToFileProc"
  set OutputFileList lput shortList OutputFileList
  set shortList []
  ] ]
end

;;
==================================================================================================
=====================================================
```

to CreateBeesProc

```
;; creates scouts to explore the landscape and to determine the detection probabilities of the flower patches   ; RUN A PROCEDURE
 ask bees [ die ]
 set-current-plot "Detection Probability per trip" clear-plot
 ask patches [ set visits 0 ]
 ask patchstatistics                    ; flower pacthes have to be reset
  [
    set modelledDetectionProb_per_s 0
    set modelledDetectionProb_per_trip 0
    ;set maxDetectProb_per_s 0
    set totalDetections 0
    set firstDetection_s 0
    set detects1000List []
    set maxDetectProb_per_s 0
    set timeMaxDetectProb_s 0
  ]
 create-bees N_Bees
   [
     setxy Col_X Col_Y
     set destinationList []
     set lastDestinationList list xcor ycor ; []
     set furthestLocationList list xcor ycor
     set furthestDistance_m 0
     set lastLocationList list xcor ycor
     set shape "bee_mb_1"
     set color white    ; bees start in the "search" mode (flightPhase 2) when they first leave the hive
     set flightPhase 2
     set size 5    ; beesize
     set knownPatchesList []
     set nTrips 1 ; 0
     set nowDetectedPatchesList []
   ]
 set MixedStratInd false
```

```
  set MixedStratCol false
end

;;
================================================================================================================================================================

to DefineHabitatsProc

ifelse file-exists? HabitatsInput
[
 set HabitatDataCSV csv:from-file HabitatsInput   ; the csv file contains information about which habitat types are represented by which colour and the relative abundance of flower species
 let headerList item 0 HabitatDataCSV   ; the header of the csv table
 let line 0

 foreach but-first HabitatDataCSV ; goes through all 'lines' (except of header) in ordered way
  [
    set line line + 1
    let currentFlowerList []
    let habitatType "none"
    let column 0
    let nameOfCurrentList first ?
    foreach but-first ? ; goes through all 'columns' of this 'line'
    [
      set column column + 1
      let content ? ; the content of a single 'cell'
      ifelse is-string? content = true ;
       [ set habitatType ? ] ; i.e. ? is a string with the habitat name
       [
         if content > 0
```

```
      [
        set content precision content 3  ; content here represents the relative abundance of a flower species in the current habitat type
        let speciesNameString (word "[" "\"" item column headerList "\"" )
        set currentFlowerList lput (word speciesNameString " " content "]") currentFlowerList

      ]
    ]
  ]

  if nameOfCurrentList = "FlowerSpeciesList_R"
  [
    set FlowerSpeciesList_R currentFlowerList
    if habitatType != "undefined" [ set Patchtype_R (word "\"" habitatType "\"") ]
  ]
  if nameOfCurrentList = "FlowerSpeciesList_Y"
  [
    set FlowerSpeciesList_Y currentFlowerList
    if habitatType != "undefined" [ set Patchtype_Y (word "\"" habitatType "\"") ]
  ]
  if nameOfCurrentList = "FlowerSpeciesList_G"
  [
    set FlowerSpeciesList_G currentFlowerList
    if habitatType != "undefined" [ set Patchtype_G (word "\"" habitatType "\"") ]
  ]
  if nameOfCurrentList = "FlowerSpeciesList_B"
  [
    if habitatType != "undefined" [ set Patchtype_B (word "\"" habitatType "\"") ]
    set FlowerSpeciesList_B currentFlowerList
  ]
  if nameOfCurrentList = "FlowerSpeciesList_BR"
  [
    set FlowerSpeciesList_BR currentFlowerList
    if habitatType != "undefined" [ set Patchtype_BR (word "\"" habitatType "\"") ]
```

```
    ]
    if nameOfCurrentList = "FlowerSpeciesList_T"
    [
      set FlowerSpeciesList_T currentFlowerList
      if habitatType != "undefined" [ set Patchtype_T (word "\"" habitatType "\"") ]
    ]
    if nameOfCurrentList = "FlowerSpeciesList_V"
    [
      set FlowerSpeciesList_V currentFlowerList
      if habitatType != "undefined" [ set Patchtype_V (word "\"" habitatType "\"") ]
    ]
    if nameOfCurrentList = "FlowerSpeciesList_M"
    [
      set FlowerSpeciesList_M currentFlowerList
      if habitatType != "undefined" [ set Patchtype_M (word "\"" habitatType "\"") ]
    ]
    if nameOfCurrentList = "FlowerSpeciesList_P"
    [
      set FlowerSpeciesList_P currentFlowerList
      if habitatType != "undefined" [ set Patchtype_P (word "\"" habitatType "\"") ]
    ]
  ]
 ]
[ ; ELSE: if file doesn't exist:

 ifelse HabitatsInput = "none"
 [
   set FlowerSpeciesList_R []
   set FlowerSpeciesList_Y []
   set FlowerSpeciesList_G []
   set FlowerSpeciesList_B []
   set FlowerSpeciesList_BR []
   set FlowerSpeciesList_T []
```

```
   set FlowerSpeciesList_V []
   set FlowerSpeciesList_M []
   set FlowerSpeciesList_P []
 ]
 [
   user-message (word "The specified HabitatsInput file cannot be loaded: " HabitatsInput)
 ]
 ]
end


;; ==========================================================================================================================================================
```

to Go

```
;; Go Procedure movement of scouts, detection of flower patches   ; RUN A PROCEDURE
  tick
  if (ticks * Gap_s > ScoutingPeriod_hrs * 3600) or (min [ nTrips ] of bees > MaxTrips)
    [
      set OutputWordResult (word "Npatches Who areaSqm meanDistance_m modelledDetectionProb_per_s modelledDetectionProb_per_trip "  count patchStatistics)
      foreach sort patchStatistics
      [
       ask ?
        [ ; creates output for BehaviorSpace
          set OutputWordResult (word OutputWordResult " " who " " areaSqm " " meanDistance_m " " modelledDetectionProb_per_s " " modelledDetectionProb_per_trip)
        ]
      ]
```

```
    set OutputWordResult (word OutputWordResult " END")
    stop
  ]
 if remainder ticks 1000 = 0
  [  ask patchStatistics
    [ set detects1000List lput totalDetections detects1000List ]
  ]
 MoveBeesProc   ; CALL A PROCEDURE
 if count PatchStatistics > 0 [ DoDetectionPlotsProc ]   ; CALL A PROCEDURE
end



;;
==================================================================================================================
=============================================
```

to AnalyseOutfileProc

```
 ;   (only) called by Analyse & Outfile button       ; RUN A PROCEDURE
 ; user-message ("Please wait while the button remains black. This may take a few minutes")
  Setup
  set WriteToFile true
  if is-string? NameOutfile  ;; We check to make sure we actually got a string just in case  the user hits the cancel button.
    [ if file-exists? nameOutfile   ;; If the file already exists, we begin by deleting it, otherwise new data would be appended to the old contents.
         [ file-delete nameOutfile ]
      file-open NameOutfile
     ]
  repeat ScoutingPeriod_hrs * 3600 / Gap_s
   [ Go ]
  WriteFromListToFileProc   ; CALL A PROCEDURE
  file-close
  set WriteToFile false
```

end

;;
==
===

to-report DisplacementREP

; [time]   ; RUN A PROCEDURE
let displ_m 0
 let result 0
 if BeeSpecies = "Honeybees"
   [set displ_m 2.96 * Gap_s] ; 2.96 m/s times (3s) time step (derived from harmonic radar dataset Emma Wright (PhD thesis))
 if BeeSpecies = "Bumblebees"
   [set displ_m 3.22 * Gap_s] ; 3.22 m/s times (3s) time step (derived from harmonic radar dataset, Osborne et al 2013)
 let displ_NLpatches displ_m / Scaling
 set result displ_NLpatches * DisplacementFactor   ; DisplacementFactor: slider (GUI), default: 1
 report result
end

;;
==
===

to-report TurningREP

 ; reports rt, the angle a bee turns to its RIGHT ; RUN A PROCEDURE

 let turn 0

 if BeeSpecies = "Honeybees"
 [

```
   let randBar random 628 + 1 ; randomly chosen bar from the empirical turning angle histogram with a total of 628 turning angles (returns random number between [1..628]
   ; 18 bars, each 10 degrees, assuming symmetry of turning right and left  (derived from harmonic radar dataset Emma Wright (PhD thesis))

   if randBar >= 1 and randBar < 92 [ set turn 0 + random-float 10 ]
   if randBar >= 92 and randBar < 144 [ set turn 10 + random-float 10 ]
   if randBar >= 144 and randBar < 206 [ set turn 20 + random-float 10 ]
   if randBar >= 206 and randBar < 262 [ set turn 30 + random-float 10 ]
   if randBar >= 262 and randBar < 302 [ set turn 40 + random-float 10 ]
   if randBar >= 302 and randBar < 343 [ set turn 50 + random-float 10 ]
   if randBar >= 343 and randBar < 363 [ set turn 60 + random-float 10 ]
   if randBar >= 363 and randBar < 396 [ set turn 70 + random-float 10 ]
   if randBar >= 396 and randBar < 419 [ set turn 80 + random-float 10 ]
   if randBar >= 419 and randBar < 440 [ set turn 90 + random-float 10 ]
   if randBar >= 440 and randBar < 456 [ set turn 100 + random-float 10 ]
   if randBar >= 456 and randBar < 483 [ set turn 110 + random-float 10 ]
   if randBar >= 483 and randBar < 506 [ set turn 120 + random-float 10 ]
   if randBar >= 506 and randBar < 526 [ set turn 130 + random-float 10 ]
   if randBar >= 526 and randBar < 542 [ set turn 140 + random-float 10 ]
   if randBar >= 542 and randBar < 570 [ set turn 150 + random-float 10 ]
   if randBar >= 570 and randBar < 599 [ set turn 160 + random-float 10 ]
   if randBar >= 599 and randBar <= 628 [ set turn 170 + random-float 10 ]
 ]


 if BeeSpecies = "Bumblebees"
 [
   let randBar random 214 + 1 ; randomly chosen bar from the empirical turning angle histogram with a total of 214 turning angles (derived from harmonic radar dataset, Osborne et al 2013)

   if randBar >= 1 and randBar < 24 [ set turn 0 + random-float 10 ]
   if randBar >= 24 and randBar < 36 [ set turn 10 + random-float 10 ]
   if randBar >= 36 and randBar < 45 [ set turn 20 + random-float 10 ]
```

```
  if randBar >= 45 and randBar < 58 [ set turn 30 + random-float 10 ]
  if randBar >= 58 and randBar < 73 [ set turn 40 + random-float 10  ]
  if randBar >= 73 and randBar < 81 [ set turn 50 + random-float 10 ]
  if randBar >= 81 and randBar < 86 [ set turn 60 + random-float 10 ]
  if randBar >= 86 and randBar < 97 [ set turn 70 + random-float 10 ]
  if randBar >= 97 and randBar < 107 [ set turn 80 + random-float 10 ]
  if randBar >= 107 and randBar < 112 [ set turn 90 + random-float 10 ]
  if randBar >= 112 and randBar < 124 [ set turn 100 + random-float 10 ]
  if randBar >= 124 and randBar < 133 [ set turn 110 + random-float 10 ]
  if randBar >= 133 and randBar < 143 [ set turn 120 + random-float 10 ]
  if randBar >= 143 and randBar < 154 [ set turn 130 + random-float 10 ]
  if randBar >= 154 and randBar < 166 [ set turn 140 + random-float 10 ]
  if randBar >= 166 and randBar < 178 [ set turn 150 + random-float 10 ]
  if randBar >= 178 and randBar < 192 [ set turn 160 + random-float 10 ]
  if randBar >= 192 and randBar <= 214 [ set turn 170 + random-float 10 ]
 ]

 if random-float 1 > 0.5 [ set turn turn * -1 ]
 if RandomWalk = true [ set turn random-float 360 ] ; uncorrelated random walk
 set turn turn * LinearisationFactor
 if FixTurningAngle = true [ set turn FixRightTurn ]

 report turn
end

;;
=================================================================================================
=====================================================
```

to-report RelocationREP

```
  ; defines new destination of scouts after a scouting round   ; RUN A PROCEDURE
  let result []
```

```
if SearchMode = "colony"   ; as scouts are already at the colony and they immediately switch into search mode..
  [
    set result []    ;  .. hence destinationList remains empty!
  ]

if SearchMode = "visited NLpatch (recruitment)" ; .. or at any location ever visited by at least one bee..
  [
    ifelse count patches with [ visits > 0 and patchColor != LakeColor ] > 0
      [
        ask one-of patches with [ visits > 0 and patchColor != LakeColor ]
          [ set result list pxcor pycor ]
      ]
      [ set result list Col_X Col_Y ]
  ]

if SearchMode = "random location" ; .. or at any location within MaxForagingRange_m
  [
     ask one-of patches with [ patchColor != LakeColor and distancexy Col_X Col_Y * Scaling < MaxForagingRange_m and originalColor != BorderColor  ]
         [ set result list pxcor pycor ]

  ]

if SearchMode = "known flowerpatch (individual)"  ; ..or at a flower patch, already known to that particular scout..
 [
    let memoKnownPatchesList []
    ifelse empty? knownPatchesList
      [ set result [] ]
      [
        set memoKnownPatchesList knownPatchesList
        let theChosenPatch one-of memoKnownPatchesList   ; bigger patches are more likely to be chosen
        ask one-of patches with [ flowerpatchid = theChosenPatch ]  ; (do NOT replace theChosenPatch by "one-of memoKnownPatchesList"!)
          [ set result list pxcor pycor ]
      ]
```

```
  ]

if SearchMode = "known flowerpatch (recruitment)"   ; .. or at any one of all flower patches known to the whole colony..
 [
   let memoKnownPatchesList []
   ask patchstatistics
    [
     if totalDetections > 0  [ set memoKnownPatchesList fput who memoKnownPatchesList ]
    ]
   ifelse empty? memoKnownPatchesList
     [ set result [] ]
     [
       let theChosenPatch one-of memoKnownPatchesList             ; bigger patches are more likely to be chosen
       ask one-of patches with [ flowerpatchid = theChosenPatch ]  ; (do NOT replace theChosenPatch by "one-of memoKnownPatchesList"!)
         [ set result list pxcor pycor ]
     ]
  ]

if SearchMode = "furthest location (individual)"  ; .. or at the furthest location this individual scout has ever been
  [ set result furthestLocationList ]

if SearchMode = "last location (individual)"  ; .. or at the last location this individual scout has been before returning to the hive
  [ set result lastLocationList ]

ifelse empty? result   ; new colour, depending if bees are in search mode or have a destination
 [
  set color white
  set flightPhase 2
 ]
 [
  set color green
  set flightPhase 1
 ]
```

```
  report result
end

;; ========================================================================================================================================================

```

to MoveBeesProc

```
  let returningProb Gap_s / TripDuration_s ; if trip duration is randomly determined, this results in an average trip duration of TripDuration_s (only if ImmediateReturn = false)
  set TotalTrips 0
  ask bees
   [
     set time_s time_s + Gap_s               ; 3s between each recorded position in experimental radar data
     if SearchMode = "mixed strategy (individual)"
       [
         set MixedStratInd true
         set SearchMode one-of [ "colony" "known flowerpatch (individual)" "furthest location (individual)" "last location (individual)" ]
       ]

     if SearchMode = "mixed strategy (recruitment)"
       [
         set MixedStratCol true
         set SearchMode one-of [ "colony" "visited NLpatch (recruitment)" "known flowerpatch (recruitment)" "furthest location (individual)" "last location (individual)" ]
       ]

     if flightPhase = 2 [ set lastLocationList list xcor ycor ]     ; saves current location (only if bees are in search mode)

     ; if this is the END OF AN INDIVIDUAL'S SCOUTING ROUND:
     if (RandomTripDuration = false and remainder (ticks * Gap_s) (Gap_s * round (TripDuration_s / Gap_s)) = 0)   ; rounding: to make sure that trip duration can be divided by Gap_s
```

```
    or (RandomTripDuration = true and random-float 1 <= returningProb)
   [
    set destinationList list Col_x Col_y  ; then the new destination of a bee is the colony
    set color sky
    set flightPhase 3
   ]

  ; determine direction:
  ifelse destinationList = []            ; movement depends if or if not a bee has a destination
    [ TurnNoDestinationProc ]    ; CALL A PROCEDURE
    [ TurnDestinationProc ]     ; (remember: lakes do not affect bee movement, if it has a destination!)  ; CALL A PROCEDURE

  if (distancexy Col_x Col_y) * Scaling > MaxHiveDisplAllBees_m
    [ set MaxHiveDisplAllBees_m Scaling * distancexy Col_x Col_y ]  ; MaxHiveDisplAllBees_m: furthest distance of any individual from the colony ever

  if (distancexy Col_x Col_y) > DisplacementREP ; time_s    ; "visits": patches-own variable to show the distribution of bees in the landscape
    [ set visits visits + 1 ]             ; not recorded directly at the hive (i.e. within 1 Netlogo-Patch), as this would outshine visits elsewhere

  DetectionProc   ; to determine if a patch is detected   ; CALL A PROCEDURE

  if MixedStratInd = true
   [ set SearchMode "mixed strategy (individual)" ]
  if MixedStratCol = true
   [ set SearchMode "mixed strategy (recruitment)" ]

  ifelse nTrips <= MaxTrips
   [ set TotalTrips TotalTrips + nTrips ]
   [ set TotalTrips TotalTrips + (nTrips - 1) ] ; scouts are not allowed to perform more than MaxTrips trips!
 ]  ; end ask bees


; calculate DETECTION PROBABILITIES and save max. detect. prob. of a patch:
 ask patchStatistics
```

```
    [
      ifelse totalTrips > 0
        [
          set modelledDetectionProb_per_trip totalDetections / totalTrips
          if modelledDetectionProb_per_trip > 1 [ show "Warning! Detection Probability > 1!" ]
        ]
        [ set modelledDetectionProb_per_trip 0 ]

      set modelledDetectionProb_per_s totalDetections / (ticks * Gap_s * N_Bees)


      if modelledDetectionProb_per_s > maxDetectProb_per_s and ticks > TripDuration_s    ;  ticks > TripDuration_s: to avoid random peaks at beginning of
simulation
         [
           set maxDetectProb_per_s modelledDetectionProb_per_s
           set timeMaxDetectProb_s ticks * Gap_s
         ]
    ]
   if remainder ticks 10 = 0 [ if any? patches with [ patchColor = -1 ] [ show "BUGALARM!" ask patches [ set pcolor pink ]]]

   ; actual MOVEMENT:
   ask bees with [ nTrips <= MaxTrips ] ;          when bees have finished the max. number of trips, they don't move anymore (i.e. they stay in the hive)
    [
      fd DisplacementREP ; time_s
      if (patchColor = LakeColor)                        ; LAKES & BORDERS:
        or (originalColor = BorderColor)                     ;       if a scout reaches a lake - or the borders of the world..
        or (pxcor = max-pxcor) or (pxcor = min-pxcor)
        or (pycor = max-pycor) or (pycor = min-pycor)
         [
           if flightPhase = 2 and destinationList = [] ; i.e. lakes only affect "white" bees in searching phase, not "orange" bees doing a loop
            [
              setxy item 0 lastLocationList item 1 lastLocationList   ;       .. it jumps back to its earlier position..
              set heading random-float 360                          ;       .. and chooses a new dircetion (only in searching phase!)
```

```
          ]
        ]
    set distanceFromColony_m (distancexy Col_X Col_Y) * Scaling
    ]

end


;; ==============================================================================================================================================================================
```

to TurnDestinationProc

```
; (CALLED BY INDIVIDUAL BEE) only called, if the bee has a destination   ; RUN A PROCEDURE

 facexy item 0 destinationList item 1 destinationList ; bee turns towards its destination
 if (distancexy Col_x Col_y < DisplacementREP) and (flightPhase = 2) ; if a (orange) bee loops around the colony: it retruns to ordinary search mode (white) once it reaches the colony
      [
        set destinationList []
        set color white
       ]
 if (distancexy Col_x Col_y < DisplacementREP) and (flightPhase = 3) ; if (blue) bee is back (i.e. within range of 1 step) at the colony, and flightPhase is 3 (i.e. blue, returning) (to avoid multiple relocation) their new destination is determined
      [
         let newXYlist RelocationREP
         if SearchMode = "last location (individual)" or MixedStratInd = true  or MixedStratCol = true
            [ set lastLocationList list xcor ycor ]
         set destinationList newXYlist
         set nowDetectedPatchesList [] ; a new scouting trip starts, hence list is empty
         if nTrips <= MaxTrips [ set nTrips nTrips + 1 ]
       ]
```

```
 if destinationList != []  ; only relevant, if bee has a destination (might not be the case if bees are about to leave the colony again, e.g. if SearchMode = "colony")
   [
     if (distancexy item 0 destinationList item 1 destinationList) < DisplacementREP and destinationList != list Col_x Col_y ; if this bee is at its field destination (within range of 1 step) (i.e. not at the colony)
       [
         set lastDestinationList destinationList
         set destinationList []              ; no more destination, i.e. bees switches into search behaviour
         set heading random-float 360
         set color white
         set flightPhase 2
       ]
   ]

end

;;
======================================================================================================================================================
==========================================================
```

to TurnNoDestinationProc

```
  ; (CALLED BY INDIVIDUAL BEE)   ; RUN A PROCEDURE
        rt TurningREP                              ; MOVEMENT if bee is in searching phase (i.e. without a destination):
        if random-float 1 <= TurnToDestinationProb
           [
            set destinationList lastDestinationList
            set color orange
           ]

        if SearchMode = "furthest location (individual)" or MixedStratInd = true or MixedStratCol = true
```

```
        [
         if (distancexy Col_X Col_Y) * Scaling > furthestDistance_m
         [
           set furthestLocationList list xcor ycor
           set furthestDistance_m (distancexy Col_X Col_Y) * Scaling
         ]
        ]

end

;;
====================================================================================================================================================================
```

to DetectionProc

```
; (CALLED BY INDIVIDUAL BEE) determines if a patch is detected   ; RUN A PROCEDURE
   ; possible DETECTION of a flower patch, if bee enters a red, blue, yellow or green NL-patch:

 if nTrips <= MaxTrips   ; patches can only dected, while MaxTrips is not exceeded!
  [
   if member? patchColor PatchColoursList  ; if a bee arrives at a patch

     [
      let patchNumber flowerPatchID
      if member? patchNumber nowDetectedPatchesList = false
      [
        set nowDetectedPatchesList fput patchNumber nowDetectedPatchesList
        ask PatchStatistic patchNumber
          [
           set totalDetections totalDetections + 1
           if firstDetection_s = 0
             [ set firstDetection_s ticks * Gap_s ]; time [s] when patch was detected for the first time
```

```
          ]
        ] ;; patch is added to the nowDetectedPatchesList if it wasn't detected earlier during this trip
        if member? patchNumber knownPatchesList = false
        [
         set knownPatchesList fput patchNumber knownPatchesList   ;; patch is added to the knownPatchesList if it wasn't detected ever
         if ImmediateReturn = true [
                                set destinationList list Col_x Col_y
                                set color sky
                                set flightPhase 3
                              ]
        ]
      ]
  ]
end

;;
==============================================================================================================
==========================================
```

to WriteFromListToFileProc

```
 ;; called by: AnalyseProc   ; RUN A PROCEDURE
 let dayCounter 1
 let patchCounter 0
 file-print "day id oldPatchID patchType distance_m xcor ycor size_sqm quantityPollen_g concentration quantityNectar_l calculatedDetectionProb_per_trip
modelledDetectionProb_per_trip nectarGathering_s pollenGathering_s "
 foreach sort patchStatistics
 [
   ask ?
   [
     if closestDistance_m <= MaxForagingRange_m
     [
       repeat 365
```

```
      [
        file-type dayCounter file-type " "                                    ;;   1.) day
        file-type patchCounter file-type " "                                  ;;   2.) ID (= who, if MaxForagingRange > closestDistance_m of furthest patch)
        file-type who file-type " "                                           ;;   3.) who = "old ID"
        file-type patchType file-type " "                                     ;;   4.) patchType
        file-type closestDistance_m file-type " "                             ;;   5.) closestDistanceM
        file-type round ((xcor - Col_X) * (ScaleDistance_m / (Scale_X2 - Scale_X1)))   file-type " "       ;;   6.) xcor relative to hive
        file-type round ((ycor - Col_Y) * (ScaleDistance_m / (Scale_X2 - Scale_X1)))  file-type " "        ;;   7.) ycor relative to hive
        file-type areaSqm file-type " "                                       ;;   8.) areaSqm
        ifelse dayCounter >= startDay and dayCounter <= stopDay               ;;   9.) quantityPollen_g
          [ file-type quantityPollen_g file-type " " ]
          [ file-type 0 file-type " " ]                                       ;;       ( no pollen, if patch is not in flower)
        file-type concentration file-type " "                                 ;;   10.) concentration
        ifelse dayCounter >= startDay and dayCounter <= stopDay               ;;   11.) quantityNectar_l
          [ file-type quantityNectar_l file-type " " ]
          [ file-type 0 file-type " " ]                                       ;;       ( no nectar, if patch is not in flower)
        file-type calculatedDetectionProb_per_trip file-type " "              ;;   12.) calculatedDetectionProb_per_trip
        file-type modelledDetectionProb_per_trip file-type " "                ;;   13.) modelledDetectionProb_per_trip
        file-type nectarGathering_s file-type " "                             ;;   14.) nectarGathering_s
        file-type pollenGathering_s file-type " "                             ;;   15.) pollenGathering_s "
        file-print (" ")
        set dayCounter dayCounter + 1
        if dayCounter = 366 [ set dayCounter 1 ]
      ]
      set patchCounter patchCounter + 1
    ]
   ]
  ]
end

;;
==================================================================================================
======================================
```

to DoDetectionPlotsProc

```
 ; RUN A PROCEDURE
 let detectProb1 [ modelledDetectionProb_per_trip ] of PatchStatistic Plot1
 let detectProb2 [ modelledDetectionProb_per_trip ] of PatchStatistic Plot2
 let detectProb3 [ modelledDetectionProb_per_trip ] of PatchStatistic Plot3
 let detectProb4 [ modelledDetectionProb_per_trip ] of PatchStatistic Plot4

  set-current-plot "Detection Probability per trip"
    set-current-plot-pen "plot1"
    plot (detectProb1)
    set-current-plot-pen "plot2"
    plot (detectProb2)
    set-current-plot-pen "plot3"
    plot (detectProb3)
    set-current-plot-pen "plot4"
    plot (detectProb4)
end


;;
==========================================================================================================
========================================
```

to ReadForagingDataProc

```
 ; RUN A PROCEDURE
   set ForagingFileList []
   file-open InputForagingFile
   let nPatchesInFile file-read
```

```
  let dustbin file-read-line  ;; to skip the headings of the txt file
  while [ not file-at-end? ] [ set ForagingFileList sentence ForagingFileList (list (list file-read file-read file-read file-read))]
  set ForagingFileList fput nPatchesInFile ForagingFileList
  file-close
  set NewTimestepForaging 1
end

;;
=========================================================================================================================================================================
```

to ShowForagingDataProc

```
 let ForagingFileListToday []
 let nPatchesInFile item 0 ForagingFileList   ; first item (i.e. number 0) in list: # of patches, second item: first day, first patch data
 let itemNumber 1 + nPatchesInFile * (NewTimestepForaging - 1)   ; this is the first item with foraging data for today
 let itemCounter 0
 let columns length item 1 ForagingFileList ; number of data columns in the foraging input file

 repeat nPatchesInFile
  [
   set ForagingFileListToday sentence ForagingFileListToday (item (itemNumber + itemCounter) ForagingFileList)
   set itemCounter itemCounter + 1
  ]

 if item 0 ForagingFileListToday != NewTimestepForaging
   [ user-message "Error in ShowForagingDataProc - wrong day!" ]

 let currentItem 1  ; i.e. the first "who" in the list (item 0 is the day)
 let nextWho item currentItem ForagingFileListToday
 let maxItems length ForagingFileListToday
```

```
foreach sort patchStatistics
[
 ask ?
 [
   ifelse who = nextWho and currentItem + columns - 1 <= maxItems  ; if who is on the list and its not the end of the list yet, read data from list

   [
     set nNectarVisits item (currentItem + 1) ForagingFileListToday
     set nPollenVisits item (currentItem + 2) ForagingFileListToday
     set currentItem currentItem + columns  ; the position of the next "who" in the list
     if currentItem <= maxItems [ set nextWho item currentItem ForagingFileListToday ]

   ]
   [    ; ELSE: if patch is not on the list, it can't have had any visitors!
     set nNectarVisits 0
     set nPollenVisits 0
   ]

 ]
]

ask PatchStatistics
[
  if ForagingMap = "Nectar" [ set visitColor scale-color yellow nNectarVisits 0 MaxVisitsColour ]
  if ForagingMap = "Pollen" [ set visitColor scale-color orange nPollenVisits 0 MaxVisitsColour ]
  if ForagingMap = "All visits" [ set visitColor scale-color red (nNectarVisits + nPollenVisits) 0 MaxVisitsColour ]
  let memoWho who
  ask patches with [ flowerPatchID = memoWho ] [ set pcolor [ visitColor ] of patchStatistic memoWho ]
  if nNectarVisits + nPollenVisits > 0 and closestDistance_m > MaxForagingRange_m
    [ show "Warning! Foraging outside the max. foraging range!" ]
]
```

```
end

;;
=================================================================================================
=====================================
```

to-report DateREP

```
 let month-names (list "January" "February" "March" "April" "May" "June" "July" "August" "September" "October" "November" "December")
 let days-in-months (list 31 28 31 30 31 30 31 31 30 31 30 31)


 let year floor (NewTimeStepForaging / 365.01) + 1
 let month 0
 let dayOfYear remainder NewTimeStepForaging 365
 if dayOfYear = 0 [ set dayOfYear 365 ]
 let dayOfMonth 0
 let sumDaysInMonths 0
 while [ sumDaysInMonths < dayOfYear ]
 [
  set month month + 1
  set sumDaysInMonths sumDaysInMonths + item (month - 1) days-in-months
  set dayOfMonth dayOfYear - sumDaysInMonths + item (month - 1) days-in-months
 ]

 report (word dayOfMonth "  " (item (month - 1) month-names) ) ; " " year )

end

;;
=================================================================================================
```

```
==============================================================================================================================================================================================================================
====

to BumbleBeehaveSetFlowerSpeciesProc
; updates flowerspecies in each patchtype

ask patchStatistics
[
  if patchColor = Red [ set flowerSpeciesList FlowerSpeciesList_R ]
  if patchColor = Yellow [ set flowerSpeciesList FlowerSpeciesList_Y ]
  if patchColor = Green  [ set flowerSpeciesList FlowerSpeciesList_G ]
  if patchColor = Blue  [ set flowerSpeciesList FlowerSpeciesList_B ]
  if patchColor = Brown [ set flowerSpeciesList FlowerSpeciesList_BR ]
  if patchColor = Turquoise  [ set flowerSpeciesList FlowerSpeciesList_T ]
  if patchColor = Violet [ set flowerSpeciesList FlowerSpeciesList_V ]
  if patchColor = Magenta [ set flowerSpeciesList FlowerSpeciesList_M ]
  if patchColor = Pink [ set flowerSpeciesList FlowerSpeciesList_P ]
]

end

;;
==============================================================================================================================================================================

to BumbleBeehaveOutProc
 BumbleBeehaveSetFlowerSpeciesProc
 ask (turtle-set Patchstatistics bees hives) [ hide-turtle]
```

```
 export-view  BumbleBeehaveImage  Filename
 if file-exists? BumbleBeehave  Inputfile [ file-delete BumbleBeehave  Inputfile ]
 file-open BumbleBeehave  Inputfile
 file-print Scaling
 file-print count patchStatistics
 file-print "id patchType patchColour xcor ycor size_sqm quantityPollen_g proteinPollenProp quantityNectar_l concentration_mol/l startDay stopDay
corollaDepth_mm nectarFlowerVolume_myl intFlowerTime_s flowerSpeciesList info"

 foreach sort patchStatistics
 [
   ask ?
   [
     file-type who file-type " "
     file-type patchType file-type " "
     file-type color + 1 file-type " "
     file-type precision xcor 3 file-type " "
     file-type precision ycor 3 file-type " "
     file-type precision areaSqm 1 file-type " "
     file-type precision quantityPollen_g 0 file-type " "
     file-type precision proteinPollenProp 3 file-type " "
     file-type precision quantityNectar_l 3 file-type " "
     file-type concentration file-type " "
     file-type startDay file-type " "
     file-type stopDay file-type " "
     file-type corollaDepth_mm file-type " "
     file-type nectarFlowerVolume_myl file-type " "
     file-type interFlowerTime_s  file-type " "
     file-type flowerSpeciesList  file-type " "

     file-write patchInfo ; printed as string i.e. with " "
     file-print (" ")
   ]
 ]
```

```
 file-close
end


;;
==========================================================================================================================
===============================================================================

;---------------  BUTTONS  ---------------  BUTTONS  ---------------  BUTTONS  ---------------  BUTTONS  ---------------  BUTTONS  ---------------  BUTTONS  ---------------  BUTTONS  ---------------

;;
==========================================================================================================================
===============================================================================
```

to Default_ButtonProc

; ~~RUN A PROCEDURE~~"Default (Honeybees") button

;; NOT set by Default button:
 ;set RandSeed 1
 ;set Plot1 1
 ;set Plot2 2
 ;set Plot3 3
 ;set Plot4 4

;; SET by "Default (Honeybees") button:
 set BeeSpecies "Honeybees"
 set Black_th 1
 set Blue_max 110
 set Blue_min 90
 set BluePatches TRUE ;White Clover

```
set TurquoisePatches FALSE ;Grassland Pasture
set Turquoise_min 70
set Turquoise_max 80
set VioletPatches FALSE ;Gardens
set Violet_min 110
set Violet_max 120
set BrownPatches FALSE ;Woodland
set Brown_min 30
set Brown_max 40
set MagentaPatches FALSE ;Semi-natural Scrub
set Magenta_min 120
set Magenta_max 130
set PinkPatches FALSE ;Semi-natural Grassland
set Pink_min 130
set Pink_max 140
set BrushSize 3
set ByColour 0 ; 0: black
set Col_X max-pxcor / 2 ; 160
set Col_Y max-pycor / 2 ; 106
set Conc_B 1.5
set Conc_G 1.5
set Conc_R 1.5
set Conc_Y 1.5
set Conc_T 1.5 ; Turquoise- Grassland Pasture
set Conc_V 1.5 ; Violet- Gardens
set Conc_BR 1.5 ; Brown- Woodland
set Conc_M 1.5 ; Magenta- Semi-natural Scrub
set Conc_P 1.5 ; Pink- Semi-natural Grassland
set Day_x 1
set DisplacementFactor 1
set FixRightTurn 0.2
set FixTurningAngle FALSE
set ForagingMap "Nectar"
```

```
set  Green_max  70
set  Green_min  50
set  GreenPatches TRUE ; false
set  Gridsize 1000
set  Highlight_Patch  0
set  ImmediateReturn  FALSE
set  InputFile "No input file"
set  InputForagingFile  "Input_1-2_Foraging.txt"
set  Lakes  FALSE
set  LinearisationFactor  1
set  MaxForagingRange_m  10000
set  MaxPatchRadius_m  500
set  MaxTrips 999999
set  MaxVisitsColour  1000
set  N_Bees  10000
set  NameOutfile  "Input_2-1_FoodFlow.txt"
set  Nectar_B  1
set  Nectar_G  1
set  Nectar_R  1
set  Nectar_Y  1
set  Nectar_T  1 ; Turquoise- Grassland Pasture
set  Nectar_V  1 ; Violet- Gardens
set  Nectar_BR  1 ; Brown- Woodland
set  Nectar_M  1 ; Magenta- Semi-natural Scrub
set  Nectar_P  1 ; Pink Semi-natural Grassland
set  Patchtype_B  "\"BlueField\""
set  Patchtype_G  "\"GreenField\""
set  Patchtype_R  "\"RedField\""
set  Patchtype_Y  "\"YellowField\""
set  Patchtype_T  "\"TurquoiseField\"" ; Grassland Pasture
set  Patchtype_V  "\"VioletField\"" ; Gardens
set  Patchtype_BR  "\"BrownField\"" ; Woodland
set  Patchtype_M  "\"MagentaField\"" ;Semi-natural Scrub
```

```
set  Patchtype_P  "\"PinkField\"" ; Semi-natural Grassland
set  Pollen_B  1
set  Pollen_G  1
set  Pollen_R  1
set  Pollen_Y  1
set  Pollen_T  1 ; Turquoise- Grassland Pasture
set  Pollen_V  1 ; Violet- Gardens
set  Pollen_BR  1 ; Brown- Woodlands
set  Pollen_M  1 ; Magenta- Semi-natural Scrub
set  Pollen_P  1 ; Pink Semi-natural Grasslands
set  RandomTripDuration  TRUE
set  RandomWalk  FALSE
set  Red_max  30
set  Red_min  10
set  RedPatches  TRUE
set  ReplaceColour  85   ; 85: cyan ("lake" colour)
set  SatelliteFile  "No satellite image"
set  Scale_X1  1
set  Scale_X2  max-pxcor
set  ScaleDistance_m  3000 ; average foraging distance (i.e. radius) ca. 1500m; focus on efficient patches
set  ScoutingPeriod_hrs  9
set  SearchMode  "known flowerpatch (recruitment)"
set  SetColour  "Yellow"
set  SetDirection_deg  90
set  SetDistanceToCentre_m  250
set  SetRadius_m  50
set  Start_B  271
set  Start_G  181
set  Start_R  1
set  Start_Y  91
set  Start_T  0 ;Turquoise- Grassland Pasture
set  Start_V  0 ;Violet- Gardens
set  Start_BR 0 ;Brown- Woodlands
```

```
set Start_M 0 ;Magenta- Semi-natural Scrub
set Start_P 0 ;Pink-Semi-natural Grasslands
set Stop_B 360
set Stop_G 270
set Stop_R 90
set Stop_Y 180
set Stop_T 0 ;Turquoise- Grassland Pasture
set Stop_V 0 ;Violet- Gardens
set Stop_BR 0 ;Brown- Woodlands
set Stop_M 0 ;Magenta- Semi-natural Scrub
set Stop_P 0 ;Pink-Semi-natural Grasslands
set t_Nectar_B 1200
set t_Nectar_G 1200
set t_Nectar_R 1200
set t_Nectar_Y 1200
set t_Nectar_T 1200 ; Turquoise- Grassland Pasture
set t_Nectar_V 1200 ; Violet- Gradens
set t_Nectar_BR 1200 ;Brown- Woodland
set t_Nectar_M 1200 ; Magenta- Semi-natural Scrub
set t_Nectar_P 1200 ; Pink- Semi-natural Grassland
set t_Pollen_B 600
set t_Pollen_G 600
set t_Pollen_R 600
set t_Pollen_Y 600
set t_Pollen_T 600 ;Turquoise- Grassland Pasture
set t_Pollen_V 600 ; Violet-Gardens
set t_Pollen_BR 600 ; Brown- Woodland
set t_Pollen_M 600 ; Magenta- semi-natural Scrub
set t_Pollen_P 600 ; Pinl- Semi-natural Grassland
set TripDuration_s 1020
set TurnToDestinationProb 0.02
set White_th 9
set Yellow_max 50
```

set Yellow_min 40
set YellowPatches TRUE

; new for BEESCOUT 2.0:

set AutoName TRUE
set BumbleBeehave_Inputfile "No input_ Patches.txt"
set BumbleBeehaveImage_Filename "No input_ View.png"
set Corolla_B 5
set Corolla_BR 5
set Corolla_G 5
set Corolla_M 5
set Corolla_P 5
set Corolla_R 5
set Corolla_T 5
set Corolla_V 5
set Corolla_Y 5
set FlowerVol_B 4
set FlowerVol_BR 4
set FlowerVol_G 4
set FlowerVol_M 4
set FlowerVol_P 4
set FlowerVol_R 4
set FlowerVol_T 4
set FlowerVol_V 4
set FlowerVol_Y 4
set HabitatsInput "BS-Habitats_Herts.csv"
set HighResolution FALSE
set IntFlowerT_B 1.54 ; Pyke (1978): inter flower time: 1.54s, (Oecologia 34, 255-266)
set IntFlowerT_BR 1.54 ; Alternative values: 2.5s (Pyke 1978, Fig. 2, taking time within and between inflorescences into account, i.e. calculated as: ((1.6 * flowers per inflorescence) + ca. 3)/ flowers per inflorescence
set IntFlowerT_G 1.54 ; or 0.6s (Balfour et al 2013, Tab. 1)

```
 set  IntFlowerT_M 1.54
 set  IntFlowerT_P 1.54
 set  IntFlowerT_R 1.54
 set  IntFlowerT_T 1.54
 set  IntFlowerT_V 1.54
 set  IntFlowerT_Y 1.54
 set  Protein_B 0.2        ; Hrassnig & Crailsheim 2005: assume 20% protein in pollen, (Apidologie 36, 255–277)
 set  Protein_BR 0.2
 set  Protein_G 0.2
 set  Protein_M 0.2
 set  Protein_P 0.2
 set  Protein_R 0.2
 set  Protein_T 0.2
 set  Protein_V 0.2
 set  Protein_Y 0.2
 set  TextMap "SI_07_BS-Map-T_Suss1.txt"


end

;; ================================================================================================================================================
```

to ClearBeesProc

```
 if RandSeed > 0 [ random-seed RandSeed ]
 CreateBeesProc
 set MaxHiveDisplAllBees_m 0
 set TotalTrips 0
 set SCALING ScaleDistance_m / (Scale_X2 - Scale_X1)
 clear-drawing
 clear-all-plots
```

```
 clear-ticks
 RESET-TICKS
end

;;
==============================================================================================================================
=================================================
```

to BumblebeesProc

```
 set BeeSpecies "Bumblebees"
 set SearchMode "known flowerpatch (individual)"
 set N_Bees 30
 set TripDuration_s 600
 set ScoutingPeriod_hrs 9
 set LinearisationFactor 1
 set DisplacementFactor 1
 set RandomWalk false
 set RandomTripDuration true
 set ImmediateReturn false
 set MaxTrips 999999
 set TurnToDestinationProb 0.02
end

;;
==============================================================================================================================
=================================================
```

to HoneybeesProc

```
 set BeeSpecies "Honeybees"
 set SearchMode "known flowerpatch (recruitment)"
 set N_Bees 10000
```

```
 set TripDuration_s 1020 ; as in BEEHAVE model
 set ScoutingPeriod_hrs 9
 set LinearisationFactor 1
 set DisplacementFactor 1
 set RandomWalk false
 set RandomTripDuration true
 set ImmediateReturn false
 set MaxTrips 999999
 set TurnToDestinationProb 0.02
end

;;
====================================================================================================================================================================
;; ---------------         END         ;;
====================================================================================================================================================================
;; ---------------         END         ---------------         END         ---------------         END         ---------------         END         ---------------         END         ---------------         END         ---------------
;;
====================================================================================================================================================================
```
