# Supplementary figures and images for: Bumble‐BEEHAVE: A systems model for exploring multifactorial causes of bumblebee decline at individual, colony, population and community level
Source: J Appl Ecol. 2018 May 22;55(6):2790–801. doi: 10.1111/1365-2664.13165 (PMC6221040; doi:10.1111/1365-2664.13165)

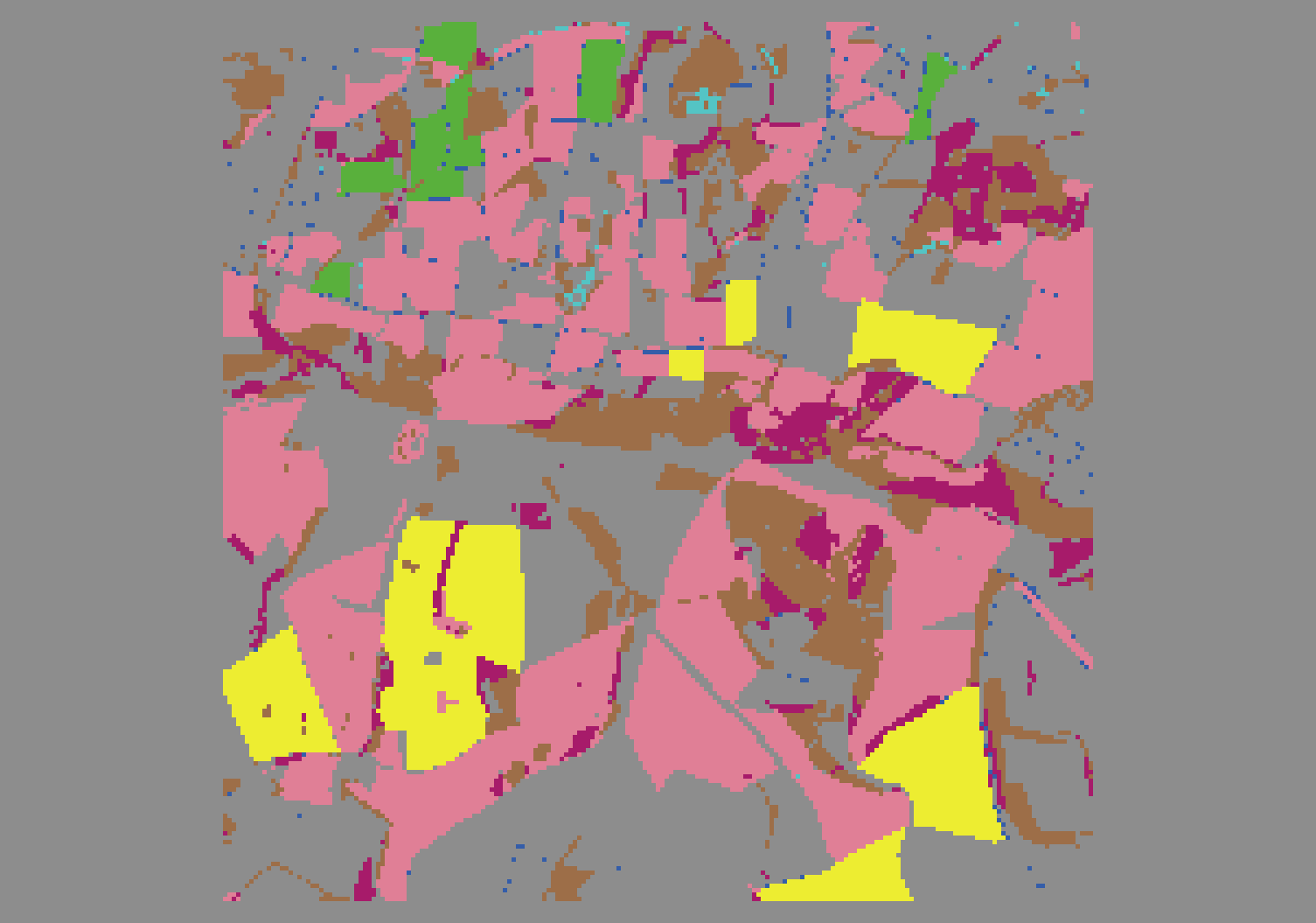

Supplement: Supplementary file 11 [file JPE-55-2790-s011.png]
