# Supplementary material for: Bumble‐BEEHAVE: A systems model for exploring multifactorial causes of bumblebee decline at individual, colony, population and community level
Source: J Appl Ecol. 2018 May 22;55(6):2790–801. doi: 10.1111/1365-2664.13165 (PMC6221040; doi:10.1111/1365-2664.13165)
Supplement: Supplementary file 15 [file JPE-55-2790-s015.pdf]

SI_07_Methodology for calculating BEESCOUT 2.0 Habitats input file and Bumble-BEEHAVE

Flower species input file

In *Bumble*-BEEHAVE, different types of forage patches with multiple flower species differing in nectar and pollen quality, quantity and availability can be simulated. In order to do this, BEESCOUT 2.0 imports a habitats input file, detailing the abundance of each forage flower species in each habitat type. *Bumble*-BEEHAVE imports a flower species input file detailing the nectar quality, quantity and availability for each forage flower species. The methodology of how these data were collected and calculated is detailed below.

BEESCOUT 2.0 Habitats input file

BEESCOUT 2.0 (SI_05, SI_06) enables multiple forage flower species to be modelled in the same forage patch. The new BEESCOUT 2.0 habitats input file (defined by *HabitatsInput*, under the procedure "*DefineHabitatsProc*", e.g. file "BS-Habitats_Suss.csv") represents the abundance as the number of flowers per m^2 of the different forage flower species across the different semi-natural habitat types and crop type patches.

The habitats input file specifies the habitat type and its flower composition for each of the nine possible BEESCOUT colour types (e.g. red (_R), yellow (_Y) and green (_G) etc.). Users can choose to create new crop types using the same methodology as for semi-natural habitat types by adding a new flower species and habitat type to the habitats input file or users can refer to the methodology described previously for BEESCOUT (version 1.0) (Becher *et al.* 2016). To distinguish between crop species in an agricultural field, where the flower density is very high from the sparse occurrence of this crop flower in semi-natural habitats, the word "Crop" was inserted before the crop habitat type and species name in the habitats input file e.g. oilseed rape was named "Crop_Oilseed_rape" in the habitats input file when describing OSR fields but "Oilseed_rape" when it occurred in scrub or hedgerows (see SI_07_BS-Habitats_Suss.csv).

The BEESCOUT 2.0 habitats input file is **SI_07_BS-Habitats_Suss.csv**

The data for the semi-natural habitats in the habitats input file were collected across Sussex in South-East England, UK from transects in the different habitat types of permanent grassland, woodland, scrub and hedgerow. Transects were walked in three replicates of each semi-natural habitat type in five different Sussex landscapes (a total of 60 transects). These were repeated monthly in 2013 from May to August and measured 300m by 4m for scrub, grassland and woodland transects and measured 300m by 2m for hedgerow transects. The number of inflorescences of bumblebee forage plants were counted, along with the numbers of bumblebees and the flowers they were foraging upon. From these data, 34 of the most important flower species for foraging bumblebees were determined in the different habitats types including wild-growing Oilseed rape (*Brassica napus*) and the average of three different species of Willow (*Salix caprea, S. caprea* (hybrid) and *S. cinerea*) which was renamed for the flower species input file as "Average_willow". These 34 flower species are in the *Bumble*-BEEHAVE flower species input file.
The average number of flowers for each forage species was calculated per habitat type and then divided by the total area in m^2 of that transect represents ($1200m^2$ for grassland, woodland and scrub and $600m^2$ for hedgerow) resulting in the average number of flowers per m^2 of that habitat type. This value was then used as the abundance for the corresponding semi-natural habitat type and flower species in the habitats input file.
The abundance of crop flower species (Crop_Oilseed_rape, Crop_Maize and Crop_Field_bean) were taken from the literature as reported in BEESCOUT (Version 1, in S7_Table_CropData_BEESCOUT.pdf , Table A2).

Format:

- The habitats file is displayed as a csv table with the habitat type as rows and the flower species as columns. The first two columns define the colour of the habitat type and the second column defines the classification of the habitat type.
- The first column **Colour(format:FlowerSpeciesList_+abbr.colour)** defines which habitat type's flower species list receives the data of the current row. For each of the nine BEESCOUT colour types a flower species list is defined as a global variable, e.g. "FlowerSpeciesList_R" contains the flower species and their abundances in "red" flower patches, "FlowerSpeciesList_G" in "green" flower

patches etc. The second column **HabitatType** is the category name of the habitat type e.g. “Crop_Field_beans” or "Grassland". For crops the term “Crop_” is used before the crop type name and the same name is used for the crops corresponding “flower” species e.g. Habitat type “Crop_Field_beans”, flower species “Crop_Field_beans”.

– All other columns are the different flower species present in the landscape e.g. **Bugle**. These flower species are named exactly the same as they are in the *Bumble*-BEEHAVE flower species input file.

***Bumble*-BEEHAVE Flower species input file**

For each of the 34 flower species (Table SI_07_1) important for bumblebee forage surveyed in the semi-natural habitat types, up to 30 inflorescences of each flower species were located across the study area and nectar and pollen production rates were quantified following methods in Fowler *et al.* (2016). Nectar production rates were measured by removing existing nectar from flowers at 09:00, bagging flowers using fine-mesh cotton fabric and masking tape, and then quantifying the volume and concentration of nectar produced per unit time. This was repeated three times during the day (15:00, 21:00 and 09:00 the following day– although bees do not forage in the dark, nectar accrued during the night is available the following morning) or in total after 24 hours. Each flower was emptied of nectar using 5ul micropipettes (BLAUBRAND® intraMARK). Micropipettes were prepared by heating the centre of the pipette using a Bunsen burner, and upon melting, pulling apart sharply, and breaking off the melted tip, in order to have a fine end to insert into the corolla. Nectar volume was quantified by measuring the distance along the pipette (the extruded tip was not considered long enough to affect the measurement considerably), and the concentration was measured in the field by expelling the nectar collected per inflorescence onto a refractometer. The average nectar volume (µg) was calculated along with sugar concentration (mol/l) per inflorescence calculated as the average nectar concentration percentage (%) multiplied by the molecular concentration of 1% concentration (10g/l/342.3g/mol) based on 342.3 as the molar mass of sucrose.

Pollen production was quantified by collecting unopened flowers, cutting at the base of the plant, and placing them in water in the lab. Or netting unopened flowers in the field (this was required for

herbaceous species such as *Lotus corniculatus*). Upon opening and ensuring anthers had dehisced, the flower heads were removed to a 5cm petri dish, where the anthers were removed and counted into the dish, before being dried at 40˚C for 24h. Remaining pollen was then brushed off using a small paintbrush. The sample was then weighed, sealed, and stored in -20˚C freezer. This was repeated 10 times per species and the average mg of pollen per flower was calculated. Protein extraction and detection followed Roulston et al. (2000) using the Bradford assay technique (Bradford 1976). From each inflorescence, 1 mg pollen was dusted with aluminium powder, wetted with 20 µl 0.1 mol/L NaOH and ground with a micro-pestle. Ground pollen was reanimated with 480 µl 0.1 mol/L NaOH and placed in a refrigerator for 24 hours before analysis, but used within 1 week. Prior to absorbance measurement samples were placed in hot plate at 95C for 5 minutes and centrifuged for 5 minutes. Then 10 µl of supernatant was slowly vortexed with 300 µl of dye reagent. This was repeated in triplicate for each sample and left to incubate at room temperature for 15 minutes. Protein standards were made up each time samples were run, using pre-mixed concentrations of Bovine Serum Albimum (BSA) from the BIO-RAD Quick-Start™ Bradford Protein Assay kit. Once samples and standards were created, they were measured for absorbance within an hour of mixing at 595 nm using a Thermo Scientific Nanodrop 2000 UV-Vis Spectrophotometer. This produced the standing crop of both mean pollen weight and a crude mean protein concentration for each test species. Then protein (g) was calculated by dividing the total weight produced per inflorescence by 3 (mean number of days for anthers to empty). Finally the proportion of pollen was calculated as the percentage of protein in the pollen divided by 100.

Data on flower phenology and morphology was taken from the literature (Table SI 07), the typical start date of flowering and end date of flowering was recorded along with the depth of the flower corolla tube. Species where the length of corolla tube could not be found were measured in the field by GTD, by selecting 30 individual flowers and measuring from the base of the corolla tube to where the petal separate with digital callipers.

The *Bumble*-BEEHAVE flower species input file is defined by the NetLogo chooser "FlowerspeciesFile" (e.g. **SI_07_BBH-Flowerspecies_Suss.csv**).

Table SI 07. Common name used in the “**Flowerspecies**” column of the flower species input file for *Bumble*-BEEHAVE, scientific name, whether bumblebees use them for nectar (n) or pollen (p) and references for the biological values. Notes: (A) wild growing Oilseed rape, (B) average taken from three *Salix* sp and (C) crops of Oilseed rape, Field beans and maize. References (Refs): Fitter (Fitter & Peat 1994), Brian (Brian 1957), Harder {Harder, 1985 #547}, Conner& Sterling (Conner & Sterling 1995), Clapham {Clapham, 1987 #546}, Peat (Peat *et al.* 2005) , (Sulborska *et al.* 2014), Arbulo (Arbulo *et al.* 2011), Ibanez (Ibanez 2012), data collected by GTD, Brown & Scott (Brown & Scott 1992), Radchenko (Radchenko 1964), Emberlin (Emberlin 1999) and Open (open flower assume corolla depth of 0).

Flowerspecies	Scientific name	n	p	Notes	Refs
"Bugle"	*Ajuga reptans*	✓	✓		Fitter & Peat, Brian
"Burdock"	*Arctium minus*	✓	✓		Fitter & Peat, Harder
"Oilseed_rape"	*Brassica napus*	✓	✓	A	Fitter & Peat, Conner & Sterling
"Giant_bindweed"	*Calystegia sepium*	✓	✓		Fitter & Peat
"Common_knapweed"	*Centaurea nigra*	✓	✓		Fitter & Peat, Brian
"Greater_knapweed"	*Centaurea scabiosa*	×	✓		Fitter & Peat,
"Rosebay_willowherb"	*Chamerion angustifolium*	×	✓		Fitter & Peat,
"Marsh_thistle"	*Cirsium palustre*	✓	✓		Rose, Brian
"Spear_thistle"	*Cirsium vulgare*	✓	✓		Fitter & Peat, Harder
"Hawthorn"	*Crataegus monogyna*	✓	✓		Fitter & Peat,
"Foxglove"	*Digitalis purpurea*	✓	✓		Fitter & Peat, Brian
"Wild_teasel"	*Dipsacus fullonum*	✓	✓		Fitter & Peat, Clapham
"Vipers_bugloss"	*Echium vulgare*	✓	✓		Fitter & Peat, Peat
"Ground_ivy"	*Glechoma hederacea*	✓	✓		Rose, Brian
"Bluebell"	*Hyacinthoides non-scripta*	×	✓		Fitter & Peat
"St_Johns_wort"	*Hypericum perforatum*	×	✓		Fitter & Peat
"Field_scabious"	*Knautia arvensis*	×	✓		Fitter & Peat
"White_dead_nettle"	*Lamium album*	✓	✓		Fitter & Peat, Sulborska
"Red_dead_nettle"	*Lamium purpureum*	✓	✓		Fitter & Peat, Clapham

"Birdsfoot_trefoil"	*Lotus corniculatus*	✓	✓		Fitter & Peat, Arbulo
"Selfheal"	*Prunella vulgaris*	✓	✓		Fitter & Peat, Brian
"Blackthorn"	*Prunus spinose*	✓	✓		Fitter & Peat, Open
"Buttercup"	*Ranunculus sp.*	✓	✓		Fitter & Peat, Open
"Dog_rose"	*Rosa canina*	×	✓		Fitter & Peat, Open
"Bramble"	*Rubus fruticosus*	✓	✓		Rose, Open
"Average_Willow"	*salix spp*	✓	✓	B	Fitter & Peat
"Ragwort"	*Senecio sp.*	×	✓		Fitter & Peat,
"Hedge_woundwort"	*Stachys sylvatica*	✓	✓		Fitter & Peat, Brian
"Comfry"	*Symphytum officinale*	✓	✓		Rose, Brian
"Dandelion"	*Taraxacum officinale*	✓	✓		Rose, Ibanez
"Red_clover"	*Trifolium pratense*	✓	✓		Fitter & Peat, Brian
"White_clover"	*Trifolium reptens*	✓	✓		Fitter & Peat, Brian
"Tufted_vetch"	*Vicia cracca*	✓	✓		Fitter & Peat, Harder
"Common_vetch"	*Vicia sativa*	✓	✓		Fitter & Peat, GTD
"Crop_Field_beans"	*Vicia faba*	✓	✓	C	Brown & Scott, Brian
"Crop_Oilseed_rape"	*Brassica napus*	✓	✓	C	Radchenko, Connor
"Crop_Maize"	*Zea mayes*	×	✓	C	Emberlin

Biological values for the 34 flower species were collected from the literature. This included their phenological start day ("**startDay**") and stop day ("**stopDay"**) for flowering as day of the year and corolla depth in mm ("**corollaDepth_mm"**). Corolla depth is measured as the tube part of the flower that will determine the length of tongue required by a bee to reach the nectar. Reference for this recorded and those species that we only have pollen data for are recorded as having a corolla depth of 0 as corolla depth is irrelevant to pollen collection.

The flower species input file also includes the data for three crop species data taken from the BEESCOUT publication and accompanying references. All the data from the BEESCOUT manuscript on the crop values see Supporting Information of the BEESCOUT manuscript (Becher *et al.* 2016) 2006) Table A2 for full data and references.

Format;

- **Flowerspecies-** The common name of the species in quotes with spaces replaced with "_" and lowercase for the second word in a species name except for willow "Average_Willow".
- **pollen_g/flower-** The quantity of pollen (g) for 1 flower of all the flowers that were surveyed in the semi-natural habitats and crop types.
- **nectar_ml/flower-** As above but for nectar quantity (ml).
- **proteinPollenProp-** The quality of pollen recorded as the proportion of protein in pollen.
- **concentration_mol/l-** The quality of nectar recorded as the concentration of sugar in nectar in mols per litre.
- **startDay-** The availability of nectar and pollen recorded as start day of flowering as day of the year.
- **stopDay-** The last day of flowering as day of the year.
- **corolladepth_mm-** The availability of nectar and pollen recorded as the depth of the Corolla tube (mm) of that species. Note that if a species only provides pollen and not nectar for bumblebees the corolla depth is recorded as 0, this is also true for open flowers such as bramble and close relatives and Oilseed rape.
- **nectarFlowerVolume_myl-** The nectar flower volume recorded as the µl of nectar per flower.
- **intFlowerTime_s-** The amount of time (seconds) for a bumblebee to fly between flowers is recorded as 0.6 seconds for all flower species.

REFERENCES

Arbulo, N., Santos, E., Salvarrey, S. & Invernizzi, C. (2011). Proboscis Length and Resource Utilization in Two Uruguayan Bumblebees: *Bombus atratus* Franklin and Bombus bellicosus Smith (Hymenoptera: Apidae). *Neotrop. Entomol.*, 40, 72-77.

Becher, M.A., Grimm, V., Knapp, J., Horn, J., Twiston-Davies, G. & Osborne, J.L. (2016). BEESCOUT: A model of bee scouting behaviour and a software tool for characterizing nectar/pollen landscapes for BEEHAVE. *Ecological Modelling*, 340, 126-133.

Bradford, M.M. (1976). Rapid and sensitive method for quantification of microgram quantities of protein utilizing principle of protein-dye binding. *Analytical Biochemistry*, 72, 248-254.

Brian, A.D. (1957). Differences in the flowers visited by 4 species of bumble-bees and their causes. *J Anim Ecol*, 26, 71-98.

Brown, B. & Scott, R.R. (1992). An assessment of *Vicia faba* and *Trifolium pratense* as forage crops for *Bombus hortorum*. *New Zealand Entomologist*, 15, 42-47.

Conner, J.K. & Sterling, A. (1995). Testing hypotheses of functional-relationahips - a comparative survey of correlation patterns among floral traits in 5 insect-pollinated plants. *Am. J. Bot.*, 82, 1399-1406.

Emberlin, J. (1999). A report on the dispersal of maize pollen. Research paper commissioned by the Soil Association.

Fitter, A.H. & Peat, H.J. (1994). The Ecoflora database. *J Ecol*, 82, 415-425.

Fowler, R.E., Rotheray, E.L. & Goulson, D. (2016). Floral abundance and resource quality influence pollinator choice. *Insect Conserv Diver*, 9, 481-494.

Ibanez, S. (2012). Optimizing size thresholds in a plant-pollinator interaction web: towards a mechanistic understanding of ecological networks. *Oecologia*, 170, 233-242.

Peat, J., Tucker, J. & Goulson, D. (2005). Does intraspecific size variation in bumblebees allow colonies to efficiently exploit different flowers? *Ecol Entomol*, 30, 176-181.

Radchenko, T.G. (1964). The influence of pollination on the crop and quality of seed of winter rape. *Bdzhil'nitstvo*, 1, 68-74.

Roulston, T.H., Cane, J.H. & Buchmann, S.L. (2000). What governs protein content of pollen: Pollinator preferences, pollen-pistil interactions, or phylogeny? *Ecol Monogr*, 70, 617-643.

Sulborska, A., Dmitruk, M., Konarska, A. & Weryszko-Chmielewska, E. (2014). Adapations of *Lamium album* L. flowers to pollination by Apoidea. *Acta Scientiarum Polonorum-Hortorum Cultus*, 13, 31-43.
