# Supplementary material for: Bumble‐BEEHAVE: A systems model for exploring multifactorial causes of bumblebee decline at individual, colony, population and community level
Source: J Appl Ecol. 2018 May 22;55(6):2790–801. doi: 10.1111/1365-2664.13165 (PMC6221040; doi:10.1111/1365-2664.13165)
Supplement: Supplementary file 16 [file JPE-55-2790-s016.pdf]

SI_08 Sensitivity Analysis

Table SI 08. Sensitivity analysis for biologically relevant parameters defined as numeric, non-integer global variables (either on the interface or the code) with a default value of less or more than zero. For each run we multiplied a parameter's default value by either 0.5, 0.75, 1, 1.25, 1.5 or 2 separately (Sensitivity multiplier) and left all other parameters at default values and recorded the total number of hibernating queens (Hibernating queens) and the total number of males produced (Males produced).

Variable	Sensitivity multiplier					
	0.5	0.75	1	1.25	1.5	2
**Hibernating queens:**						
AbundanceBoost	550.2	654.0	718.2	689.4	642	753.6
DailySwitchProbability	973.8	730.8	590.4	561.6	571.2	525.6
EnergyFactorOnFlower	615.0	614.4	590.4	617.4	701.4	654.0
EnergyRequiredForPollenAssimilation_kJ_per_g	376.2	546.0	590.4	703.8	620.4	521.4
FoodSourceLimit	579.0	626.4	663.6	696.6	741.0	699.6
ForagingMortalityFactor	1365.0	1011.6	576.6	470.4	334.8	126.0
ForagingRangeMax_m	808.8	664.8	590.4	638.4	606.6	684.0
Lambda_detectProb	575.4	628.2	590.4	677.4	721.8	822.0
LarvaWorkerRatioTH	656.4	691.2	590.4	790.2	836.4	828.6
MaxLifespanMales	594.6	558.0	590.4	614.4	616.2	641.4
MetabolicRateFlight_W.kg	688.8	656.4	732.0	658.2	652.2	603.0
MortalityForager_per_s	1804.8	1056.0	590.4	380.4	283.8	98.4
NestSearchTime_h	948.0	744.6	590.4	568.2	516.0	474.6
QueenDestinedEggsBeforeSP_d	210.0	358.2	590.4	741.0	787.2	1063.2
Weather	313.2	547.2	590.4	650.4	586.8	552.6
**Males produced:**						
AbundanceBoost	657.6	810.0	886.2	932.4	856.2	967.8
DailySwitchProbability	565.2	734.4	757.2	888.0	1060.2	1144.2

EnergyFactorOnFlower	893.4	858.0	757.2	868.2	857.4	845.4
EnergyRequiredForPollenAssimilation_kJ_per_g	1348.8	1099.2	757.2	775.2	619.8	482.4
FoodSourceLimit	679.8	812.4	855.0	862.8	1007.4	911.4
ForagingMortalityFactor	1956.0	1246.2	761.4	567.6	439.2	159.6
ForagingRangeMax_m	1126.8	954.6	757.2	817.8	815.4	777.0
Lambda_detectProb	714.6	831.6	757.2	844.2	956.4	1180.2
LarvaWorkerRatioTH	1228.2	897.0	757.2	714	676.8	678.6
MaxLifespanMales	820.2	709.2	757.2	764.4	778.8	815.4
MetabolicRateFlight_W.kg	928.8	862.2	963.6	835.8	792.6	717.6
MortalityForager_per_s	2398.2	1398.0	757.2	511.8	315.6	142.8
NestSearchTime_h	1089.6	982.8	757.2	808.2	657.0	651.0
QueenDestinedEggsBeforeSP_d	1397.4	1279.8	757.2	739.8	615.6	444.6
Weather	565.2	715.2	757.2	1128.0	958.8	1023.6

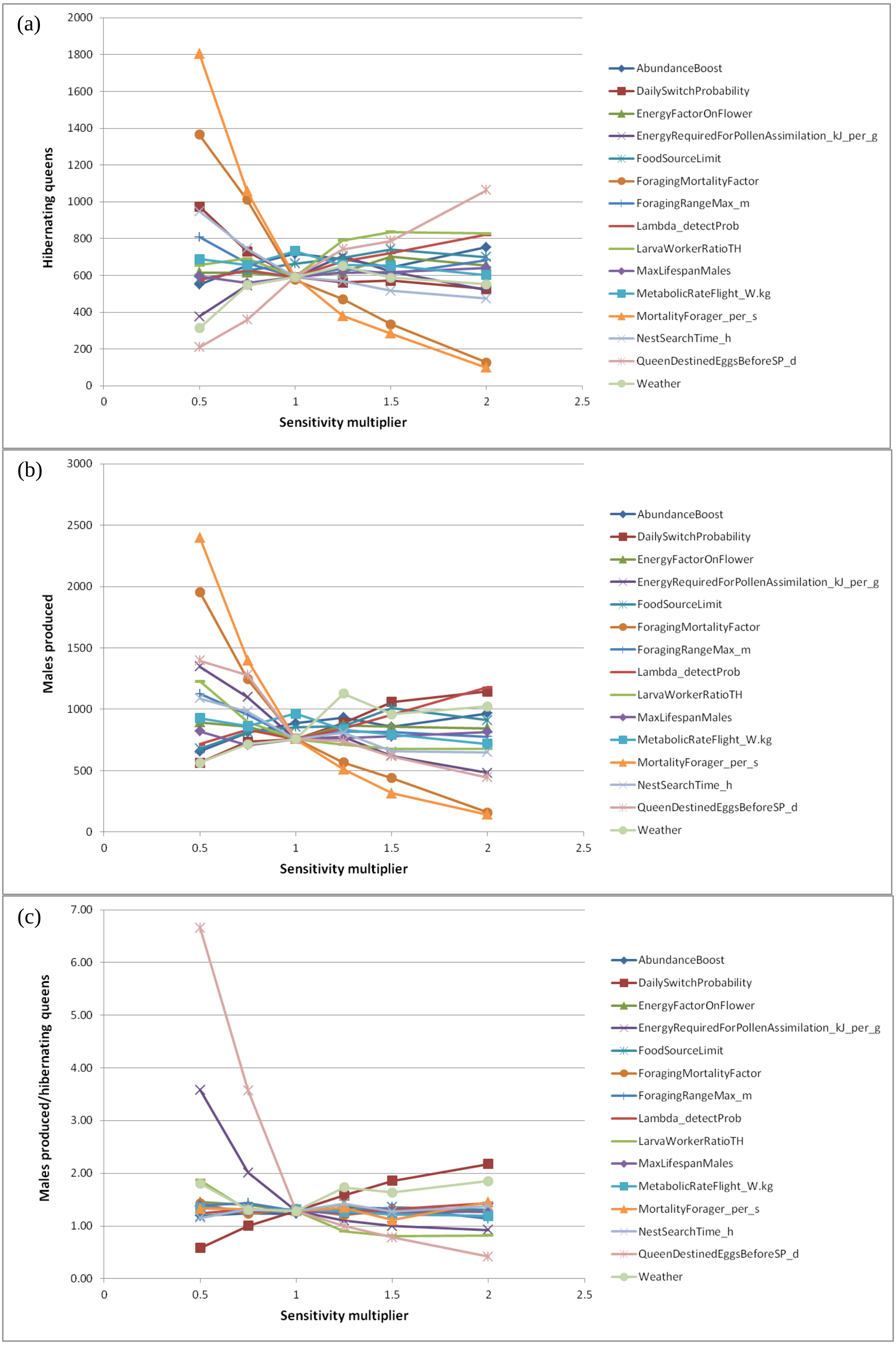
(a)
Hibernating queens
2000
1800
1600
1400
1200
1000
800
600
400
200
0
0
0.5
1
1.5
2
2.5
Sensitivity multiplier
AbundanceBoost
DailySwitchProbability
EnergyFactorOnFlower
EnergyRequiredForPollenAssimilation_kJ_per_g
FoodSourceLimit
ForagingMortalityFactor
ForagingRangeMax_m
Lambda_detectProb
LarvaWorkerRatioTH
MaxLifespanMales
MetabolicRateFlight_W.kg
MortalityForager_per_s
NestSearchTime_h
QueenDestinedEggsBeforeSP_d
Weather
(b)
Males produced
3000
2500
2000
1500
1000
500
0
0
0.5
1
1.5
2
2.5
Sensitivity multiplier
AbundanceBoost
DailySwitchProbability
EnergyFactorOnFlower
EnergyRequiredForPollenAssimilation_kJ_per_g
FoodSourceLimit
ForagingMortalityFactor
ForagingRangeMax_m
Lambda_detectProb
LarvaWorkerRatioTH
MaxLifespanMales
MetabolicRateFlight_W.kg
MortalityForager_per_s
NestSearchTime_h
QueenDestinedEggsBeforeSP_d
Weather
(c)
Males produced/hibernating queens
7.00
6.00
5.00
4.00
3.00
2.00
1.00
0.00
0
0.5
1
1.5
2
2.5
Sensitivity multiplier
AbundanceBoost
DailySwitchProbability
EnergyFactorOnFlower
EnergyRequiredForPollenAssimilation_kJ_per_g
FoodSourceLimit
ForagingMortalityFactor
ForagingRangeMax_m
Lambda_detectProb
LarvaWorkerRatioTH
MaxLifespanMales
MetabolicRateFlight_W.kg
MortalityForager_per_s
NestSearchTime_h
QueenDestinedEggsBeforeSP_d
Weather

Figure SI 08. Graphical representation of the sensitivity analysis on (a) the number of hibernating queens and (b) the number of males produced and (c) on the sex ratio (males produced/hibernating queens) from changes in each of the 15 biologically relevant variables tested.
