# Supplementary material for: Bumble‐BEEHAVE: A systems model for exploring multifactorial causes of bumblebee decline at individual, colony, population and community level
Source: J Appl Ecol. 2018 May 22;55(6):2790–801. doi: 10.1111/1365-2664.13165 (PMC6221040; doi:10.1111/1365-2664.13165)
Supplement: Supplementary file 19 [file JPE-55-2790-s019.pdf]

SI_11_ Investment in queens

Colony Investment in queens

We compared modelled colony investment in queen production from *Bumble*-BEEHAVE to empirical data (Duchateau & Velthuis 1988; Duchateau *et al.* 2004; Gosterit & Gurel 2016). We simulated the colony development in a realistic ("Sussex 1") landscape with one initial *B. terrestris* queen. The colony was implemented in the fully individual based model and ran for 365 days with 7500 replicates (see SI 10, table with setup). We recorded the number of males and queens produced by each colony in each run where workers were produced (Figure SI 12). We then calculated the colony investment in queens for each run where workers were produced using the cost-ratios described in Duchateau *et al*. (2004) assuming a queen is either 2.11 times as costly to produce as a male due to dry weight (Duchateau & Velthuis 1988) or 1.69 times as costly due to the energy required to produce a queen (Boomsma 1989; Bourke 1997).

no. queens x cost ratio / (no. queens x cost-ratio + no. males)

We then calculated the mean for each cost ratio resulting in values of 0.46 and 0.49 for 1.69 and 2.11 cost-ratios, respectively, which were within the range of empirical results of 0.44-0.51 as reported by Duchateau *et al.* (2004), Duchateau & Velthuis (1988) and Gosterit & Gurel (2016).

Empirical data show a bi-modal pattern where colonies either invest all in queen production (1) or all in male production (0). *Bumble*-BEEHAVE simulated results show a weak but significant bi-modal pattern (Figure SI 12). Significance of bimodality was determined using Hartigan's Dip test statistic for non-unimodality (Hartigan & Hartigan 1985) and the *dip.test* function in the package "diptest" in R (Version 3.2.3). Both empirical and simulated results are significant ($p < 0.05$) with Dip test statistic values of 0.12 for empirical data (both 1.69 and 2.11 cost-ratio factors) and 0.13 and 0.12 (2dp) for simulated data, respectively (1.69 and 2.11 cost-ratio factors, respectively).

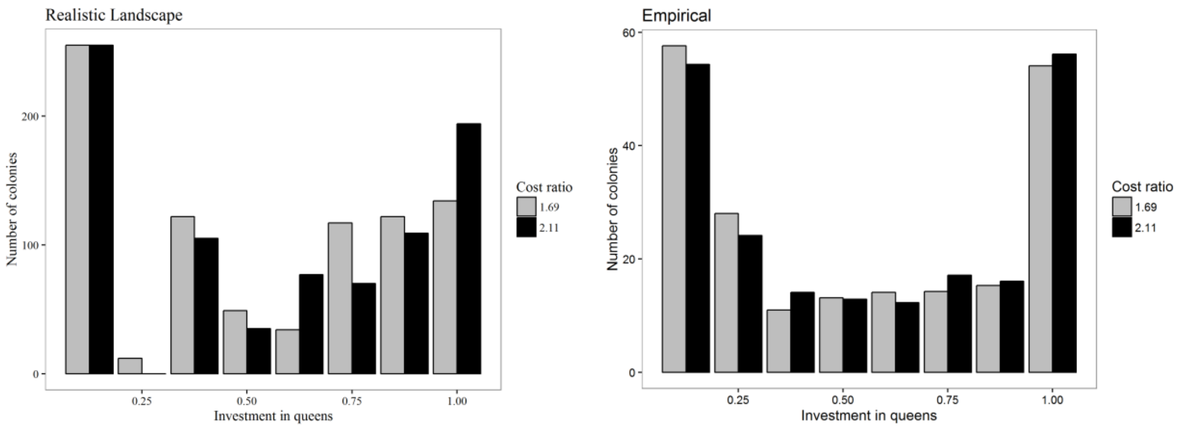

Figure SI 11. Comparison between *Bumble*-BEEHAVE model simulated colonies a realistic landscape “Sussex 1” and empirical data of experimental colonies Overall colony investments in queens based on cost-ratio factors of 1.69 (grey bars) and 2.11 (black bars) from Figure 3 in Duchateau *et al.* (2004). 1 = colony produces all queens, and 0 = colonies produce all males. $p < 0.05$ for Hartigan’s Dip test.

REFERENCES

Boomsma, J.J. (1989). Sex-investment ratios in ants- has female bias been systematically overestimated. *Am. Nat.*, 133, 517-532.

Bourke, A.F.G. (1997). Sex ratios in bumble bees. *Philos T Roy Soc B*, 352, 1921-1932.

Duchateau, M.J. & Velthuis, H.H.W. (1988). Development and Reproductive Strategies in *Bombus* Colonies. *Behaviour*, 107, 186-207.

Duchateau, M.J., Velthuis, H.H.W. & Boomsma, J.J. (2004). Sex ratio variation in the bumblebee *Bombus terrestris*. *Behav. Ecol.*, 15, 71-82.

Gosterit, A. & Gurel, F. (2016). Male remating and its influences on queen colony foundation success in the bumblebee, *Bombus terrestris*. *Apidologie*, 47, 828-834.

Hartigan, J.A. & Hartigan, P.M. (1985). The Dip test of Unimodality. *Annals of Statistics*, 13, 70-84.
